# Supplementary material for: Global climate forcing on late Miocene establishment of the Pampean aeolian system in South America
Source: Nat Commun. 2023 Oct 30;14:6899. doi: 10.1038/s41467-023-42537-3 (PMC10613622; doi:10.1038/s41467-023-42537-3)
Supplement: Supplementary file 1 — Supplementary Information [file 41467_2023_42537_MOESM1_ESM.pdf]

Supplementary Figure S1

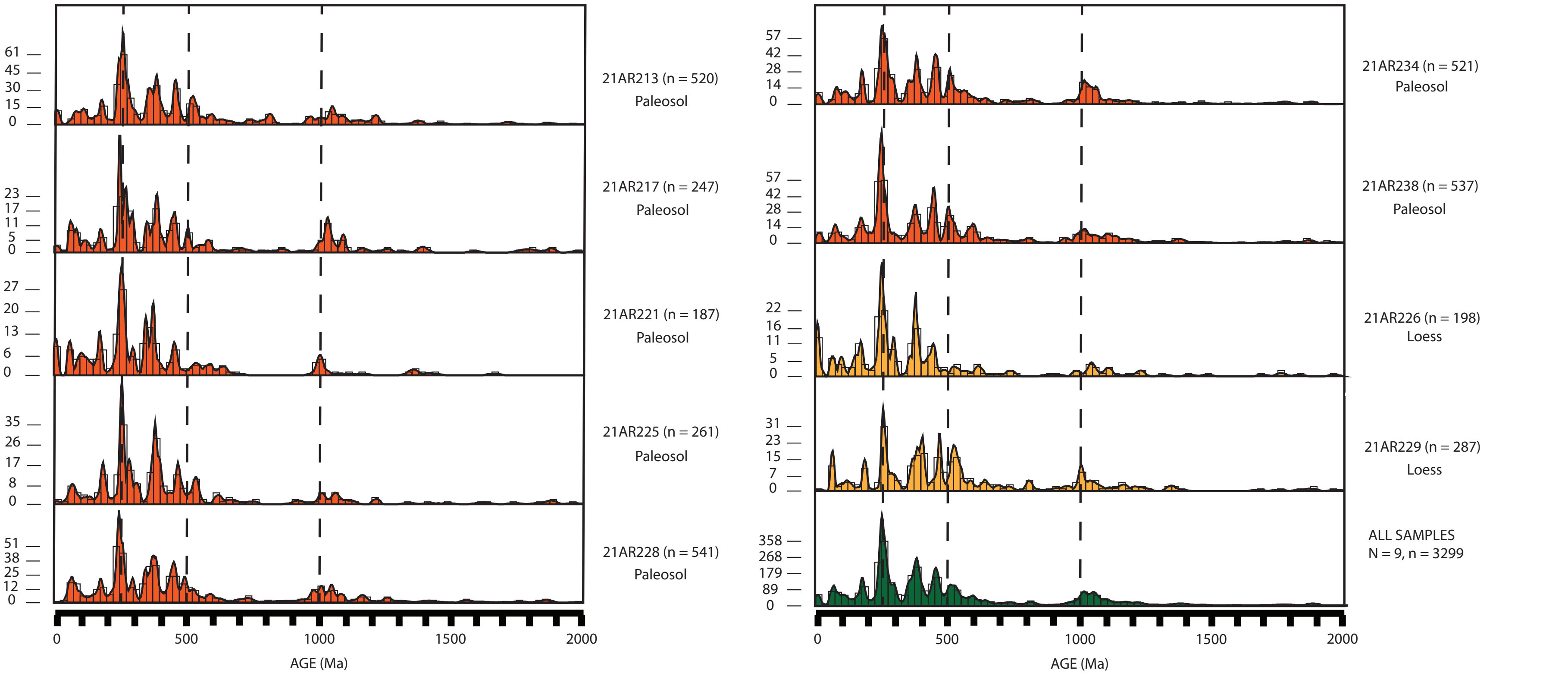

Detrital zircon results from each sample used in this study. Images display the kernal density estimates (in-filled curves) as well as the histogram of detrital zircon ages, collected in 25 myr bins.

Supplementary Figure S1: kernal density estimates for U-Pb detrital zircon ages.

Figure S2

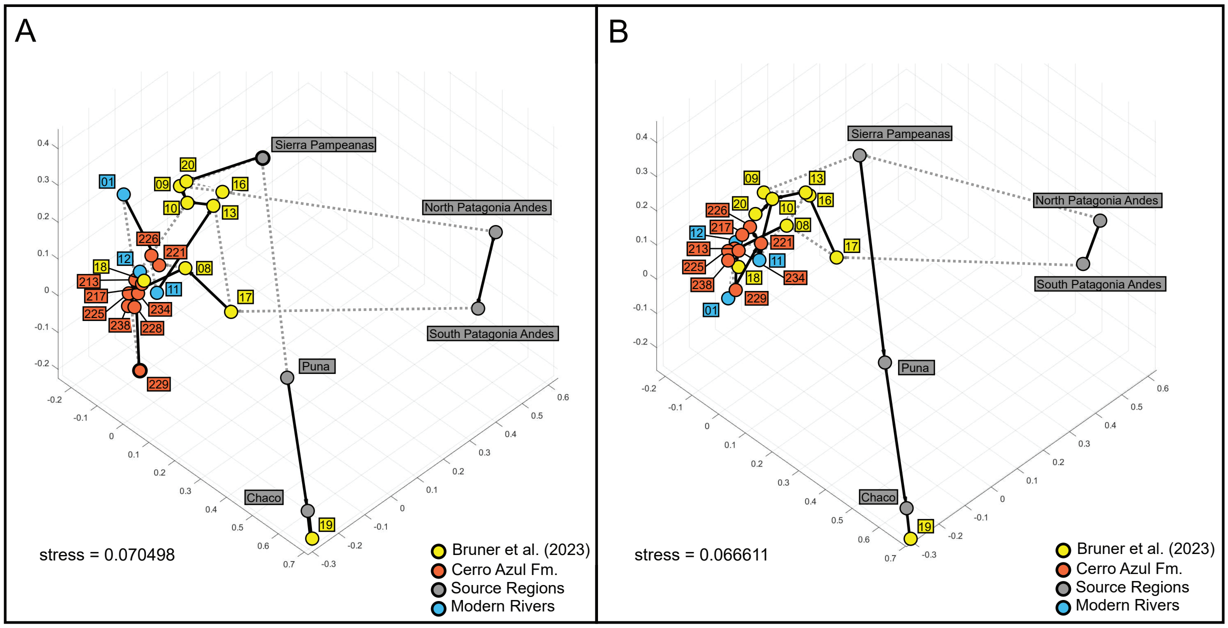

River samples: 1 = Rio Desaguadero; 11 = Rio Negro; 12 = Rio Colorado; 1,11,12 from Bruner et al., 2023
Source regions: Sierras Pampeanas (compiled from Adams et al., 2011; Fosdick et al., 2015, 2017; Capaldi et al., 2017; Reat and Fosdick, 2018); Chaco Plains (compiled from McGlue et al., 2016; Pepper et al., 2016); North and South Patagonian Andes (compiled from Encinas et al., 2014; Leonard et al., 2020); Puna Plateau (Zhou et al., 2016, 2017; Siks and Horton, 2011; DeCelles et al., 2007; Streit et al., 2017; Henriquez et al., 2019, 2020).

A) All data Pleistocene-Holocene and Cerro Azul
B) <5 Ma grains omitted from all samples

Supplementary Figure S1: multidimensional scaling plots of Cerro Azul U-Pb data with comparison data.

Table S1

Sample	Formation	Lithology	Description	Locality	Latitude	Longitude
21AR213	Cerro Azul	Paleosol	Silt to very fine-grained sand, pink to dark brown, poorly-cemented, abundant CaCO3 nodules	Guanaco	-36.3053	-64.27051
21AR217	Cerro Azul	Paleosol	Silt, dark brown, CaCO3-cemented with moderate amounts of CaCO3 nodules.	Telén (lake)	-36.25338	-65.51344
21AR221	Cerro Azul	Paleosol	Silt, medium brown, abundant CaCO3, crumbly texture. Peds visible at exposure.	Type 1 Locality	-36.39974	-67.26686
21AR225	Cerro Azul	Paleosol	Silt, medium brown, abundant CaCO3, crumbly texture. Peds visible at exposure.	Type 1 Locality	-36.39974	-67.26686
21AR226	Cerro Azul	Loess	Silt to very fine-grained sand, light brown, abundant CaCO3 nodules with some clasts exceeding 50 mm, crumbly texture.	Type 1 Locality	-36.39974	-67.26686
21AR228	Cerro Azul	Paleosol	Silt to very fine-grained sand, medium-dark brown, abundant CaCO3 nodules, poorly-cemented.	Bajo Del Carbon (south side)	-36.69971	-65.47549
21AR229	Cerro Azul	Loess	Silt to very fine-grained sand, light-medium brown, absence of CaCO3 nodules, poorly-cemented.	Bajo Del Carbon (south side)	-36.69971	-65.47549
21AR234	Cerro Azul	Paleosol	Silt to very fine-grained sand, medium brown, abundant CaCO3 nodules, poorly-cemented.	Route 13	-36.7356	-65.29059
21AR238	Cerro Azul	Paleosol	Silt to medium-grained sand, light brown to tan, abundant, CaCO3 nodules <25 mm in diameter, crumbly texture, poorly cemented	Estancia La Malvina, Santa Rosa	-36.61736	-64.33147

Type 1 Locality refers to the type section of the Cerro Azul Formation, Algarrobo del Aguila, Argentina, as defined in Visconti et al., 2010

Supplementary Table S1: sample descriptions and locations

Table S2

Analysis	U (ppm)	$^{206}Pb/^{204}Pb$	U/Th	$^{206}Pb^{*}/^{207}Pb^{*}$	±2s (%)	$^{207}Pb^{*}/^{235}U$	±2s (%)	$^{206}Pb^{*}/^{238}U$	±2s (%)	error correlation	$^{206}Pb^{*}/^{238}U$ (Ma)	±2s (myr)	$^{207}Pb^{*}/^{235}U$ (Ma)	±2s (myr)	$^{206}Pb^{*}/^{207}Pb^{*}$ (Ma)	±2s (myr)	[1]Best age (Ma)	±2s (myr)	[2]Concordance (%)
**21AR213**																			
21AR213_1	306.6	1145.974	1.692	14.880	9.8	0.09660	35.4	0.01289	1.7	0.049	82.6	1.4	93.6	31.6	386.5	798.7	82.6	1.4	21.4
21AR213_2	399.1	19667.289	1.649	18.080	2.6	0.46085	3.3	0.06151	1.7	0.524	384.8	6.4	384.8	10.4	386.0	62.3	384.8	6.4	99.7
21AR213_3	138.2	9164.848	1.703	15.675	6.8	0.32205	7.4	0.03784	1.8	0.250	239.5	4.3	283.5	18.2	665.3	152.8	239.5	4.3	36.0
21AR213_4	132.8	16776.640	1.199	18.396	3.9	0.43500	5.0	0.05946	1.8	0.354	372.4	6.4	366.7	15.4	332.2	106.4	372.4	6.4	112.1
21AR213_5	59.5	4506.036	1.068	15.014	9.7	0.32875	10.6	0.03791	3.1	0.290	239.9	7.2	288.6	26.6	705.4	215.8	239.9	7.2	34.0
21AR213_6	189.8	64171.422	2.374	11.262	1.4	2.80816	2.3	0.23170	1.9	0.792	1343.4	22.5	1357.7	17.6	1381.2	27.5	1381.2	27.5	97.3
21AR213_7	170.2	9855.366	2.012	17.840	3.7	0.30151	5.2	0.04035	1.5	0.283	255.0	3.7	267.6	12.2	380.4	111.9	255.0	3.7	67.0
21AR213_8	146.1	17971.216	2.038	17.032	4.0	0.49196	4.4	0.06204	1.6	0.354	388.0	5.9	406.2	14.7	512.1	90.5	388.0	5.9	75.8
21AR213_9	217.7	17897.683	0.942	18.167	4.4	0.29922	4.9	0.04029	1.3	0.264	254.6	3.2	265.8	11.5	366.2	106.6	254.6	3.2	69.5
21AR213_10	763.4	76036.573	27.982	16.884	1.3	0.71563	1.9	0.08852	1.2	0.654	546.8	6.4	548.1	7.9	554.5	30.6	546.8	6.4	98.6
21AR213_11	179.1	14173.377	6.351	14.744	2.1	1.04917	3.0	0.11437	1.2	0.411	698.1	8.3	728.5	15.8	824.0	57.7	698.1	8.3	84.7
21AR213_12	246.7	6789.686	1.578	14.416	1.7	1.12651	3.2	0.12235	2.4	0.758	744.1	17.1	766.1	17.3	831.7	43.7	744.1	17.1	89.5
21AR213_13	451.1	20393.196	3.026	13.467	1.2	1.60365	2.1	0.15925	1.6	0.799	952.6	14.6	971.6	12.9	1015.8	25.1	1015.8	25.1	93.8
21AR213_14	71.6	20253.406	2.724	13.820	3.2	1.62284	4.2	0.16543	2.2	0.529	986.9	20.4	979.1	26.5	962.5	73.1	962.5	73.1	102.5
21AR213_15	147.8	9874.770	1.139	17.579	6.0	0.25812	7.8	0.03403	2.3	0.298	215.7	4.9	233.1	16.1	413.8	165.4	215.7	4.9	52.1
21AR213_16	340.1	21110.845	3.337	19.129	4.3	0.20096	5.4	0.02845	2.2	0.413	180.8	4.0	185.9	9.1	252.6	112.8	180.8	4.0	71.6
21AR213_17	227.9	24217.492	4.493	14.500	2.5	1.27807	3.6	0.13642	2.2	0.599	824.4	16.9	836.0	20.7	867.8	60.4	824.4	16.9	95.0
21AR213_18	67.5	16752.785	1.411	12.900	2.8	1.94942	3.2	0.18556	1.4	0.420	1097.3	13.7	1098.2	21.8	1101.0	58.8	1101.0	58.8	99.7
21AR213_19	761.9	8083.219	1.342	18.503	1.8	0.32064	2.6	0.04480	1.6	0.612	282.5	4.4	282.4	6.4	282.3	46.8	282.5	4.4	100.1
21AR213_20	133.4	16635.028	1.408	17.328	3.5	0.58561	3.8	0.07520	1.4	0.356	467.4	6.1	468.1	14.3	472.2	79.0	467.4	6.1	99.0
21AR213_21	141.8	26698.854	3.191	13.335	1.5	1.76820	2.4	0.17297	1.2	0.520	1028.4	11.9	1033.9	15.5	1046.3	41.3	1046.3	41.3	98.3
21AR213_22	305.5	44510.701	28.954	16.717	2.0	0.72410	4.2	0.08873	3.7	0.880	548.1	19.4	553.1	17.9	574.8	43.4	548.1	19.4	95.3
21AR213_23	174.2	6793.344	2.445	17.044	3.3	0.45017	7.8	0.05802	6.7	0.863	363.5	23.8	377.4	24.6	464.3	87.5	363.5	23.8	78.3
21AR213_24	97.0	7394.867	0.435	16.870	6.5	0.32328	7.3	0.04112	2.3	0.321	259.8	5.9	284.4	18.0	493.1	151.9	259.8	5.9	52.7
21AR213_25	362.1	5388.228	1.660	16.623	6.9	0.13339	8.6	0.01693	1.7	0.194	108.2	1.8	127.1	10.3	498.6	186.0	108.2	1.8	21.7
21AR213_26	60.9	8453.999	0.542	16.193	6.0	0.59911	7.3	0.07274	1.9	0.262	452.7	8.3	476.7	27.6	594.9	151.7	452.7	8.3	76.1
21AR213_27	450.8	54327.597	4.161	14.611	1.4	1.13340	2.8	0.12110	2.3	0.845	736.9	16.3	769.4	15.0	865.7	30.8	736.9	16.3	85.1
21AR213_28	345.3	11050.213	6.335	15.489	1.6	0.85474	2.2	0.09845	1.4	0.621	605.3	8.0	627.2	10.4	708.1	36.9	605.3	8.0	85.5
21AR213_29	242.8	6225.750	1.416	18.613	6.1	0.17590	10.3	0.02494	1.8	0.177	158.8	2.9	164.5	15.7	249.2	234.6	158.8	2.9	63.7
21AR213_30	101.1	41572.276	2.611	11.139	1.4	2.97425	2.1	0.24229	1.5	0.712	1398.6	18.7	1401.1	15.9	1405.6	28.1	1405.6	28.1	99.5
21AR213_31	75.3	2394.020	1.412	9.613	21.5	0.25598	23.1	0.01898	3.1	0.135	121.2	3.7	231.4	47.7	1583.5	428.6	121.2	3.7	7.7
21AR213_32	102.8	11648.694	1.535	15.501	8.6	0.38884	9.3	0.04478	1.9	0.201	282.4	5.2	333.5	26.5	708.6	194.0	282.4	5.2	39.8
21AR213_33	63.8	4975.341	0.605	15.299	8.1	0.48337	10.8	0.05639	2.1	0.198	353.6	7.4	400.4	35.7	680.8	225.8	353.6	7.4	51.9
21AR213_34	178.0	13901.044	1.648	17.902	4.6	0.30662	5.3	0.04074	1.8	0.333	257.4	4.4	271.6	12.5	396.1	111.2	257.4	4.4	65.0
21AR213_35	240.2	16797.171	2.855	17.194	2.8	0.59799	4.2	0.07601	1.8	0.435	472.3	8.4	476.0	16.1	494.7	83.9	472.3	8.4	95.5
21AR213_36	112.3	7391.452	1.700	16.228	6.8	0.30942	7.3	0.03777	1.8	0.248	239.0	4.3	273.7	17.6	583.2	154.3	239.0	4.3	41.0
21AR213_37	110.5	8969.256	1.314	17.274	6.0	0.31594	7.4	0.04089	1.9	0.256	258.4	4.8	278.8	18.0	454.6	158.2	258.4	4.8	56.8
21AR213_38	119.4	11071.865	1.227	17.115	4.3	0.48846	4.9	0.06230	1.8	0.375	389.6	7.0	403.9	16.4	487.2	100.6	389.6	7.0	80.0
21AR213_39	348.2	48489.288	14.299	13.762	1.6	1.52070	3.3	0.15303	2.9	0.856	917.9	24.5	938.8	20.5	988.9	35.2	988.9	35.2	92.8
21AR213_40	132.8	11343.159	1.451	17.097	5.5	0.44204	5.9	0.05627	1.3	0.217	352.9	4.4	371.7	18.3	491.4	126.3	352.9	4.4	71.8
21AR213_41	89.3	10713.460	10.387	13.650	3.1	1.55692	3.5	0.15764	1.2	0.344	943.6	10.5	953.2	21.5	976.4	66.6	976.4	66.6	96.6
21AR213_42	238.2	8330.609	2.887	18.080	4.8	0.20199	5.9	0.02749	1.9	0.329	174.8	3.3	186.8	10.0	342.1	125.7	174.8	3.3	51.1
21AR213_43	310.3	25393.986	1.608	18.300	2.9	0.41226	3.9	0.05552	1.7	0.430	348.3	5.7	350.5	11.5	365.8	78.8	348.3	5.7	95.2
21AR213_44	194.5	48667.769	2.775	13.399	1.9	1.47814	5.3	0.14479	5.0	0.928	871.7	40.4	921.5	32.3	1043.4	40.2	1043.4	40.2	83.5
21AR213_45	271.2	6199.352	0.764	17.248	2.8	0.47760	3.4	0.06252	1.8	0.545	390.9	6.9	396.4	11.0	429.5	62.7	390.9	6.9	91.0
21AR213_46	135.6	32967.795	3.803	14.037	2.4	1.28768	4.1	0.13243	3.2	0.785	801.7	24.2	840.3	23.3	944.5	51.8	801.7	24.2	84.9
21AR213_47	274.1	34238.881	1.623	18.125	2.8	0.41994	3.6	0.05583	1.6	0.440	350.2	5.3	356.0	10.7	395.0	71.6	350.2	5.3	88.7
21AR213_48	181.0	25330.368	3.031	16.448	2.7	0.71902	3.2	0.08697	1.7	0.525	537.6	8.8	550.1	13.8	603.1	59.7	537.6	8.8	89.1
21AR213_49	257.9	75753.018	3.488	13.336	1.6	1.63523	2.4	0.15910	1.8	0.740	951.8	15.8	983.9	15.2	1057.1	32.8	1057.1	32.8	90.0
21AR213_50	222.2	9466.253	1.090	18.349	3.0	0.28917	4.5	0.03974	1.7	0.368	251.2	4.1	257.9	10.2	320.0	95.0	251.2	4.1	78.5
21AR213_51	624.4	37444.103	0.764	19.467	1.8	0.26761	2.6	0.03818	1.4	0.560	241.5	3.4	240.8	5.5	234.4	49.4	241.5	3.4	103.0
21AR213_52	193.2	2243.281	0.488	16.275	7.4	0.30161	23.3	0.03996	2.2	0.092	252.6	5.3	267.7	54.9	402.7	522.0	252.6	5.3	62.7
21AR213_53	275.5	19877.690	2.501	16.702	1.8	0.69646	3.0	0.08571	1.5	0.496	530.1	7.6	536.7	12.6	565.5	57.2	530.1	7.6	93.7
21AR213_54	159.4	77628.761	1.457	8.695	1.3	5.10072	2.0	0.32330	1.5	0.755	1805.8	23.8	1836.2	17.0	1871.7	23.6	1871.7	23.6	96.5
21AR213_55	140.9	8586.947	4.758	14.115	2.5	1.27825	5.1	0.13449	2.3	0.460	813.4	17.9	836.1	29.0	897.6	93.3	813.4	17.9	90.6
21AR213_56	676.3	27555.106	35.086	17.120	1.7	0.55654	3.8	0.06993	3.4	0.888	435.7	14.1	449.3	13.7	520.3	38.0	435.7	14.1	83.7
21AR213_57	136.5	24609.347	2.589	15.085	2.2	1.09827	2.8	0.12166	1.6	0.584	740.1	11.4	752.5	14.8	790.5	47.6	740.1	11.4	93.6
21AR213_58	55.7	4394.470	1.884	13.706	11.5	0.45233	12.4	0.04723	2.5	0.200	297.5	7.2	378.9	39.2	913.1	250.0	297.5	7.2	32.6
21AR213_59	87.6	7821.973	2.521	14.366	12.3	0.38373	13.4	0.04123	1.6	0.123	260.5	4.2	329.8	37.8	854.1	277.1	260.5	4.2	30.5
21AR213_60	261.6	27794.560	2.889	17.888	1.9	0.55481	2.5	0.07300	1.4	0.555	454.2	6.0	448.1	8.9	418.1	45.7	454.2	6.0	108.6
21AR213_61	57.9	1775.055	1.107	9.818	22.4	0.20080	23.6	0.01556	4.1	0.175	99.5	4.1	185.8	40.1	1500.5	440.6	99.5	4.1	6.6
21AR213_62	764.4	29042.017	5.119	14.414	1.5	1.29663	3.9	0.13716	3.5	0.894	828.6	27.2	844.2	22.4	886.5	36.2	828.6	27.2	93.5
21AR213_63	144.8	4999.554	1.029	15.111	11.1	0.14554	14.0	0.01676	2.7	0.193	107.1	2.9	138.0	18.1	708.7	292.5	107.1	2.9	15.1
21AR213_64	375.0	32157.594	1.578	18.269	2.2	0.42615	3.2	0.05724	1.8	0.573	358.8	6.4	360.4	9.7	371.9	58.8	358.8	6.4	96.5
21AR213_65	185.8	4434.800	1.457	17.217	6.5	0.20265	13.8	0.02695	1.8	0.131	171.4	3.1	187.4	23.5	394.6	306.1	171.4	3.1	43.4
21AR213_66	192.7	27332.169	1.684	17.023	3.5	0.59064	3.9	0.07400	1.7	0.426	460.2	7.4	471.3	14.8	526.5	78.0	460.2	7.4	87.4
21AR213_67	226.8	8929.447	1.551	17.960	4.4	0.29615	7.4	0.03997	2.1	0.278	252.6	5.1	263.4	17.2	361.1	160.4	252.6	5.1	70.0
21AR213_68	277.7	83244.086	4.955	13.654	1.2	1.67873	1.9	0.16757	1.4	0.740	998.7	12.9	1000.5	12.0	1005.3	25.8	1005.3	25.8	99.4
21AR213_69	371.6	122303.232	2.065	12.735	1.2	2.03342	1.9	0.18915	1.5	0.790	1116.8	15.4	1126.8	13.0	1146.9	23.2	1146.9	23.2	97.4
21AR213_70	268.4	17374.283	2.374	18.184	2.5	0.45536	3.6	0.06135	1.2	0.350	383.8	4.6	381.0	11.3	365.1	75.0	383.8	4.6	105.1
21AR213_71	45.7	4867.976	1.152	14.313	14.3	0.44165	15.9	0.04814	2.6	0.165	303.1	7.8	371.4	49.5	824.2	328.0	303.1	7.8	36.8
21AR213_72	104.1	35710.357	1.337	9.379	1.4	4.16329	2.0	0.28581	1.4	0.688	1620.5	20.2	1666.8	16.7	1726.4	27.2	1726.4	27.2	93.9
21AR213_73	194.1	6278.210	3.060	12.234	1.6	1.84254	6.1	0.16907	5.4	0.886	1007.0	50.3	1060.8	40.1	1174.0	55.8	1174.0	55.8	85.8
21AR213_74	95.4	6516.698	1.571	16.504	6.6	0.34597	8.5	0.04325	2.1	0.240	272.9	5.5	301.7	22.3	531.5	181.6	272.9	5.5	51.4
21AR213_75	226.9	33697.903	20.635	17.154	2.6	0.67263	3.0	0.08468	1.4	0.454	524.0	6.8	522.3	12.2	515.9	58.4	524.0	6.8	101.6
21AR213_76	232.2	3702.239	2.237	14.784	10.7	0.09140	13.3	0.01046	2.3	0.171	67.1	1.5	88.8	11.3	721.0	277.8	67.1	1.5	9.3
21AR213_77	236.4	23245.013	1.654	18.482	2.9	0.41746	3.4	0.05696	1.6	0.478	357.1	5.6	354.2	10.1	336.5	67.4	357.1	5.6	106.1
21AR213_78	650.8	126660.955	15.960	6.739	2.1	7.45427	4.3	0.36690	3.8	0.875	2014.8	65.1	2167.5	38.5	2316.1	35.7	2316.1	35.7	87.0
21AR213_79	334.6	17969.052	1.424	19.135	2.9	0.26675	4.1	0.03780	1.5	0.357	239.2	3.5	240.1	8.8	249.9	88.7	239.2	3.5	95.7
21AR213_80	214.6	2933.928	1.619	12.318	14.8	0.10760	16.7	0.01028	2.1	0.123	65.9	1.3	103.8	16.5	1094.3	333.3	65.9	1.3	6.0
21AR213_81	129.5	60366.070	1.556	8.198	1.1	5.85951	2.0	0.35083	1.7	0.822	1938.5	27.8	1955.3	17.5	1973.8	20.5	1973.8	20.5	98.2
21AR213_82	238.5	6964.221	1.591	17.649	5.6	0.20861	6.5	0.02786	1.9	0.286	177.1	3.2	192.4	11.4	384.8	140.0	177.1	3.2	46.0
21AR213_83	182.0	2936.816	1.791	16.405	4.2	0.48164	16.2	0.06268	2.0	0.126	391.9	7.7	399.2	53.5	442.6	358.2	391.9	7.7	88.6
21AR213_84	91.8	9105.414	2.420	15.835	8.4	0.36653	8.9	0.04339	2.1	0.236	273.8	5.6	317.1	24.2	649.6	185.3	273.8	5.6	42.1
21AR213_85	327.2	123495.120	37.214	12.377	1.4	2.16046	3.9	0.19526	3.6	0.929	1149.8	38.0	1168.4	26.9	1203.9	28.2	1203.9	28.2	95.5
21AR213_86	145.2	24077.488	3.135	17.287	3.5	0.64467	4.0	0.08207	1.5	0.374	508.5	7.3	505.2	15.9	491.3	81.9	508.5	7.3	103.5
21AR213_87	921.5	4999.158	2.973	15.448	10.4	0.02712	12.1	0.00320	2.0	0.167	20.6	0.4	27.2	3.2	659.7	255.1	20.6	0.4	3.1
21AR213_88	175.3	20573.991	3.524	17.332	3.0	0.65423	3.5	0.08357	1.4	0.396	517.4	6.9	511.1	14.0	483.8	70.9	517.4	6.9	107.0
21AR213_89	231.6	33820.100	4.891	16.986	2.4	0.72780	4.2	0.09066	3.4	0.819	559.5	18.4	555.3	18.0	539.0	52.7	559.5	18.4	103.8
21AR213_90	194.5	5739.536	1.573	17.346	7.3	0.14579	8.2	0.01926	1.7	0.204	123.0	2.0	138.2	10.6	408.9	180.6	123.0	2.0	30.1
21AR213_91	153.5	17973.201	1.239	17.056	4.8	0.48521	5.8	0.06109	1.8	0.310	382.3	6.7	401.6	19.2	515.6	121.0	382.3	6.7	74.1
21AR213_92	155.7	13059.260	1.130	16.925	4.7	0.31276	6.2	0.03928	1.6	0.256	248.4	3.9	276.3	15.1	521.2	132.5	248.4	3.9	47.6
21AR213_93	196.3	2519.368	1.046	16.436	5.5	0.30653	14.8	0.04055	2.4	0.161	256.3	6.0	271.5	35.3	405.8	327.8	256.3	6.0	63.2
21AR213_94	288.2	639.243	1.884	4.000	36.7	0.02566	40.5	0.00080	16.6	0.409	5.2	0.9	25.7	10.3	3066.1	596.4	5.2	0.9	0.2
21AR213_95	652.3	11110.431	0.781	19.128	5.1	0.07682	5.6	0.01098	1.5	0.259	70.4	1.0	75.2	4.1	229.7	125.9	70.4	1.0	30.7
21AR213_96	110.4	3784.646	1.723	13.811	13.7	0.18685	14.6	0.01984	3.1	0.211	126.6	3.8	173.9	23.3	879.1	295.0	126.6	3.8	14.4
21AR213_97	184.0	5595.190	2.250	14.931	9.4	0.17370	12.2	0.01965	2.1	0.170	125.4	2.6	162.6	18.4	746.5	255.3	125.4	2.6	16.8
21AR213_98	181.1	13101.243	0.894	18.545	3.7	0.29780	4.5	0.04108	1.9	0.416	259.5	4.8	264.7	10.6	311.8	94.0	259.5	4.8	83.2
21AR213_99	116.6	41195.643	0.796	12.220	1.9	2.25631	2.6	0.20167	1.6	0.636	1184.3	17.6	1198.8	18.0	1225.7	38.7	1225.7	38.7	96.6
21AR213_100	393.0	55426.194	29.829	17.785	2.0	0.55313	2.8	0.07196	1.8	0.663	448.0	7.9	447.0	10.0	443.2	46.0	448.0	7.9	101.1
21AR213_101	128.9	7571.220	1.046	16.845	5.4	0.44763	7.7	0.05672	1.7	0.220	355.7	5.9	375.6	24.3	501.5	166.2	355.7	5.9	70.9
21AR213_102	67.9	19361.596	2.915	13.223	3.2	1.88789	3.6	0.18350	1.7	0.475	1086.1	17.3	1076.8	24.2	1059.1	64.6	1059.1	64.6	102.5
21AR213_103	147.6	620.536	1.676	5.005	51.7	0.05483	55.1	0.00222	13.1	0.237	14.3	1.9	54.2	29.1	2647.3	906.4	14.3	1.9	0.5
21AR213_104	100.7	11541.207	1.732	16.920	6.6	0.45139	7.4	0.05681	2.1	0.281	356.2	7.2	378.3	23.3	516.7	155.7	356.2	7.2	68.9
21AR213_105	117.5	13311.361	1.141	16.903	4.3	0.60080	5.2	0.07533	1.4	0.276	468.2	6.5	477.7	19.8	524.7	109.4	468.2	6.5	89.2
21AR213_106	307.8	10119.825	0.547	18.163	4.6	0.21145	5.8	0.02873	1.4	0.251	182.6	2.6	194.8	10.2	345.7	126.0	182.6	2.6	52.8
21AR213_107	1915.9	2726.730	1.307	12.741	13.2	0.00963	14.4	0.00096	3.1	0.213	6.2	0.2	9.7	1.4	1013.0	284.6	6.2	0.2	0.6
21AR213_108	187.7	22427.384	1.956	17.675	3.5	0.56460	4.4	0.07353	1.7	0.385	457.4	7.4	454.5	15.9	441.0	89.4	457.4	7.4	103.7
21AR213_109	771.6	5700.846	1.639	17.908	2.0	0.35005	2.4	0.04784	1.3	0.538	301.3	3.9	304.8	6.4	332.6	46.8	301.3	3.9	90.6
21AR213_110	499.5	19731.560	4.250	17.336	1.7	0.59349	3.5	0.07589	2.2	0.625	471.5	10.0	473.1	13.4	481.7	60.9	471.5	10.0	97.9

21AR213_111	157.7	52987.429	2.452	13.870	2.0	1.49020	4.8	0.15107	4.3	0.898	906.9	36.5	926.4	29.2	973.9	43.1	973.9	43.1	93.1
21AR213_112	95.1	5259.672	1.234	13.785	12.1	0.33041	12.8	0.03446	1.9	0.152	218.4	4.2	289.9	32.4	915.8	261.4	218.4	4.2	23.8
21AR213_113	403.6	25681.983	2.029	18.143	2.4	0.44220	3.3	0.05903	1.5	0.452	369.7	5.4	371.8	10.4	385.9	66.6	369.7	5.4	95.8
21AR213_114	125.6	7205.578	1.120	16.531	6.7	0.31142	9.0	0.03879	1.6	0.176	245.3	3.8	275.3	21.6	539.2	193.0	245.3	3.8	45.5
21AR213_115	264.6	20532.874	1.255	18.027	2.2	0.44436	2.9	0.05908	1.5	0.516	370.0	5.3	373.3	8.9	394.7	55.0	370.0	5.3	93.8
21AR213_116	93.7	1303.093	2.335	9.360	18.2	0.13002	23.5	0.00984	6.2	0.263	63.1	3.9	124.1	27.4	1545.3	427.1	63.1	3.9	4.1
21AR213_117	180.8	56193.691	2.460	13.367	1.4	1.72155	2.0	0.16820	1.3	0.667	1002.2	12.1	1016.6	12.6	1048.7	29.5	1048.7	29.5	95.6
21AR213_118	366.1	27745.974	7.995	16.646	3.4	0.53080	6.5	0.06490	5.3	0.810	405.3	20.8	432.3	23.0	579.8	83.2	405.3	20.8	69.9
21AR213_119	243.4	8236.644	0.799	17.328	5.4	0.22649	6.8	0.02948	1.8	0.274	187.3	3.4	207.3	12.7	442.2	144.5	187.3	3.4	42.4
21AR213_120	70.3	7054.658	0.449	16.451	5.9	0.52894	6.4	0.06558	1.4	0.226	409.5	5.7	431.1	22.5	549.5	136.3	409.5	5.7	74.5
21AR213_121	367.5	5049.715	1.514	16.659	1.9	0.55727	3.4	0.07100	1.5	0.452	442.2	6.5	449.7	12.2	489.7	66.2	442.2	6.5	90.3
21AR213_122	329.8	12870.576	2.067	13.329	1.5	1.73551	2.9	0.17090	1.3	0.457	1017.1	12.4	1021.8	18.5	1032.8	51.8	1032.8	51.8	98.5
21AR213_123	118.4	1333.897	0.642	10.918	21.7	0.14210	28.4	0.01277	3.9	0.138	81.8	3.2	134.9	35.9	1214.9	556.7	81.8	3.2	6.7
21AR213_124	414.6	26642.177	2.398	18.824	2.5	0.34509	3.3	0.04778	1.5	0.460	300.9	4.5	301.0	8.6	303.1	66.7	300.9	4.5	99.3
21AR213_125	114.7	18033.277	2.645	13.576	2.2	1.65256	3.3	0.16508	2.2	0.671	985.0	19.9	990.5	20.6	1003.8	48.9	1003.8	48.9	98.1
21AR213_126	322.2	5640.777	0.884	17.633	4.9	0.18245	7.4	0.02455	1.4	0.186	156.3	2.1	170.2	11.6	368.1	163.3	156.3	2.1	42.5
21AR213_127	25.1	7588.648	1.089	13.650	7.6	1.67456	8.0	0.17089	1.5	0.181	1017.0	13.7	998.9	51.2	960.3	161.7	960.3	161.7	105.9
21AR213_128	235.8	35220.384	1.957	17.444	2.5	0.67127	3.1	0.08586	1.8	0.561	531.0	8.9	521.5	12.7	480.9	57.2	531.0	8.9	110.4
21AR213_129	209.6	5917.894	2.170	14.906	11.3	0.12256	13.2	0.01380	4.9	0.374	88.4	4.3	117.4	14.6	755.9	258.4	88.4	4.3	11.7
21AR213_130	454.1	23900.669	2.131	19.014	3.0	0.19994	3.3	0.02800	1.3	0.392	178.0	2.3	185.1	5.7	277.0	70.6	178.0	2.3	64.3
21AR213_131	219.0	31111.486	2.931	17.050	2.3	0.69065	2.9	0.08641	1.7	0.565	534.3	8.5	533.2	12.2	529.5	53.1	534.3	8.5	100.9
21AR213_132	711.7	25160.926	0.416	19.282	2.9	0.11055	3.4	0.01569	1.5	0.449	100.4	1.5	106.5	3.5	246.1	70.6	100.4	1.5	40.8
21AR213_133	174.0	15512.241	1.233	9.488	1.2	3.88571	2.4	0.27066	1.6	0.699	1544.1	22.7	1610.7	19.1	1699.7	31.1	1699.7	31.1	90.9
21AR213_134	268.1	23477.442	2.232	18.754	3.4	0.29613	4.2	0.04091	1.8	0.421	258.5	4.5	263.4	9.8	308.2	87.2	258.5	4.5	83.9
21AR213_135	87.0	10145.432	0.515	16.599	5.8	0.51582	6.7	0.06385	1.9	0.277	399.0	7.2	422.4	23.2	552.9	140.8	399.0	7.2	72.2
21AR213_136	250.1	27560.432	0.839	17.727	2.8	0.56320	3.3	0.07337	1.4	0.421	456.4	6.0	453.6	11.9	440.3	65.6	456.4	6.0	103.7
21AR213_137	138.3	20292.767	1.321	17.407	3.6	0.59136	4.9	0.07587	2.5	0.511	471.4	11.5	471.7	18.6	474.2	93.7	471.4	11.5	99.4
21AR213_138	436.3	21644.996	6.325	16.587	1.6	0.75557	2.1	0.09223	1.4	0.644	568.7	7.4	571.5	9.3	583.3	35.3	568.7	7.4	97.5
21AR213_139	312.0	4418.119	12.636	17.156	3.6	0.50314	10.3	0.06664	2.4	0.233	415.9	9.7	413.8	35.0	403.5	224.2	415.9	9.7	103.1
21AR213_140	429.6	51197.390	3.164	18.340	2.0	0.47352	2.6	0.06358	1.4	0.556	397.4	5.5	393.6	8.4	372.7	48.4	397.4	5.5	106.6
21AR213_141	277.4	17360.886	1.739	18.437	3.6	0.28686	4.3	0.03914	1.5	0.346	247.5	3.6	256.1	9.8	336.3	92.2	247.5	3.6	73.6
21AR213_142	257.9	24895.672	1.268	18.417	3.7	0.30689	4.2	0.04163	1.6	0.383	262.9	4.2	271.8	10.1	349.7	88.4	262.9	4.2	75.2
21AR213_143	135.4	54704.021	1.818	10.874	1.3	3.09701	2.0	0.24604	1.5	0.736	1418.0	18.8	1432.0	15.4	1453.6	25.9	1453.6	25.9	97.6
21AR213_144	135.0	3309.732	0.992	13.142	10.4	0.12371	11.8	0.01256	3.3	0.278	80.5	2.6	118.4	13.2	971.1	231.3	80.5	2.6	8.3
21AR213_145	102.9	57118.165	1.613	9.675	1.4	3.98358	2.1	0.28144	1.5	0.731	1598.6	21.8	1630.9	17.1	1673.5	26.5	1673.5	26.5	95.5
21AR213_146	388.5	32521.974	0.991	17.630	1.9	0.55047	2.7	0.07132	1.5	0.565	444.1	6.5	445.3	9.6	452.5	48.8	444.1	6.5	98.1
21AR213_147	411.1	67536.008	1.085	17.616	1.6	0.56884	2.3	0.07335	1.5	0.655	456.3	6.7	457.3	8.6	463.2	39.0	456.3	6.7	98.5
21AR213_148	123.4	441.805	2.104	6.900	39.1	0.03677	53.8	0.00235	17.0	0.317	15.1	2.6	36.7	19.4	1860.3	937.4	15.1	2.6	0.8
21AR213_149	446.8	29999.925	2.277	18.960	2.5	0.27534	3.0	0.03835	1.2	0.422	242.6	3.0	247.0	6.5	289.6	61.2	242.6	3.0	83.8
21AR213_150	421.0	40953.237	1.596	18.174	2.3	0.45278	3.2	0.06039	2.2	0.668	378.0	7.9	379.2	10.2	387.5	53.9	378.0	7.9	97.5
21AR213_151	186.8	42056.163	1.685	12.725	1.6	2.02400	2.3	0.18861	1.7	0.715	1113.8	17.0	1123.6	15.8	1143.4	32.3	1143.4	32.3	97.4
21AR213_152	255.9	32150.956	2.075	17.659	2.4	0.58258	3.1	0.07557	1.8	0.561	469.6	8.0	466.1	11.7	449.9	57.6	469.6	8.0	104.4
21AR213_153	128.8	7373.861	1.389	17.415	6.2	0.21225	7.8	0.02792	2.1	0.267	177.5	3.6	195.4	13.8	419.1	167.1	177.5	3.6	42.4
21AR213_154	209.2	62830.445	5.711	12.272	1.3	2.22369	2.1	0.19953	1.6	0.767	1172.8	17.1	1188.5	14.5	1218.1	26.2	1218.1	26.2	96.3
21AR213_155	416.3	60076.936	1.528	17.519	1.8	0.57234	2.6	0.07342	1.9	0.710	456.7	8.3	459.5	9.8	474.6	41.2	456.7	8.3	96.2
21AR213_156	656.9	12598.994	1.243	19.024	1.6	0.27336	2.1	0.03871	1.3	0.590	244.8	3.0	245.4	4.7	251.9	39.8	244.8	3.0	97.2
21AR213_157	240.3	29496.995	3.155	16.326	2.4	0.79884	3.8	0.09577	2.8	0.744	589.6	15.8	596.2	17.0	622.2	54.5	589.6	15.8	94.8
21AR213_158	129.6	18362.743	2.070	16.389	3.6	0.83755	4.0	0.10130	1.3	0.334	622.0	7.8	617.8	18.3	603.2	80.7	622.0	7.8	103.1
21AR213_159	62.6	5773.223	0.829	14.831	8.8	0.39320	11.6	0.04411	2.2	0.194	278.3	6.1	336.7	33.1	763.7	239.2	278.3	6.1	36.4
21AR213_160	231.7	52613.422	1.551	13.046	1.4	1.80556	2.1	0.17221	1.5	0.712	1024.3	14.1	1047.5	13.6	1097.1	29.3	1097.1	29.3	93.4
21AR213_161	58.4	8722.568	1.652	16.102	7.3	0.69620	8.0	0.08383	2.0	0.247	519.0	9.9	536.5	33.5	612.8	168.1	519.0	9.9	84.7
21AR213_162	327.9	27560.236	2.156	18.704	3.6	0.27745	4.2	0.03813	1.5	0.366	241.2	3.6	248.6	9.2	320.0	87.9	241.2	3.6	75.4
21AR213_163	95.1	3580.268	1.076	15.732	5.1	0.79297	12.8	0.09692	1.8	0.142	596.3	10.3	592.9	57.5	580.5	275.7	596.3	10.3	102.7
21AR213_164	63.5	5556.981	1.361	15.608	9.3	0.40104	11.2	0.04747	2.1	0.189	299.0	6.2	342.4	32.6	649.8	236.5	299.0	6.2	46.0
21AR213_165	500.6	103946.635	7.678	14.707	1.4	1.12309	2.8	0.12041	2.4	0.872	732.9	16.9	764.5	15.0	858.6	28.4	732.9	16.9	85.4
21AR213_166	128.3	19011.690	0.799	17.533	4.3	0.49434	4.8	0.06391	1.8	0.378	399.4	7.0	407.9	16.2	457.1	98.8	399.4	7.0	87.4
21AR213_167	134.4	44754.686	2.164	11.483	1.5	2.52549	3.0	0.21182	2.6	0.869	1238.5	29.8	1279.4	22.1	1349.6	29.0	1349.6	29.0	91.8
21AR213_168	177.5	20167.617	1.524	17.542	3.6	0.48163	4.1	0.06231	1.7	0.413	389.6	6.5	399.2	13.6	455.8	83.5	389.6	6.5	85.5
21AR213_169	170.8	14166.535	1.271	17.727	4.9	0.30821	5.7	0.04052	2.0	0.345	256.1	5.0	272.8	13.7	419.8	120.4	256.1	5.0	61.0
21AR213_170	376.2	14056.210	0.831	18.670	3.2	0.27958	3.6	0.03873	1.4	0.380	244.9	3.3	250.3	8.1	302.1	76.9	244.9	3.3	81.1
21AR213_171	156.4	1724.744	1.374	15.983	6.0	0.30152	21.2	0.04060	1.5	0.072	256.6	3.8	267.6	49.9	366.1	477.6	256.6	3.8	70.1
21AR213_172	157.9	15845.986	1.086	18.207	5.9	0.30908	6.2	0.04169	1.6	0.253	263.3	4.0	273.5	14.8	362.3	135.1	263.3	4.0	72.7
21AR213_173	137.0	14266.107	0.652	17.703	4.3	0.45024	4.9	0.05913	1.5	0.307	370.3	5.5	377.4	15.6	422.4	105.0	370.3	5.5	87.7
21AR213_174	165.1	12035.478	1.051	17.926	5.0	0.33779	5.5	0.04509	1.6	0.296	284.3	4.6	295.5	14.2	386.0	119.1	284.3	4.6	73.7
21AR213_175	408.9	62082.326	127.036	17.711	1.7	0.56864	2.2	0.07373	1.5	0.667	458.6	6.6	457.1	8.3	450.8	37.2	458.6	6.6	101.7
21AR213_176	315.1	32102.559	1.233	17.484	2.2	0.58308	2.7	0.07493	1.4	0.511	465.8	6.3	466.4	10.3	470.7	52.3	465.8	6.3	98.9
21AR213_177	186.5	15760.166	1.675	17.608	5.0	0.33496	5.4	0.04372	1.5	0.282	275.8	4.1	293.3	13.8	436.2	116.0	275.8	4.1	63.2
21AR213_178	760.9	14977.854	3.076	18.516	1.8	0.35645	2.9	0.04892	2.1	0.717	307.9	6.3	309.6	7.8	323.2	46.5	307.9	6.3	95.3
21AR213_179	103.8	14717.748	0.824	16.342	5.5	0.52171	6.1	0.06318	1.6	0.263	394.9	6.1	426.3	21.1	600.5	126.7	394.9	6.1	65.8
21AR213_180	168.8	20428.384	2.013	17.233	3.7	0.48194	4.2	0.06131	1.4	0.324	383.6	5.1	399.4	14.0	493.0	88.4	383.6	5.1	77.8
21AR213_181	229.9	32509.334	3.439	17.510	2.8	0.59501	3.5	0.07657	2.0	0.559	475.6	9.0	474.1	13.3	467.6	64.4	475.6	9.0	101.7
21AR213_182	109.7	8400.532	1.477	16.729	6.2	0.38895	6.9	0.04882	2.2	0.321	307.3	6.6	333.6	19.5	522.3	142.4	307.3	6.6	58.8
21AR213_183	328.0	21284.009	2.409	18.919	3.5	0.29887	4.3	0.04176	1.9	0.444	263.7	4.9	265.5	9.9	282.4	87.1	263.7	4.9	93.4
21AR213_184	291.6	12976.811	1.038	16.871	3.0	0.67452	4.6	0.08454	1.9	0.410	523.2	9.4	523.5	18.7	525.6	91.5	523.2	9.4	99.5
21AR213_185	511.7	41082.974	8.736	13.382	1.8	1.67431	2.8	0.16407	2.0	0.732	979.4	18.5	998.8	17.7	1042.6	38.3	1042.6	38.3	93.9
21AR213_186	512.1	38584.227	1.096	18.879	2.9	0.27972	3.9	0.03877	2.2	0.573	245.2	5.3	250.4	8.6	300.7	72.2	245.2	5.3	81.5
21AR213_187	98.9	44707.259	1.686	10.729	1.6	3.10197	2.3	0.24368	1.6	0.710	1405.8	20.7	1433.2	17.8	1474.8	30.9	1474.8	30.9	95.3
21AR213_188	153.4	3186.841	1.737	12.492	13.0	0.14761	14.1	0.01421	2.6	0.188	91.0	2.4	139.8	18.4	1078.1	278.3	91.0	2.4	8.4
21AR213_189	172.6	24289.506	0.594	17.248	3.5	0.49167	4.3	0.06249	1.8	0.412	390.8	6.7	406.0	14.3	494.9	85.8	390.8	6.7	79.0
21AR213_190	414.1	13868.745	1.348	18.754	2.9	0.28088	3.9	0.03919	1.6	0.401	247.8	3.8	251.4	8.8	285.6	82.8	247.8	3.8	86.8
21AR213_191	129.1	2559.632	1.567	13.209	15.2	0.11749	17.1	0.01222	2.7	0.160	78.3	2.1	112.8	18.2	921.0	347.4	78.3	2.1	8.5
21AR213_192	131.4	5694.442	1.879	14.985	11.4	0.22199	12.4	0.02522	2.1	0.171	160.5	3.4	203.6	22.9	737.4	258.8	160.5	3.4	21.8
21AR213_193	385.4	23574.272	0.763	19.055	2.8	0.28366	3.5	0.03988	1.8	0.530	252.1	4.5	253.6	7.8	268.4	67.3	252.1	4.5	93.9
21AR213_194	293.7	14705.462	1.471	18.947	3.9	0.30408	5.1	0.04281	1.7	0.332	270.2	4.5	269.6	12.1	265.1	110.5	270.2	4.5	101.9
21AR213_195	50.5	3440.740	2.054	13.484	11.3	0.53310	13.6	0.05552	2.1	0.158	348.3	7.3	433.9	48.0	918.8	276.4	348.3	7.3	37.9
21AR213_196	203.4	21631.434	2.240	12.187	1.7	2.17594	2.4	0.19480	1.7	0.694	1147.3	17.4	1173.4	16.6	1222.6	33.7	1222.6	33.7	93.8
21AR213_197	75.2	12084.132	2.508	16.927	5.0	0.68724	6.2	0.08631	1.8	0.288	533.7	9.2	531.1	25.7	521.1	130.8	533.7	9.2	102.4
21AR213_198	282.7	96157.265	8.999	12.914	1.5	1.94744	2.3	0.18362	1.8	0.760	1086.7	17.6	1097.6	15.5	1120.0	30.0	1120.0	30.0	97.0
21AR213_199	159.2	19838.660	2.189	3.247	1.1	28.64480	1.6	0.67789	1.2	0.751	3336.3	31.3	3441.4	15.7	3503.9	16.3	3503.9	16.3	95.2
21AR213_200	502.9	31138.228	1.093	19.074	2.4	0.28620	2.9	0.04008	1.3	0.444	253.3	3.2	255.6	6.5	277.1	59.1	253.3	3.2	91.4
21AR213_201	749.0	29009.959	4.901	17.909	1.3	0.48523	2.1	0.06381	1.4	0.646	398.8	5.3	401.7	7.0	419.3	36.1	398.8	5.3	95.1
21AR213_202	82.1	5928.340	1.079	14.696	10.0	0.37160	11.3	0.04121	1.9	0.170	260.3	4.9	320.8	31.0	788.0	233.2	260.3	4.9	33.0
21AR213_203	469.2	2498.624	3.958	17.130	4.3	0.28546	21.9	0.03955	2.8	0.127	250.1	6.8	255.0	49.4	301.3	497.1	250.1	6.8	83.0
21AR213_204	252.7	84256.787	2.826	13.207	1.4	1.85744	1.9	0.17891	1.3	0.681	1061.0	12.5	1066.1	12.3	1077.3	27.4	1077.3	27.4	98.5
21AR213_205	328.8	25805.701	1.902	18.387	2.7	0.45402	3.2	0.06138	1.5	0.481	384.0	5.7	380.1	10.0	357.1	62.7	384.0	5.7	107.5
21AR213_206	32.7	1569.085	2.097	10.945	9.8	2.16297	21.6	0.19107	4.3	0.197	1127.2	44.1	1169.2	150.3	1248.8	415.7	1248.8	415.7	90.3
21AR213_207	953.4	69545.923	1.809	17.928	1.3	0.51945	1.9	0.06804	1.4	0.714	424.3	5.6	424.8	6.7	428.2	29.9	424.3	5.6	99.1
21AR213_208	301.1	38236.973	1.206	17.065	1.9	0.68342	2.6	0.08532	1.4	0.546	527.8	7.3	528.8	10.8	534.2	48.2	527.8	7.3	98.8
21AR213_209	104.3	16922.266	1.074	12.634	1.9	1.99093	2.6	0.18504	1.6	0.615	1094.5	15.8	1112.4	17.3	1148.6	40.1	1148.6	40.1	95.3
21AR213_210	493.0	51092.338	1.935	13.432	1.2	1.71368	1.8	0.16817	1.3	0.708	1002.0	11.7	1013.7	11.4	1039.8	25.4	1039.8	25.4	96.4
21AR213_211	411.6	100551.150	11.113	13.061	1.2	1.92239	1.8	0.18309	1.3	0.732	1083.9	12.9	1088.9	11.8	1099.8	24.0	1099.8	24.0	98.5
21AR213_212	77.3	5312.499	0.725	14.972	12.0	0.36244	14.3	0.04117	2.1	0.145	260.1	5.3	314.0	38.7	737.4	300.7	260.1	5.3	35.3
21AR213_213	689.5	123819.488	4.436	16.208	1.2	0.86623	1.7	0.10239	1.2	0.700	628.4	7.2	633.5	8.1	652.8	26.2	628.4	7.2	96.3
21AR213_214	68.9	22212.732	3.623	12.512	2.4	2.18616	2.9	0.20069	1.5	0.509	1179.0	15.7	1176.6	19.9	1173.1	48.7	1173.1	48.7	100.5
21AR213_215	276.5	36479.312	0.881	17.446	2.4	0.57913	3.0	0.07406	1.3	0.444	460.6	5.9	463.9	11.2	481.5	59.7	460.6	5.9	95.7
21AR213_216	128.5	21120.074	8.918	16.453	3.3	0.75046	3.8	0.09090	1.4	0.366	560.9	7.5	568.5	16.6	600.1	77.0	560.9	7.5	93.5
21AR213_217	661.3	22598.028	3.142	13.996	1.1	1.35808	1.9	0.13959	1.5	0.791	842.3	11.6	871.0	10.8	945.6	23.2	842.3	11.6	89.1
21AR213_218	441.9	40555.111	3.328	17.850	1.9	0.50090	2.5	0.06541	1.4	0.568	408.4	5.7	412.3	8.6	434.9	46.5	408.4	5.7	93.9
21AR213_219	283.1	14146.570	0.945	18.085	4.7	0.20279	5.1	0.02723	1.3	0.258	173.2	2.2	187.5	8.7	372.6	110.2	173.2	2.2	46.5
21AR213_220	204.8	15850.496	1.825	17.696	5.1	0.30502	5.6	0.03998	1.8	0.324	252.7	4.5	270.3	13.3	426.8	118.5	252.7	4.5	59.2
21AR213_221	234.9	5833.165	1.871	16.849	3.0	0.54776	7.0	0.07019	2.2	0.320	437.3	9.4	443.5	25.0	476.8	145.9	437.3	9.4	91.7
21AR213_222	148.9	10080.195	1.086	17.564	5.1	0.31749	6.6	0.04172	1.9	0.287	263.5	4.9	280.0	16.3	421.0	142.2	263.5	4.9	62.6
21AR213_223	122.8	9998.914	3.166	15.921	6.8	0.34042	7.5	0.04040	2.3	0.313	255.3	5.9	297.5	19.4	644.2	153.3	255.3	5.9	39.6
21AR213_224	503.5	4371.339	46.570	16.781	3.1	0.57099	3.9	0.07390	1.5	0.383	459.6	6.6	458.7	14.4	454.7	80.3	459.6	6.6	101.1
21AR213_225	220.6	24636.578	0.710	16.376	2.4	0.84425	3.4	0.10165	1.5	0.458	624.1	9.1	621.5	15.6	613.0	64.5	624.1	9.1	101.8
21AR213_226	97.1	2481.843	1.489	15.431	6.5	0.46503	14.6	0.05743	1.6	0.109	360.0	5.6	387.8	47.1	557.9	317.2	360.0	5.6	64.5

21AR213_227	211.7	15147.836	3.578	12.954	1.5	1.86916	2.6	0.17816	1.3	0.475	1056.9	12.2	1070.2	17.5	1098.3	46.5	1098.3	46.5	96.2
21AR213_228	245.8	15428.469	1.524	17.533	3.3	0.45963	3.9	0.05973	1.3	0.328	374.0	4.6	384.0	12.5	445.6	81.8	374.0	4.6	83.9
21AR213_229	189.0	5822.878	1.101	17.233	6.6	0.31426	9.0	0.04123	2.4	0.268	260.5	6.1	277.5	21.8	424.6	192.8	260.5	6.1	61.3
21AR213_230	33.4	5048.128	16.926	14.425	8.9	0.88405	11.3	0.09687	4.6	0.407	596.1	26.1	643.2	53.7	813.2	215.4	596.1	26.1	73.3
21AR213_231	102.3	12587.729	1.685	18.524	4.2	0.53061	5.3	0.07317	2.1	0.398	455.2	9.2	432.2	18.6	312.3	110.5	455.2	9.2	145.8
21AR213_232	180.3	19828.974	3.342	13.407	2.2	1.71187	3.4	0.16881	1.7	0.498	1005.6	15.7	1013.0	21.7	1029.9	59.5	1029.9	59.5	97.6
21AR213_233	137.4	11659.789	2.036	17.322	6.1	0.39157	6.5	0.05058	1.8	0.281	318.1	5.7	335.5	18.7	459.3	139.2	318.1	5.7	69.3
21AR213_234	114.4	2626.338	1.268	11.820	16.6	0.13518	17.9	0.01246	3.2	0.179	79.8	2.5	128.7	21.6	1165.9	349.1	79.8	2.5	6.8
21AR213_235	249.5	21678.737	1.420	19.144	3.4	0.32263	4.3	0.04567	1.5	0.357	287.9	4.3	283.9	10.7	252.7	92.8	287.9	4.3	113.9
21AR213_236	209.2	16374.741	1.057	18.589	4.5	0.31176	5.4	0.04284	1.6	0.293	270.4	4.2	275.5	13.0	320.6	117.3	270.4	4.2	84.4
21AR213_237	450.6	25704.933	2.767	15.605	1.5	0.92309	4.6	0.10605	4.1	0.901	649.8	25.6	664.0	22.4	713.5	42.4	649.8	25.6	91.1
21AR213_238	273.3	57718.480	5.171	15.971	1.4	0.93045	2.1	0.10889	1.5	0.719	666.3	9.6	667.9	10.3	674.2	31.3	666.3	9.6	98.8
21AR213_239	139.5	15680.628	0.693	17.679	4.6	0.49486	5.0	0.06492	1.3	0.267	405.5	5.3	408.2	16.9	424.8	108.1	405.5	5.3	95.4
21AR213_240	156.1	52045.949	4.056	11.772	1.7	2.31893	2.7	0.19983	2.1	0.768	1174.4	22.3	1218.1	19.2	1297.2	33.7	1297.2	33.7	90.5
21AR213_241	225.6	31884.746	5.203	15.940	2.6	0.69855	5.0	0.08181	4.1	0.822	506.9	20.2	537.9	21.0	672.7	61.4	506.9	20.2	75.4
21AR213_242	164.1	12411.622	1.215	17.895	4.8	0.29915	5.8	0.03989	1.4	0.244	252.1	3.5	265.7	13.5	388.5	125.7	252.1	3.5	64.9
21AR213_243	124.8	19291.083	1.168	16.703	3.5	0.78310	3.9	0.09661	1.4	0.363	594.5	8.0	587.3	17.4	560.2	79.1	594.5	8.0	106.1
21AR213_244	129.5	9077.087	15.686	16.981	4.3	0.62715	7.0	0.07985	1.8	0.262	495.2	8.7	494.3	27.3	491.1	148.5	495.2	8.7	100.8
21AR213_245	116.7	17576.395	1.968	17.861	4.1	0.46710	4.3	0.06177	1.4	0.326	386.4	5.3	389.2	13.9	407.0	91.2	386.4	5.3	94.9
21AR213_246	633.5	15714.232	1.763	18.114	1.8	0.40886	3.9	0.05498	2.1	0.530	345.0	7.0	348.1	11.6	369.4	75.0	345.0	7.0	93.4
21AR213_247	265.9	19304.986	1.162	18.706	3.7	0.30091	4.6	0.04169	1.4	0.294	263.3	3.5	267.1	10.9	301.9	101.0	263.3	3.5	87.2
21AR213_248	105.9	16156.124	0.497	17.100	4.9	0.50788	5.6	0.06440	1.6	0.278	402.3	6.1	417.0	19.3	500.3	119.5	402.3	6.1	80.4
21AR213_249	223.2	14534.985	2.463	18.559	3.7	0.32255	5.1	0.04452	1.4	0.271	280.8	3.8	283.9	12.5	310.3	110.8	280.8	3.8	90.5
21AR213_250	261.1	19355.726	1.961	18.725	3.8	0.27497	4.3	0.03811	1.4	0.317	241.1	3.2	246.7	9.5	300.8	93.5	241.1	3.2	80.2
21AR213_251	130.9	12429.803	1.368	17.778	5.1	0.42792	5.9	0.05670	1.4	0.241	355.5	4.9	361.7	18.1	402.6	129.2	355.5	4.9	88.3
21AR213_252	177.3	5889.786	1.713	14.925	3.0	0.76481	4.0	0.08645	2.1	0.524	534.5	10.7	576.8	17.4	747.9	71.3	534.5	10.7	71.5
21AR213_253	12.1	933.357	0.891	6.719	26.4	0.69532	33.1	0.03765	11.4	0.344	238.3	26.7	536.0	138.1	2150.9	546.5	238.3	26.7	11.1
21AR213_254	497.2	59755.956	11.862	17.551	1.7	0.56733	2.4	0.07300	1.5	0.627	454.2	6.5	456.3	8.8	467.9	41.1	454.2	6.5	97.1
21AR213_255	920.0	95156.898	5.326	17.978	1.4	0.50863	2.0	0.06676	1.3	0.652	416.6	5.2	417.5	6.8	423.6	33.5	416.6	5.2	98.4
21AR213_256	260.7	116353.930	8.970	9.418	1.2	4.39321	2.0	0.30252	1.6	0.782	1703.8	23.4	1711.1	16.5	1720.7	22.9	1720.7	22.9	99.0
21AR213_257	487.5	6737.095	1.227	18.500	2.4	0.27999	2.8	0.03942	1.3	0.453	249.2	3.1	250.7	6.2	265.1	56.9	249.2	3.1	94.0
21AR213_258	201.8	16877.769	6.512	18.038	3.4	0.43440	4.3	0.05813	1.5	0.339	364.3	5.2	366.3	13.3	380.1	91.2	364.3	5.2	95.8
21AR213_259	554.0	13302.659	4.147	18.861	2.1	0.29890	3.8	0.04203	2.0	0.530	265.4	5.3	265.5	8.9	267.8	74.4	265.4	5.3	99.1
21AR213_260	113.3	7809.533	2.163	15.753	8.2	0.32130	8.7	0.03806	1.8	0.209	240.8	4.3	282.9	21.4	648.1	181.9	240.8	4.3	37.2
21AR213_261	279.9	12087.809	1.573	17.790	2.5	0.40662	2.9	0.05397	1.3	0.454	338.8	4.4	346.4	8.5	398.7	58.1	338.8	4.4	85.0
21AR213_262	47.2	5758.411	2.995	13.540	5.4	1.32516	10.0	0.13553	5.8	0.580	819.3	44.5	856.8	57.7	955.8	166.0	819.3	44.5	85.7
21AR213_263	100.9	18699.003	1.980	16.251	3.9	0.80309	4.4	0.09653	1.8	0.409	594.1	10.2	598.6	19.9	616.6	86.5	594.1	10.2	96.3
21AR213_264	144.1	11390.560	1.522	17.435	4.2	0.45666	5.9	0.05954	1.9	0.321	372.8	6.8	381.9	18.8	438.4	124.3	372.8	6.8	85.0
21AR213_265	202.0	9585.553	1.858	16.896	3.3	0.57864	5.4	0.07336	1.7	0.315	456.4	7.5	463.6	20.1	500.4	112.7	456.4	7.5	91.2
21AR213_266	666.3	8100.327	26.314	15.244	2.8	0.88123	5.3	0.10072	3.5	0.653	618.6	20.4	641.6	25.1	724.4	84.9	618.6	20.4	85.4
21AR213_267	178.4	49279.215	3.049	13.757	1.7	1.54096	2.5	0.15515	1.6	0.656	929.7	14.0	946.9	15.1	987.8	37.7	987.8	37.7	94.1
21AR213_268	18.3	3699.547	1.738	12.097	9.4	1.87396	11.4	0.17349	2.4	0.211	1031.3	23.0	1071.9	75.6	1156.3	221.6	1156.3	221.6	89.2
21AR213_269	303.7	17283.520	1.101	18.422	2.9	0.31810	3.6	0.04345	1.7	0.464	274.2	4.5	280.4	8.8	333.8	72.2	274.2	4.5	82.1
21AR213_270	126.2	5055.452	0.814	14.285	10.5	0.21320	11.1	0.02317	1.8	0.161	147.7	2.6	196.2	19.7	830.1	227.6	147.7	2.6	17.8
21AR213_271	54.2	4971.499	1.919	13.908	10.0	0.52937	12.1	0.05602	2.1	0.174	351.4	7.2	431.4	42.6	885.5	246.7	351.4	7.2	39.7
21AR213_272	40.2	3632.550	1.648	13.008	13.8	0.56261	16.9	0.05636	2.2	0.130	353.5	7.5	453.2	61.8	997.9	340.8	353.5	7.5	35.4
21AR213_273	175.4	16858.794	1.567	17.118	3.6	0.64622	4.8	0.08205	1.4	0.305	508.4	7.1	506.2	18.9	497.1	99.7	508.4	7.1	102.3
21AR213_274	830.1	64138.286	39.451	18.387	1.5	0.43164	2.2	0.05825	1.6	0.730	365.0	5.8	364.3	6.8	361.3	34.4	365.0	5.8	101.0
21AR213_275	209.9	18790.703	3.178	15.474	2.6	0.84241	6.0	0.09641	4.9	0.814	593.3	27.5	620.5	27.7	721.6	73.5	593.3	27.5	82.2
21AR213_276	340.1	33606.386	1.688	18.163	2.5	0.44667	3.5	0.05980	2.0	0.566	374.4	7.2	374.9	10.9	379.2	64.5	374.4	7.2	98.7
21AR213_277	293.7	19151.441	2.309	17.569	2.7	0.56138	3.8	0.07286	1.8	0.484	453.4	8.1	452.4	13.9	448.4	74.2	453.4	8.1	101.1
21AR213_278	161.1	16703.623	2.254	17.255	3.6	0.54960	4.1	0.07028	1.7	0.419	437.8	7.2	444.7	14.6	481.5	81.4	437.8	7.2	90.9
21AR213_279	527.9	58241.213	2.554	13.336	1.5	1.69840	2.5	0.16588	2.0	0.785	989.4	18.0	1007.9	15.9	1049.4	31.2	1049.4	31.2	94.3
21AR213_280	265.8	79539.145	2.162	13.314	1.3	1.75657	2.2	0.17112	1.7	0.784	1018.3	16.0	1029.6	14.1	1054.6	27.2	1054.6	27.2	96.6
21AR213_281	287.3	6701.143	0.905	15.816	6.6	0.10946	8.5	0.01308	1.9	0.221	83.8	1.6	105.5	8.5	629.5	178.4	83.8	1.6	13.3
21AR213_282	261.8	19649.177	1.617	17.796	3.3	0.42867	4.1	0.05640	1.4	0.339	353.7	4.7	362.2	12.4	418.1	85.3	353.7	4.7	84.6
21AR213_283	476.5	54262.507	36.711	17.700	1.7	0.56052	2.4	0.07273	1.5	0.625	452.6	6.7	451.9	8.9	449.1	42.3	452.6	6.7	100.8
21AR213_284	140.3	51810.611	3.264	13.205	1.8	1.82777	2.3	0.17673	1.5	0.642	1049.1	14.6	1055.5	15.4	1069.6	36.1	1069.6	36.1	98.1
21AR213_285	218.2	35659.182	1.471	16.915	2.6	0.69386	3.5	0.08636	2.2	0.649	534.0	11.5	535.1	14.4	541.0	57.7	534.0	11.5	98.7
21AR213_286	486.6	20990.240	2.016	18.730	2.4	0.31506	3.5	0.04358	1.5	0.444	275.0	4.1	278.1	8.4	305.2	70.6	275.0	4.1	90.1
21AR213_287	161.9	3270.540	1.263	16.746	7.4	0.27239	12.8	0.03600	2.0	0.159	228.0	4.6	244.6	27.9	408.0	283.9	228.0	4.6	55.9
21AR213_288	575.8	42893.840	0.876	19.256	2.3	0.27534	3.1	0.03898	1.8	0.570	246.5	4.2	247.0	6.8	252.4	58.3	246.5	4.2	97.7
21AR213_289	85.5	9519.191	0.456	15.250	8.0	0.54971	8.9	0.06257	1.7	0.195	391.2	6.6	444.8	32.0	733.4	184.8	391.2	6.6	53.3
21AR213_290	517.4	59625.349	6.217	17.978	1.8	0.47911	2.5	0.06308	1.6	0.631	394.3	6.2	397.5	8.4	416.7	44.2	394.3	6.2	94.6
21AR213_291	261.5	11042.011	1.489	16.528	5.8	0.18937	6.4	0.02333	2.0	0.305	148.7	2.9	176.1	10.4	563.5	133.6	148.7	2.9	26.4
21AR213_292	108.3	101084.100	2.225	7.715	1.3	6.58000	2.1	0.37004	1.6	0.789	2029.6	28.5	2056.7	18.3	2084.7	22.4	2084.7	22.4	97.4
21AR213_293	228.2	2816.735	1.524	15.787	2.5	0.69221	3.6	0.08663	1.8	0.492	535.6	9.2	534.1	15.0	528.9	69.1	535.6	9.2	101.3
21AR213_294	303.1	47110.478	1.143	16.435	1.7	0.86557	4.1	0.10413	3.7	0.904	638.5	22.5	633.2	19.3	614.9	38.0	638.5	22.5	103.8
21AR213_295	105.3	6879.399	1.824	16.905	5.7	0.31493	6.9	0.04027	2.0	0.291	254.5	5.0	278.0	16.7	481.8	145.1	254.5	5.0	52.8
21AR213_296	131.0	32580.299	1.043	13.893	2.0	1.55394	2.7	0.15819	1.4	0.510	946.7	12.2	952.1	16.8	965.4	47.7	965.4	47.7	98.1
21AR213_297	197.5	12351.965	1.257	18.240	3.8	0.30918	4.6	0.04198	1.6	0.341	265.1	4.1	273.5	11.1	347.7	98.3	265.1	4.1	76.2
21AR213_298	489.4	64114.679	3.273	13.183	1.3	1.80869	2.1	0.17415	1.7	0.780	1034.9	15.9	1048.6	14.0	1078.0	26.8	1078.0	26.8	96.0
21AR213_299	308.2	6849.536	4.780	17.647	5.2	0.39533	6.8	0.05277	1.8	0.264	331.5	5.8	338.3	19.5	385.8	146.8	331.5	5.8	85.9
21AR213_300	351.6	23406.818	2.568	18.218	3.9	0.30659	4.5	0.04115	1.9	0.422	260.0	4.9	271.5	10.8	373.5	92.5	260.0	4.9	69.6
21AR213_301	274.9	15378.737	2.312	18.422	3.3	0.32653	3.9	0.04460	1.7	0.444	281.3	4.7	286.9	9.7	334.2	78.6	281.3	4.7	84.2
21AR213_302	822.9	73986.414	88.490	17.515	1.5	0.56404	2.2	0.07221	1.5	0.711	449.4	6.7	454.2	7.9	479.0	33.7	449.4	6.7	93.8
21AR213_303	360.4	55751.648	1.416	9.455	1.6	3.72211	3.0	0.25692	2.6	0.848	1474.0	33.9	1576.1	24.3	1716.4	29.6	1716.4	29.6	85.9
21AR213_304	243.4	2127.352	0.496	16.642	5.8	0.22506	9.1	0.03078	1.5	0.160	195.4	2.8	206.1	16.9	330.9	202.8	195.4	2.8	59.1
21AR213_305	238.8	6374.991	4.087	17.617	4.3	0.40674	6.9	0.05448	1.7	0.246	342.0	5.6	346.5	20.2	378.2	149.8	342.0	5.6	90.4
21AR213_306	336.8	40555.297	4.015	15.151	1.6	1.14037	2.2	0.12651	1.4	0.646	767.9	10.3	772.7	12.0	787.4	35.4	767.9	10.3	97.5
21AR213_307	344.7	29285.044	1.856	16.860	1.9	0.69422	2.7	0.08596	1.7	0.639	531.6	8.7	535.3	11.2	552.3	45.0	531.6	8.7	96.3
21AR213_308	326.0	16601.946	1.297	18.392	3.4	0.27636	4.0	0.03763	1.6	0.395	238.1	3.7	247.8	8.8	341.3	83.5	238.1	3.7	69.8
21AR213_309	283.3	22089.011	1.036	18.632	3.1	0.35751	3.8	0.04912	1.6	0.421	309.1	4.9	310.4	10.2	320.8	78.9	309.1	4.9	96.4
21AR213_310	438.1	56916.114	8.246	13.964	1.5	1.39976	2.9	0.14276	2.5	0.862	860.3	20.4	888.8	17.4	961.6	30.5	961.6	30.5	89.5
21AR213_311	154.4	7310.484	0.510	16.170	7.8	0.25652	8.5	0.03120	1.8	0.206	198.1	3.4	231.9	17.6	590.8	180.5	198.1	3.4	33.5
21AR213_312	433.7	12305.289	0.566	18.625	3.3	0.27203	5.1	0.03773	2.0	0.404	238.7	4.8	244.3	11.0	299.3	105.3	238.7	4.8	79.8
21AR213_313	1532.5	128523.449	19.616	6.497	1.1	7.68843	1.8	0.36397	1.4	0.797	2001.0	24.5	2195.3	16.1	2382.7	18.4	2382.7	18.4	84.0
21AR213_314	144.5	11031.767	1.409	17.005	4.4	0.46857	5.7	0.05938	1.6	0.281	371.9	5.8	390.2	18.6	501.4	121.2	371.9	5.8	74.2
21AR213_315	293.1	8117.127	0.912	17.748	3.2	0.42984	4.7	0.05750	1.9	0.406	360.4	6.7	363.1	14.3	381.2	96.2	360.4	6.7	94.5
21AR213_316	128.0	12998.336	1.025	17.299	4.2	0.56377	4.6	0.07248	1.5	0.333	451.1	6.6	454.0	16.7	469.7	95.3	451.1	6.6	96.0
21AR213_317	252.2	9760.637	0.914	17.873	3.0	0.43109	3.7	0.05769	1.5	0.416	361.6	5.4	364.0	11.2	380.1	74.9	361.6	5.4	95.1
21AR213_318	76.0	4574.287	1.323	15.271	10.4	0.28197	11.8	0.03298	2.9	0.249	209.2	6.0	252.2	26.3	675.1	244.4	209.2	6.0	31.0
21AR213_319	429.1	59657.972	3.293	17.081	1.7	0.67007	2.3	0.08383	1.5	0.650	519.0	7.4	520.8	9.3	529.7	38.0	519.0	7.4	98.0
21AR213_320	133.5	3630.028	1.150	13.495	13.1	0.16020	13.8	0.01666	2.5	0.184	106.5	2.7	150.9	19.3	922.2	278.7	106.5	2.7	11.5
21AR213_321	249.1	9660.242	1.923	18.164	4.1	0.30528	6.5	0.04159	1.4	0.219	262.7	3.7	270.5	15.5	339.9	144.6	262.7	3.7	77.3
21AR213_322	583.7	88167.900	13.438	14.661	1.6	1.20475	3.4	0.12913	3.0	0.872	782.9	21.9	802.8	18.9	859.2	34.7	782.9	21.9	91.1
21AR213_323	1003.6	5002.829	1.739	18.691	2.1	0.21314	3.0	0.03076	1.9	0.640	195.3	3.7	196.2	5.4	208.0	53.7	195.3	3.7	93.9
21AR213_324	215.8	74319.860	3.886	13.616	1.7	1.67385	2.3	0.16673	1.5	0.661	994.1	14.3	998.6	14.9	1009.6	35.6	1009.6	35.6	98.5
21AR213_325	388.4	3972.128	0.770	18.243	3.0	0.26349	5.6	0.03763	1.2	0.223	238.1	2.9	237.5	11.9	231.9	126.2	238.1	2.9	102.7
21AR213_326	441.6	42651.594	1.458	18.329	2.2	0.43559	3.4	0.05866	2.4	0.699	367.5	8.5	367.1	10.5	366.0	55.1	367.5	8.5	100.4
21AR213_327	161.8	10463.929	1.104	16.262	6.8	0.23081	7.8	0.02804	2.0	0.254	178.2	3.5	210.9	14.8	593.9	162.8	178.2	3.5	30.0
21AR213_328	330.8	8876.912	1.162	17.421	6.9	0.12661	8.3	0.01657	1.6	0.189	105.9	1.7	121.0	9.5	429.8	182.0	105.9	1.7	24.6
21AR213_329	244.0	35003.636	1.245	13.577	1.6	1.69720	2.8	0.16914	2.2	0.787	1007.4	20.4	1007.5	17.7	1008.6	34.7	1008.6	34.7	99.9
21AR213_330	284.7	10734.703	2.099	17.633	4.3	0.19287	5.2	0.02544	1.7	0.329	161.9	2.7	179.1	8.5	413.0	108.8	161.9	2.7	39.2
21AR213_331	331.5	9303.471	1.549	17.175	6.7	0.13570	7.2	0.01748	1.8	0.247	111.7	2.0	129.2	8.7	464.9	154.8	111.7	2.0	24.0
21AR213_332	277.1	68458.439	2.418	14.290	1.5	1.43191	2.1	0.14993	1.5	0.683	900.6	12.2	902.3	12.7	907.5	32.0	907.5	32.0	99.2
21AR213_333	494.0	9892.304	3.491	17.904	2.4	0.43178	3.4	0.05793	2.2	0.656	363.1	7.9	364.4	10.4	374.3	57.9	363.1	7.9	97.0
21AR213_334	129.9	4456.060	0.669	15.543	4.7	0.70034	6.6	0.08365	1.4	0.216	517.9	7.1	539.0	27.5	630.2	138.1	517.9	7.1	82.2
21AR213_335	201.3	24449.998	0.795	17.251	2.5	0.65719	3.4	0.08359	1.7	0.513	517.5	8.6	512.9	13.5	493.3	63.4	517.5	8.6	104.9
21AR213_336	834.5	4420.245	0.908	18.200	2.2	0.27042	3.9	0.03822	1.8	0.476	241.8	4.4	243.0	8.4	255.9	78.5	241.8	4.4	94.5
21AR213_337	269.1	10856.943	0.701	18.338	3.2	0.31888	5.2	0.04375	1.7	0.325	276.1	4.6	281.0	12.8	323.6	112.2	276.1	4.6	85.3
21AR213_338	313.5	47883.000	4.668	16.259	2.2	0.77380	3.1	0.09217	2.2	0.701	568.4	11.9	581.9	13.8	636.3	47.7	568.4	11.9	89.3
21AR213_339	324.6	17499.538	2.158	19.101	2.9	0.29745	4.4	0.04209	1.5	0.336	265.8	3.9	264.4	10.3	253.1	96.1	265.8	3.9	105.0
21AR213_340	368.9	36138.311	9.153	18.288	2.2	0.46352	2.9	0.06226	1.4	0.501	389.3	5.4	386.7	9.2	371.9	55.9	389.3	5.4	104.7
21AR213_341	370.7	25872.763	4.851	13.245	1.9	1.75616	3.0	0.17095	1.9	0.622	1017.4	17.8	1029.4	19.7	1056.1	48.0	1056.1	48.0	96.3
21AR213_342	221.2	9224.689	1.872	16.703	3.2	0.64374	4.3	0.08050	1.9	0.453	499.1	9.4	504.6	17.1	530.7	83.9	499.1	9.4	94.1

21AR213_343	179.4	3854.933	0.751	16.954	7.5	0.32007	11.4	0.04227	2.3	0.202	266.9	6.0	282.0	28.0	409.6	249.5	266.9	6.0	65.2
21AR213_344	152.3	17977.379	1.786	13.049	2.5	1.86527	3.6	0.17905	2.2	0.608	1061.8	21.3	1068.9	23.6	1084.3	56.8	1084.3	56.8	97.9
21AR213_345	150.4	25741.629	2.228	12.283	2.6	2.25698	3.5	0.20327	2.2	0.628	1192.9	24.2	1199.0	24.9	1210.8	54.2	1210.8	54.2	98.5
21AR213_346	38.4	3175.743	63.724	12.845	11.0	0.84392	12.2	0.08378	2.1	0.174	518.6	10.6	621.3	56.5	1016.4	242.7	518.6	10.6	51.0
21AR213_347	108.8	1745.990	1.038	13.555	11.7	0.20597	15.0	0.02290	2.7	0.181	145.9	3.9	190.2	25.9	783.1	309.4	145.9	3.9	18.6
21AR213_348	371.6	11043.314	1.049	8.643	1.3	4.58899	1.9	0.29199	1.4	0.722	1651.5	19.9	1747.3	15.8	1864.7	23.6	1864.7	23.6	88.6
21AR213_349	980.8	45161.293	2.195	18.083	1.5	0.45682	2.1	0.06054	1.5	0.686	378.9	5.4	382.0	6.8	402.0	34.9	378.9	5.4	94.3
21AR213_350	195.2	8212.743	2.898	17.141	5.5	0.21805	6.9	0.02809	1.6	0.227	178.6	2.7	200.3	12.5	465.4	148.5	178.6	2.7	38.4
21AR213_351	344.7	23148.629	2.593	18.603	2.9	0.32543	3.8	0.04468	1.8	0.480	281.8	5.1	286.1	9.6	322.5	76.5	281.8	5.1	87.4
21AR213_352	213.6	411.092	1.466	3.702	54.2	0.04108	58.6	0.00123	16.1	0.275	7.9	1.3	40.9	23.5	3133.8	917.0	7.9	1.3	0.3
21AR213_353	135.6	856.517	0.701	13.218	7.3	0.32153	33.3	0.03991	1.7	0.050	252.3	4.1	283.1	82.4	546.7	732.1	252.3	4.1	46.2
21AR213_354	119.2	45367.841	2.437	13.093	1.8	1.91308	2.4	0.18325	1.5	0.645	1084.7	15.3	1085.7	15.9	1088.4	36.5	1088.4	36.5	99.7
21AR213_355	213.1	27367.611	1.465	17.525	2.8	0.59629	3.4	0.07687	1.5	0.445	477.4	6.9	474.9	12.7	463.7	66.6	477.4	6.9	103.0
21AR213_356	194.0	56918.165	4.410	13.167	1.7	1.90762	2.2	0.18372	1.4	0.633	1087.3	14.2	1083.8	14.9	1077.6	34.8	1077.6	34.8	100.9
21AR213_357	116.9	3064.569	1.750	14.951	6.5	0.81769	16.0	0.09588	2.1	0.131	590.2	11.8	606.8	73.1	670.0	339.9	590.2	11.8	88.1
21AR213_358	116.0	12120.692	2.829	14.112	2.4	1.29325	3.9	0.13531	2.4	0.609	818.1	18.4	842.7	22.5	909.2	64.1	818.1	18.4	90.0
21AR213_359	55.7	4304.586	1.705	10.254	21.8	0.54425	22.3	0.04208	3.4	0.154	265.7	8.9	441.2	79.7	1505.2	416.7	265.7	8.9	17.7
21AR213_360	729.0	47665.712	1.508	18.495	1.8	0.38884	2.4	0.05273	1.4	0.581	331.2	4.6	333.5	7.0	350.5	45.0	331.2	4.6	94.5
21AR213_361	135.8	10179.330	1.409	17.612	4.8	0.31710	5.6	0.04177	1.9	0.329	263.8	4.8	279.7	13.8	415.4	119.0	263.8	4.8	63.5
21AR213_362	346.8	60400.397	4.946	13.248	1.5	1.76920	2.8	0.17150	2.4	0.844	1020.4	22.3	1034.2	18.1	1064.6	30.2	1064.6	30.2	95.8
21AR213_363	35.4	7847.884	1.825	14.270	6.2	1.21429	7.7	0.12967	2.4	0.304	786.0	17.4	807.2	43.1	867.0	153.1	786.0	17.4	90.7
21AR213_364	557.2	7281.925	1.404	16.908	1.8	0.56038	3.0	0.07149	2.3	0.758	445.1	9.7	451.8	10.9	486.8	42.9	445.1	9.7	91.4
21AR213_365	188.3	9995.193	1.899	17.843	4.4	0.28337	5.8	0.03785	1.6	0.276	239.5	3.8	253.3	13.0	384.5	125.0	239.5	3.8	62.3
21AR213_366	91.1	6390.589	0.491	15.899	6.9	0.33706	8.4	0.04053	1.7	0.198	256.1	4.2	294.9	21.4	615.8	176.9	256.1	4.2	41.6
21AR213_367	25.2	2018.759	2.336	11.763	17.0	0.52651	20.8	0.04921	4.3	0.207	309.7	13.0	429.5	72.8	1137.6	405.6	309.7	13.0	27.2
21AR213_368	325.0	40194.578	7.808	16.585	1.7	0.75617	3.3	0.09196	2.7	0.825	567.1	14.6	571.8	14.3	591.5	39.9	567.1	14.6	95.9
21AR213_369	181.7	73853.176	2.763	11.390	1.4	2.81368	2.0	0.23409	1.4	0.714	1355.9	17.1	1359.2	14.6	1365.2	26.4	1365.2	26.4	99.3
21AR213_370	43.1	2182.161	0.467	10.324	19.0	0.36113	21.7	0.02910	3.7	0.170	184.9	6.7	313.1	58.4	1426.7	408.6	184.9	6.7	13.0
21AR213_371	507.4	10695.233	5.454	18.681	2.5	0.31391	3.2	0.04386	1.6	0.494	276.7	4.3	277.2	7.8	282.1	63.8	276.7	4.3	98.1
21AR213_372	178.5	21624.446	0.719	17.270	3.1	0.65058	3.7	0.08286	1.4	0.389	513.1	7.1	508.8	14.8	490.5	75.1	513.1	7.1	104.6
21AR213_373	234.6	47658.063	4.326	14.062	1.6	1.23304	3.8	0.12692	3.4	0.888	770.2	24.5	815.7	21.3	942.8	35.8	770.2	24.5	81.7
21AR213_374	631.7	31378.913	1.778	16.525	1.8	0.79715	2.7	0.09676	1.7	0.620	595.4	9.6	595.2	12.3	595.6	46.4	595.4	9.6	100.0
21AR213_375	518.6	1766.560	1.940	9.390	24.7	0.02505	26.7	0.00185	4.2	0.157	11.9	0.5	25.1	6.6	1590.8	494.2	11.9	0.5	0.7
21AR213_376	282.4	807.030	1.144	6.833	40.7	0.03533	48.7	0.00198	19.2	0.394	12.8	2.4	35.3	16.9	2090.0	797.8	12.8	2.4	0.6
21AR213_377	420.0	96343.777	8.206	11.391	1.2	2.75231	2.9	0.22883	2.6	0.910	1328.4	31.3	1342.7	21.3	1366.5	22.8	1366.5	22.8	97.2
21AR213_378	260.8	9516.647	2.392	15.656	1.7	0.95431	2.5	0.11152	1.6	0.640	681.5	10.4	680.3	12.4	677.3	41.2	681.5	10.4	100.6
21AR213_379	435.7	30522.528	2.093	19.397	2.7	0.26563	3.3	0.03790	1.6	0.497	239.8	3.8	239.2	7.0	234.5	65.6	239.8	3.8	102.3
21AR213_380	357.6	39074.022	2.792	18.020	2.2	0.44722	3.0	0.05911	1.8	0.591	370.2	6.3	375.3	9.3	408.0	53.6	370.2	6.3	90.7
21AR213_381	112.4	17011.746	1.185	16.589	3.6	0.71164	4.3	0.08718	1.5	0.360	538.8	8.0	545.7	18.0	575.5	86.6	538.8	8.0	93.6
21AR213_382	109.6	6820.041	1.267	14.486	8.3	0.23799	9.1	0.02592	2.2	0.242	165.0	3.6	216.8	17.8	826.0	184.5	165.0	3.6	20.0
21AR213_383	184.8	569.534	1.728	5.915	50.0	0.02927	59.9	0.00146	18.7	0.312	9.4	1.8	29.3	17.3	2298.0	1000.3	9.4	1.8	0.4
21AR213_384	719.6	33794.480	6.400	15.122	1.1	1.18342	2.2	0.13113	1.8	0.828	794.3	13.4	792.9	11.9	789.8	25.5	794.3	13.4	100.6
21AR213_385	274.0	80914.695	2.938	12.385	1.5	2.15152	3.6	0.19443	3.3	0.907	1145.3	34.4	1165.5	25.1	1204.2	30.0	1204.2	30.0	95.1
21AR213_386	158.6	12299.148	0.847	18.503	3.2	0.45028	4.8	0.06203	1.4	0.291	387.9	5.2	377.5	15.1	314.8	103.9	387.9	5.2	123.2
21AR213_387	231.6	11670.902	1.461	18.718	4.2	0.31005	5.6	0.04327	1.5	0.265	273.1	4.0	274.2	13.5	284.8	124.1	273.1	4.0	95.9
21AR213_388	1078.3	4746.713	1.217	18.847	2.2	0.13250	5.0	0.01931	1.4	0.283	123.3	1.7	126.3	5.9	184.9	111.7	123.3	1.7	66.7
21AR213_389	196.9	12987.835	0.623	18.800	4.2	0.31449	4.9	0.04398	1.5	0.301	277.4	4.0	277.7	11.9	280.6	106.5	277.4	4.0	98.9
21AR213_390	240.5	12637.546	1.140	16.800	2.4	0.66788	4.0	0.08339	1.8	0.452	516.3	9.1	519.4	16.4	534.0	78.8	516.3	9.1	96.7
21AR213_391	332.8	48290.971	1.920	17.315	1.9	0.66421	2.5	0.08423	1.5	0.602	521.3	7.5	517.2	10.1	499.9	43.6	521.3	7.5	104.3
21AR213_392	200.9	32087.314	2.176	16.899	2.9	0.73065	3.4	0.09065	1.4	0.415	559.4	7.6	556.9	14.6	547.8	67.6	559.4	7.6	102.1
21AR213_393	53.3	733.651	1.168	10.798	13.3	0.52100	16.6	0.05187	2.2	0.131	326.0	6.9	425.8	57.7	1010.4	333.8	326.0	6.9	32.3
21AR213_394	77.0	1526.168	1.562	12.816	4.3	1.27165	25.4	0.13498	1.6	0.062	816.2	12.1	833.1	144.5	879.5	526.2	816.2	12.1	92.8
21AR213_395	512.4	43037.561	3.430	13.323	1.4	1.70529	2.1	0.16623	1.6	0.731	991.3	14.3	1010.5	13.7	1053.3	29.4	1053.3	29.4	94.1
21AR213_396	55.6	20812.580	2.601	13.223	4.5	1.87487	4.9	0.18210	1.7	0.356	1078.4	17.2	1072.3	32.2	1060.6	91.4	1060.6	91.4	101.7
21AR213_397	446.9	5731.092	5.567	15.415	1.9	0.92078	4.1	0.10749	2.1	0.512	658.2	13.1	662.8	19.9	679.4	74.9	658.2	13.1	96.9
21AR213_398	191.7	17902.134	3.653	17.613	3.8	0.44378	4.4	0.05776	2.0	0.447	362.0	6.9	372.9	13.7	442.1	87.7	362.0	6.9	81.9
21AR213_399	105.0	15353.516	0.878	13.755	2.7	1.58677	3.8	0.16124	1.9	0.510	963.7	17.1	965.0	23.4	969.1	66.0	969.1	66.0	99.4
21AR213_400	218.4	11859.379	0.886	17.305	3.7	0.50167	4.7	0.06467	1.8	0.390	404.0	7.1	412.8	15.8	463.6	95.0	404.0	7.1	87.1
21AR213_401	111.1	5164.180	0.821	15.291	11.2	0.20184	12.1	0.02351	2.3	0.193	149.8	3.5	186.7	20.7	684.1	253.9	149.8	3.5	21.9
21AR213_402	218.3	1608.515	1.327	15.821	6.0	0.21065	20.1	0.02842	1.6	0.079	180.7	2.8	194.1	35.5	361.8	452.3	180.7	2.8	49.9
21AR213_403	211.2	14919.951	2.360	18.471	4.3	0.30120	5.0	0.04140	1.4	0.277	261.5	3.6	267.3	11.8	319.9	109.9	261.5	3.6	81.7
21AR213_404	528.6	19222.357	2.007	17.525	2.4	0.57320	3.5	0.07438	1.4	0.402	462.5	6.3	460.1	12.9	449.1	71.2	462.5	6.3	103.0
21AR213_405	308.5	12233.979	2.542	18.626	3.0	0.33262	4.4	0.04630	1.7	0.382	291.7	4.8	291.6	11.1	291.2	92.7	291.7	4.8	100.2
21AR213_406	14.3	878.907	0.485	6.245	27.0	0.73904	30.0	0.03713	8.7	0.288	235.0	20.0	561.8	129.8	2280.7	498.1	235.0	20.0	10.3
21AR213_407	115.9	10492.020	1.332	16.540	5.8	0.49306	7.1	0.06098	1.7	0.231	381.6	6.1	407.0	24.0	554.8	151.8	381.6	6.1	68.8
21AR213_408	402.0	11403.371	5.755	13.254	1.5	1.75746	3.2	0.17292	2.3	0.733	1028.2	22.2	1029.9	20.6	1034.5	43.8	1034.5	43.8	99.4
21AR213_409	147.0	19171.207	1.551	17.027	3.5	0.66706	4.2	0.08401	1.7	0.408	520.0	8.5	518.9	16.9	515.1	83.4	520.0	8.5	100.9
21AR213_410	172.8	116212.516	1.949	5.459	1.2	12.34091	2.5	0.49120	2.1	0.862	2575.9	45.0	2630.7	23.1	2673.9	20.6	2673.9	20.6	96.3
21AR213_411	293.6	32061.266	16.653	14.233	1.7	1.29988	3.0	0.13594	2.4	0.803	821.7	18.7	845.7	17.4	910.1	37.2	821.7	18.7	90.3
21AR213_412	919.9	25618.116	1.414	19.769	2.6	0.12435	3.2	0.01815	1.5	0.460	116.0	1.7	119.0	3.6	181.0	66.2	116.0	1.7	64.1
21AR213_413	761.5	21182.118	1.332	18.887	1.8	0.30800	2.7	0.04298	1.5	0.548	271.3	3.9	272.6	6.3	285.1	50.7	271.3	3.9	95.2
21AR213_414	286.8	78896.758	6.249	10.824	1.2	3.22906	1.9	0.25532	1.4	0.751	1465.9	18.3	1464.2	14.4	1462.5	23.4	1462.5	23.4	100.2
21AR213_415	438.3	12128.576	1.277	17.639	3.9	0.13060	5.1	0.01716	1.4	0.281	109.7	1.6	124.6	6.0	421.2	109.8	109.7	1.6	26.0
21AR213_416	137.1	30430.669	3.473	13.318	1.8	1.80192	2.5	0.17606	1.3	0.523	1045.4	12.4	1046.2	16.1	1048.6	42.4	1048.6	42.4	99.7
21AR213_417	257.0	8236.005	1.737	18.094	2.8	0.45516	6.3	0.06204	1.5	0.230	388.0	5.5	380.9	20.1	338.8	139.2	388.0	5.5	114.5
21AR213_418	320.7	4463.283	1.596	16.767	2.5	0.58091	5.3	0.07512	1.5	0.282	466.9	6.8	465.0	19.9	456.7	113.8	466.9	6.8	102.2
21AR213_419	131.6	10605.339	1.431	17.395	5.1	0.44833	5.6	0.05834	1.5	0.263	365.5	5.3	376.1	17.7	442.6	121.0	365.5	5.3	82.6
21AR213_420	167.0	12187.319	1.318	17.344	3.9	0.53067	4.6	0.06849	1.5	0.317	427.1	6.1	432.3	16.3	461.0	97.7	427.1	6.1	92.6
21AR213_421	162.3	8892.029	1.577	17.912	4.1	0.34209	6.4	0.04609	1.6	0.245	290.5	4.4	298.8	16.5	364.8	139.3	290.5	4.4	79.6
21AR213_422	330.5	15736.521	3.619	12.397	1.2	2.10702	1.9	0.19285	1.4	0.757	1136.8	14.7	1151.1	12.9	1179.1	24.2	1179.1	24.2	96.4
21AR213_423	821.2	36039.009	4.864	18.881	1.8	0.31991	2.4	0.04444	1.5	0.606	280.3	4.0	281.8	6.0	295.5	44.0	280.3	4.0	94.9
21AR213_424	188.1	9621.128	5.727	14.075	1.9	1.27193	3.3	0.13350	2.2	0.657	807.8	16.7	833.3	19.0	902.6	51.9	807.8	16.7	89.5
21AR213_425	157.6	1788.956	1.565	15.835	8.4	0.34235	21.3	0.04542	1.5	0.070	286.4	4.2	299.0	55.3	399.4	478.2	286.4	4.2	71.7
21AR213_426	431.4	4418.354	2.275	17.469	2.2	0.41076	4.1	0.05557	1.7	0.401	348.6	5.6	349.4	12.3	355.8	85.8	348.6	5.6	98.0
21AR213_427	397.9	5584.820	1.296	18.663	3.2	0.25793	9.0	0.03694	1.7	0.192	233.8	4.0	233.0	18.7	225.8	204.2	233.8	4.0	103.5
21AR213_428	696.1	29157.504	2.017	18.920	1.9	0.30070	2.6	0.04191	1.6	0.603	264.7	4.1	266.9	6.1	288.0	47.4	264.7	4.1	91.9
21AR213_429	205.2	23817.870	2.037	16.874	2.9	0.69595	3.3	0.08655	1.4	0.415	535.1	7.1	536.4	13.8	542.8	65.8	535.1	7.1	98.6
21AR213_430	301.6	15895.789	1.002	13.636	2.1	1.58459	3.4	0.15942	1.6	0.467	953.5	14.1	964.2	21.2	989.4	61.3	989.4	61.3	96.4
21AR213_431	39.6	7237.079	3.957	11.954	3.4	2.36139	5.1	0.21077	1.5	0.292	1232.9	16.6	1231.0	36.0	1228.5	94.8	1228.5	94.8	100.4
21AR213_432	247.6	9911.977	1.977	10.107	2.1	3.09800	8.7	0.23162	8.2	0.949	1343.0	99.7	1432.2	66.5	1568.1	51.4	1568.1	51.4	85.6
21AR213_433	395.0	22241.870	1.586	14.653	1.3	1.22410	2.4	0.13190	1.7	0.713	798.7	13.1	811.6	13.7	848.2	35.7	798.7	13.1	94.2
21AR213_434	584.5	108460.740	6.892	16.246	1.4	0.87388	2.5	0.10363	2.1	0.836	635.7	12.5	637.7	11.7	645.7	29.3	635.7	12.5	98.4
21AR213_435	676.7	22575.521	1.572	17.251	1.4	0.60395	2.0	0.07674	1.5	0.735	476.7	6.9	479.7	7.8	495.4	30.4	476.7	6.9	96.2
21AR213_436	126.6	21721.763	2.138	14.115	2.7	1.30344	4.6	0.13527	3.7	0.793	817.8	28.1	847.2	26.5	925.9	57.8	817.8	28.1	88.3
21AR213_437	341.7	21409.993	0.676	18.588	2.7	0.32707	3.4	0.04492	1.5	0.449	283.2	4.3	287.3	8.6	321.7	69.6	283.2	4.3	88.0
21AR213_438	160.9	9031.100	2.036	18.148	4.3	0.30247	6.4	0.04125	1.7	0.261	260.6	4.3	268.3	15.2	337.6	140.8	260.6	4.3	77.2
21AR213_439	185.2	8416.307	0.697	16.918	5.8	0.21975	7.1	0.02793	1.7	0.235	177.6	2.9	201.7	13.0	494.6	151.8	177.6	2.9	35.9
21AR213_440	307.9	34005.715	1.668	17.571	2.5	0.57104	3.0	0.07368	1.5	0.505	458.3	6.7	458.7	11.1	461.8	57.8	458.3	6.7	99.2
21AR213_441	132.4	12428.547	2.674	17.504	4.5	0.46025	5.6	0.05999	1.6	0.278	375.6	5.7	384.4	18.1	439.1	120.8	375.6	5.7	85.5
21AR213_442	755.8	6284.252	0.923	18.350	2.0	0.29396	2.6	0.04110	1.5	0.576	259.7	3.9	261.7	6.1	280.7	49.3	259.7	3.9	92.5
21AR213_443	77.9	2634.400	1.049	10.541	21.8	0.19542	23.9	0.01591	4.9	0.203	101.8	4.9	181.2	39.7	1406.2	450.2	101.8	4.9	7.2
21AR213_444	350.1	24448.736	1.817	18.657	2.5	0.31536	3.2	0.04337	1.5	0.471	273.7	4.0	278.3	7.8	318.3	64.0	273.7	4.0	86.0
21AR213_445	168.5	7058.992	1.001	11.076	2.1	2.79131	4.2	0.23034	1.6	0.374	1336.3	19.0	1353.2	31.5	1380.9	75.2	1380.9	75.2	96.8
21AR213_446	607.2	37257.638	2.421	18.030	1.5	0.48134	2.5	0.06369	1.7	0.671	398.0	6.6	399.0	8.4	405.7	42.3	398.0	6.6	98.1
21AR213_447	741.6	75850.534	5.236	16.622	1.5	0.76893	2.3	0.09343	1.6	0.695	575.8	8.7	579.1	10.0	593.4	35.3	575.8	8.7	97.0
21AR213_448	570.5	128900.749	3.518	14.480	1.3	1.16720	3.4	0.12330	3.1	0.925	749.5	22.2	785.3	18.5	889.3	26.6	749.5	22.2	84.3
21AR213_449	397.7	41195.338	1.806	17.665	2.0	0.56410	2.5	0.07304	1.5	0.589	454.5	6.5	454.2	9.2	453.8	45.1	454.5	6.5	100.1
21AR213_450	174.2	12183.719	6.304	17.660	4.6	0.31227	5.5	0.04105	1.6	0.284	259.4	4.0	275.9	13.4	420.0	118.8	259.4	4.0	61.8
21AR213_451	932.0	34529.680	2.099	17.423	1.4	0.51157	2.5	0.06543	1.8	0.713	408.6	7.0	419.5	8.5	481.1	38.3	408.6	7.0	84.9
21AR213_452	469.8	12921.384	1.426	18.357	3.0	0.34612	4.3	0.04728	2.2	0.514	297.8	6.5	301.8	11.3	333.8	84.4	297.8	6.5	89.2
21AR213_453	147.0	54783.605	4.481	12.455	2.5	2.05070	3.4	0.18674	2.3	0.683	1103.7	23.5	1132.5	23.1	1189.1	48.9	1189.1	48.9	92.8
21AR213_454	568.6	20929.274	2.242	18.919	3.2	0.27416	4.4	0.03832	2.4	0.536	242.4	5.6	246.0	9.6	281.4	85.2	242.4	5.6	86.2
21AR213_455	169.7	51746.849	2.036	12.302	1.4	2.24689	2.2	0.20223	1.7	0.756	1187.3	18.1	1195.8	15.5	1212.1	28.3	1212.1	28.3	98.0
21AR213_456	106.3	20254.156	1.746	16.812	4.4	0.71594	4.8	0.08887	1.6	0.340	548.9	8.6	548.3	20.4	546.7	98.8	548.9	8.6	100.4
21AR213_457	193.2	21915.991	1.262	17.525	3.0	0.57388	3.5	0.07423	1.6	0.447	461.6	7.1	460.5	13.1	456.2	70.2	461.6	7.1	101.2
21AR213_458	614.1	3710.869	1.045	17.917	2.7	0.24551	3.5	0.03449	1.6	0.467	218.6	3.5	222.9	7.1	269.7	71.5	218.6	3.5	81.1

21AR213_459	636.7	17034.040	2.460	18.823	2.3	0.31775	3.0	0.04435	1.5	0.499	279.7	4.0	280.2	7.3	284.9	58.8	279.7	4.0	98.2
21AR213_460	211.1	51808.094	1.889	13.242	1.6	1.74268	2.3	0.16875	1.5	0.643	1005.2	13.7	1024.5	14.8	1066.6	35.3	1066.6	35.3	94.2
21AR213_461	351.8	6415.476	1.234	18.197	3.8	0.29917	9.4	0.04137	1.5	0.159	261.3	3.8	265.8	22.0	306.0	211.5	261.3	3.8	85.4
21AR213_462	180.5	26535.063	2.468	17.227	2.8	0.63282	3.2	0.08013	1.2	0.392	496.9	5.9	497.8	12.5	503.2	64.1	496.9	5.9	98.7
21AR213_463	77.2	4149.843	1.483	14.708	8.7	0.42451	11.9	0.04791	2.9	0.241	301.7	8.5	359.3	36.0	751.2	244.3	301.7	8.5	40.2
21AR213_464	172.9	27082.752	8.789	15.452	3.3	0.95917	4.1	0.10873	2.4	0.591	665.4	15.5	682.9	20.6	741.8	70.6	665.4	15.5	89.7
21AR213_465	69.5	18064.169	1.769	12.199	2.6	2.28169	3.1	0.20461	1.5	0.477	1200.0	16.4	1206.6	22.2	1219.3	54.4	1219.3	54.4	98.4
21AR213_466	98.2	5667.522	2.050	16.786	4.8	0.64525	7.8	0.08256	1.5	0.191	511.4	7.4	505.6	31.2	480.2	170.1	511.4	7.4	106.5
21AR213_467	67.3	35461.596	1.844	11.293	2.0	2.89311	2.6	0.23895	1.3	0.513	1381.3	16.3	1380.1	19.3	1379.2	42.3	1379.2	42.3	100.2
21AR213_468	390.7	35072.193	1.713	19.293	2.5	0.30624	3.3	0.04337	1.8	0.555	273.7	4.9	271.3	7.9	251.6	63.7	273.7	4.9	108.8
21AR213_469	389.8	31795.486	1.376	19.019	3.1	0.30056	3.8	0.04199	1.8	0.479	265.1	4.7	266.8	8.9	282.8	76.0	265.1	4.7	93.8
21AR213_470	270.2	4313.529	1.278	16.746	6.6	0.13998	11.0	0.01810	2.0	0.179	115.6	2.2	133.0	13.7	457.0	239.5	115.6	2.2	25.3
21AR213_471	370.8	82466.842	2.010	12.847	1.6	1.96932	2.4	0.18473	1.8	0.746	1092.8	17.7	1105.1	15.9	1130.2	31.4	1130.2	31.4	96.7
21AR213_472	76.6	10145.598	1.358	17.543	4.6	0.58119	5.1	0.07620	1.9	0.361	473.4	8.5	465.2	19.2	426.0	106.9	473.4	8.5	111.1
21AR213_473	389.7	44177.153	4.942	18.588	2.3	0.42803	3.0	0.05833	1.5	0.516	365.4	5.5	361.8	9.1	339.4	57.8	365.4	5.5	107.7
21AR213_474	488.1	35255.391	1.657	18.931	2.9	0.29086	3.7	0.04043	1.7	0.475	255.5	4.4	259.2	8.4	294.0	73.7	255.5	4.4	86.9
21AR213_475	44.6	5168.817	1.035	14.647	8.3	0.66804	10.8	0.07432	2.4	0.220	462.1	10.6	519.5	43.9	781.5	221.3	462.1	10.6	59.1
21AR213_476	435.7	4771.393	2.882	17.690	4.8	0.21088	9.3	0.02876	1.5	0.166	182.8	2.8	194.3	16.5	337.6	208.5	182.8	2.8	54.1
21AR213_477	281.9	22664.142	1.464	17.644	2.7	0.50527	3.7	0.06578	1.6	0.431	410.7	6.4	415.3	12.6	441.9	74.4	410.7	6.4	92.9
21AR213_478	191.6	12066.610	2.056	17.178	3.8	0.47740	5.7	0.06105	2.3	0.395	382.0	8.4	396.3	18.8	481.5	116.3	382.0	8.4	79.3
21AR213_479	409.0	50176.066	4.865	17.162	1.8	0.66580	2.7	0.08371	1.9	0.732	518.2	9.7	518.2	10.8	518.7	39.7	518.2	9.7	99.9
21AR213_480	37.8	3871.129	2.054	12.884	11.5	0.56361	11.9	0.05557	2.2	0.184	348.6	7.5	453.9	43.7	1030.3	237.5	348.6	7.5	33.8
21AR213_481	211.9	96250.071	2.095	8.576	1.5	5.35294	2.6	0.33504	2.1	0.816	1862.8	34.0	1877.4	22.0	1894.3	26.8	1894.3	26.8	98.3
21AR213_482	126.4	3904.235	1.586	13.367	12.2	0.14678	15.1	0.01503	3.6	0.241	96.2	3.5	139.1	19.6	953.0	299.6	96.2	3.5	10.1
21AR213_483	130.0	41850.900	1.802	13.047	1.8	1.85292	2.4	0.17694	1.4	0.590	1050.2	13.6	1064.5	15.6	1094.7	38.3	1094.7	38.3	95.9
21AR213_484	497.0	26790.272	1.859	19.184	2.4	0.26607	3.0	0.03759	1.5	0.517	237.9	3.6	239.5	6.4	257.2	58.7	237.9	3.6	92.5
21AR213_485	147.5	47000.177	2.865	13.357	1.7	1.71396	2.4	0.16756	1.5	0.635	998.7	14.0	1013.8	15.3	1047.4	37.2	1047.4	37.2	95.3
21AR213_486	300.2	12756.887	1.500	18.535	2.9	0.32850	4.1	0.04536	2.3	0.578	286.0	6.6	288.4	10.2	309.3	75.4	286.0	6.6	92.5
21AR213_487	459.6	149750.474	1.637	12.309	1.3	2.23550	2.2	0.20088	1.8	0.794	1180.0	19.0	1192.2	15.6	1215.3	26.5	1215.3	26.5	97.1
21AR213_488	211.5	2203.080	2.381	16.210	4.7	0.46124	6.5	0.06105	1.7	0.258	382.0	6.2	385.1	20.7	405.0	139.8	382.0	6.2	94.3
21AR213_489	74.7	1481.705	2.259	8.210	27.2	0.15633	29.6	0.01011	5.6	0.191	64.9	3.6	147.5	40.6	1834.5	528.9	64.9	3.6	3.5
21AR213_490	396.2	64346.920	2.993	13.992	1.2	1.45704	1.9	0.14906	1.5	0.757	895.7	12.2	912.8	11.6	955.2	25.8	955.2	25.8	93.8
21AR213_491	335.4	7640.984	1.210	18.178	2.5	0.28391	5.1	0.03896	1.6	0.325	246.4	4.0	253.8	11.4	323.8	108.6	246.4	4.0	76.1
21AR213_492	116.9	8704.906	0.846	14.904	10.0	0.36388	10.4	0.04050	2.0	0.194	256.0	5.1	315.1	28.3	780.3	215.6	256.0	5.1	32.8
21AR213_493	162.7	9004.008	1.490	17.718	4.6	0.31612	5.8	0.04201	1.8	0.308	265.3	4.6	278.9	14.1	396.0	123.5	265.3	4.6	67.0
21AR213_494	154.1	16753.073	0.591	17.221	4.2	0.48342	4.7	0.06165	1.4	0.298	385.6	5.2	400.4	15.5	487.6	98.4	385.6	5.2	79.1
21AR213_495	76.0	14025.737	1.599	12.803	3.2	1.96583	4.2	0.18574	1.5	0.348	1098.2	14.7	1103.9	28.1	1115.9	78.0	1115.9	78.0	98.4
21AR213_496	147.2	17806.238	1.778	16.883	4.3	0.61890	5.9	0.07718	3.0	0.518	479.3	14.0	489.2	22.7	536.7	109.7	479.3	14.0	89.3
21AR213_497	419.0	26784.373	0.896	18.771	3.0	0.27437	3.4	0.03790	1.4	0.404	239.8	3.2	246.2	7.5	308.5	71.2	239.8	3.2	77.7
21AR213_498	1303.0	1704.287	1.168	10.313	19.8	0.01138	25.8	0.00093	4.5	0.176	6.0	0.3	11.5	3.0	1391.7	489.5	6.0	0.3	0.4
21AR213_499	628.3	13583.438	7.537	16.369	2.0	0.55240	3.7	0.06701	3.2	0.848	418.1	12.8	446.6	13.5	596.8	42.9	418.1	12.8	70.1
21AR213_500	86.0	4396.338	0.821	13.159	15.5	0.28955	16.4	0.02899	2.7	0.164	184.2	4.9	258.2	37.4	999.3	329.5	184.2	4.9	18.4
21AR213_501	142.1	8882.215	0.824	17.523	4.7	0.31717	5.5	0.04174	1.6	0.284	263.6	4.0	279.7	13.4	417.7	117.5	263.6	4.0	63.1
21AR213_502	264.8	843.288	1.523	4.286	51.0	0.03943	52.5	0.00131	10.4	0.198	8.4	0.9	39.3	20.2	2971.2	845.4	8.4	0.9	0.3
21AR213_503	305.9	26207.965	1.311	18.836	3.5	0.28801	3.9	0.03995	1.6	0.402	252.5	3.9	257.0	8.9	299.1	82.0	252.5	3.9	84.4
21AR213_504	438.9	8953.090	5.811	17.740	2.0	0.46511	2.9	0.06191	1.7	0.602	387.2	6.5	387.8	9.2	392.3	51.4	387.2	6.5	98.7
21AR213_505	80.0	5260.151	1.547	14.455	11.0	0.38064	11.5	0.04174	2.4	0.205	263.6	6.1	327.5	32.1	811.8	234.8	263.6	6.1	32.5
21AR213_506	168.0	6903.356	1.577	17.018	2.9	0.63726	5.4	0.08192	1.8	0.324	507.5	8.6	500.6	21.4	470.0	113.4	507.5	8.6	108.0
21AR213_507	70.5	8462.003	5.795	15.672	7.6	0.59580	8.1	0.06990	1.9	0.239	435.6	8.1	474.6	30.5	668.7	167.5	435.6	8.1	65.1
21AR213_508	265.4	5508.666	2.254	15.588	8.2	0.10571	9.7	0.01252	1.9	0.200	80.2	1.5	102.0	9.4	648.5	204.4	80.2	1.5	12.4
21AR213_509	407.1	37033.519	4.186	15.349	1.7	1.05683	3.8	0.11890	3.3	0.880	724.2	22.8	732.3	19.8	757.8	38.0	724.2	22.8	95.6
21AR213_510	166.2	32134.667	2.660	13.588	2.0	1.63341	3.7	0.16269	3.0	0.815	971.7	26.9	983.2	23.0	1009.7	42.9	1009.7	42.9	96.2
21AR213_511	231.2	24445.467	5.523	18.168	2.4	0.46228	3.5	0.06188	1.7	0.484	387.0	6.4	385.8	11.3	379.6	69.0	387.0	6.4	102.0
21AR213_512	273.1	12591.347	1.901	18.761	3.7	0.26468	5.7	0.03702	1.9	0.341	234.3	4.5	238.4	12.1	280.3	122.2	234.3	4.5	83.6
21AR213_513	121.4	9904.042	1.651	17.227	4.8	0.47097	6.4	0.06070	1.7	0.271	379.9	6.4	391.9	20.8	464.3	136.4	379.9	6.4	81.8
21AR213_514	237.1	12897.278	0.752	18.022	4.2	0.29228	5.1	0.03919	2.4	0.480	247.8	5.9	260.3	11.6	375.7	99.7	247.8	5.9	66.0
21AR213_515	131.3	13309.037	0.651	18.416	4.0	0.44911	4.8	0.06153	2.4	0.508	384.9	9.1	376.7	15.1	327.2	93.9	384.9	9.1	117.6
21AR213_516	563.8	652.966	1.291	5.530	54.1	0.01597	57.1	0.00072	15.2	0.266	4.6	0.7	16.1	9.1	2463.4	949.5	4.6	0.7	0.2
21AR213_517	139.5	65524.367	0.538	9.224	1.4	4.45309	2.1	0.29995	1.5	0.739	1691.1	23.0	1722.3	17.3	1761.2	25.8	1761.2	25.8	96.0
21AR213_518	644.1	80326.261	2.207	16.896	1.5	0.69424	2.3	0.08585	1.7	0.739	530.9	8.6	535.3	9.5	555.1	33.5	530.9	8.6	95.6
21AR213_519	267.9	8450.610	1.766	16.949	2.5	0.57186	4.5	0.07288	1.8	0.396	453.5	7.8	459.2	16.7	489.0	91.6	453.5	7.8	92.7
21AR213_520	42.4	2328.166	0.542	11.791	15.2	0.42251	18.5	0.03917	3.0	0.163	247.7	7.3	357.8	55.8	1153.4	362.6	247.7	7.3	21.5
**21AR217**																			
21AR217_1	282.4	13245.142	6.785	14.518	5.4	1.12074	8.5	0.12045	6.4	0.758	733.1	44.6	763.3	45.6	853.7	115.0	733.1	44.6	85.9
21AR217_2	745.0	3067.220	2.562	16.006	4.0	0.62857	11.6	0.07920	3.3	0.287	491.3	15.8	495.2	45.5	514.1	244.5	491.3	15.8	95.6
21AR217_3	1288.6	16259.666	1.598	17.100	1.9	0.64762	2.8	0.08182	1.7	0.624	507.0	8.5	507.0	11.1	508.3	47.9	507.0	8.5	99.7
21AR217_4	1043.1	9605.256	15.013	17.814	2.8	0.49431	3.9	0.06584	2.3	0.598	411.1	9.3	407.8	13.1	390.6	69.9	411.1	9.3	105.2
21AR217_5	704.4	112871.749	1.368	4.784	1.2	13.17609	1.8	0.45881	1.3	0.736	2434.3	27.1	2692.3	17.1	2892.9	19.9	2892.9	19.9	84.1
21AR217_6	123.2	502.386	2.028	7.256	51.1	0.14678	66.7	0.00964	15.8	0.237	61.8	9.7	139.1	86.8	1807.3	1212.7	61.8	9.7	3.4
21AR217_7	111.8	7625.141	1.554	13.375	7.0	1.79854	7.8	0.17930	1.7	0.220	1063.2	16.8	1044.9	50.8	1007.9	154.2	1007.9	154.2	105.5
21AR217_8	178.2	2478.602	1.093	12.850	9.9	0.45576	17.9	0.04585	2.0	0.114	289.0	5.7	381.3	56.9	989.3	362.1	289.0	5.7	29.2
21AR217_9	45.6	878.749	0.520	5.358	32.2	0.94336	35.0	0.03944	9.2	0.263	249.3	22.5	674.6	173.0	2592.3	568.0	249.3	22.5	9.6
21AR217_10	1021.2	19733.015	1.371	18.428	3.2	0.31556	4.2	0.04274	1.7	0.411	269.8	4.6	278.5	10.3	353.1	87.2	269.8	4.6	76.4
21AR217_11	106.1	1800.252	2.411	11.478	17.1	0.59339	26.2	0.05427	3.5	0.135	340.7	11.7	473.0	99.1	1180.5	514.9	340.7	11.7	28.9
21AR217_12	654.2	52302.333	6.218	13.592	1.6	1.67390	2.2	0.16520	1.3	0.579	985.6	11.7	998.7	14.0	1028.3	36.4	1028.3	36.4	95.8
21AR217_13	629.2	11005.174	1.384	18.177	4.5	0.32961	5.3	0.04451	2.0	0.368	280.7	5.4	289.3	13.4	359.9	112.0	280.7	5.4	78.0
21AR217_14	376.2	4889.399	1.019	14.505	14.6	0.24213	16.6	0.02660	2.1	0.126	169.2	3.5	220.2	32.8	808.1	344.9	169.2	3.5	20.9
21AR217_15	458.6	15384.336	2.856	16.725	3.6	0.73778	4.1	0.09088	1.9	0.451	560.8	10.0	561.1	17.8	563.5	80.1	560.8	10.0	99.5
21AR217_16	162.0	11081.179	0.402	13.080	3.5	1.58666	5.0	0.15301	1.7	0.337	917.8	14.4	965.0	31.1	1074.9	94.3	1074.9	94.3	85.4
21AR217_17	145.4	4421.410	0.621	14.034	9.3	0.58815	11.5	0.06267	2.5	0.219	391.9	9.6	469.7	43.3	871.4	233.0	391.9	9.6	45.0
21AR217_18	510.4	13051.337	6.932	16.707	4.7	0.56736	5.4	0.07000	2.2	0.409	436.1	9.2	456.3	19.7	560.2	106.8	436.1	9.2	77.9
21AR217_19	34.0	109.426	0.619	4.491	66.7	0.36497	83.0	0.02731	15.1	0.181	173.7	25.8	315.9	226.2	1566.5	1600.6	173.7	25.8	11.1
21AR217_20	434.7	8558.620	1.065	16.053	7.9	0.31827	9.0	0.03804	2.2	0.243	240.7	5.2	280.6	22.1	628.7	188.9	240.7	5.2	38.3
21AR217_21	192.2	4093.260	0.956	14.213	10.6	0.57738	12.1	0.06247	2.4	0.198	390.6	9.1	462.8	45.1	839.7	247.7	390.6	9.1	46.5
21AR217_22	296.9	27791.784	0.667	12.274	2.4	2.32981	3.4	0.20813	2.4	0.696	1218.9	26.5	1221.4	24.4	1226.8	48.4	1226.8	48.4	99.4
21AR217_23	313.0	15812.375	1.420	13.401	2.6	1.67028	3.6	0.16386	1.7	0.467	978.2	15.4	997.3	23.1	1040.3	65.0	1040.3	65.0	94.0
21AR217_24	606.9	13061.131	1.133	17.734	4.3	0.36918	5.1	0.04838	1.8	0.361	304.6	5.5	319.0	13.9	427.0	105.6	304.6	5.5	71.3
21AR217_25	626.6	22956.023	9.240	13.603	1.3	1.64227	3.2	0.16348	2.6	0.806	976.1	23.4	986.6	20.2	1010.9	38.4	1010.9	38.4	96.6
21AR217_26	237.9	6914.950	2.812	15.544	7.4	0.68733	8.2	0.08012	1.9	0.236	496.9	9.2	531.2	33.7	682.5	169.3	496.9	9.2	72.8
21AR217_27	230.9	20071.195	0.809	12.561	3.2	2.05475	3.8	0.18895	1.6	0.418	1115.7	16.3	1133.9	26.0	1169.7	68.4	1169.7	68.4	95.4
21AR217_28	1195.7	16450.397	1.635	18.258	3.7	0.32151	4.1	0.04330	1.6	0.379	273.2	4.2	283.1	10.2	366.0	86.1	273.2	4.2	74.7
21AR217_29	959.5	3052.255	0.869	17.119	7.8	0.29429	16.3	0.03944	1.9	0.117	249.3	4.7	261.9	37.7	377.1	365.0	249.3	4.7	66.1
21AR217_30	569.5	653.991	0.654	14.057	7.3	0.20360	12.9	0.03038	3.8	0.293	192.9	7.2	188.2	22.1	129.9		192.9	7.2	148.5
21AR217_31	58.5	3706.905	1.117	10.875	8.1	2.52510	11.0	0.20740	2.2	0.202	1215.0	24.7	1279.3	80.3	1389.9	207.4	1389.9	207.4	87.4
21AR217_32	229.0	832.985	1.687	9.212	24.0	0.12371	30.4	0.00977	7.2	0.236	62.7	4.5	118.4	34.0	1464.3	564.0	62.7	4.5	4.3
21AR217_33	547.4	27544.447	3.113	14.579	2.3	1.22332	2.8	0.13035	1.5	0.545	789.9	11.5	811.3	15.8	871.5	49.2	789.9	11.5	90.6
21AR217_34	1307.3	35954.868	1.581	13.708	1.3	1.59565	2.1	0.15952	1.6	0.759	954.1	14.0	968.5	13.0	1002.1	27.5	1002.1	27.5	95.2
21AR217_35	41.7	2644.542	2.392	11.357	10.5	2.19163	11.8	0.19178	3.2	0.270	1131.0	33.0	1178.4	82.4	1267.3	222.3	1267.3	222.3	89.2
21AR217_36	866.1	1563.708	1.052	16.273	4.4	0.33641	5.6	0.04670	1.7	0.299	294.2	4.8	294.5	14.2	297.2	121.2	294.2	4.8	99.0
21AR217_37	249.3	3647.054	0.582	15.878	10.1	0.32332	12.5	0.03938	2.2	0.175	249.0	5.4	284.5	31.1	588.1	267.6	249.0	5.4	42.3
21AR217_38	1159.9	2968.478	1.379	11.594	12.1	0.06257	16.3	0.00555	2.9	0.178	35.7	1.0	61.6	9.7	1240.3	314.7	35.7	1.0	2.9
21AR217_39	259.0	10493.071	4.499	15.579	6.4	0.70596	6.9	0.08126	1.8	0.255	503.6	8.6	542.3	29.2	709.4	142.8	503.6	8.6	71.0
21AR217_40	321.5	2055.498	0.700	11.332	23.7	0.15084	24.8	0.01336	5.5	0.223	85.6	4.7	142.7	33.1	1243.3	476.1	85.6	4.7	6.9
21AR217_41	343.6	847.865	1.695	10.734	19.0	0.17115	24.2	0.01620	3.4	0.141	103.6	3.5	160.4	35.9	1111.7	479.5	103.6	3.5	9.3
21AR217_42	114.7	993.785	31.996	12.532	11.3	0.86579	40.7	0.09586	2.0	0.049	590.1	11.2	633.3	192.6	791.5	862.3	590.1	11.2	74.6
21AR217_43	643.2	2222.904	2.543	16.129	3.7	0.46070	4.8	0.06021	2.0	0.420	376.9	7.4	384.7	15.5	433.0	97.7	376.9	7.4	87.0
21AR217_44	81.0	314.891	1.721	4.749	22.8	0.30433	32.4	0.01290	17.3	0.534	82.6	14.2	269.8	76.8	2569.0	460.8	82.6	14.2	3.2
21AR217_45	1075.9	1642.273	6.781	15.070	3.6	0.51174	6.1	0.06451	4.5	0.728	403.0	17.5	419.6	21.1	513.1	92.4	403.0	17.5	78.5
21AR217_46	765.9	15799.554	1.226	17.154	2.6	0.59683	3.5	0.07541	2.0	0.586	468.6	9.3	475.2	13.3	508.1	62.4	468.6	9.3	92.2
21AR217_47	375.5	2836.093	1.869	14.474	11.8	0.16908	17.5	0.01899	3.0	0.169	121.2	3.5	158.6	25.7	762.0	364.0	121.2	3.5	15.9
21AR217_48	180.2	9781.502	1.725	14.681	6.0	1.32587	7.2	0.14388	2.0	0.273	866.6	15.9	857.1	41.7	833.6	144.5	866.6	15.9	104.0
21AR217_49	834.3	4172.094	1.735	14.980	11.7	0.11174	14.7	0.01277	2.4	0.162	81.8	1.9	107.6	15.0	725.2	307.5	81.8	1.9	11.3
21AR217_50	589.6	16004.354	2.660	16.886	3.2	0.60355	4.5	0.07484	1.9	0.416	465.3	8.3	479.5	17.0	549.0	88.5	465.3	8.3	84.8
21AR217_51	568.7	12393.799	2.172	17.478	4.8	0.37886	6.3	0.04889	2.0	0.314	307.7	6.0	326.2	17.7	461.4	133.3	307.7	6.0	66.7
21AR217_52	410.6	17596.265	3.306	15.286	3.6	1.01273	5.3	0.11352	3.7	0.686	693.1	24.0	710.3	27.2	765.6	81.8	693.1	24.0	90.5

21AR217_53	242.2	7509.747	2.089	16.479	6.1	0.72262	9.4	0.08923	3.7	0.395	551.0	19.7	552.2	40.1	558.3	188.7	551.0	19.7	98.7
21AR217_54	584.1	4298.777	2.366	8.911	1.3	3.87180	2.0	0.25757	1.4	0.696	1477.4	18.4	1607.8	16.2	1783.9	26.2	1783.9	26.2	82.8
21AR217_55	789.1	47945.664	6.747	13.563	2.1	1.70450	3.2	0.16839	2.3	0.721	1003.2	21.5	1010.2	20.5	1026.2	45.0	1026.2	45.0	97.8
21AR217_56	102.7	1799.479	0.692	9.436	18.6	0.54556	22.5	0.03988	4.1	0.181	252.1	10.1	442.1	80.7	1610.3	413.7	252.1	10.1	15.7
21AR217_57	222.3	1332.362	1.361	8.094	27.0	0.20333	30.3	0.01301	5.5	0.181	83.3	4.5	187.9	52.0	1854.7	541.6	83.3	4.5	4.5
21AR217_58	518.0	8146.853	3.087	16.254	4.0	0.76822	6.1	0.09328	1.8	0.292	574.9	9.8	578.7	26.9	594.7	126.2	574.9	9.8	96.7
21AR217_59	217.7	5472.510	1.304	15.647	9.5	0.52311	11.7	0.06206	2.0	0.169	388.1	7.5	427.2	40.9	645.0	248.6	388.1	7.5	60.2
21AR217_60	1005.5	11740.467	1.651	18.438	3.8	0.28944	4.3	0.03963	1.7	0.391	250.5	4.1	258.1	9.8	328.7	90.2	250.5	4.1	76.2
21AR217_61	301.1	5642.950	2.922	14.943	8.0	0.38683	9.3	0.04360	2.0	0.216	275.1	5.4	332.1	26.4	753.9	192.4	275.1	5.4	36.5
21AR217_62	438.2	31855.569	5.601	13.429	2.0	1.73869	2.7	0.17041	1.6	0.599	1014.4	15.0	1023.0	17.2	1042.3	43.2	1042.3	43.2	97.3
21AR217_63	330.5	6026.733	1.906	17.426	6.9	0.33168	8.4	0.04378	2.2	0.263	276.2	6.0	290.8	21.3	411.0	181.9	276.2	6.0	67.2
21AR217_64	123.0	5712.051	2.187	14.587	6.3	1.08110	10.6	0.11855	4.2	0.391	722.2	28.4	744.2	56.1	811.8	204.8	722.2	28.4	89.0
21AR217_65	700.0	6947.641	18.282	17.838	4.4	0.43099	5.5	0.05784	1.9	0.339	362.5	6.6	363.9	16.9	374.0	117.4	362.5	6.6	96.9
21AR217_66	871.0	12924.582	1.784	17.953	3.3	0.47167	4.3	0.06258	2.4	0.556	391.3	9.0	392.3	13.8	399.5	79.2	391.3	9.0	98.0
21AR217_67	591.3	39778.203	2.921	13.449	1.9	1.75997	2.7	0.17090	1.8	0.688	1017.1	17.2	1030.8	17.2	1061.1	38.8	1061.1	38.8	95.8
21AR217_68	282.2	18044.360	2.253	13.047	3.3	1.83475	4.6	0.17512	3.0	0.655	1040.3	29.0	1058.0	30.2	1095.6	69.5	1095.6	69.5	94.9
21AR217_69	745.1	12291.820	0.823	18.083	4.0	0.30900	5.0	0.04134	2.0	0.391	261.1	5.0	273.4	12.0	380.8	103.6	261.1	5.0	68.6
21AR217_70	802.3	13716.010	6.434	16.994	3.6	0.57580	6.1	0.07216	4.8	0.794	449.1	21.0	461.8	22.6	526.0	81.3	449.1	21.0	85.4
21AR217_71	428.3	4393.526	2.489	16.598	5.5	0.46610	9.0	0.05928	1.8	0.195	371.3	6.3	388.5	29.1	493.4	195.0	371.3	6.3	75.2
21AR217_72	496.2	20701.096	2.029	13.564	1.9	1.65005	2.8	0.16358	1.7	0.606	976.7	15.2	989.6	17.5	1019.2	44.5	1019.2	44.5	95.8
21AR217_73	402.7	10707.276	1.944	17.464	4.3	0.56891	5.1	0.07370	1.5	0.298	458.4	6.8	457.3	19.0	452.7	109.1	458.4	6.8	101.3
21AR217_74	805.2	13172.203	4.841	16.589	2.9	0.67805	4.6	0.08297	2.1	0.452	513.8	10.2	525.6	18.8	577.9	88.8	513.8	10.2	88.9
21AR217_75	143.2	2460.375	0.741	13.528	12.1	0.44479	15.3	0.04731	2.7	0.176	298.0	7.8	373.6	47.9	875.3	312.6	298.0	7.8	34.0
21AR217_76	827.5	40294.413	1.755	9.012	1.7	3.97337	7.1	0.25943	6.9	0.969	1486.9	91.4	1628.8	57.6	1818.0	31.6	1818.0	31.6	81.8
21AR217_77	1400.7	1712.118	1.339	16.265	2.9	0.35431	6.1	0.04834	4.6	0.748	304.3	13.7	308.0	16.3	336.4	92.5	304.3	13.7	90.5
21AR217_78	340.6	2217.025	1.132	11.256	18.7	0.14967	20.0	0.01312	4.0	0.203	84.1	3.4	141.6	26.4	1263.3	383.0	84.1	3.4	6.7
21AR217_79	535.4	33707.724	7.840	13.871	2.4	1.49719	3.9	0.15109	3.0	0.771	907.1	25.5	929.2	23.8	983.2	50.8	983.2	50.8	92.3
21AR217_80	153.7	2325.634	1.147	13.062	12.3	0.46136	14.1	0.04739	3.0	0.215	298.5	8.8	385.2	45.1	946.9	281.6	298.5	8.8	31.5
21AR217_81	544.3	2203.016	1.438	12.865	15.6	0.09910	17.4	0.01004	4.4	0.255	64.4	2.8	95.9	15.9	974.7	343.6	64.4	2.8	6.6
21AR217_82	339.8	24743.236	1.323	13.119	2.2	1.82324	2.8	0.17435	1.8	0.634	1036.0	17.3	1053.9	18.6	1091.8	44.0	1091.8	44.0	94.9
21AR217_83	488.3	18254.473	0.410	13.372	2.2	1.64677	3.0	0.16133	1.8	0.588	964.1	15.7	988.3	18.9	1043.2	48.8	1043.2	48.8	92.4
21AR217_84	59.0	2300.239	1.627	10.628	15.2	1.12262	19.5	0.09247	3.9	0.201	570.1	21.3	764.2	104.5	1384.5	366.7	1384.5	366.7	41.2
21AR217_85	682.4	6997.089	1.986	16.831	3.9	0.55365	6.0	0.07000	2.9	0.488	436.2	12.4	447.4	21.9	506.4	116.1	436.2	12.4	86.1
21AR217_86	1909.7	35652.354	1.736	18.414	2.1	0.35538	2.6	0.04780	1.5	0.572	301.0	4.4	308.8	6.9	368.6	48.2	301.0	4.4	81.7
21AR217_87	327.3	2865.379	1.481	12.141	16.9	0.20968	19.6	0.01968	4.2	0.216	125.7	5.3	193.3	34.6	1128.8	382.7	125.7	5.3	11.1
21AR217_88	1433.7	567.250	0.614	7.442	27.5	0.01623	42.2	0.00107	10.3	0.245	6.9	0.7	16.3	6.8	1803.1	751.8	6.9	0.7	0.4
21AR217_89	322.0	28097.869	1.945	13.464	2.9	1.63646	3.4	0.16122	1.8	0.520	963.6	15.8	984.3	21.5	1031.9	58.8	1031.9	58.8	93.4
21AR217_90	215.6	17649.054	1.026	13.315	2.9	1.78667	3.7	0.17357	2.0	0.541	1031.7	19.0	1040.6	24.0	1060.2	62.3	1060.2	62.3	97.3
21AR217_91	963.3	12083.240	0.633	19.038	3.9	0.27662	6.4	0.03918	1.9	0.305	247.7	4.7	248.0	14.0	251.3	139.4	247.7	4.7	98.6
21AR217_92	427.1	30606.883	0.949	7.353	1.3	7.26138	2.5	0.38925	2.1	0.829	2119.4	37.5	2144.1	22.4	2168.6	24.4	2168.6	24.4	97.7
21AR217_93	332.9	4922.237	1.509	15.630	8.3	0.33573	9.6	0.04002	2.1	0.222	252.9	5.3	293.9	24.6	634.9	202.1	252.9	5.3	39.8
21AR217_94	466.0	29163.838	4.362	14.361	2.2	1.30780	3.2	0.13769	2.0	0.637	831.6	15.8	849.2	18.3	896.2	50.5	831.6	15.8	92.8
21AR217_95	550.1	10076.718	1.454	16.584	3.7	0.58799	4.4	0.07272	1.9	0.429	452.5	8.3	469.6	16.6	554.8	86.8	452.5	8.3	81.6
21AR217_96	350.4	3457.396	1.153	15.665	8.4	0.29446	14.6	0.03594	2.2	0.153	227.6	5.0	262.1	33.7	583.4	313.1	227.6	5.0	39.0
21AR217_97	324.9	21303.039	2.116	14.430	3.6	1.36353	4.4	0.14463	1.8	0.400	870.8	14.3	873.4	25.8	880.8	83.5	870.8	14.3	98.9
21AR217_98	758.2	11387.662	0.764	17.617	4.1	0.29733	6.3	0.03904	2.2	0.350	246.9	5.3	264.3	14.6	423.0	131.2	246.9	5.3	58.4
21AR217_99	337.2	6765.947	1.293	12.824	2.9	1.56325	3.6	0.15009	1.7	0.478	901.5	14.4	955.8	22.1	1083.7	62.9	1083.7	62.9	83.2
21AR217_100	680.1	6988.338	22.316	17.403	3.1	0.56311	6.5	0.07409	1.6	0.248	460.7	7.2	453.5	23.9	418.3	141.6	460.7	7.2	110.2
21AR217_101	509.7	1907.306	1.346	11.472	15.1	0.11021	16.2	0.00998	4.0	0.250	64.0	2.6	106.2	16.3	1201.0	308.8	64.0	2.6	5.3
21AR217_102	371.5	11897.736	1.504	16.621	6.1	0.58699	7.5	0.07255	1.8	0.235	451.5	7.7	468.9	28.3	556.1	159.6	451.5	7.7	81.2
21AR217_103	478.0	8191.117	0.909	17.875	4.9	0.41662	6.9	0.05604	2.6	0.382	351.5	9.0	353.6	20.5	368.5	142.8	351.5	9.0	95.4
21AR217_104	372.3	30808.889	2.635	12.949	3.0	1.85103	4.1	0.17603	2.8	0.669	1045.2	26.7	1063.8	27.2	1102.9	61.3	1102.9	61.3	94.8
21AR217_105	212.2	2564.413	0.659	13.139	12.7	0.33329	16.8	0.03450	2.6	0.154	218.6	5.6	292.1	42.8	931.2	342.2	218.6	5.6	23.5
21AR217_106	835.7	21413.617	6.190	17.650	3.0	0.54009	3.7	0.07044	1.3	0.358	438.8	5.6	438.5	13.1	437.8	76.4	438.8	5.6	100.2
21AR217_107	347.5	4612.143	0.914	16.723	7.8	0.35752	8.3	0.04615	1.7	0.205	290.8	4.8	310.4	22.1	460.9	179.3	290.8	4.8	63.1
21AR217_108	309.6	4109.975	3.016	15.157	10.1	0.23890	12.0	0.02791	3.2	0.269	177.5	5.6	217.5	23.5	677.6	247.3	177.5	5.6	26.2
21AR217_109	712.7	32593.407	2.737	16.289	2.6	0.81126	3.3	0.09713	1.7	0.526	597.6	10.0	603.2	15.1	625.2	60.8	597.6	10.0	95.6
21AR217_110	414.7	5046.187	1.272	16.294	7.0	0.45373	8.3	0.05660	2.6	0.318	354.9	9.1	379.9	26.1	536.0	171.2	354.9	9.1	66.2
21AR217_111	618.0	19690.805	3.473	15.061	2.3	1.03153	4.2	0.11462	3.4	0.815	699.5	22.6	719.7	21.6	783.9	50.9	699.5	22.6	89.2
21AR217_112	1033.6	17179.009	0.617	18.159	3.9	0.31203	5.0	0.04151	1.7	0.333	262.2	4.3	275.8	12.0	393.7	105.2	262.2	4.3	66.6
21AR217_113	265.5	603.447	1.061	7.866	29.3	0.13481	35.4	0.00944	6.6	0.186	60.6	4.0	128.4	42.7	1690.4	646.9	60.6	4.0	3.6
21AR217_114	293.1	5925.354	3.381	16.040	8.1	0.53356	8.5	0.06498	1.7	0.205	405.9	6.9	434.2	30.1	588.2	181.2	405.9	6.9	69.0
21AR217_115	864.1	6155.288	2.350	17.578	4.3	0.34250	9.2	0.04583	2.1	0.231	288.9	6.0	299.1	23.9	380.3	201.8	288.9	6.0	76.0
21AR217_116	575.1	42718.670	3.634	13.431	1.4	1.69445	2.3	0.16677	1.8	0.785	994.3	16.6	1006.4	14.7	1033.9	28.8	1033.9	28.8	96.2
21AR217_117	1374.1	76411.161	2.061	13.382	1.0	1.70268	1.9	0.16664	1.5	0.823	993.6	14.1	1009.5	11.9	1045.3	21.3	1045.3	21.3	95.1
21AR217_118	261.7	1892.928	0.982	10.736	22.7	0.22170	25.1	0.01887	3.2	0.126	120.5	3.8	203.3	46.3	1321.4	484.2	120.5	3.8	9.1
21AR217_119	530.8	2227.910	1.636	12.144	14.2	0.10900	17.5	0.01049	4.0	0.230	67.3	2.7	105.0	17.5	1079.5	343.0	67.3	2.7	6.2
21AR217_120	2074.5	22885.033	15.707	18.609	2.0	0.31101	4.0	0.04284	3.0	0.757	270.4	8.1	275.0	9.7	314.6	59.6	270.4	8.1	86.0
21AR217_121	421.6	5310.845	0.642	8.519	1.4	4.91590	2.9	0.31312	2.2	0.751	1756.0	33.2	1805.0	24.3	1862.8	34.3	1862.8	34.3	94.3
21AR217_122	528.8	12807.964	0.601	17.164	4.5	0.46924	5.7	0.06008	1.7	0.297	376.1	6.2	390.7	18.4	478.6	120.0	376.1	6.2	78.6
21AR217_123	1270.7	8315.337	30.318	17.249	2.3	0.55002	3.9	0.07063	3.0	0.770	439.9	12.8	445.0	14.1	472.3	55.3	439.9	12.8	93.2
21AR217_124	461.6	6319.027	8.309	12.937	2.3	1.72169	4.0	0.16780	2.0	0.492	1000.0	18.3	1016.7	25.9	1053.6	70.7	1053.6	70.7	94.9
21AR217_125	476.5	1783.015	0.885	10.238	19.0	0.12371	24.9	0.01007	4.1	0.167	64.6	2.7	118.4	27.8	1406.6	472.0	64.6	2.7	4.6
21AR217_126	375.6	7709.508	1.104	15.859	8.4	0.36181	9.5	0.04316	1.9	0.202	272.4	5.1	313.6	25.6	632.9	200.0	272.4	5.1	43.0
21AR217_127	393.9	4837.056	1.034	15.496	7.5	0.32634	8.0	0.03861	1.9	0.244	244.2	4.7	286.8	20.0	650.8	166.3	244.2	4.7	37.5
21AR217_128	404.4	9240.913	1.466	16.978	6.0	0.46435	8.1	0.05897	2.1	0.264	369.4	7.7	387.3	26.0	496.6	172.0	369.4	7.7	74.4
21AR217_129	1209.3	19565.373	2.538	18.193	3.6	0.31528	4.5	0.04234	2.0	0.439	267.3	5.2	278.3	11.0	372.5	91.4	267.3	5.2	71.8
21AR217_130	1038.1	424.188	0.725	4.158	33.5	0.02820	37.6	0.00096	14.3	0.380	6.2	0.9	28.2	10.5	2933.2	567.6	6.2	0.9	0.2
21AR217_131	144.2	10718.538	5.981	12.457	4.7	2.27275	8.1	0.20824	6.5	0.802	1219.4	72.5	1203.9	57.4	1176.9	96.1	1176.9	96.1	103.6
21AR217_132	834.3	15925.751	1.808	18.047	4.3	0.32020	4.8	0.04242	2.0	0.416	267.8	5.2	282.1	11.8	402.6	97.2	267.8	5.2	66.5
21AR217_133	169.8	6142.176	0.854	15.434	8.8	0.82541	10.5	0.09548	2.1	0.196	587.8	11.6	611.1	48.2	699.1	219.3	587.8	11.6	84.1
21AR217_134	367.0	8057.282	1.055	16.511	6.5	0.47775	8.9	0.05872	1.8	0.203	367.8	6.5	396.5	29.2	568.3	189.6	367.8	6.5	64.7
21AR217_135	294.7	3226.258	0.490	13.452	3.7	1.40249	9.6	0.14490	3.9	0.399	872.3	31.4	890.0	57.2	935.1	181.5	935.1	181.5	93.3
21AR217_136	339.3	8700.163	1.431	16.302	7.2	0.49669	8.1	0.06012	1.9	0.238	376.3	7.0	409.5	27.2	601.7	170.0	376.3	7.0	62.5
21AR217_137	265.3	21121.622	1.043	9.303	1.9	4.38159	2.7	0.29535	1.8	0.671	1668.2	26.8	1708.9	22.5	1759.9	36.9	1759.9	36.9	94.8
21AR217_138	701.4	43303.461	0.873	13.171	1.9	1.68262	3.1	0.16016	2.4	0.787	957.7	21.8	1002.0	19.8	1101.0	38.3	1101.0	38.3	87.0
21AR217_139	466.6	8098.635	1.011	17.103	7.8	0.34578	8.7	0.04390	2.2	0.250	277.0	5.9	301.5	22.7	497.2	185.8	277.0	5.9	55.7
21AR217_140	343.0	22785.540	1.558	13.550	2.6	1.72714	3.4	0.16984	2.0	0.596	1011.2	19.1	1018.7	22.0	1035.7	55.4	1035.7	55.4	97.6
21AR217_141	459.6	11781.355	2.189	17.342	5.7	0.56380	6.8	0.07189	2.2	0.320	447.6	9.4	454.0	24.9	487.7	142.3	447.6	9.4	91.8
21AR217_142	374.0	8350.812	0.926	16.773	5.9	0.50037	7.0	0.06242	1.8	0.263	390.3	6.9	412.0	23.6	536.1	146.9	390.3	6.9	72.8
21AR217_143	383.8	30222.966	0.886	13.667	2.3	1.63446	3.2	0.16123	2.0	0.633	963.6	18.2	983.6	20.2	1029.3	50.3	1029.3	50.3	93.6
21AR217_144	1068.3	12526.562	1.048	18.226	3.0	0.41915	3.6	0.05608	1.6	0.457	351.7	5.6	355.4	10.7	380.7	71.5	351.7	5.6	92.4
21AR217_145	509.1	13204.666	1.316	17.566	3.6	0.50690	5.4	0.06524	3.5	0.649	407.4	13.9	416.4	18.6	467.3	91.6	407.4	13.9	87.2
21AR217_146	207.0	1052.072	1.012	10.804	17.9	0.13911	34.3	0.01264	6.6	0.193	81.0	5.3	132.3	42.5	1193.9	668.0	81.0	5.3	6.8
21AR217_147	281.6	23830.317	1.144	11.279	2.5	2.75184	5.0	0.22447	4.3	0.854	1305.5	50.4	1342.6	37.2	1403.1	49.8	1403.1	49.8	93.0
21AR217_148	316.3	10564.482	0.916	16.662	7.3	0.50820	8.3	0.06226	1.9	0.235	389.4	7.4	417.2	28.4	575.3	175.3	389.4	7.4	67.7
21AR217_149	606.3	36448.422	1.018	9.132	1.2	4.47995	2.9	0.29494	2.6	0.892	1666.2	37.5	1727.3	23.7	1802.9	23.5	1802.9	23.5	92.4
21AR217_150	734.8	7998.541	0.819	18.204	4.2	0.31292	7.1	0.04226	1.6	0.230	266.8	4.3	276.4	17.3	359.8	156.8	266.8	4.3	74.1
21AR217_151	395.4	11580.805	1.032	16.792	3.4	0.76933	4.5	0.09477	2.1	0.478	583.7	12.0	579.4	19.8	563.5	85.9	583.7	12.0	103.6
21AR217_152	563.7	6520.331	0.853	17.231	6.5	0.22417	10.1	0.02885	2.4	0.239	183.4	4.4	205.4	18.8	467.1	217.6	183.4	4.4	39.3
21AR217_153	503.2	7764.371	1.180	17.183	6.6	0.30765	7.7	0.03944	2.1	0.274	249.4	5.1	272.4	18.4	476.0	163.7	249.4	5.1	52.4
21AR217_154	350.5	3147.666	1.222	15.450	9.3	0.32672	11.9	0.03903	2.0	0.170	246.8	4.9	287.1	29.8	630.1	253.3	246.8	4.9	39.2
21AR217_155	584.3	9657.386	0.821	17.659	5.5	0.30383	6.3	0.03959	1.8	0.282	250.3	4.4	269.4	15.0	439.9	135.2	250.3	4.4	56.9
21AR217_156	217.9	4157.726	2.079	14.656	10.3	0.40397	11.2	0.04486	2.9	0.255	282.9	7.9	344.5	32.8	785.1	228.3	282.9	7.9	36.0
21AR217_157	369.5	24938.568	2.778	13.448	2.2	1.72139	3.1	0.16794	1.7	0.546	1000.7	15.6	1016.5	19.9	1051.6	52.2	1051.6	52.2	95.2
21AR217_158	514.2	19594.216	1.510	12.772	1.9	1.87454	3.1	0.17397	2.2	0.690	1033.9	20.7	1072.1	20.8	1151.5	45.2	1151.5	45.2	89.8
21AR217_159	860.5	5552.016	0.623	17.133	5.9	0.21926	10.0	0.02846	2.0	0.197	180.9	3.5	201.3	18.2	448.1	217.5	180.9	3.5	40.4
21AR217_160	225.6	9005.131	1.570	13.332	3.0	1.73657	4.4	0.17052	2.1	0.476	1015.0	19.8	1022.2	28.5	1038.5	78.7	1038.5	78.7	97.7
21AR217_161	329.6	5091.014	0.948	15.340	8.2	0.33502	10.0	0.03903	2.4	0.235	246.8	5.7	293.4	25.5	683.9	208.1	246.8	5.7	36.1
21AR217_162	741.6	18246.724	2.133	17.460	2.8	0.56345	3.9	0.07247	1.8	0.469	451.0	7.9	453.8	14.2	468.8	75.9	451.0	7.9	96.2
21AR217_163	300.4	1225.822	1.919	7.825	29.1	0.15895	30.6	0.00988	6.2	0.203	63.4	3.9	149.8	42.6	1906.0	540.7	63.4	3.9	3.3
21AR217_164	489.0	3751.681	1.185	13.655	11.5	0.17983	14.3	0.01889	2.5	0.177	120.6	3.0	167.9	22.1	901.4	290.5	120.6	3.0	13.4
21AR217_165	493.2	12275.634	0.942	17.414	5.0	0.49325	5.8	0.06368	2.3	0.396	398.0	8.8	407.1	19.3	460.4	117.5	398.0	8.8	86.4
21AR217_166	313.2	26695.272	3.511	12.004	2.0	2.31371	3.0	0.20293	2.1	0.691	1191.0	22.6	1216.5	21.3	1262.8	42.4	1262.8	42.4	94.3
21AR217_167	460.4	7133.394	1.045	17.301	6.5	0.33748	7.6	0.04395	2.2	0.287	277.2	5.9	295.3	19.5	441.2	162.0	277.2	5.9	62.8
21AR217_168	260.4	3445.131	1.416	16.720	7.2	0.33081	12.4	0.04322	2.6	0.209	272.7	6.9	290.2	31.3	434.1	269.9	272.7	6.9	62.8

21AR217_169	314.4	11544.891	0.950	16.735	6.0	0.66579	6.5	0.08259	1.5	0.236	511.6	7.6	518.2	26.5	548.3	138.4	511.6	7.6	93.3
21AR217_170	138.1	1954.091	0.848	11.449	16.5	0.42970	18.8	0.03902	3.5	0.186	246.8	8.5	363.0	57.5	1194.6	365.6	246.8	8.5	20.7
21AR217_171	414.7	3149.555	1.721	14.309	3.2	0.95779	3.8	0.10667	1.5	0.404	653.4	9.6	682.1	19.0	779.3	73.6	653.4	9.6	83.8
21AR217_172	53.7	1087.576	0.755	8.596	21.4	0.56545	29.7	0.03965	5.5	0.185	250.7	13.5	455.1	108.9	1687.2	540.8	250.7	13.5	14.9
21AR217_173	1176.0	115178.566	1.761	7.003	1.2	6.83134	3.5	0.34831	3.3	0.938	1926.5	54.3	2089.8	30.8	2255.5	20.8	2255.5	20.8	85.4
21AR217_174	284.8	4036.558	0.538	13.866	13.0	0.27105	13.8	0.02876	2.8	0.202	182.8	5.0	243.5	30.0	880.2	280.9	182.8	5.0	20.8
21AR217_175	829.1	20095.997	0.807	18.102	4.2	0.46759	4.6	0.06231	1.6	0.350	389.7	6.1	389.5	14.9	389.5	97.1	389.7	6.1	100.0
21AR217_176	736.9	21963.772	1.687	17.535	3.1	0.57916	3.9	0.07463	1.7	0.448	464.0	7.8	463.9	14.4	464.6	76.8	464.0	7.8	99.9
21AR217_177	303.8	5524.250	1.391	15.836	12.6	0.37535	13.9	0.04504	2.0	0.145	284.0	5.6	323.6	38.5	620.2	296.7	284.0	5.6	45.8
21AR217_178	271.5	5533.514	1.885	15.309	4.9	0.81491	8.5	0.09381	6.3	0.738	578.1	34.7	605.2	38.8	709.1	122.0	578.1	34.7	81.5
21AR217_179	442.7	8809.263	1.084	16.913	6.4	0.30829	8.1	0.03896	2.3	0.281	246.4	5.5	272.9	19.4	507.6	171.3	246.4	5.5	48.5
21AR217_180	775.7	10760.125	2.061	18.301	4.3	0.31440	6.5	0.04279	1.8	0.276	270.1	4.7	277.6	15.7	342.1	140.6	270.1	4.7	79.0
21AR217_181	622.8	12458.959	1.026	17.938	5.3	0.31710	6.5	0.04220	2.8	0.424	266.5	7.2	279.7	15.9	392.6	132.4	266.5	7.2	67.9
21AR217_182	101.9	9282.190	1.526	11.003	4.5	2.85663	5.1	0.23212	1.6	0.319	1345.6	19.7	1370.6	38.2	1410.5	92.2	1410.5	92.2	95.4
21AR217_183	871.0	15761.722	0.760	17.923	5.4	0.28135	6.4	0.03719	3.1	0.480	235.4	7.1	251.7	14.3	408.0	126.3	235.4	7.1	57.7
21AR217_184	296.0	28826.397	1.743	10.120	2.5	3.00104	5.5	0.22131	4.9	0.885	1288.8	56.7	1407.9	41.8	1593.8	47.8	1593.8	47.8	80.9
21AR217_185	467.3	21478.375	1.919	13.037	2.2	1.60484	4.1	0.15303	3.0	0.739	917.9	25.7	972.1	25.5	1097.5	54.9	1097.5	54.9	83.6
21AR217_186	898.1	297.067	1.238	4.755	72.7	0.02466	77.9	0.00106	18.3	0.235	6.9	1.3	24.7	19.0	2540.3	1324.2	6.9	1.3	0.3
21AR217_187	348.8	5413.083	0.529	16.286	7.1	0.45584	8.0	0.05632	1.5	0.191	353.2	5.3	381.4	25.6	557.1	172.3	353.2	5.3	63.4
21AR217_188	152.0	4666.687	1.115	15.199	7.4	0.64308	9.5	0.07454	2.5	0.259	463.4	11.0	504.2	37.7	694.7	195.5	463.4	11.0	66.7
21AR217_189	1113.8	2658.094	1.573	16.800	5.0	0.16893	9.4	0.02273	3.8	0.403	144.9	5.4	158.5	13.8	368.2	194.3	144.9	5.4	39.3
21AR217_190	293.3	2898.734	1.179	16.180	6.1	0.57445	13.4	0.07352	2.0	0.146	457.3	8.6	460.9	49.6	479.7	292.6	457.3	8.6	95.3
21AR217_191	560.7	5497.725	0.862	16.918	5.3	0.30940	11.0	0.03983	2.0	0.186	251.7	5.0	273.7	26.3	467.0	238.5	251.7	5.0	53.9
21AR217_192	529.9	37987.460	6.453	13.446	1.6	1.66666	2.3	0.16387	1.5	0.645	978.2	13.4	995.9	14.5	1035.9	35.3	1035.9	35.3	94.4
21AR217_193	700.8	17315.311	1.619	17.473	4.2	0.47710	4.9	0.06149	1.6	0.327	384.7	6.0	396.1	16.1	464.3	102.8	384.7	6.0	82.9
21AR217_194	1013.7	9130.761	3.577	12.888	1.2	1.84352	3.0	0.17620	1.6	0.510	1046.2	15.0	1061.1	20.0	1092.8	52.4	1092.8	52.4	95.7
21AR217_195	580.7	18129.945	55.098	17.027	3.5	0.65707	4.5	0.08243	2.3	0.519	510.6	11.4	512.8	18.0	523.6	83.9	510.6	11.4	97.5
21AR217_196	227.6	3907.940	4.009	14.557	10.6	0.39540	13.5	0.04420	2.6	0.195	278.8	7.2	338.3	38.9	771.2	279.1	278.8	7.2	36.2
21AR217_197	390.1	4075.986	1.399	16.678	6.2	0.46378	12.0	0.05938	1.7	0.145	371.9	6.3	386.9	38.5	478.6	261.6	371.9	6.3	77.7
21AR217_198	339.4	2811.967	1.990	12.011	15.6	0.24085	18.1	0.02238	3.1	0.173	142.7	4.4	219.1	35.7	1149.1	354.7	142.7	4.4	12.4
21AR217_199	450.7	16567.295	5.415	15.781	4.6	0.76312	5.1	0.08879	2.1	0.402	548.4	10.8	575.8	22.5	686.5	99.9	548.4	10.8	79.9
21AR217_200	551.2	15889.850	1.091	16.971	3.7	0.57603	4.7	0.07215	2.1	0.456	449.1	9.2	461.9	17.3	527.2	90.9	449.1	9.2	85.2
21AR217_201	741.2	2349.267	1.441	16.864	3.7	0.34611	4.8	0.04739	1.7	0.353	298.5	5.0	301.8	12.7	328.7	102.9	298.5	5.0	90.8
21AR217_202	331.8	51029.359	1.495	5.847	1.3	8.93288	1.9	0.38003	1.4	0.736	2076.4	24.3	2331.2	17.0	2563.1	21.1	2563.1	21.1	81.0
21AR217_203	576.9	11449.041	1.438	17.338	6.3	0.33060	6.8	0.04251	1.8	0.273	268.4	4.9	290.0	17.1	469.3	144.6	268.4	4.9	57.2
21AR217_204	1019.1	6952.737	1.073	18.437	3.3	0.29068	7.2	0.04042	1.7	0.241	255.5	4.3	259.1	16.4	293.2	159.0	255.5	4.3	87.1
21AR217_205	265.2	5671.167	1.825	15.703	7.9	0.47433	10.7	0.05625	2.0	0.183	352.8	6.7	394.2	35.0	645.8	226.5	352.8	6.7	54.6
21AR217_206	74.7	2068.101	0.474	10.528	23.9	0.74092	26.0	0.06063	7.2	0.276	379.5	26.5	562.9	112.6	1397.0	481.5	379.5	26.5	27.2
21AR217_207	325.8	3427.024	1.027	13.996	9.8	0.26566	14.3	0.02864	2.2	0.152	182.0	3.9	239.2	30.4	847.4	293.4	182.0	3.9	21.5
21AR217_208	413.4	10098.621	1.327	18.010	3.8	0.46958	4.6	0.06295	1.8	0.386	393.5	6.8	390.9	15.0	376.2	96.2	393.5	6.8	104.6
21AR217_209	747.7	15485.967	1.459	17.713	4.3	0.42672	5.4	0.05572	1.5	0.286	349.6	5.2	360.8	16.3	435.0	114.7	349.6	5.2	80.4
21AR217_210	196.4	3219.340	3.154	11.406	10.0	0.47017	10.7	0.04091	2.7	0.254	258.5	6.9	391.3	34.7	1278.1	201.3	258.5	6.9	20.2
21AR217_211	457.2	3200.223	0.377	15.610	9.5	0.21210	13.7	0.02583	2.0	0.144	164.4	3.2	195.3	24.3	588.3	293.9	164.4	3.2	27.9
21AR217_212	497.8	9331.069	1.192	17.127	5.0	0.36902	6.6	0.04706	3.7	0.563	296.4	10.8	318.9	18.1	487.7	120.7	296.4	10.8	60.8
21AR217_213	225.4	3453.507	0.649	13.786	8.2	0.61215	11.1	0.06489	2.8	0.253	405.3	11.0	484.9	42.8	882.3	222.2	405.3	11.0	45.9
21AR217_214	555.0	70820.052	1.596	8.652	1.6	5.22005	2.6	0.32792	2.0	0.778	1828.3	32.2	1855.9	22.1	1887.7	29.4	1887.7	29.4	96.9
21AR217_215	1367.8	40686.071	5.288	18.129	2.2	0.50819	3.6	0.06722	2.8	0.779	419.4	11.5	417.2	12.4	406.3	50.9	419.4	11.5	103.2
21AR217_216	59.8	1218.210	1.172	8.742	23.7	0.79406	28.2	0.05581	4.5	0.159	350.1	15.3	593.5	127.0	1683.0	517.1	350.1	15.3	20.8
21AR217_217	553.6	9140.364	0.938	16.598	7.5	0.30964	8.8	0.03809	2.4	0.273	241.0	5.7	273.9	21.0	566.3	183.4	241.0	5.7	42.6
21AR217_218	149.7	5390.482	0.994	8.499	2.3	4.59454	5.8	0.28934	4.5	0.778	1638.2	64.9	1748.3	48.1	1883.3	65.2	1883.3	65.2	87.0
21AR217_219	275.2	7987.271	2.095	16.118	6.4	0.57810	7.3	0.06955	2.7	0.365	433.5	11.2	463.2	27.3	614.6	147.5	433.5	11.2	70.5
21AR217_220	642.4	1055.150	0.226	15.111	6.6	0.27694	16.1	0.03838	1.6	0.100	242.8	3.8	248.2	35.4	301.3	365.2	242.8	3.8	80.6
21AR217_221	179.9	5545.517	1.617	14.977	7.7	0.74660	9.8	0.08431	2.1	0.218	521.8	10.8	566.2	42.7	750.0	203.1	521.8	10.8	69.6
21AR217_222	337.1	9460.358	1.591	17.356	4.8	0.56383	5.6	0.07286	1.9	0.344	453.3	8.5	454.0	20.6	458.4	117.0	453.3	8.5	98.9
21AR217_223	157.9	809.829	1.155	7.019	30.3	0.22705	33.0	0.01305	8.7	0.264	83.6	7.2	207.8	62.0	2045.9	566.4	83.6	7.2	4.1
21AR217_224	375.4	7318.509	0.440	16.318	4.6	0.51158	6.8	0.06245	2.5	0.369	390.5	9.4	419.5	23.2	583.1	136.3	390.5	9.4	67.0
21AR217_225	239.3	21947.359	1.817	11.664	2.4	2.56898	3.2	0.21839	1.9	0.599	1273.3	22.3	1291.9	23.5	1323.6	50.0	1323.6	50.0	96.2
21AR217_226	274.6	5863.391	3.471	16.422	7.4	0.50197	11.2	0.06216	2.3	0.207	388.8	8.7	413.0	37.9	552.0	238.8	388.8	8.7	70.4
21AR217_227	217.6	1234.667	0.728	10.245	23.6	0.18298	27.0	0.01535	6.1	0.224	98.2	5.9	170.6	42.4	1349.4	509.6	98.2	5.9	7.3
21AR217_228	157.1	20069.390	0.937	8.207	1.7	5.76635	2.5	0.34422	1.6	0.643	1906.9	26.2	1941.4	21.3	1979.1	33.6	1979.1	33.6	96.4
21AR217_229	88.5	2359.375	1.472	12.664	12.1	0.56334	13.5	0.05595	4.0	0.297	351.0	13.7	453.7	49.5	1015.5	261.8	351.0	13.7	34.6
21AR217_230	137.8	4071.806	0.406	14.379	12.3	0.64003	14.4	0.07035	2.5	0.173	438.3	10.5	502.3	57.0	806.7	296.9	438.3	10.5	54.3
21AR217_231	617.5	6304.864	1.228	17.074	5.1	0.30810	6.0	0.03974	1.8	0.295	251.2	4.4	272.7	14.5	462.3	128.0	251.2	4.4	54.3
21AR217_232	435.3	7927.820	1.515	16.626	6.1	0.32462	7.2	0.04039	1.8	0.245	255.3	4.4	285.5	17.9	541.5	152.6	255.3	4.4	47.1
21AR217_233	632.8	6199.178	0.750	16.577	6.3	0.31260	7.2	0.03912	1.7	0.230	247.4	4.0	276.2	17.4	528.9	153.8	247.4	4.0	46.8
21AR217_234	221.2	3868.586	1.432	14.775	8.6	0.41556	14.5	0.04718	2.7	0.183	297.2	7.7	352.9	43.3	738.8	302.7	297.2	7.7	40.2
21AR217_235	592.4	469.248	0.735	12.484	12.7	0.10879	109.2	0.01624	2.4	0.022	103.9	2.5	104.9	108.8	128.8		103.9	2.5	80.7
21AR217_236	628.8	17895.085	1.291	17.664	3.3	0.57333	4.5	0.07470	2.0	0.442	464.4	8.9	460.2	16.5	439.9	89.2	464.4	8.9	105.6
21AR217_237	579.3	7000.958	1.698	16.851	9.1	0.21045	10.4	0.02656	1.8	0.176	169.0	3.1	193.9	18.3	510.4	224.8	169.0	3.1	33.1
21AR217_238	146.9	8973.931	2.274	13.474	6.1	1.71441	6.6	0.17144	1.8	0.265	1020.0	16.6	1013.9	42.5	1001.7	129.7	1001.7	129.7	101.8
21AR217_239	468.6	1261.528	29.134	15.422	6.6	0.46081	28.2	0.06290	2.2	0.077	393.2	8.3	384.8	90.3	335.7	639.6	393.2	8.3	117.1
21AR217_240	309.6	6335.363	0.998	17.155	4.9	0.47676	6.5	0.06190	1.9	0.296	387.1	7.2	395.8	21.3	448.0	138.0	387.1	7.2	86.4
21AR217_241	287.3	7019.672	1.469	16.607	5.5	0.65226	8.6	0.08154	1.7	0.201	505.3	8.4	509.9	34.3	531.3	183.5	505.3	8.4	95.1
21AR217_242	233.9	3604.842	0.551	15.763	9.9	0.31724	13.9	0.03890	2.7	0.195	246.0	6.5	279.8	33.9	573.3	296.1	246.0	6.5	42.9
21AR217_243	75.6	981.480	1.289	9.038	23.0	0.51957	31.2	0.03915	6.4	0.205	247.6	15.5	424.9	108.3	1553.3	576.2	247.6	15.5	15.9
21AR217_244	170.9	4804.968	1.190	16.045	7.2	0.66189	11.1	0.08119	3.7	0.331	503.2	17.8	515.8	44.8	572.6	227.6	503.2	17.8	87.9
21AR217_245	359.3	1487.680	0.889	9.363	17.2	0.12958	24.7	0.00967	5.6	0.228	62.0	3.5	123.7	28.7	1572.0	451.4	62.0	3.5	3.9
21AR217_246	1020.6	2244.266	3.903	15.432	2.0	0.70862	2.7	0.08779	1.4	0.505	542.5	7.1	543.9	11.4	551.0	50.8	542.5	7.1	98.4
21AR217_247	192.5	4619.641	1.512	15.235	7.2	0.88851	7.9	0.10228	1.9	0.239	627.8	11.3	645.6	37.8	709.2	163.2	627.8	11.3	88.5

21AR221

21AR221_1	185.6	3241.245	0.294	13.676	11.2	0.36895	12.1	0.03910	2.3	0.190	247.2	5.6	318.9	33.3	882.8	246.9	247.2	5.6	28.0
21AR221_2	157.6	3836.481	0.954	12.623	13.9	0.42090	14.5	0.04058	2.9	0.200	256.4	7.3	356.7	43.6	1075.6	285.3	256.4	7.3	23.8
21AR221_3	362.5	5512.995	2.007	16.802	6.7	0.21890	7.8	0.02800	2.4	0.310	178.0	4.3	201.0	14.3	481.0	164.2	178.0	4.3	37.0
21AR221_4	267.7	5668.645	1.511	16.473	6.7	0.32555	7.6	0.04080	2.4	0.311	257.8	6.0	286.2	19.0	526.1	158.8	257.8	6.0	49.0
21AR221_5	246.1	1677.543	0.903	10.880	17.8	0.14821	20.0	0.01293	3.3	0.165	82.8	2.7	140.3	26.3	1273.1	386.6	82.8	2.7	6.5
21AR221_6	1143.8	212.926	0.611	5.302	32.1	0.01917	57.3	0.00111	12.9	0.225	7.1	0.9	19.3	10.9	2037.5	1008.4	7.1	0.9	0.4
21AR221_7	199.6	11908.718	2.619	12.860	2.8	1.82302	4.4	0.17347	1.5	0.343	1031.2	14.4	1053.8	28.8	1101.8	82.4	1101.8	82.4	93.6
21AR221_8	236.1	5046.730	1.294	15.260	11.0	0.36058	11.8	0.04185	1.8	0.154	264.3	4.7	312.6	31.7	691.5	248.2	264.3	4.7	38.2
21AR221_9	542.0	13982.408	2.266	17.029	3.2	0.58865	5.1	0.07431	1.8	0.364	462.1	8.2	470.0	19.1	509.8	103.8	462.1	8.2	90.6
21AR221_10	1353.8	508.096	0.884	5.977	38.6	0.01998	46.4	0.00103	7.1	0.154	6.6	0.5	20.1	9.2	2241.6	804.9	6.6	0.5	0.3
21AR221_11	163.0	4493.009	1.032	14.511	11.2	0.43509	11.6	0.04802	2.4	0.203	302.4	7.0	366.8	35.8	798.0	238.8	302.4	7.0	37.9
21AR221_12	410.3	8069.914	0.856	17.011	6.3	0.31121	7.4	0.03981	1.6	0.223	251.7	4.0	275.1	17.7	480.5	158.7	251.7	4.0	52.4
21AR221_13	437.1	391.308	1.385	4.860	46.3	0.03356	56.2	0.00141	18.9	0.336	9.1	1.7	33.5	18.5	2589.3	900.6	9.1	1.7	0.3
21AR221_14	555.2	16879.603	2.606	18.164	4.0	0.44220	4.5	0.05948	1.9	0.411	372.5	6.7	371.8	14.1	368.7	93.2	372.5	6.7	101.0
21AR221_15	605.0	15384.264	1.453	18.997	3.5	0.28036	4.2	0.03956	1.7	0.408	250.1	4.2	250.9	9.4	260.0	88.5	250.1	4.2	96.2
21AR221_16	279.1	8895.851	1.242	17.244	5.2	0.55914	6.9	0.07241	1.8	0.264	450.7	7.9	451.0	25.1	453.4	147.7	450.7	7.9	99.4
21AR221_17	518.3	23079.329	0.911	17.221	2.7	0.65899	3.5	0.08373	1.9	0.544	518.4	9.4	514.0	14.0	495.6	64.2	518.4	9.4	104.6
21AR221_18	53.1	5106.099	1.426	12.210	7.0	2.00751	7.8	0.18515	2.5	0.316	1095.0	24.9	1118.0	53.0	1163.9	147.2	1163.9	147.2	94.1
21AR221_19	183.4	6710.433	1.333	15.375	7.0	0.52063	9.6	0.06042	1.8	0.184	378.2	6.5	425.6	33.3	692.0	200.6	378.2	6.5	54.6
21AR221_20	52.5	2006.038	0.885	10.739	16.8	0.76343	18.9	0.06474	4.0	0.215	404.4	15.9	576.0	83.0	1328.2	357.3	404.4	15.9	30.4
21AR221_21	415.1	4151.935	1.080	16.843	5.5	0.31097	11.6	0.04066	1.7	0.149	256.9	4.4	274.9	28.0	432.0	255.9	256.9	4.4	59.5
21AR221_22	167.6	1863.939	1.205	9.440	30.0	0.24627	32.9	0.01826	5.1	0.155	116.6	5.9	223.5	66.1	1584.0	612.6	116.6	5.9	7.4
21AR221_23	752.0	33487.818	3.213	17.024	2.2	0.74170	2.8	0.09284	1.6	0.554	572.3	8.5	563.4	12.1	528.6	51.1	572.3	8.5	108.3
21AR221_24	505.4	14014.814	1.256	17.558	3.6	0.46103	4.8	0.06018	2.2	0.446	376.7	7.9	385.0	15.5	436.0	96.3	376.7	7.9	86.4
21AR221_25	184.6	10480.836	1.290	15.411	5.0	0.90033	6.1	0.10345	1.7	0.286	634.6	10.5	651.9	29.2	713.2	123.4	634.6	10.5	89.0
21AR221_26	82.9	2822.883	0.884	13.184	13.7	0.54678	16.5	0.05637	2.9	0.174	353.5	9.9	442.9	59.3	939.3	334.0	353.5	9.9	37.6
21AR221_27	348.0	8262.198	1.214	16.302	6.9	0.34102	8.0	0.04174	2.0	0.252	263.6	5.2	297.9	20.6	577.6	168.0	263.6	5.2	45.6
21AR221_28	98.3	2162.882	0.750	11.321	15.9	0.41463	18.9	0.03691	3.5	0.187	233.7	8.1	352.2	56.1	1233.7	364.1	233.7	8.1	18.9
21AR221_29	336.4	7242.669	2.548	16.212	5.5	0.36079	5.8	0.04403	1.6	0.278	277.7	4.4	312.8	15.7	584.0	121.8	277.7	4.4	47.6
21AR221_30	193.3	2755.915	1.493	14.152	9.9	0.23381	18.3	0.02602	2.9	0.157	165.6	4.7	213.3	35.2	780.5	380.0	165.6	4.7	21.2
21AR221_31	152.7	3967.756	1.904	15.743	8.8	0.38258	10.4	0.04644	2.8	0.270	292.6	8.1	328.9	29.3	595.5	217.5	292.6	8.1	49.1
21AR221_32	335.0	2265.683	2.386	13.035	16.6	0.09552	17.7	0.00986	4.4	0.248	63.3	2.8	92.6	15.7	936.7	352.0	63.3	2.8	6.8
21AR221_33	374.6	4972.020	1.551	14.839	9.4	0.23223	11.3	0.02613	2.1	0.185	166.3	3.4	212.0	21.7	757.6	234.8	166.3	3.4	21.9
21AR221_34	191.5	2760.205	0.163	15.329	7.1	0.60685	10.0	0.07356	2.6	0.258	457.6	11.4	481.6	38.4	598.4	209.5	457.6	11.4	76.5
21AR221_35	417.8	510.869	0.791	8.581	25.8	0.34310	64.5	0.02798	5.4	0.083	177.9	9.4	299.5	167.7	1403.8	1264.9	177.9	9.4	12.7

21AR221_36	317.4	1993.824	1.003	9.738	22.9	0.12532	23.7	0.00952	4.7	0.197	61.1	2.8	119.9	26.8	1538.5	437.8	61.1	2.8	4.0
21AR221_37	222.0	4184.310	1.259	16.124	8.2	0.33148	13.6	0.04098	2.1	0.152	258.9	5.3	290.7	34.3	555.4	293.1	258.9	5.3	46.6
21AR221_38	264.1	14868.143	4.924	15.325	4.3	0.93040	5.4	0.10502	1.8	0.326	643.8	10.8	667.8	26.3	750.9	107.4	643.8	10.8	85.7
21AR221_39	432.6	2862.814	0.662	13.369	13.2	0.14932	15.4	0.01552	2.1	0.139	99.3	2.1	141.3	20.4	923.0	314.4	99.3	2.1	10.8
21AR221_40	528.9	20457.208	1.371	18.137	3.7	0.44753	4.3	0.05964	1.9	0.442	373.5	6.8	375.6	13.4	389.4	85.8	373.5	6.8	95.9
21AR221_41	921.0	4198.888	0.707	16.055	2.9	0.64902	4.1	0.08001	1.7	0.409	496.2	8.1	507.9	16.5	561.8	82.0	496.2	8.1	88.3
21AR221_42	1221.9	100.298	0.516	5.390	27.8	0.00835	76.3	0.00168	5.6	0.073	10.8	0.6	8.4	6.4	NA		10.8	0.6	
21AR221_43	307.8	3999.542	1.944	14.258	10.5	0.18429	11.7	0.02009	3.0	0.252	128.2	3.8	171.7	18.5	824.1	237.0	128.2	3.8	15.6
21AR221_44	747.3	18891.118	0.978	18.022	3.0	0.42808	3.8	0.05675	1.6	0.405	355.8	5.4	361.8	11.7	401.4	78.7	355.8	5.4	88.6
21AR221_45	711.3	3761.608	2.023	17.646	4.2	0.28940	4.8	0.03978	1.4	0.287	251.5	3.4	258.1	11.1	319.7	105.6	251.5	3.4	78.7
21AR221_46	350.1	2524.693	0.853	13.316	15.6	0.20805	18.3	0.02169	2.6	0.142	138.3	3.6	191.9	32.0	916.3	373.8	138.3	3.6	15.1
21AR221_47	94.1	3935.483	1.001	10.763	18.5	0.68579	20.4	0.05555	3.7	0.183	348.5	12.6	530.3	84.3	1416.7	384.5	348.5	12.6	24.6
21AR221_48	551.5	13952.675	0.931	18.320	3.3	0.28677	4.9	0.03877	1.3	0.275	245.2	3.2	256.0	11.0	357.0	105.3	245.2	3.2	68.7
21AR221_49	1244.5	1857.656	0.617	9.258	24.5	0.03285	25.4	0.00236	5.1	0.199	15.2	0.8	32.8	8.2	1639.8	464.7	15.2	0.8	0.9
21AR221_50	125.9	2502.549	0.872	11.305	22.4	0.41338	24.3	0.03612	2.6	0.108	228.8	5.9	351.3	72.2	1269.9	472.9	228.8	5.9	18.0
21AR221_51	354.1	16455.202	4.729	15.079	3.1	0.96829	3.7	0.10691	1.5	0.417	654.8	9.6	687.6	18.5	797.4	70.4	654.8	9.6	82.1
21AR221_52	705.0	12740.260	0.885	18.747	3.5	0.27503	4.1	0.03821	1.7	0.414	241.7	4.0	246.7	9.0	295.3	84.9	241.7	4.0	81.9
21AR221_53	328.3	9382.957	3.044	17.028	4.9	0.50341	5.6	0.06385	2.3	0.410	399.0	8.9	414.0	19.1	499.7	112.7	399.0	8.9	79.8
21AR221_54	290.3	10713.660	1.792	16.942	3.7	0.71368	4.9	0.08993	1.7	0.340	555.1	8.9	546.9	20.7	513.9	101.2	555.1	8.9	108.0
21AR221_55	548.6	6735.916	0.982	15.917	7.9	0.17093	9.8	0.02047	1.8	0.183	130.6	2.3	160.2	14.5	624.3	207.9	130.6	2.3	20.9
21AR221_56	365.9	12086.649	1.072	17.038	5.4	0.46320	5.9	0.05855	1.5	0.248	366.8	5.2	386.5	19.0	507.0	125.9	366.8	5.2	72.3
21AR221_57	338.0	5268.182	0.683	16.816	6.7	0.30177	7.6	0.03867	1.8	0.233	244.6	4.2	267.8	17.8	476.6	162.5	244.6	4.2	51.3
21AR221_58	567.8	16964.183	3.806	17.983	3.2	0.45199	4.3	0.05976	2.1	0.483	374.2	7.5	378.7	13.6	407.2	84.0	374.2	7.5	91.9
21AR221_59	1124.6	10041.652	1.749	18.892	2.4	0.30115	3.9	0.04241	1.4	0.351	267.7	3.6	267.3	9.2	264.5	84.2	267.7	3.6	101.2
21AR221_60	63.4	7917.184	1.825	11.305	7.9	2.57638	8.5	0.21581	1.6	0.192	1259.7	18.6	1294.0	61.9	1352.1	160.3	1352.1	160.3	93.2
21AR221_61	304.7	1544.696	1.363	10.877	19.5	0.10960	21.0	0.00961	3.9	0.184	61.7	2.4	105.6	21.1	1263.3	404.7	61.7	2.4	4.9
21AR221_62	280.5	9749.361	0.875	17.256	4.9	0.55131	5.6	0.07091	1.6	0.290	441.6	7.0	445.9	20.4	468.8	119.6	441.6	7.0	94.2
21AR221_63	323.8	12713.792	0.923	17.161	5.4	0.45227	6.2	0.05754	1.9	0.303	360.7	6.6	378.9	19.6	492.6	130.6	360.7	6.6	73.2
21AR221_64	654.5	8478.702	0.782	18.377	3.7	0.27158	5.9	0.03746	2.1	0.363	237.1	5.0	244.0	12.8	311.5	124.9	237.1	5.0	76.1
21AR221_65	529.3	14099.218	24.411	17.727	3.5	0.43910	4.4	0.05768	1.6	0.363	361.5	5.6	369.6	13.5	421.9	91.0	361.5	5.6	85.7
21AR221_66	83.4	1230.984	0.887	9.659	15.6	0.43928	28.3	0.03448	4.6	0.161	218.5	9.8	369.7	87.8	1476.6	532.7	218.5	9.8	14.8
21AR221_67	495.9	5838.758	1.273	16.972	5.8	0.18104	9.1	0.02334	3.2	0.356	148.7	4.8	169.0	14.1	463.6	187.8	148.7	4.8	32.1
21AR221_68	98.6	2868.434	0.963	11.455	17.4	0.46487	19.3	0.04111	3.7	0.191	259.7	9.4	387.6	62.4	1246.7	372.6	259.7	9.4	20.8
21AR221_69	1207.0	761.008	2.197	7.674	19.7	0.01734	28.4	0.00112	8.7	0.306	7.2	0.6	17.5	4.9	1834.3	491.4	7.2	0.6	0.4
21AR221_70	108.4	10421.753	1.917	11.414	4.4	2.23681	5.0	0.18875	1.4	0.291	1114.6	14.8	1192.7	35.0	1337.9	92.2	1337.9	92.2	83.3
21AR221_71	178.7	2438.307	0.628	13.060	14.8	0.26354	17.9	0.02716	3.6	0.200	172.7	6.1	237.5	37.8	940.5	359.3	172.7	6.1	18.4
21AR221_72	861.4	418.735	1.105	4.756	35.1	0.02491	39.2	0.00100	14.1	0.358	6.5	0.9	25.0	9.7	2654.6	613.1	6.5	0.9	0.2
21AR221_73	99.9	1295.948	0.614	10.087	24.7	0.32000	26.4	0.02639	4.0	0.153	167.9	6.7	281.9	65.1	1382.2	504.2	167.9	6.7	12.1
21AR221_74	997.3	9133.179	6.951	18.444	3.1	0.30531	6.8	0.04227	3.4	0.494	266.9	8.8	270.5	16.1	303.1	134.5	266.9	8.8	88.1
21AR221_75	627.2	5585.214	0.495	14.952	9.3	0.14567	11.0	0.01647	2.0	0.179	105.3	2.1	138.1	14.2	746.9	229.4	105.3	2.1	14.1
21AR221_76	298.3	6173.682	1.145	16.270	7.3	0.38950	7.9	0.04785	2.0	0.255	301.3	6.0	334.0	22.6	569.3	167.1	301.3	6.0	52.9
21AR221_77	550.8	15221.942	2.484	18.036	3.5	0.39760	4.7	0.05307	1.7	0.358	333.4	5.5	339.9	13.7	385.8	99.5	333.4	5.5	86.4
21AR221_78	158.9	9269.835	2.086	15.439	7.4	0.82132	8.6	0.09475	2.0	0.233	583.6	11.2	608.8	39.2	704.7	177.4	583.6	11.2	82.8
21AR221_79	76.3	898.761	2.757	9.933	15.9	0.63488	32.7	0.05447	3.6	0.109	341.9	11.9	499.1	129.2	1305.8	635.5	341.9	11.9	26.2
21AR221_80	168.3	5902.986	2.722	15.835	7.1	0.52151	10.9	0.06269	2.4	0.216	392.0	9.0	426.2	38.1	616.4	230.8	392.0	9.0	63.6
21AR221_81	142.1	2956.636	1.243	11.115	13.9	0.48319	17.7	0.04137	2.9	0.165	261.3	7.5	400.3	58.7	1309.5	340.3	261.3	7.5	20.0
21AR221_82	699.7	3130.409	1.834	15.149	10.2	0.08417	11.6	0.01001	2.4	0.209	64.2	1.6	82.1	9.2	640.4	244.8	64.2	1.6	10.0
21AR221_83	179.9	3955.702	1.308	14.650	10.5	0.38282	13.4	0.04323	2.0	0.146	272.8	5.2	329.1	37.7	749.8	280.3	272.8	5.2	36.4
21AR221_84	61.4	1273.952	1.463	9.031	31.1	0.67439	37.9	0.04925	3.7	0.098	309.9	11.3	523.4	155.4	1611.9	709.8	309.9	11.3	19.2
21AR221_85	123.1	2351.629	0.852	12.149	16.7	0.42381	20.2	0.04060	2.7	0.136	256.6	6.9	358.8	61.0	1088.2	401.6	256.6	6.9	23.6
21AR221_86	211.2	4466.984	0.537	15.702	10.0	0.33319	13.1	0.04028	2.6	0.195	254.6	6.4	292.0	33.2	604.3	277.4	254.6	6.4	42.1
21AR221_87	179.7	902.175	1.920	8.468	27.1	0.13366	39.2	0.00945	10.4	0.266	60.6	6.3	127.4	46.9	1672.2	705.2	60.6	6.3	3.6
21AR221_88	555.0	766.309	1.747	13.811	5.4	0.21089	10.5	0.02891	4.6	0.438	183.7	8.4	194.3	18.6	325.9	215.2	183.7	8.4	56.4
21AR221_89	222.3	16558.155	1.419	13.414	2.8	1.71515	3.2	0.17006	1.4	0.438	1012.5	13.0	1014.2	20.3	1018.8	57.5	1018.8	57.5	99.4
21AR221_90	674.6	52522.046	3.101	13.497	1.6	1.52526	4.1	0.15099	3.8	0.922	906.5	32.0	940.6	25.2	1022.1	32.2	1022.1	32.2	88.7
21AR221_91	451.4	37209.317	2.039	13.656	2.0	1.57419	3.7	0.15837	3.0	0.800	947.7	26.3	960.1	23.2	989.4	45.5	989.4	45.5	95.8
21AR221_92	1208.5	22762.824	10.550	15.576	1.6	0.75951	2.3	0.08757	1.4	0.595	541.1	7.0	573.7	10.0	706.0	38.8	541.1	7.0	76.6
21AR221_93	246.9	6433.586	1.741	14.850	9.1	0.33684	10.4	0.03792	2.0	0.194	239.9	4.8	294.8	26.6	756.5	215.6	239.9	4.8	31.7
21AR221_94	336.2	10689.437	2.093	16.826	5.2	0.49881	5.6	0.06295	1.6	0.280	393.5	5.9	410.9	18.8	510.6	117.3	393.5	5.9	77.1
21AR221_95	550.3	2997.713	1.048	12.109	15.7	0.10246	19.0	0.00966	3.3	0.171	62.0	2.0	99.0	17.9	1119.2	374.3	62.0	2.0	5.5
21AR221_96	421.3	2805.171	1.070	13.681	11.1	0.14090	12.7	0.01522	3.1	0.242	97.4	3.0	133.8	15.9	842.7	256.2	97.4	3.0	11.6
21AR221_97	160.3	5777.363	1.755	14.559	8.2	0.55561	11.1	0.06163	3.2	0.288	385.5	11.9	448.7	40.2	787.5	222.8	385.5	11.9	49.0
21AR221_98	394.5	11590.067	1.540	11.738	10.5	0.80211	14.7	0.06909	10.2	0.694	430.7	42.6	598.0	66.6	1298.1	206.1	430.7	42.6	33.2
21AR221_99	429.5	2970.045	2.532	13.131	14.0	0.11985	18.6	0.01235	3.3	0.177	79.1	2.6	114.9	20.2	940.2	376.3	79.1	2.6	8.4
21AR221_100	737.5	19343.427	0.915	18.128	3.3	0.44262	3.8	0.05975	1.5	0.407	374.1	5.6	372.1	11.9	360.6	78.4	374.1	5.6	103.8
21AR221_101	77.7	10596.422	2.004	11.201	4.0	2.85682	4.8	0.23854	1.7	0.343	1379.1	20.6	1370.6	36.5	1358.2	87.8	1358.2	87.8	101.5
21AR221_102	330.6	20889.141	37.293	16.172	4.1	0.86815	4.6	0.10429	1.7	0.369	639.5	10.4	634.6	21.8	618.0	92.8	639.5	10.4	103.5
21AR221_103	375.0	3433.486	0.950	16.462	5.4	0.46575	8.9	0.06054	1.8	0.197	378.9	6.5	388.2	28.8	445.3	194.8	378.9	6.5	85.1
21AR221_104	404.8	3756.174	1.937	16.186	9.2	0.14391	10.7	0.01827	2.3	0.214	116.7	2.6	136.5	13.6	497.3	229.9	116.7	2.6	23.5
21AR221_105	186.9	10692.479	6.813	16.371	6.5	0.70158	6.8	0.08627	1.6	0.239	533.5	8.4	539.7	28.6	567.3	144.6	533.5	8.4	94.0
21AR221_106	316.8	41045.301	1.642	9.676	1.4	3.95822	3.2	0.28220	2.8	0.902	1602.4	40.4	1625.7	25.6	1656.7	25.2	1656.7	25.2	96.7
21AR221_107	484.0	18187.728	1.608	18.247	3.9	0.43160	4.5	0.05870	2.0	0.453	367.7	7.2	364.3	13.7	343.6	90.0	367.7	7.2	107.0
21AR221_108	318.3	1966.990	1.608	16.431	6.0	0.29549	8.7	0.04024	2.3	0.262	254.3	5.7	262.9	20.1	340.6	189.9	254.3	5.7	74.7
21AR221_109	545.2	9242.490	1.437	17.444	3.8	0.46350	4.8	0.06083	2.1	0.435	380.6	7.6	386.7	15.3	424.0	95.5	380.6	7.6	89.8
21AR221_110	81.3	2280.403	2.301	10.603	22.4	0.67117	24.2	0.05569	3.8	0.159	349.3	13.1	521.4	98.7	1370.5	460.9	349.3	13.1	25.5
21AR221_111	214.8	8258.457	6.496	15.836	6.8	0.49368	8.5	0.05876	2.0	0.236	368.1	7.2	407.4	28.7	637.8	178.7	368.1	7.2	57.7
21AR221_112	280.7	5766.833	2.197	16.408	6.9	0.37471	8.7	0.04692	2.5	0.282	295.6	7.1	323.1	24.1	527.7	182.9	295.6	7.1	56.0
21AR221_113	452.6	11172.599	1.401	16.787	5.6	0.38347	6.9	0.04786	2.2	0.320	301.4	6.5	329.6	19.3	534.7	142.5	301.4	6.5	56.4
21AR221_114	284.7	6486.717	1.758	16.379	6.8	0.33104	8.7	0.04113	2.0	0.231	259.8	5.2	290.4	22.1	544.7	186.1	259.8	5.2	47.7
21AR221_115	127.8	2100.352	0.495	10.203	18.9	0.37757	20.3	0.03018	4.2	0.205	191.7	7.9	325.3	56.6	1441.9	380.3	191.7	7.9	13.3
21AR221_116	31.3	340.101	0.903	6.410	40.0	0.86762	78.4	0.05444	11.1	0.142	341.7	37.0	634.3	373.8	1890.0	1455.9	341.7	37.0	18.1
21AR221_117	198.0	5165.972	1.546	15.791	8.1	0.44992	9.2	0.05429	2.0	0.217	340.8	6.6	377.2	28.9	608.1	193.8	340.8	6.6	56.0
21AR221_118	202.0	17198.349	1.744	13.225	2.5	1.78679	3.2	0.17418	1.6	0.501	1035.1	15.6	1040.7	21.2	1053.3	56.7	1053.3	56.7	98.3
21AR221_119	236.5	16222.739	2.270	13.511	2.9	1.65221	3.4	0.16458	1.7	0.492	982.2	15.1	990.4	21.3	1009.5	59.3	1009.5	59.3	97.3
21AR221_120	88.0	2290.168	0.867	11.904	15.6	0.59330	17.3	0.05555	3.0	0.172	348.5	10.1	473.0	65.5	1134.1	340.3	348.5	10.1	30.7
21AR221_121	87.4	1266.427	1.767	11.592	14.7	0.76010	24.8	0.07379	2.9	0.117	459.0	12.8	574.1	108.6	1061.6	496.3	459.0	12.8	43.2
21AR221_122	226.3	10514.162	0.968	16.385	5.7	0.68105	6.9	0.08318	1.9	0.281	515.0	9.5	527.4	28.2	582.2	142.9	515.0	9.5	88.5
21AR221_123	654.7	2906.409	0.717	16.203	3.5	0.43298	6.5	0.05553	2.0	0.308	348.4	6.7	365.3	19.8	475.1	135.8	348.4	6.7	73.3
21AR221_124	557.0	39148.281	1.774	13.634	1.8	1.64037	2.6	0.16386	1.8	0.691	978.2	16.3	985.9	16.4	1003.8	38.1	1003.8	38.1	97.4
21AR221_125	326.2	18207.797	2.846	16.260	3.6	0.79623	4.8	0.09562	2.8	0.591	588.7	15.9	594.7	21.4	618.6	82.9	588.7	15.9	95.2
21AR221_126	42.0	541.308	0.996	6.470	44.5	0.49200	51.4	0.02756	11.1	0.215	175.3	19.1	406.3	172.6	2091.4	898.2	175.3	19.1	8.4
21AR221_127	176.7	5682.783	1.198	17.112	8.8	0.41692	11.7	0.05436	1.9	0.162	341.2	6.3	353.8	35.0	438.3	257.4	341.2	6.3	77.9
21AR221_128	316.4	4474.951	1.779	14.222	3.4	1.04955	8.8	0.11394	2.3	0.259	695.6	15.1	728.7	46.0	832.6	178.3	695.6	15.1	83.5
21AR221_129	291.2	3417.699	2.138	15.209	10.4	0.21031	13.3	0.02491	3.0	0.228	158.6	4.8	193.8	23.5	648.4	279.3	158.6	4.8	24.5
21AR221_130	696.8	33303.890	539.050	16.835	2.5	0.72342	3.6	0.08938	2.1	0.592	551.9	11.3	552.7	15.3	556.9	63.3	551.9	11.3	99.1
21AR221_131	332.4	78493.469	1.178	7.879	1.3	6.27038	2.2	0.35973	1.7	0.790	1980.9	29.2	2014.3	19.0	2049.5	23.5	2049.5	23.5	96.7
21AR221_132	29.5	3340.382	1.889	10.772	9.7	2.60596	10.6	0.21373	2.6	0.242	1248.7	29.1	1302.3	77.8	1392.6	197.3	1392.6	197.3	89.7
21AR221_133	183.3	4254.158	1.076	14.987	11.1	0.36484	12.4	0.04190	2.7	0.217	264.6	7.0	315.8	33.6	714.3	257.0	264.6	7.0	37.0
21AR221_134	163.2	5138.812	1.154	14.953	10.3	0.49280	13.4	0.05593	6.5	0.484	350.8	22.2	406.8	45.0	739.5	248.6	350.8	22.2	47.4
21AR221_135	1314.2	4064.790	3.213	16.603	2.4	0.43246	3.4	0.05551	2.3	0.665	348.3	7.7	364.9	10.4	473.1	56.2	348.3	7.7	73.6
21AR221_136	326.0	7805.842	2.761	16.470	6.0	0.35003	7.4	0.04320	1.9	0.257	272.6	5.1	304.7	19.5	559.3	156.1	272.6	5.1	48.7
21AR221_137	1716.6	10243.019	1.576	18.090	6.0	0.10609	6.7	0.01431	1.9	0.280	91.6	1.7	102.4	6.5	362.2	144.7	91.6	1.7	25.3
21AR221_138	388.8	6991.918	0.909	16.671	6.4	0.31109	9.0	0.03900	2.0	0.220	246.6	4.8	275.0	21.7	525.2	192.5	246.6	4.8	47.0
21AR221_139	687.4	15141.917	5.881	18.770	3.2	0.30865	3.9	0.04286	1.7	0.425	270.6	4.4	273.1	9.3	296.3	80.4	270.6	4.4	91.3
21AR221_140	330.7	30179.851	2.046	13.692	2.6	1.58157	3.3	0.15833	1.7	0.507	947.5	14.6	963.0	20.3	999.4	57.0	999.4	57.0	94.8
21AR221_141	120.7	3009.245	0.425	14.857	11.2	0.56934	15.3	0.06616	3.2	0.208	413.0	12.7	457.6	56.3	689.1	319.0	413.0	12.7	59.9
21AR221_142	280.0	11152.760	2.327	16.714	4.3	0.59916	5.8	0.07446	1.8	0.304	462.9	7.9	476.7	22.0	544.4	120.5	462.9	7.9	85.0
21AR221_143	208.0	2216.570	1.589	12.766	17.1	0.21926	20.4	0.02214	3.4	0.165	141.2	4.7	201.3	37.2	981.8	409.8	141.2	4.7	14.4
21AR221_144	1140.0	7365.206	1.833	18.405	4.8	0.11265	6.3	0.01564	1.5	0.247	100.0	1.5	108.4	6.5	297.1	138.8	100.0	1.5	33.7
21AR221_145	171.0	6353.394	0.665	14.202	11.2	0.55699	12.0	0.05938	2.5	0.205	371.9	8.9	449.6	43.8	870.4	244.6	371.9	8.9	42.7
21AR221_146	170.5	29782.642	1.956	10.990	2.5	2.85789	3.1	0.22952	1.7	0.551	1332.0	20.7	1370.9	23.4	1432.9	49.6	1432.9	49.6	93.0
21AR221_147	155.4	2995.974	0.902	13.829	12.5	0.35930	15.7	0.03868	3.2	0.206	244.6	7.8	311.7	42.2	850.4	320.5	244.6	7.8	28.8
21AR221_148	199.6	8327.967	1.687	15.851	5.9	0.72921	6.5	0.08641	1.9	0.285	534.2	9.6	556.1	28.0	647.5	134.8	534.2	9.6	82.5
21AR221_149	728.9	5210.904	2.346	16.914	3.6	0.59188	9.9	0.07639	1.4	0.145	474.5	6.5	472.1	37.2	461.1	216.4	474.5	6.5	102.9
21AR221_150	249.9	6538.590	0.943	17.373	8.2	0.31731	10.6	0.04174	1.9	0.183	263.6	5.0	279.8	26.0	418.8	233.4	263.6	5.0	62.9
21AR221_151	237.7	4985.544	1.388	15.505	9.6	0.34803	10.8	0.04113	2.3	0.214	259.9	5.9	303.2	28.3	652.9	226.3	259.9	5.9	39.8

21AR221_152	453.2	12100.463	2.554	17.879	5.9	0.29395	7.5	0.03912	1.8	0.241	247.4	4.4	261.7	17.3	392.6	163.3	247.4	4.4	63.0
21AR221_153	356.7	9221.067	1.480	17.279	5.7	0.32358	6.9	0.04181	1.8	0.269	264.1	4.8	284.7	17.0	458.2	146.4	264.1	4.8	57.6
21AR221_154	798.9	632.411	3.118	7.011	35.8	0.02270	39.7	0.00136	11.4	0.287	8.8	1.0	22.8	8.9	1973.0	684.0	8.8	1.0	0.4
21AR221_155	301.8	5629.007	1.223	16.403	8.4	0.31561	12.3	0.03933	2.1	0.168	248.6	5.0	278.5	30.0	538.5	265.8	248.6	5.0	46.2
21AR221_156	591.7	2956.226	1.284	12.300	16.3	0.09295	18.8	0.00884	3.1	0.166	56.7	1.8	90.2	16.3	1102.7	372.0	56.7	1.8	5.1
21AR221_157	276.5	13629.541	3.865	16.825	4.4	0.61409	5.4	0.07649	2.2	0.410	475.1	10.1	486.1	20.7	539.3	107.0	475.1	10.1	88.1
21AR221_158	237.2	4920.041	1.369	14.924	9.7	0.38407	10.4	0.04359	2.1	0.205	275.0	5.7	330.0	29.3	739.3	215.5	275.0	5.7	37.2
21AR221_159	318.0	2770.195	1.380	12.413	11.3	0.17817	12.1	0.01717	3.0	0.247	109.7	3.2	166.5	18.6	1076.9	235.4	109.7	3.2	10.2
21AR221_160	266.4	8043.287	3.506	15.654	6.9	0.51579	7.6	0.06037	2.5	0.326	377.9	9.1	422.3	26.4	674.0	154.3	377.9	9.1	56.1
21AR221_161	617.3	20048.623	1.330	17.643	3.6	0.45799	4.5	0.05947	1.5	0.336	372.4	5.5	382.9	14.5	447.4	95.2	372.4	5.5	83.2
21AR221_162	282.6	12201.026	2.256	17.007	4.3	0.58036	5.2	0.07320	1.6	0.305	455.4	7.0	464.7	19.5	511.8	109.3	455.4	7.0	89.0
21AR221_163	559.0	17719.505	0.893	18.360	4.1	0.35317	5.0	0.04778	1.3	0.270	300.8	3.9	307.1	13.1	355.8	107.7	300.8	3.9	84.5
21AR221_164	132.8	4734.339	0.693	15.484	8.0	0.60616	8.7	0.07149	1.8	0.208	445.1	7.7	481.1	33.2	657.5	181.6	445.1	7.7	67.7
21AR221_165	172.9	7839.593	0.863	16.276	6.5	0.58392	7.6	0.07110	1.8	0.241	442.8	7.9	467.0	28.6	588.6	160.7	442.8	7.9	75.2
21AR221_166	419.8	22267.230	2.134	16.214	3.1	0.79628	4.0	0.09484	1.8	0.464	584.1	10.2	594.7	17.8	636.5	75.3	584.1	10.2	91.8
21AR221_167	455.6	14163.585	2.296	17.711	3.5	0.46340	4.7	0.06076	1.5	0.323	380.2	5.6	386.6	15.2	425.9	99.5	380.2	5.6	89.3
21AR221_168	306.5	2718.996	0.223	15.574	6.7	0.32073	12.7	0.03961	2.2	0.176	250.4	5.5	282.5	31.4	557.8	273.3	250.4	5.5	44.9
21AR221_169	427.7	11392.580	1.746	16.427	6.0	0.33203	6.4	0.04049	1.5	0.226	255.9	3.6	291.1	16.2	585.5	135.7	255.9	3.6	43.7
21AR221_170	448.5	8239.775	2.650	16.501	8.0	0.22840	8.6	0.02821	1.8	0.205	179.4	3.1	208.9	16.3	557.5	184.7	179.4	3.1	32.2
21AR221_171	106.2	10196.927	3.021	13.433	5.6	1.76474	5.9	0.17625	1.6	0.273	1046.5	15.7	1032.6	38.5	1004.2	115.9	1004.2	115.9	104.2
21AR221_172	153.0	741.029	2.094	6.745	26.4	0.16038	29.2	0.00897	9.7	0.332	57.6	5.6	151.0	41.0	2094.0	486.2	57.6	5.6	2.7
21AR221_173	380.1	442.146	2.491	5.705	56.6	0.04576	62.6	0.00228	12.1	0.193	14.7	1.8	45.4	27.8	2296.2	1084.0	14.7	1.8	0.6
21AR221_174	564.6	34569.378	3.715	16.458	2.2	0.81944	3.0	0.09876	1.7	0.578	607.1	9.9	607.7	13.5	611.0	52.1	607.1	9.9	99.4
21AR221_175	43.4	905.159	0.588	6.234	44.6	0.55810	46.1	0.02777	11.0	0.238	176.6	19.1	450.3	168.1	2297.7	780.3	176.6	19.1	7.7
21AR221_176	210.1	8922.595	1.407	16.856	7.4	0.57714	8.2	0.07281	1.6	0.191	453.1	6.9	462.6	30.6	511.4	177.7	453.1	6.9	88.6
21AR221_177	114.5	1401.788	1.600	9.311	25.4	0.37288	28.4	0.02768	3.1	0.108	176.0	5.3	321.8	78.4	1581.7	531.3	176.0	5.3	11.1
21AR221_178	260.7	5332.690	2.768	15.382	10.6	0.30535	11.3	0.03548	3.2	0.281	224.8	7.0	270.6	26.8	689.1	231.5	224.8	7.0	32.6
21AR221_179	887.4	1167.924	25.081	14.122	2.5	0.35901	4.8	0.04453	3.6	0.745	280.9	9.9	311.5	13.0	548.5	70.6	280.9	9.9	51.2
21AR221_180	483.6	6242.199	1.776	15.216	8.9	0.16446	10.6	0.01878	2.3	0.213	119.9	2.7	154.6	15.3	726.5	220.5	119.9	2.7	16.5
21AR221_181	277.3	5770.566	0.670	15.433	9.6	0.33805	11.1	0.03950	2.8	0.253	249.7	6.9	295.7	28.5	677.3	230.3	249.7	6.9	36.9
21AR221_182	42.2	1150.514	2.274	8.915	27.3	0.64832	32.8	0.04710	8.7	0.264	296.7	25.1	507.4	131.1	1621.8	591.9	296.7	25.1	18.3
21AR221_183	891.5	16602.647	2.741	18.044	2.9	0.41904	4.5	0.05593	1.6	0.367	350.8	5.6	355.4	13.4	386.1	93.1	350.8	5.6	90.9
21AR221_184	423.4	10264.966	1.345	17.320	4.6	0.33060	5.4	0.04273	2.1	0.392	269.8	5.6	290.0	13.5	457.6	109.6	269.8	5.6	59.0
21AR221_185	896.5	69334.399	3.696	13.725	1.5	1.60341	2.6	0.16066	2.1	0.808	960.5	19.1	971.5	16.6	997.6	31.6	997.6	31.6	96.3
21AR221_186	326.0	30847.532	5.499	13.827	2.4	1.51580	3.5	0.15336	2.4	0.693	919.8	20.5	936.8	21.1	977.9	50.7	977.9	50.7	94.1
21AR221_187	222.8	10890.589	4.121	15.787	4.9	0.84794	6.5	0.09890	1.8	0.282	607.9	10.6	623.5	30.1	681.4	132.5	607.9	10.6	89.2
**21AR225**																			
21AR225_1	91.5	338.626	2.242	8.198	37.1	0.10822	71.4	0.00970	6.9	0.097	62.3	4.3	104.3	70.8	1219.4	1442.7	62.3	4.3	5.1
21AR225_2	130.8	409.788	2.781	8.399	23.6	0.11827	45.5	0.01004	7.1	0.155	64.4	4.5	113.5	48.8	1326.9	880.9	64.4	4.5	4.9
21AR225_3	270.1	4041.512	3.267	18.054	4.9	0.32613	6.5	0.04539	1.8	0.275	286.2	5.0	286.6	16.2	291.0	142.8	286.2	5.0	98.3
21AR225_4	211.9	5130.312	1.841	17.160	3.7	0.59057	7.2	0.07682	2.0	0.271	477.1	9.0	471.2	27.3	443.6	154.9	477.1	9.0	107.5
21AR225_5	145.8	3618.726	3.699	16.829	5.4	0.54489	11.5	0.07093	3.4	0.296	441.8	14.6	441.6	41.3	441.9	244.9	441.8	14.6	100.0
21AR225_6	61.9	1516.799	2.660	14.704	9.6	0.49632	12.1	0.06125	2.2	0.178	383.2	8.0	409.2	40.8	559.7	260.4	383.2	8.0	68.5
21AR225_7	186.1	9570.942	1.368	8.873	1.2	5.00981	2.3	0.32461	1.3	0.577	1812.2	20.7	1821.0	19.2	1831.8	33.7	1831.8	33.7	98.9
21AR225_8	379.7	8300.036	2.136	17.107	2.1	0.66693	3.9	0.08504	1.7	0.427	526.1	8.3	518.8	15.7	487.7	77.1	526.1	8.3	107.9
21AR225_9	284.5	10844.555	2.465	13.557	1.6	1.71214	2.7	0.17095	1.6	0.606	1017.3	15.4	1013.1	17.3	1004.8	43.4	1004.8	43.4	101.2
21AR225_10	109.9	1045.563	1.069	14.161	11.9	0.23415	42.0	0.02988	2.9	0.068	189.8	5.4	213.6	80.9	485.7	933.2	189.8	5.4	39.1
21AR225_11	61.7	609.035	0.560	10.436	18.0	0.28822	62.4	0.02873	3.5	0.056	182.6	6.3	257.2	141.9	1008.1	1293.6	182.6	6.3	18.1
21AR225_12	127.0	2092.178	2.284	15.068	11.4	0.36918	21.8	0.04493	2.0	0.090	283.3	5.5	319.0	59.7	590.0	471.7	283.3	5.5	48.0
21AR225_13	244.9	3925.112	1.706	17.979	4.1	0.43634	6.9	0.06065	1.7	0.242	379.6	6.2	367.7	21.4	294.4	153.8	379.6	6.2	128.9
21AR225_14	261.6	4628.306	4.112	17.626	4.5	0.45199	6.0	0.06084	1.8	0.298	380.8	6.6	378.7	19.0	366.9	129.2	380.8	6.6	103.8
21AR225_15	257.1	7113.109	4.104	16.861	3.5	0.69571	4.7	0.08762	1.6	0.341	541.5	8.3	536.2	19.5	515.0	96.7	541.5	8.3	105.1
21AR225_16	94.4	3877.205	4.080	14.944	5.1	1.01758	8.2	0.11163	1.8	0.219	708.2	12.1	712.7	42.1	727.7	170.0	708.2	12.1	97.3
21AR225_17	114.4	1163.282	1.265	16.271	7.4	0.27177	35.3	0.04015	1.9	0.054	253.8	4.7	244.1	76.7	153.1		253.8	4.7	165.8
21AR225_18	110.8	1230.366	1.605	14.721	11.3	0.32044	15.0	0.04121	2.0	0.135	260.3	5.2	282.2	37.0	468.9	330.0	260.3	5.2	55.5
21AR225_19	328.2	5466.560	0.966	18.173	4.6	0.29766	7.2	0.04101	1.6	0.216	259.1	4.0	264.6	16.8	314.3	159.9	259.1	4.0	82.4
21AR225_20	164.1	3635.876	1.570	17.066	5.9	0.45586	11.4	0.06023	1.5	0.129	377.0	5.4	381.4	36.2	408.7	252.6	377.0	5.4	92.3
21AR225_21	260.6	3400.308	3.278	17.191	5.8	0.31000	12.1	0.04133	1.6	0.129	261.1	4.0	274.2	29.1	388.4	269.6	261.1	4.0	67.2
21AR225_22	263.0	4511.239	2.595	18.519	4.2	0.41083	5.5	0.05855	1.6	0.282	366.8	5.6	349.5	16.4	237.0	122.5	366.8	5.6	154.8
21AR225_23	228.4	3235.452	2.085	17.240	5.6	0.36859	17.6	0.04984	1.8	0.101	313.5	5.4	318.6	48.2	356.9	396.2	313.5	5.4	87.9
21AR225_24	270.8	13414.338	4.396	13.193	1.8	1.48634	3.5	0.14382	2.5	0.713	866.2	20.1	924.8	21.1	1068.1	49.0	1068.1	49.0	81.1
21AR225_25	312.5	2427.473	2.502	12.054	1.3	2.14736	4.3	0.20134	1.1	0.265	1182.5	12.2	1164.2	29.4	1131.1	81.6	1131.1	81.6	104.5
21AR225_26	615.1	15157.048	130.170	18.184	1.8	0.49458	2.9	0.06620	1.1	0.388	413.2	4.4	408.0	9.6	379.8	59.3	413.2	4.4	108.8
21AR225_27	139.7	3991.044	6.628	15.704	4.3	0.82944	6.2	0.09974	1.6	0.257	612.9	9.3	613.3	28.7	615.7	129.9	612.9	9.3	99.5
21AR225_28	492.3	7848.422	23.602	16.415	2.1	0.72140	3.7	0.08818	1.9	0.510	544.7	9.8	551.5	15.7	580.4	68.9	544.7	9.8	93.9
21AR225_29	673.9	3905.518	2.159	17.929	4.5	0.13744	7.2	0.01908	1.6	0.226	121.8	2.0	130.8	8.8	297.3	160.2	121.8	2.0	41.0
21AR225_30	270.5	4217.991	5.678	17.816	3.3	0.42092	8.4	0.05771	1.3	0.156	361.7	4.6	356.7	25.3	325.3	188.4	361.7	4.6	111.2
21AR225_31	135.7	930.196	1.543	15.207	7.8	0.32658	36.6	0.04694	2.1	0.058	295.7	6.1	286.9	91.6	217.4		295.7	6.1	136.0
21AR225_32	642.0	10887.542	4.100	18.240	2.0	0.44826	3.1	0.06052	1.3	0.412	378.8	4.6	376.1	9.6	360.3	62.7	378.8	4.6	105.1
21AR225_33	307.5	6398.922	2.939	17.414	2.7	0.60804	5.0	0.07961	1.4	0.278	493.8	6.6	482.3	19.0	429.1	106.1	493.8	6.6	115.1
21AR225_34	168.2	1914.474	1.110	16.243	8.3	0.30193	16.2	0.04031	1.6	0.101	254.8	4.1	267.9	38.3	385.4	363.6	254.8	4.1	66.1
21AR225_35	814.3	17567.406	4.143	18.217	1.8	0.45101	3.5	0.05997	1.2	0.338	375.4	4.3	378.0	11.0	394.6	73.6	375.4	4.3	95.1
21AR225_36	415.6	6613.711	2.101	18.361	3.0	0.29778	5.9	0.04097	1.4	0.232	258.8	3.4	264.7	13.6	317.6	129.5	258.8	3.4	81.5
21AR225_37	570.4	12439.845	3.112	16.902	1.6	0.69260	2.7	0.08584	1.1	0.418	530.9	5.7	534.4	11.2	550.1	53.5	530.9	5.7	96.5
21AR225_38	346.9	1118.819	2.155	16.950	3.8	0.25088	29.6	0.03944	2.1	0.070	249.4	5.0	227.3	60.3	5.6		249.4	5.0	4467.7
21AR225_39	215.8	3023.074	1.378	16.226	7.4	0.33826	13.1	0.04280	1.2	0.092	270.2	3.2	295.9	33.5	504.9	286.5	270.2	3.2	53.5
21AR225_40	474.3	15682.429	11.514	15.718	1.7	0.99368	2.3	0.11392	1.2	0.521	695.5	8.0	700.6	11.8	718.0	42.3	695.5	8.0	96.9
21AR225_41	336.9	3152.335	0.961	18.064	4.2	0.29026	11.2	0.04112	1.3	0.120	259.7	3.4	258.8	25.5	250.9	255.6	259.7	3.4	103.5
21AR225_42	618.8	4968.988	32.511	13.411	1.3	1.66270	2.1	0.16642	1.3	0.630	992.4	12.1	994.4	13.3	999.7	33.0	999.7	33.0	99.3
21AR225_43	129.1	384.833	2.291	8.530	25.3	0.11618	64.5	0.01036	6.2	0.096	66.5	4.1	111.6	68.2	1230.0	1293.6	66.5	4.1	5.4
21AR225_44	156.4	2928.256	2.222	17.098	5.6	0.48576	11.5	0.06516	1.3	0.110	406.9	5.0	402.0	38.0	374.8	256.5	406.9	5.0	108.6
21AR225_45	82.2	4976.910	78.781	13.338	4.1	1.80170	5.1	0.17930	1.4	0.272	1063.2	13.5	1046.1	33.2	1011.4	99.1	1011.4	99.1	105.1
21AR225_46	68.7	5045.397	2.612	10.566	2.2	3.24729	3.8	0.25361	1.4	0.369	1457.0	18.3	1468.5	29.5	1486.0	66.9	1486.0	66.9	98.1
21AR225_47	165.5	5237.394	1.407	16.113	3.7	0.82678	7.0	0.10006	1.4	0.196	614.8	8.1	611.8	32.3	601.8	149.2	614.8	8.1	102.1
21AR225_48	318.1	4316.430	1.167	18.045	4.1	0.28094	5.8	0.03882	1.5	0.259	245.5	3.6	251.4	13.0	307.7	128.4	245.5	3.6	79.8
21AR225_49	572.3	546.968	1.539	15.923	3.4	0.19076	15.0	0.03867	3.2	0.210	244.6	7.6	177.3	24.4	NA		244.6	7.6	
21AR225_50	123.7	2079.599	2.928	15.215	9.9	0.39744	16.3	0.04864	2.1	0.126	306.1	6.2	339.8	47.2	577.8	352.6	306.1	6.2	53.0
21AR225_51	69.8	5541.080	5.619	10.933	2.9	3.02576	4.4	0.24452	1.3	0.288	1410.1	15.9	1414.1	33.2	1421.0	79.6	1421.0	79.6	99.2
21AR225_52	160.7	9625.501	3.770	12.870	2.2	2.07621	3.7	0.19562	1.6	0.432	1151.7	16.9	1141.0	25.4	1121.5	66.7	1121.5	66.7	102.7
21AR225_53	42.7	932.234	0.282	12.370	15.3	0.58112	36.3	0.06377	2.7	0.074	398.5	10.4	465.2	135.7	810.3	763.4	398.5	10.4	49.2
21AR225_54	386.0	7475.426	7.565	18.454	3.1	0.42448	4.1	0.05861	1.3	0.321	367.1	4.7	359.2	12.5	309.5	88.9	367.1	4.7	118.6
21AR225_55	196.5	4255.487	1.804	17.894	3.9	0.54102	9.6	0.07439	1.3	0.140	462.5	6.0	439.1	34.4	319.0	217.0	462.5	6.0	145.0
21AR225_56	182.0	3666.692	21.455	16.874	5.1	0.50072	8.1	0.06531	1.4	0.169	407.9	5.4	412.2	27.3	437.4	176.9	407.9	5.4	93.2
21AR225_57	227.1	1801.424	1.593	17.716	4.1	0.43252	26.8	0.06460	1.9	0.072	403.5	7.5	365.0	82.3	127.8		403.5	7.5	315.8
21AR225_58	101.6	625.059	2.242	11.241	22.7	0.17508	43.4	0.01916	3.3	0.076	122.3	4.0	163.8	65.7	816.0	915.4	122.3	4.0	15.0
21AR225_59	528.5	6499.899	2.118	18.815	3.2	0.28656	5.3	0.04055	1.7	0.315	256.3	4.2	255.9	12.1	253.0	116.7	256.3	4.2	101.3
21AR225_60	60.8	4750.649	2.478	12.786	5.8	1.99281	7.7	0.19114	1.6	0.204	1127.6	16.2	1113.1	52.0	1085.8	150.8	1085.8	150.8	103.8
21AR225_61	543.8	2159.171	2.648	17.926	3.0	0.38426	5.1	0.05660	2.0	0.383	354.9	6.7	330.2	14.4	160.0	110.2	354.9	6.7	221.8
21AR225_62	139.3	1073.900	1.370	14.692	10.2	0.28067	23.5	0.03719	2.5	0.107	235.4	5.8	251.2	52.2	402.6	524.0	235.4	5.8	58.5
21AR225_63	308.9	715.321	11.725	15.755	5.9	0.45080	92.7	0.07567	1.7	0.018	470.2	7.6	377.8	294.4	NA		470.2	7.6	
21AR225_64	92.1	1016.026	1.085	14.069	15.1	0.31265	23.2	0.03975	2.6	0.112	251.3	6.4	276.2	56.2	494.0	510.4	251.3	6.4	50.9
21AR225_65	221.4	4235.757	2.361	16.615	5.3	0.34906	9.0	0.04457	1.7	0.186	281.1	4.6	304.0	23.8	484.6	196.2	281.1	4.6	58.0
21AR225_66	125.2	2468.791	0.586	17.001	5.9	0.45675	10.3	0.06258	1.6	0.157	391.3	6.1	382.0	32.8	327.1	230.7	391.3	6.1	119.6
21AR225_67	527.7	10660.250	2.361	17.692	1.9	0.55954	3.8	0.07352	1.4	0.355	457.3	6.0	451.2	13.9	421.1	79.7	457.3	6.0	108.6
21AR225_68	90.9	247.478	1.820	6.952	28.7	0.11521	87.8	0.00958	7.8	0.089	61.5	4.8	110.7	92.2	1366.3	1773.5	61.5	4.8	4.5
21AR225_69	124.8	3301.632	3.665	16.823	5.7	0.56960	16.1	0.07502	1.5	0.091	466.3	6.6	457.8	59.3	416.0	358.6	466.3	6.6	112.1
21AR225_70	657.1	32823.845	5.671	13.396	1.4	1.70512	2.0	0.16604	1.4	0.696	990.3	12.6	1010.5	12.6	1055.4	28.5	1055.4	28.5	93.8
21AR225_71	142.1	4248.021	4.629	15.766	4.3	0.84440	5.6	0.10179	1.3	0.238	624.9	8.0	621.6	26.1	610.3	117.9	624.9	8.0	102.4
21AR225_72	208.5	789.606	0.929	10.296	13.8	0.13535	29.0	0.01236	3.2	0.111	79.2	2.5	128.9	35.2	1184.4	573.3	79.2	2.5	6.7
21AR225_73	311.7	4283.431	5.893	17.998	4.3	0.30198	11.5	0.04159	1.3	0.117	262.7	3.5	267.9	27.1	314.9	259.7	262.7	3.5	83.4
21AR225_74	196.8	3866.702	2.378	17.077	5.0	0.46857	8.9	0.06189	1.5	0.165	387.1	5.5	390.2	28.7	409.7	195.9	387.1	5.5	94.5
21AR225_75	190.4	2520.448	1.189	17.273	4.1	0.59447	11.9	0.08259	1.6	0.134	511.5	7.8	473.7	44.9	295.5	268.6	511.5	7.8	173.1
21AR225_76	420.8	10847.572	2.492	16.076	2.0	0.87436	3.3	0.10395	2.0	0.623	637.5	12.3	637.9	15.4	640.4	54.8	637.5	12.3	99.6
21AR225_77	164.1	1011.894	2.098	15.727	6.1	0.29685	45.1	0.04371	1.9	0.042	275.8	5.1	263.9	105.0	161.3		275.8	5.1	171.0
21AR225_78	331.7	3037.704	0.992	18.089	3.7	0.24405	12.9	0.03501	1.6	0.126	221.8	3.5	221.7	25.7	221.7	296.1	221.8	3.5	100.0

21AR225_79	187.0	398.821	2.157	9.849	19.8	0.07281	45.4	0.00806	4.6	0.102	51.8	2.4	71.4	31.3	790.7	959.1	51.8	2.4	6.5
21AR225_80	221.0	7755.306	2.151	17.210	3.0	0.66855	7.0	0.08605	1.2	0.166	532.1	5.9	519.8	28.3	467.2	151.9	532.1	5.9	113.9
21AR225_81	249.0	8811.302	13.054	17.158	2.9	0.65032	3.6	0.08287	1.3	0.367	513.2	6.5	508.7	14.4	489.1	73.8	513.2	6.5	104.9
21AR225_82	278.5	1436.336	0.647	16.431	6.4	0.28329	28.9	0.04052	1.4	0.049	256.0	3.6	253.3	64.8	228.6		256.0	3.6	112.0
21AR225_83	483.2	9999.883	2.133	18.188	2.6	0.45012	5.7	0.06095	1.4	0.239	381.4	5.1	377.4	18.1	353.8	125.8	381.4	5.1	107.8
21AR225_84	315.5	4112.167	10.041	17.682	4.3	0.31415	7.4	0.04296	1.5	0.197	271.1	3.9	277.4	18.0	331.5	164.6	271.1	3.9	81.8
21AR225_85	621.2	5830.839	2.134	17.909	2.2	0.44971	5.1	0.06111	1.3	0.265	382.4	5.0	377.1	16.0	345.8	110.5	382.4	5.0	110.6
21AR225_86	236.7	5797.093	3.034	17.508	3.6	0.55681	5.4	0.07390	1.3	0.236	459.6	5.7	449.4	19.6	398.8	117.7	459.6	5.7	115.3
21AR225_87	791.7	3241.446	1.971	15.844	1.7	0.60108	2.5	0.07428	1.5	0.571	461.9	6.5	477.9	9.7	556.5	45.5	461.9	6.5	83.0
21AR225_88	87.2	819.654	0.722	12.451	12.8	0.34711	25.4	0.04010	3.4	0.135	253.5	8.6	302.5	66.6	701.6	538.6	253.5	8.6	36.1
21AR225_89	140.7	6792.195	2.260	12.095	2.2	2.29562	5.0	0.20612	1.2	0.244	1208.1	13.4	1210.9	35.2	1216.8	94.9	1216.8	94.9	99.3
21AR225_90	529.3	10627.768	4.539	17.512	1.6	0.57685	2.8	0.07490	1.0	0.339	465.6	4.3	462.4	10.4	447.6	58.7	465.6	4.3	104.0
21AR225_91	337.7	5689.741	3.679	18.116	3.2	0.43208	4.2	0.05942	1.4	0.340	372.1	5.1	364.7	12.8	318.7	89.2	372.1	5.1	116.7
21AR225_92	336.0	21616.313	4.124	13.686	1.4	1.71751	2.2	0.17092	1.3	0.597	1017.2	12.6	1015.1	14.4	1011.4	36.6	1011.4	36.6	100.6
21AR225_93	70.2	741.285	0.680	10.200	18.4	0.31014	39.7	0.02846	3.6	0.091	180.9	6.4	274.3	95.6	1173.8	790.7	180.9	6.4	15.4
21AR225_94	103.1	1434.834	1.191	13.441	12.9	0.36933	18.4	0.04159	1.7	0.093	262.7	4.4	319.2	50.5	755.7	388.1	262.7	4.4	34.8
21AR225_95	214.6	3755.184	2.944	18.005	4.2	0.44403	6.3	0.06231	1.5	0.241	389.7	5.8	373.1	19.8	272.4	141.0	389.7	5.8	143.0
21AR225_96	278.5	18680.301	3.557	12.284	1.7	2.19870	2.4	0.19756	1.4	0.567	1162.2	14.6	1180.6	16.9	1215.4	39.3	1215.4	39.3	95.6
21AR225_97	1551.1	6698.614	2.071	18.679	1.7	0.33889	5.2	0.04783	1.4	0.268	301.2	4.1	296.3	13.4	259.4	115.3	301.2	4.1	116.1
21AR225_98	588.9	11567.582	4.858	18.340	2.3	0.46710	4.0	0.06357	1.4	0.338	397.3	5.2	389.2	13.0	342.0	85.9	397.3	5.2	116.2
21AR225_99	464.2	5511.754	2.009	18.089	4.2	0.29300	9.8	0.04037	1.4	0.142	255.1	3.5	260.9	22.5	314.2	220.6	255.1	3.5	81.2
21AR225_100	234.3	2880.190	3.994	17.818	5.3	0.30880	14.6	0.04388	1.4	0.097	276.8	3.8	273.3	34.9	243.7	334.1	276.8	3.8	113.6
21AR225_101	294.7	3957.172	1.749	17.473	4.6	0.33106	8.0	0.04456	1.3	0.166	281.0	3.6	290.4	20.1	367.2	177.2	281.0	3.6	76.5
21AR225_102	205.2	3138.267	2.970	17.612	5.6	0.35969	14.8	0.05007	1.4	0.094	314.9	4.3	312.0	39.7	290.9	336.1	314.9	4.3	108.3
21AR225_103	42.4	87.125	1.465	5.806	38.4	-0.01798	3625.7	0.01412	11.4	0.003	90.4	10.2	NA	-700.5	NA		90.4	10.2	
21AR225_104	67.4	333.845	1.381	11.165	20.4	0.17127	104.8	0.02738	4.4	0.042	174.2	7.6	160.5	155.9	NA		174.2	7.6	
21AR225_105	524.8	4045.221	2.475	17.369	4.1	0.21346	11.6	0.02864	1.4	0.124	182.0	2.6	196.5	20.7	374.3	259.4	182.0	2.6	48.6
21AR225_106	157.3	301.878	4.516	10.940	12.4	0.40123	224.0	0.06857	6.3	0.028	427.5	26.1	342.5	675.2	NA		427.5	26.1	
21AR225_107	341.1	860.324	1.102	10.794	14.3	0.07685	36.1	0.00730	3.3	0.092	46.9	1.6	75.2	26.2	1104.0	724.7	46.9	1.6	4.2
21AR225_108	19.7	325.062	1.237	6.641	20.3	0.74496	36.9	0.04961	6.9	0.188	312.1	21.2	565.3	160.5	1782.0	667.2	312.1	21.2	17.5
21AR225_109	541.1	8683.029	94.852	17.954	3.3	0.38671	7.5	0.05183	3.5	0.469	325.7	11.2	332.0	21.2	376.9	149.1	325.7	11.2	86.4
21AR225_110	296.0	14917.181	4.411	13.531	1.5	1.59377	3.5	0.15816	1.7	0.500	946.5	15.3	967.8	21.8	1017.2	61.2	1017.2	61.2	93.0
21AR225_111	379.0	8536.551	2.638	18.346	2.5	0.45792	3.4	0.06291	1.3	0.386	393.3	5.1	382.8	11.0	320.9	72.1	393.3	5.1	122.6
21AR225_112	829.3	984.928	1.144	17.008	2.7	0.16044	6.0	0.02655	1.4	0.235	168.9	2.3	151.1	8.4	NA		168.9	2.3	
21AR225_113	166.4	2908.230	1.835	17.736	4.8	0.45495	8.9	0.06414	1.3	0.141	400.8	4.9	380.7	28.4	261.8	203.3	400.8	4.9	153.1
21AR225_114	371.9	4195.131	1.822	18.367	3.3	0.30672	7.7	0.04358	1.2	0.158	275.0	3.3	271.6	18.3	243.8	174.9	275.0	3.3	112.8
21AR225_115	157.7	3450.275	1.619	17.279	5.2	0.55683	9.1	0.07514	1.4	0.159	467.1	6.5	449.5	32.9	361.3	202.1	467.1	6.5	129.3
21AR225_116	299.5	6177.937	2.187	17.653	4.2	0.45847	7.7	0.06110	1.5	0.190	382.3	5.4	383.2	24.6	389.6	169.9	382.3	5.4	98.1
21AR225_117	580.5	13174.363	2.463	18.226	2.7	0.45556	3.4	0.06125	1.4	0.398	383.2	5.0	381.2	10.8	369.7	70.4	383.2	5.0	103.7
21AR225_118	1249.0	1378.677	1.163	18.369	2.4	0.16113	8.8	0.02665	1.9	0.218	169.6	3.2	151.7	12.4	NA		169.6	3.2	
21AR225_119	78.2	732.554	1.192	12.797	13.0	0.31294	28.7	0.03880	3.0	0.106	245.4	7.3	276.5	69.5	549.5	626.6	245.4	7.3	44.7
21AR225_120	150.8	3234.371	4.705	16.760	4.3	0.66302	7.9	0.08661	1.5	0.188	535.5	7.6	516.5	31.8	434.1	171.8	535.5	7.6	123.3
21AR225_121	209.4	2657.863	2.186	16.846	4.8	0.33940	13.1	0.04554	1.5	0.113	287.1	4.2	296.7	33.7	374.1	293.4	287.1	4.2	76.7
21AR225_122	168.5	1787.043	0.980	15.365	8.1	0.32115	15.1	0.04077	1.7	0.112	257.6	4.2	282.8	37.2	497.6	330.1	257.6	4.2	51.8
21AR225_123	253.5	1216.212	0.924	13.945	11.0	0.12660	32.4	0.01529	2.3	0.072	97.8	2.3	121.0	37.0	607.0	703.6	97.8	2.3	16.1
21AR225_124	205.6	2609.656	1.971	17.054	5.3	0.32532	14.8	0.04418	1.5	0.103	278.7	4.2	286.0	36.9	347.0	333.6	278.7	4.2	80.3
21AR225_125	36.2	736.860	2.477	11.303	17.9	0.54164	38.9	0.05651	3.1	0.080	354.4	10.7	439.5	138.9	914.8	804.4	354.4	10.7	38.7
21AR225_126	212.3	2078.501	5.277	15.920	4.6	0.70308	12.9	0.09073	2.3	0.181	559.8	12.5	540.6	53.9	461.4	280.6	559.8	12.5	121.3
21AR225_127	558.5	2079.723	13.231	16.926	2.4	0.50649	5.2	0.07009	1.2	0.225	436.7	5.0	416.1	17.8	304.3	116.0	436.7	5.0	143.5
21AR225_128	32.7	463.194	0.421	8.593	31.1	0.44633	45.1	0.03741	6.1	0.136	236.8	14.2	374.7	141.5	1350.9	872.6	236.8	14.2	17.5
21AR225_129	55.6	430.301	1.510	8.624	26.4	0.31566	72.2	0.02725	4.5	0.062	173.3	7.7	278.6	176.5	1293.5	1450.9	173.3	7.7	13.4
21AR225_130	201.1	1501.115	0.808	16.794	6.1	0.21165	15.8	0.03062	1.6	0.099	194.4	3.0	194.9	28.0	202.0	364.6	194.4	3.0	96.2
21AR225_131	49.8	954.081	1.952	11.314	14.1	0.51574	23.9	0.05043	3.5	0.147	317.1	10.9	422.3	82.7	1047.2	478.8	317.1	10.9	30.3
21AR225_132	471.6	21294.451	2.751	13.531	1.3	1.72839	1.8	0.16947	1.2	0.664	1009.2	11.0	1019.2	11.4	1041.4	26.9	1041.4	26.9	96.9
21AR225_133	539.7	6040.346	1.856	18.854	3.5	0.29189	6.4	0.04142	1.5	0.234	261.6	3.8	260.0	14.6	247.0	142.8	261.6	3.8	105.9
21AR225_134	453.4	2431.587	1.134	18.366	3.6	0.26389	13.1	0.03916	1.4	0.104	247.6	3.3	237.8	27.8	143.1		247.6	3.3	173.1
21AR225_135	524.4	8878.772	1.690	18.377	3.0	0.44883	4.4	0.06110	1.5	0.333	382.3	5.5	376.5	13.9	341.5	94.2	382.3	5.5	111.9
21AR225_136	186.2	5069.375	3.282	7.883	1.4	5.26785	3.8	0.30560	1.4	0.376	1719.0	21.8	1863.7	32.8	2029.8	63.0	2029.8	63.0	84.7
21AR225_137	839.4	517.625	0.946	16.332	2.5	0.13408	10.7	0.03001	2.3	0.219	190.6	4.4	127.8	12.8	NA		190.6	4.4	
21AR225_138	415.9	10481.408	15.035	17.284	2.3	0.63257	4.7	0.08073	1.2	0.245	500.5	5.6	497.7	18.7	485.9	101.6	500.5	5.6	103.0
21AR225_139	225.5	4537.661	3.913	17.501	4.4	0.53823	8.6	0.07183	2.6	0.305	447.2	11.3	437.3	30.5	386.2	183.4	447.2	11.3	115.8
21AR225_140	388.7	5500.282	2.488	17.832	3.4	0.45397	4.7	0.06118	1.1	0.245	382.8	4.3	380.1	14.9	364.2	102.8	382.8	4.3	105.1
21AR225_141	66.7	4326.113	2.591	12.887	5.5	1.93686	6.9	0.18799	1.3	0.180	1110.5	12.8	1093.9	46.4	1062.0	137.2	1062.0	137.2	104.6
21AR225_142	616.6	1283.216	2.434	16.304	2.4	0.53834	17.4	0.07780	1.5	0.086	483.0	7.0	437.3	61.7	204.5	402.0	483.0	7.0	236.2
21AR225_143	88.1	624.322	0.791	10.535	15.5	0.31812	55.2	0.03185	2.8	0.051	202.1	5.6	280.5	135.4	999.0	1140.2	202.1	5.6	20.2
21AR225_144	113.3	2272.058	1.634	16.951	5.7	0.54651	11.6	0.07500	2.0	0.168	466.2	8.8	442.7	41.7	323.4	260.3	466.2	8.8	144.2
21AR225_145	892.1	7086.359	10.265	12.124	1.5	2.20635	2.2	0.19771	1.6	0.716	1163.0	16.7	1183.1	15.3	1220.8	30.2	1220.8	30.2	95.3
21AR225_146	184.7	661.752	1.985	11.303	19.0	0.11649	39.9	0.01262	5.0	0.124	80.9	4.0	111.9	42.3	836.4	832.6	80.9	4.0	9.7
21AR225_147	159.9	660.194	2.643	11.272	20.3	0.17794	39.3	0.01930	3.0	0.078	123.2	3.7	166.3	60.3	835.1	823.2	123.2	3.7	14.8
21AR225_148	346.9	8276.239	4.030	12.776	1.5	1.93093	3.1	0.18299	1.9	0.598	1083.3	18.8	1091.9	21.0	1109.9	50.3	1109.9	50.3	97.6
21AR225_149	171.1	14924.601	2.543	9.992	1.4	3.60575	2.5	0.26383	1.4	0.543	1509.4	18.3	1550.8	19.9	1608.4	39.2	1608.4	39.2	93.8
21AR225_150	54.6	318.899	1.760	8.677	19.8	0.22804	45.7	0.02334	7.1	0.156	148.7	10.5	208.6	86.1	954.5	933.5	148.7	10.5	15.6
21AR225_151	76.1	2512.829	0.824	9.432	1.9	3.77345	7.7	0.27227	1.3	0.167	1552.3	17.7	1587.1	61.7	1634.5	140.8	1634.5	140.8	95.0
21AR225_152	132.7	2956.263	1.176	17.361	5.5	0.45633	15.9	0.06288	1.7	0.110	393.1	6.6	381.7	50.5	314.1	359.2	393.1	6.6	125.2
21AR225_153	206.3	6162.126	1.857	16.614	3.9	0.66524	5.1	0.08347	1.5	0.293	516.8	7.4	517.8	20.7	523.2	107.3	516.8	7.4	98.8
21AR225_154	63.9	899.149	0.894	11.964	18.0	0.36958	36.2	0.03970	2.9	0.080	251.0	7.1	319.3	99.3	854.5	755.4	251.0	7.1	29.4
21AR225_155	133.1	2253.758	2.543	16.503	7.2	0.35650	23.6	0.04785	1.7	0.070	301.3	4.9	309.6	62.9	373.6	530.8	301.3	4.9	80.7
21AR225_156	937.8	22527.852	29.896	17.867	1.4	0.53198	2.9	0.06984	1.1	0.390	435.2	4.7	433.1	10.1	423.1	59.1	435.2	4.7	102.9
21AR225_157	144.7	2377.793	2.036	16.767	5.6	0.44753	14.7	0.06086	1.5	0.100	380.9	5.4	375.5	46.2	343.8	331.9	380.9	5.4	110.8
21AR225_158	147.2	922.147	1.043	14.762	8.9	0.27467	60.4	0.03847	2.0	0.034	243.4	4.8	246.4	132.4	276.6		243.4	4.8	88.0
21AR225_159	624.9	7149.196	2.313	19.758	3.4	0.27126	6.9	0.04058	2.4	0.345	256.4	6.0	243.7	15.0	124.0	152.8	256.4	6.0	206.8
21AR225_160	591.4	2397.001	10.920	15.360	2.9	0.63077	4.3	0.07753	2.5	0.575	481.3	11.4	496.6	16.8	568.4	76.4	481.3	11.4	84.7
21AR225_161	69.8	959.895	0.743	12.379	17.3	0.36434	47.0	0.04020	3.1	0.067	254.1	7.8	315.4	127.7	798.7	996.7	254.1	7.8	31.8
21AR225_162	48.6	1879.750	3.664	13.615	9.1	0.96335	14.6	0.10626	2.1	0.145	651.0	13.2	685.0	72.9	799.4	303.7	651.0	13.2	81.4
21AR225_163	135.4	2016.989	1.933	16.368	5.9	0.47252	14.9	0.06377	2.0	0.131	398.5	7.5	392.9	48.5	361.0	333.5	398.5	7.5	110.4
21AR225_164	142.3	2644.510	5.431	16.622	7.8	0.44041	14.8	0.05845	1.7	0.112	366.2	5.9	370.5	45.9	398.6	329.7	366.2	5.9	91.9
21AR225_165	343.6	9627.219	1.527	17.483	3.4	0.58636	4.7	0.07637	1.6	0.339	474.4	7.3	468.5	17.7	440.8	98.7	474.4	7.3	107.6
21AR225_166	221.2	2035.571	1.713	17.807	7.8	0.28603	13.7	0.04239	1.8	0.131	267.7	4.7	255.4	31.0	145.7		267.7	4.7	183.7
21AR225_167	673.0	2634.851	2.123	15.041	1.5	0.75129	4.3	0.08877	2.9	0.674	548.2	15.2	569.0	18.6	653.7	67.9	548.2	15.2	83.9
21AR225_168	193.0	613.881	1.763	13.531	10.4	0.11556	32.3	0.01676	2.8	0.086	107.1	3.0	111.0	33.9	196.9		107.1	3.0	54.4
21AR225_169	301.9	4501.738	0.642	18.136	3.3	0.47860	6.3	0.06681	1.6	0.262	416.9	6.6	397.1	20.6	284.3	138.5	416.9	6.6	146.6
21AR225_170	673.0	8278.262	1.282	19.045	2.8	0.27849	5.6	0.03976	1.3	0.229	251.4	3.2	249.5	12.5	232.7	126.6	251.4	3.2	108.0
21AR225_171	461.8	8106.624	2.252	18.792	2.9	0.32590	6.3	0.04592	1.4	0.229	289.4	4.1	286.4	15.7	262.9	141.0	289.4	4.1	110.1
21AR225_172	616.6	21915.073	21.489	15.962	1.6	0.93911	2.9	0.10948	1.9	0.667	669.7	12.2	672.4	14.2	682.4	46.0	669.7	12.2	98.1
21AR225_173	464.2	5477.657	0.944	18.926	3.3	0.27452	7.1	0.03955	1.5	0.212	250.1	3.7	246.3	15.4	211.7	160.0	250.1	3.7	118.1
21AR225_174	912.1	305.745	0.839	8.399	30.0	0.01157	84.2	0.00116	6.5	0.078	7.5	0.5	11.7	9.8	1001.1	1782.8	7.5	0.5	0.7
21AR225_175	96.1	2876.758	1.924	15.951	6.3	0.62478	12.0	0.07832	2.0	0.167	486.1	9.4	492.8	47.1	525.2	260.8	486.1	9.4	92.6
21AR225_176	244.0	14907.294	2.702	13.385	1.8	1.74732	3.4	0.17120	2.6	0.765	1018.7	24.2	1026.2	21.7	1043.0	43.7	1043.0	43.7	97.7
21AR225_177	233.1	13823.972	0.825	13.168	1.7	1.90025	2.5	0.18352	1.6	0.645	1086.2	15.9	1081.2	16.4	1072.0	37.9	1072.0	37.9	101.3
21AR225_178	85.5	10676.628	0.988	8.244	1.7	5.94372	2.3	0.35782	1.3	0.560	1971.9	22.3	1967.6	20.4	1964.0	34.7	1964.0	34.7	100.4
21AR225_179	64.4	1376.220	1.902	13.786	11.2	0.50160	22.4	0.05844	2.9	0.128	366.2	10.2	412.8	76.0	683.6	475.9	366.2	10.2	53.6
21AR225_180	201.4	2620.446	0.755	16.232	7.9	0.31679	12.7	0.04085	1.8	0.141	258.1	4.5	279.4	31.1	462.8	279.2	258.1	4.5	55.8
21AR225_181	299.9	17817.427	2.772	13.549	1.6	1.74702	2.7	0.17296	1.7	0.633	1028.4	16.4	1026.1	17.6	1022.0	42.6	1022.0	42.6	100.6
21AR225_182	453.6	7925.498	3.053	17.795	2.8	0.47933	4.5	0.06364	1.6	0.352	397.7	6.1	397.6	14.7	397.9	93.8	397.7	6.1	100.0
21AR225_183	452.8	833.653	7.047	15.614	2.5	0.49318	21.6	0.07672	2.5	0.115	476.5	11.4	407.1	72.4	30.9		476.5	11.4	1540.4
21AR225_184	420.2	4597.834	0.866	18.534	3.6	0.28337	9.7	0.04023	1.4	0.140	254.3	3.4	253.3	21.8	245.7	221.4	254.3	3.4	103.5
21AR225_185	16.6	603.061	0.919	10.854	15.8	1.17043	39.0	0.12342	3.9	0.101	750.2	28.0	786.8	214.3	893.1	808.0	750.2	28.0	84.0
21AR225_186	55.9	65.871	1.755	4.338	42.7	-0.01076	2491.9	0.00790	14.1	0.006	50.7	7.1	NA	-276.9	NA		50.7	7.1	
21AR225_187	146.6	3128.462	3.122	16.927	6.3	0.42822	9.1	0.05685	2.1	0.233	356.4	7.3	361.9	27.6	398.2	197.4	356.4	7.3	89.5
21AR225_188	343.6	1111.803	1.415	17.101	5.0	0.24280	13.0	0.03882	3.2	0.244	245.5	7.6	220.7	25.7	NA		245.5	7.6	
21AR225_189	451.6	4211.578	2.187	16.895	5.7	0.21696	7.5	0.02812	1.5	0.201	178.8	2.7	199.4	13.6	451.4	163.9	178.8	2.7	39.6
21AR225_190	265.3	3363.432	1.425	18.199	4.0	0.31419	14.4	0.04479	1.8	0.122	282.4	4.9	277.4	35.1	236.3	331.1	282.4	4.9	119.5
21AR225_191	42.0	334.238	0.609	7.765	22.9	0.33577	81.6	0.02791	6.2	0.075	177.5	10.8	294.0	209.1	1366.8	1638.0	177.5	10.8	13.0
21AR225_192	139.1	2734.848	2.314	16.128	5.5	0.46632	10.1	0.05939	1.7	0.167	371.9	6.1	388.6	32.5	490.4	218.9	371.9	6.1	75.8
21AR225_193	157.2	7432.338	3.639	14.008	3.2	1.54672	6.8	0.16074	2.5	0.374	960.9	22.6	949.2	41.8	923.0	129.1	923.0	129.1	104.1
21AR225_194	989.2	13753.054	11.545	8.600	1.3	4.57180	1.9	0.28646	1.4	0.708	1623.8	19.6	1744.1	16.1	1892.3	24.5	1892.3	24.5	85.8

21AR225_195	490.2	7472.510	3.885	17.880	2.9	0.48049	5.8	0.06437	2.3	0.405	402.2	9.1	398.4	19.0	377.7	118.4	402.2	9.1	106.5
21AR225_196	183.9	5216.967	4.142	17.008	4.2	0.65828	6.4	0.08500	1.8	0.277	525.9	8.9	513.6	25.6	460.1	135.4	525.9	8.9	114.3
21AR225_197	743.2	23160.997	15.316	16.439	1.4	0.81341	2.4	0.09770	1.4	0.582	600.9	8.1	604.4	11.0	618.2	42.3	600.9	8.1	97.2
21AR225_198	378.2	3799.294	1.716	17.708	5.4	0.21231	10.4	0.02918	1.8	0.170	185.4	3.2	195.5	18.6	320.0	234.0	185.4	3.2	57.9
21AR225_199	93.9	852.930	0.705	13.182	12.6	0.23634	26.8	0.02906	3.1	0.117	184.6	5.7	215.4	52.0	567.7	581.5	184.6	5.7	32.5
21AR225_200	175.9	2639.314	2.005	16.941	7.3	0.32758	12.3	0.04433	1.7	0.137	279.6	4.6	287.7	30.8	354.9	275.4	279.6	4.6	78.8
21AR225_201	160.9	2048.113	1.065	16.080	7.0	0.30214	21.0	0.03972	1.6	0.076	251.1	3.9	268.1	49.6	420.1	469.3	251.1	3.9	59.8
21AR225_202	252.0	6745.540	1.518	16.364	3.4	0.70184	4.7	0.08616	1.6	0.331	532.8	8.0	539.9	19.8	570.8	97.2	532.8	8.0	93.3
21AR225_203	30.2	1290.923	2.498	12.177	11.5	1.41285	27.4	0.14328	2.6	0.096	863.2	21.1	894.4	163.0	973.2	557.7	973.2	557.7	88.7
21AR225_204	446.7	15282.868	2.921	16.350	1.5	0.84100	2.6	0.10110	1.7	0.637	620.9	9.8	619.7	12.1	616.4	43.4	620.9	9.8	100.7
21AR225_205	88.3	343.546	1.599	8.249	23.4	0.13870	37.7	0.01255	7.5	0.199	80.4	6.0	131.9	46.6	1201.4	734.6	80.4	6.0	6.7
21AR225_206	74.6	1083.333	2.764	11.024	15.2	0.50235	22.7	0.04684	2.6	0.116	295.1	7.6	413.3	77.0	1142.0	448.8	295.1	7.6	25.8
21AR225_207	931.6	6713.245	1.006	19.746	2.8	0.16165	3.8	0.02415	1.7	0.461	153.8	2.7	152.1	5.4	126.9	79.1	153.8	2.7	121.3
21AR225_208	114.4	672.737	2.824	15.019	6.4	0.51156	97.2	0.08294	2.0	0.021	513.6	9.9	419.5	337.2	NA		513.6	9.9	
21AR225_209	390.8	4032.741	2.339	17.689	4.1	0.21747	6.5	0.02974	2.5	0.385	188.9	4.7	199.8	11.8	331.1	136.1	188.9	4.7	57.1
21AR225_210	281.9	23170.695	1.080	9.356	1.2	4.36947	2.1	0.29726	1.5	0.713	1677.7	21.7	1706.6	17.0	1743.0	26.4	1743.0	26.4	96.3
21AR225_211	181.1	649.697	1.396	9.753	17.8	0.12371	52.8	0.01108	3.9	0.073	71.0	2.7	118.4	59.0	1222.2	1052.8	71.0	2.7	5.8
21AR225_212	344.4	4158.148	1.088	18.117	4.7	0.29480	8.9	0.04124	1.8	0.202	260.5	4.6	262.3	20.6	279.5	199.6	260.5	4.6	93.2
21AR225_213	452.4	6915.341	1.835	18.386	4.4	0.32975	5.9	0.04564	1.8	0.308	287.7	5.1	289.4	14.8	304.0	127.1	287.7	5.1	94.6
21AR225_214	188.3	2784.301	2.230	16.592	6.2	0.34514	15.3	0.04537	2.1	0.137	286.1	5.8	301.1	39.8	420.0	338.1	286.1	5.8	68.1
21AR225_215	431.7	19896.598	1.986	13.159	1.2	1.83829	2.3	0.17666	1.3	0.560	1048.7	12.7	1059.2	15.4	1081.9	38.8	1081.9	38.8	96.9
21AR225_216	71.1	225.830	3.544	7.269	34.5	0.11079	88.0	0.01077	8.6	0.098	69.1	5.9	106.7	89.2	1058.8	1854.8	69.1	5.9	6.5
21AR225_217	350.9	161.980	1.007	6.540	44.6	0.01352	132.4	0.00154	19.5	0.148	9.9	1.9	13.6	17.9	734.0		9.9	1.9	1.3
21AR225_218	1208.9	2247.944	15.864	16.298	1.3	0.56507	4.6	0.07444	2.9	0.630	462.9	12.8	454.8	16.7	415.2	79.1	462.9	12.8	111.5
21AR225_219	48.2	844.785	1.147	13.035	9.9	0.48804	43.2	0.05924	2.6	0.061	371.0	9.5	403.6	144.1	595.4	944.2	371.0	9.5	62.3
21AR225_220	1009.7	1020.842	2.283	17.421	3.5	0.21182	38.6	0.03571	6.5	0.168	226.2	14.4	195.1	68.6	NA		226.2	14.4	
21AR225_221	84.2	1368.209	2.266	14.527	10.0	0.41162	32.3	0.05111	2.2	0.069	321.3	7.0	350.0	95.7	546.0	707.9	321.3	7.0	58.8
21AR225_222	71.1	1268.869	3.441	15.150	8.7	0.45044	33.9	0.05956	2.3	0.069	372.9	8.5	377.6	106.9	407.2	761.1	372.9	8.5	91.6
21AR225_223	510.6	13754.338	2.471	17.171	2.0	0.70562	3.9	0.08914	1.7	0.422	550.5	8.8	542.1	16.5	508.3	78.4	550.5	8.8	108.3
21AR225_224	100.1	14437.337	0.786	8.686	1.5	5.40855	2.5	0.34300	1.6	0.643	1901.1	26.7	1886.2	21.6	1870.7	34.9	1870.7	34.9	101.6
21AR225_225	158.8	714.681	2.288	12.860	14.8	0.17891	62.8	0.02258	2.8	0.044	144.0	3.9	167.1	96.8	510.3		144.0	3.9	28.2
21AR225_226	219.3	8614.525	2.309	15.236	2.2	1.09647	4.1	0.12414	1.8	0.452	754.4	13.1	751.6	21.6	744.5	76.6	754.4	13.1	101.3
21AR225_227	62.1	1269.970	2.351	13.557	9.6	0.54626	36.1	0.06341	2.2	0.060	396.3	8.3	442.5	129.7	691.5	774.5	396.3	8.3	57.3
21AR225_228	252.2	15996.595	4.545	11.504	1.7	2.74171	2.8	0.23074	1.9	0.697	1338.4	23.4	1339.8	20.7	1343.0	38.5	1343.0	38.5	99.7
21AR225_229	507.4	8096.875	2.539	18.500	2.9	0.30172	5.6	0.04169	1.7	0.298	263.3	4.3	267.7	13.1	307.5	121.2	263.3	4.3	85.6
21AR225_230	466.8	622.209	1.456	15.810	5.1	0.16583	44.1	0.03044	5.4	0.124	193.3	10.4	155.8	63.7	NA		193.3	10.4	
21AR225_231	382.7	9424.506	2.090	17.736	2.1	0.57630	3.5	0.07585	2.0	0.571	471.3	9.0	462.1	12.9	417.3	63.8	471.3	9.0	112.9
21AR225_232	94.4	910.113	2.120	15.383	8.1	0.39969	38.5	0.05903	1.6	0.042	369.7	5.8	341.4	111.9	153.8		369.7	5.8	240.4
21AR225_233	38.3	691.963	2.435	12.112	15.7	0.52924	59.2	0.06195	3.9	0.065	387.5	14.5	431.3	208.8	673.8	1289.8	387.5	14.5	57.5
21AR225_234	173.7	3353.712	1.862	17.633	4.5	0.44721	11.1	0.06166	1.6	0.144	385.7	6.0	375.3	35.0	312.7	251.1	385.7	6.0	123.3
21AR225_235	693.5	26495.173	54.992	14.184	2.1	1.40904	8.9	0.14622	8.6	0.971	879.8	70.7	892.8	52.6	926.0	43.3	926.0	43.3	95.0
21AR225_236	244.2	29450.238	0.802	8.669	1.3	5.32173	2.0	0.33554	1.3	0.666	1865.2	21.2	1872.4	16.8	1881.1	26.5	1881.1	26.5	99.2
21AR225_237	98.6	2514.084	0.775	15.411	11.4	0.51689	15.4	0.06334	1.9	0.123	395.9	7.3	423.1	53.4	574.9	333.4	395.9	7.3	68.9
21AR225_238	287.7	3482.980	1.853	17.936	4.9	0.27943	7.6	0.03927	1.6	0.208	248.3	3.9	250.2	16.9	268.9	171.4	248.3	3.9	92.4
21AR225_239	83.9	1396.140	1.009	13.216	13.7	0.37390	34.1	0.04145	2.4	0.069	261.8	6.0	322.5	94.2	788.8	718.1	261.8	6.0	33.2
21AR225_240	422.1	17695.697	7.300	15.406	2.1	0.96660	3.9	0.10901	2.8	0.709	667.0	17.7	686.7	19.6	752.7	58.5	667.0	17.7	88.6
21AR225_241	155.3	2998.905	3.624	17.238	3.6	0.55005	6.6	0.07455	2.6	0.390	463.5	11.4	445.0	23.6	351.6	136.6	463.5	11.4	131.8
21AR225_242	129.6	5241.870	2.028	14.004	3.1	1.46758	6.0	0.15390	3.4	0.557	922.8	28.9	917.1	36.5	904.5	103.3	904.5	103.3	102.0
21AR225_243	201.9	5244.283	1.795	13.010	1.8	1.81136	5.8	0.17604	1.5	0.267	1045.3	14.8	1049.6	37.7	1059.4	111.7	1059.4	111.7	98.7
21AR225_244	563.4	2239.530	3.768	17.845	2.7	0.41458	21.0	0.06034	1.3	0.062	377.7	4.8	352.2	62.4	188.1		377.7	4.8	200.8
21AR225_245	216.5	2019.402	0.877	16.266	7.9	0.29436	10.8	0.03901	1.5	0.143	246.7	3.7	262.0	25.0	402.3	239.8	246.7	3.7	61.3
21AR225_246	211.4	2716.960	1.636	16.337	7.3	0.30350	17.5	0.03906	1.7	0.099	247.0	4.2	269.1	41.3	467.2	385.3	247.0	4.2	52.9
21AR225_247	65.2	697.143	1.090	11.990	14.1	0.29401	66.9	0.03372	3.2	0.048	213.8	6.8	261.7	154.7	716.8	1458.5	213.8	6.8	29.8
21AR225_248	214.9	6872.631	2.004	17.030	3.6	0.68245	6.5	0.08666	1.5	0.229	535.7	7.7	528.3	26.8	497.0	139.6	535.7	7.7	107.8
21AR225_249	538.1	6736.576	1.490	18.771	3.1	0.29380	5.8	0.04133	2.4	0.419	261.1	6.2	261.5	13.3	266.9	119.8	261.1	6.2	97.8
21AR225_250	292.7	3834.588	1.899	17.514	3.6	0.45225	4.8	0.06100	1.4	0.282	381.7	5.0	378.9	15.2	362.4	104.2	381.7	5.0	105.3
21AR225_251	86.9	1110.080	2.217	14.703	9.1	0.45815	34.3	0.06001	2.0	0.059	375.7	7.4	383.0	109.4	428.2	767.8	375.7	7.4	87.7
21AR225_252	121.4	1154.678	0.824	15.250	10.7	0.21267	48.2	0.02893	3.0	0.062	183.8	5.4	195.8	86.0	343.6		183.8	5.4	53.5
21AR225_253	143.4	2154.825	0.768	15.721	7.1	0.39277	14.0	0.04980	1.7	0.122	313.3	5.2	336.4	40.2	500.5	307.0	313.3	5.2	62.6
21AR225_254	214.4	3130.041	2.623	17.225	5.4	0.42984	8.7	0.05804	1.7	0.197	363.7	6.1	363.1	26.5	360.1	192.2	363.7	6.1	101.0
21AR225_255	298.9	4127.939	1.759	18.302	4.1	0.28405	7.7	0.04008	1.5	0.190	253.3	3.6	253.9	17.3	259.8	173.8	253.3	3.6	97.5
21AR225_256	525.4	5483.319	1.412	18.477	3.1	0.28229	10.5	0.03955	1.9	0.185	250.0	4.8	252.5	23.4	276.0	235.7	250.0	4.8	90.6
21AR225_257	487.5	2066.341	8.428	16.638	3.1	0.49757	6.0	0.06755	2.0	0.339	421.4	8.3	410.1	20.2	347.8	127.1	421.4	8.3	121.1
21AR225_258	361.8	6442.885	1.317	17.055	2.8	0.56512	8.3	0.07207	1.9	0.228	448.6	8.2	454.9	30.4	487.5	177.9	448.6	8.2	92.0
21AR225_259	516.9	13815.081	1.157	17.038	1.7	0.69632	3.2	0.08685	1.5	0.476	536.9	7.9	536.6	13.4	536.2	61.8	536.9	7.9	100.1
21AR225_260	202.5	2692.782	4.905	17.928	6.4	0.27731	15.3	0.03962	1.8	0.116	250.4	4.3	248.5	33.8	231.3	351.6	250.4	4.3	108.3
21AR225_261	353.9	8556.059	10.502	16.211	2.8	0.64999	5.6	0.07808	1.4	0.253	484.7	6.6	508.5	22.2	617.9	116.0	484.7	6.6	78.4
**21AR226**																			
21AR226_01	96.2	29130.111	2.207	11.090	2.1	2.96041	2.5	0.23981	1.4	0.549	1385.7	17.2	1397.5	19.1	1416.4	40.2	1416.4	40.2	97.8
21AR226_02	347.5	9008.865	5.554	16.593	2.6	0.55524	4.2	0.06877	2.3	0.548	428.8	9.5	448.4	15.2	551.5	76.7	428.8	9.5	77.7
21AR226_03	49.6	3808.943	1.396	12.466	12.5	0.50643	13.6	0.04810	3.3	0.240	302.9	9.6	416.0	46.4	1105.3	263.9	302.9	9.6	27.4
21AR226_04	253.7	3698.205	6.325	15.999	3.0	0.61828	3.6	0.07672	1.5	0.406	476.5	6.7	488.8	13.9	547.4	71.5	476.5	6.7	87.1
21AR226_05	169.1	7362.304	1.021	13.182	2.7	1.61384	4.1	0.15869	1.3	0.329	949.5	11.8	975.6	25.4	1035.7	77.3	1035.7	77.3	91.7
21AR226_06	666.4	14770.317	1.909	18.666	2.8	0.28505	4.2	0.03945	1.8	0.436	249.4	4.5	254.7	9.4	304.3	85.6	249.4	4.5	82.0
21AR226_07	211.8	5194.768	1.612	15.170	10.8	0.21836	11.5	0.02517	2.4	0.206	160.3	3.7	200.5	20.8	706.3	238.6	160.3	3.7	22.7
21AR226_08	95.0	41966.624	1.884	8.245	1.9	5.49126	3.0	0.33047	2.3	0.763	1840.7	36.8	1899.2	25.9	1964.6	34.8	1964.6	34.8	93.7
21AR226_09	361.4	17724.571	1.850	18.053	4.4	0.29590	5.1	0.03949	1.5	0.299	249.7	3.8	263.2	11.9	386.1	109.8	249.7	3.8	64.7
21AR226_10	318.7	4479.406	1.682	16.986	5.1	0.46827	12.7	0.06132	3.9	0.303	383.7	14.3	390.0	41.2	428.6	270.3	383.7	14.3	89.5
21AR226_11	265.2	809.988	1.433	14.498	8.2	0.17472	25.7	0.02503	2.2	0.084	159.4	3.4	163.5	38.8	224.9		159.4	3.4	70.8
21AR226_12	73.6	1329.980	2.321	9.130	22.6	0.18726	27.3	0.01376	6.9	0.252	88.1	6.0	174.3	43.7	1601.1	494.6	88.1	6.0	5.5
21AR226_13	503.4	78043.487	9.925	8.889	1.5	4.43984	3.9	0.28788	3.5	0.921	1630.9	51.1	1719.8	31.9	1830.5	27.2	1830.5	27.2	89.1
21AR226_14	206.3	40340.393	1.153	12.936	1.9	1.79335	2.4	0.16969	1.4	0.578	1010.4	13.1	1043.0	15.8	1112.9	39.6	1112.9	39.6	90.8
21AR226_15	112.1	5117.327	1.317	15.158	7.9	0.35526	9.3	0.04096	2.3	0.252	258.8	5.9	308.7	24.7	705.7	191.3	258.8	5.9	36.7
21AR226_16	631.3	2956.180	2.057	16.785	2.5	0.35028	4.1	0.04668	3.0	0.742	294.1	8.7	304.9	10.7	389.5	61.2	294.1	8.7	75.5
21AR226_17	731.6	29974.157	6.175	15.677	1.9	0.84964	4.0	0.09773	3.2	0.814	601.1	18.5	624.4	18.4	710.9	48.8	601.1	18.5	84.5
21AR226_18	217.2	2333.890	1.733	12.708	22.2	0.10076	25.4	0.01011	4.0	0.158	64.9	2.6	97.5	23.6	994.6	512.2	64.9	2.6	6.5
21AR226_19	316.6	10241.514	0.724	16.757	6.4	0.21791	7.4	0.02724	2.3	0.308	173.3	3.9	200.2	13.5	531.2	155.0	173.3	3.9	32.6
21AR226_20	424.8	759.283	1.235	6.769	21.9	0.02087	30.3	0.00117	16.0	0.529	7.5	1.2	21.0	6.3	2089.8	453.7	7.5	1.2	0.4
21AR226_21	190.9	27290.614	2.391	16.275	3.2	0.80610	3.7	0.09670	1.5	0.407	595.1	8.5	600.3	16.5	620.8	71.9	595.1	8.5	95.8
21AR226_22	122.8	5471.637	1.136	14.082	13.4	0.36503	13.8	0.03900	2.1	0.153	246.6	5.1	316.0	37.4	865.8	282.7	246.6	5.1	28.5
21AR226_23	86.1	5263.951	1.951	13.809	14.4	0.45547	15.1	0.04786	3.8	0.250	301.4	11.1	381.1	48.1	900.3	302.5	301.4	11.1	33.5
21AR226_24	149.0	2855.845	1.165	12.600	15.0	0.14985	17.3	0.01473	4.0	0.233	94.3	3.8	141.8	22.9	1036.1	340.1	94.3	3.8	9.1
21AR226_25	129.3	9636.804	14.825	16.252	7.7	0.59739	8.2	0.07268	2.5	0.304	452.3	10.9	475.6	31.2	590.6	170.0	452.3	10.9	76.6
21AR226_26	342.7	1511.097	1.666	14.965	2.5	0.52520	4.0	0.06705	2.5	0.618	418.4	10.0	428.6	14.0	485.1	69.4	418.4	10.0	86.2
21AR226_27	239.2	10402.710	1.959	16.648	6.5	0.22661	7.3	0.02821	2.2	0.300	179.3	3.9	207.4	13.8	540.8	153.2	179.3	3.9	33.2
21AR226_28	3924.1	2998.814	0.491	10.945	14.5	0.00815	17.5	0.00069	2.9	0.164	4.4	0.1	8.2	1.4	1341.1	334.0	4.4	0.1	0.3
21AR226_29	173.7	3433.506	1.568	13.298	12.1	0.20726	13.4	0.02123	3.0	0.221	135.4	4.0	191.3	23.3	952.7	266.7	135.4	4.0	14.2
21AR226_30	229.0	73398.867	4.675	9.588	1.4	3.97991	2.1	0.27813	1.6	0.771	1582.0	23.0	1630.1	17.3	1693.6	25.0	1693.6	25.0	93.4
21AR226_31	177.8	10147.907	0.771	16.136	8.5	0.33805	9.2	0.04062	1.9	0.208	256.7	4.8	295.7	23.5	617.3	193.9	256.7	4.8	41.6
21AR226_32	520.9	9177.736	1.906	17.649	5.5	0.13522	7.3	0.01788	1.8	0.240	114.2	2.0	128.8	8.8	407.1	158.5	114.2	2.0	28.1
21AR226_33	527.8	30425.811	2.525	18.932	3.2	0.32158	3.6	0.04474	1.6	0.454	282.1	4.5	283.1	9.0	292.3	73.8	282.1	4.5	96.5
21AR226_34	115.5	22799.417	3.582	13.137	3.0	1.89553	3.8	0.18281	1.7	0.448	1082.3	17.0	1079.5	25.3	1074.8	68.2	1074.8	68.2	100.7
21AR226_35	62.2	656.973	2.758	5.850	46.9	0.19671	50.1	0.00948	12.8	0.256	60.8	7.8	182.3	83.7	2351.7	841.0	60.8	7.8	2.6
21AR226_36	77.6	8099.418	-2559.928	15.307	6.2	0.74385	7.6	0.08544	1.8	0.234	528.5	9.0	564.7	32.9	714.0	157.0	528.5	9.0	74.0
21AR226_37	75.0	1138.567	1.163	10.864	20.1	0.25977	22.1	0.02382	5.7	0.255	151.7	8.5	234.5	46.4	1175.7	424.6	151.7	8.5	12.9
21AR226_38	165.9	11035.738	6.170	16.896	6.1	0.48396	7.5	0.06089	1.9	0.248	381.1	6.9	400.8	24.9	517.1	160.2	381.1	6.9	73.7
21AR226_39	88.8	4029.211	1.851	13.243	15.3	0.39772	16.7	0.04025	2.5	0.151	254.4	6.3	340.0	48.2	977.6	336.7	254.4	6.3	26.0
21AR226_40	258.7	31088.263	2.819	17.063	3.6	0.68405	4.1	0.08568	1.9	0.451	529.9	9.4	529.2	17.0	527.2	80.4	529.9	9.4	100.5
21AR226_41	153.0	6787.595	2.083	15.651	10.4	0.33333	10.9	0.03930	1.9	0.172	248.5	4.6	292.1	27.6	658.1	229.6	248.5	4.6	37.8
21AR226_42	136.2	7184.518	2.398	14.347	9.2	0.38361	10.3	0.04126	2.2	0.212	260.6	5.6	329.7	28.9	852.0	208.7	260.6	5.6	30.6
21AR226_43	78.6	13160.069	1.305	14.173	4.7	1.38951	5.2	0.14560	1.8	0.342	876.2	14.5	884.5	30.6	906.1	100.5	876.2	14.5	96.7
21AR226_44	19.5	1558.971	0.432	9.814	19.5	0.88721	25.5	0.06944	7.9	0.309	432.8	33.0	644.9	121.9	1481.7	461.3	1481.7	461.3	29.2
21AR226_45	340.2	12374.879	0.714	16.638	6.6	0.21860	7.4	0.02700	1.7	0.233	171.7	2.9	200.7	13.5	557.9	157.2	171.7	2.9	30.8
21AR226_46	108.9	19213.045	1.915	15.972	5.0	0.85429	5.6	0.10053	1.7	0.311	617.5	10.2	627.0	26.1	662.3	113.6	617.5	10.2	93.2
21AR226_47	209.7	13375.973	1.678	17.260	4.1	0.48308	4.6	0.06184	1.6	0.350	386.8	6.1	400.2	15.3	479.1	96.0	386.8	6.1	80.7

21AR226_48	1457.9	2105.292	0.360	7.724	20.7	0.01982	22.7	0.00117	5.1	0.223	7.5	0.4	19.9	4.5	1996.5	393.7	7.5	0.4	0.4
21AR226_49	65.8	3549.328	0.829	11.111	15.6	0.47739	17.1	0.04037	3.6	0.213	255.1	9.1	396.3	56.3	1333.5	324.6	255.1	9.1	19.1
21AR226_50	46.9	9607.967	0.590	12.809	5.8	1.79230	6.3	0.17029	2.1	0.328	1013.7	19.4	1042.7	41.2	1104.7	119.3	1104.7	119.3	91.8
21AR226_51	71.4	3186.604	1.233	12.627	17.1	0.32145	19.7	0.03131	4.5	0.226	198.7	8.7	283.0	48.6	1054.8	386.5	198.7	8.7	18.8
21AR226_52	63.8	4629.192	0.350	14.345	11.4	0.55579	12.0	0.06075	2.1	0.171	380.2	7.6	448.8	43.6	818.4	247.8	380.2	7.6	46.5
21AR226_53	412.2	23043.353	2.976	19.208	3.1	0.29810	3.6	0.04220	1.4	0.389	266.5	3.7	264.9	8.4	252.3	76.6	266.5	3.7	105.6
21AR226_54	316.9	1289.731	1.240	15.147	6.0	0.29404	33.0	0.03913	1.4	0.041	247.5	3.3	261.7	76.1	392.4	743.1	247.5	3.3	63.1
21AR226_55	123.6	2169.207	2.816	14.891	7.5	0.49581	14.3	0.05970	1.8	0.124	373.8	6.4	408.9	48.0	612.9	306.2	373.8	6.4	61.0
21AR226_56	203.9	19951.316	3.307	17.225	3.2	0.56933	4.5	0.07232	1.8	0.407	450.1	7.9	457.6	16.4	496.3	89.7	450.1	7.9	90.7
21AR226_57	226.7	8786.185	4.180	15.299	3.3	0.77138	5.2	0.08815	2.0	0.390	544.6	10.7	580.6	23.1	724.9	102.2	544.6	10.7	75.1
21AR226_58	554.8	21516.725	1.249	18.260	3.5	0.34929	4.2	0.04702	1.6	0.372	296.2	4.6	304.2	11.1	367.1	88.4	296.2	4.6	80.7
21AR226_59	109.2	22615.453	0.944	12.801	3.0	1.91834	3.5	0.18020	1.6	0.463	1068.1	16.1	1087.5	23.5	1127.5	62.2	1127.5	62.2	94.7
21AR226_60	143.4	1082.481	2.865	13.661	10.8	0.22616	20.4	0.02754	2.6	0.128	175.1	4.5	207.0	38.2	588.9	440.0	175.1	4.5	29.7
21AR226_61	132.5	13007.540	1.392	15.987	4.5	0.65108	5.7	0.07714	2.4	0.421	479.1	11.0	509.1	22.6	647.6	110.3	479.1	11.0	74.0
21AR226_62	250.9	4719.277	1.203	14.802	9.0	0.13987	10.0	0.01579	3.2	0.321	101.0	3.2	132.9	12.5	751.1	200.1	101.0	3.2	13.4
21AR226_63	109.6	4424.559	0.969	14.757	7.7	0.33523	10.2	0.03782	2.7	0.264	239.3	6.3	293.5	26.0	751.7	208.1	239.3	6.3	31.8
21AR226_64	1612.9	11562.020	2.665	17.943	1.4	0.42863	2.0	0.05722	1.4	0.681	358.7	4.9	362.2	6.2	385.9	33.7	358.7	4.9	93.0
21AR226_65	383.2	445.752	2.731	5.774	39.1	0.02113	46.7	0.00107	18.3	0.393	6.9	1.3	21.2	9.8	2273.4	748.7	6.9	1.3	0.3
21AR226_66	342.9	8337.626	6.799	13.382	2.9	0.65260	4.8	0.06498	2.4	0.487	405.9	9.2	510.1	19.4	1010.3	85.5	405.9	9.2	40.2
21AR226_67	403.5	18132.204	1.586	18.441	3.5	0.27173	4.4	0.03696	1.7	0.393	233.9	4.0	244.1	9.6	343.7	92.1	233.9	4.0	68.1
21AR226_68	141.5	673.345	0.968	11.771	9.1	0.23893	49.8	0.02736	2.8	0.056	174.0	4.8	217.5	97.5	720.3	1070.0	174.0	4.8	24.2
21AR226_69	491.9	9984.691	1.156	17.061	3.2	0.55217	6.1	0.07018	1.7	0.272	437.2	7.1	446.4	22.2	495.1	130.5	437.2	7.1	88.3
21AR226_70	183.0	8455.316	2.370	13.157	2.3	1.59941	2.8	0.15632	1.4	0.508	936.3	12.2	970.0	17.3	1047.9	48.0	1047.9	48.0	89.3
21AR226_71	229.0	19296.326	3.018	17.340	4.5	0.48304	5.0	0.06165	1.7	0.337	385.7	6.4	400.2	16.7	485.8	104.7	385.7	6.4	79.4
21AR226_72	1355.0	1487.731	1.841	10.903	25.0	0.01245	30.8	0.00110	15.0	0.486	7.1	1.1	12.6	3.8	1248.6	529.6	7.1	1.1	0.6
21AR226_73	217.8	31104.163	2.550	15.637	2.5	1.05071	3.5	0.12012	2.3	0.671	731.3	16.2	729.2	18.2	723.9	55.0	731.3	16.2	101.0
21AR226_74	3309.7	4139.627	0.324	14.101	14.5	0.01104	15.6	0.00119	2.9	0.183	7.7	0.2	11.1	1.7	850.4	320.2	7.7	0.2	0.9
21AR226_75	68.2	5485.297	4.219	14.980	6.6	0.92204	7.6	0.10437	2.5	0.324	639.9	15.0	663.4	37.1	745.0	152.3	639.9	15.0	85.9
21AR226_76	363.1	2262.366	12.066	16.376	3.4	0.43271	4.7	0.05746	2.0	0.424	360.2	7.0	365.1	14.4	397.4	95.2	360.2	7.0	90.6
21AR226_77	145.4	6619.219	1.223	16.651	8.2	0.32481	8.8	0.04072	2.1	0.236	257.3	5.3	285.6	22.0	525.2	188.5	257.3	5.3	49.0
21AR226_78	230.6	18166.056	0.933	17.271	5.6	0.44950	6.1	0.05710	2.0	0.327	357.9	6.9	376.9	19.2	496.2	127.2	357.9	6.9	72.1
21AR226_79	262.9	13793.050	0.855	17.795	5.3	0.34915	6.0	0.04590	1.8	0.298	289.3	5.0	304.1	15.7	420.0	127.8	289.3	5.0	68.9
21AR226_80	52.6	1271.337	1.723	6.123	31.9	0.32028	33.9	0.01516	7.6	0.223	97.0	7.3	282.1	83.6	2383.1	567.6	97.0	7.3	4.1
21AR226_81	1226.4	29045.097	1.998	19.235	2.5	0.15686	3.3	0.02210	1.5	0.458	140.9	2.1	147.9	4.5	263.4	66.7	140.9	2.1	53.5
21AR226_82	267.4	8120.755	1.015	17.325	7.5	0.18289	8.3	0.02372	2.0	0.241	151.1	3.0	170.5	13.1	450.1	179.7	151.1	3.0	33.6
21AR226_83	66.8	3209.091	1.707	11.095	17.3	0.46103	19.9	0.03902	3.3	0.164	246.7	7.9	385.0	63.9	1332.3	381.4	246.7	7.9	18.5
21AR226_84	614.0	21739.452	19.665	12.208	2.2	1.06244	4.1	0.09505	3.3	0.802	585.3	18.6	735.0	21.7	1224.0	48.6	1224.0	48.6	47.8
21AR226_85	63.0	2095.952	0.796	10.815	13.4	0.54966	16.7	0.04664	3.1	0.188	293.9	9.0	444.8	60.2	1326.9	318.2	293.9	9.0	22.1
21AR226_86	409.0	17546.385	0.494	18.358	3.8	0.28015	4.3	0.03799	1.6	0.372	240.4	3.8	250.8	9.6	350.1	90.4	240.4	3.8	68.7
21AR226_87	187.3	2560.148	2.540	9.524	22.5	0.14245	24.0	0.01040	4.4	0.181	66.7	2.9	135.2	30.4	1612.1	441.8	66.7	2.9	4.1
21AR226_88	111.6	8209.777	2.530	15.812	7.3	0.50980	7.9	0.06032	1.9	0.245	377.5	7.1	418.3	27.2	650.8	165.1	377.5	7.1	58.0
21AR226_89	325.7	22439.757	15.512	17.115	2.8	0.64895	3.3	0.08177	1.5	0.445	506.7	7.1	507.8	13.1	514.1	64.3	506.7	7.1	98.6
21AR226_90	317.1	24413.253	1.205	18.218	4.0	0.44683	4.7	0.05995	1.6	0.336	375.3	5.8	375.1	14.8	374.4	99.8	375.3	5.8	100.3
21AR226_91	176.2	14415.235	4.707	17.272	4.1	0.57355	4.6	0.07344	1.6	0.347	456.8	7.1	460.3	17.2	478.6	96.2	456.8	7.1	95.5
21AR226_92	149.7	9571.471	2.961	16.393	6.2	0.48660	7.2	0.05959	1.6	0.225	373.1	5.8	402.6	23.9	576.4	152.0	373.1	5.8	64.7
21AR226_93	616.3	503.627	0.735	5.007	52.1	0.01677	57.3	0.00070	16.4	0.286	4.5	0.7	16.9	9.6	2600.8	934.3	4.5	0.7	0.2
21AR226_94	130.1	31612.157	2.138	13.115	2.6	1.77901	3.1	0.17117	1.6	0.530	1018.6	15.5	1037.8	20.1	1079.5	52.7	1079.5	52.7	94.4
21AR226_95	350.9	8783.253	1.625	17.394	4.0	0.28490	5.3	0.03722	1.6	0.299	235.6	3.7	254.5	12.0	434.2	113.1	235.6	3.7	54.3
21AR226_96	133.3	4912.350	0.647	13.345	12.0	0.27101	13.5	0.02745	2.9	0.213	174.5	5.0	243.5	29.2	975.9	269.0	174.5	5.0	17.9
21AR226_97	77.8	2975.641	1.008	10.948	12.9	0.42252	14.4	0.03558	2.7	0.188	225.4	6.0	357.9	43.3	1342.0	272.8	225.4	6.0	16.8
21AR226_98	80.3	4950.870	1.138	13.477	8.5	0.47003	9.6	0.04808	2.1	0.224	302.7	6.3	391.2	31.0	955.5	190.5	302.7	6.3	31.7
21AR226_99	318.7	20940.025	2.873	17.668	3.5	0.47812	4.1	0.06238	1.9	0.460	390.1	7.2	396.8	13.6	437.0	81.7	390.1	7.2	89.3
21AR226_100	541.4	15545.187	2.489	13.212	1.2	1.55393	2.6	0.15162	2.2	0.820	910.1	18.4	952.1	16.4	1051.3	30.6	1051.3	30.6	86.6
21AR226_101	89.4	5278.470	1.554	14.633	9.1	0.54481	9.8	0.06059	1.8	0.186	379.2	6.7	441.6	35.2	782.3	203.2	379.2	6.7	48.5
21AR226_102	182.4	14812.285	1.939	17.615	5.4	0.42552	6.0	0.05565	1.5	0.256	349.1	5.2	360.0	18.2	431.4	129.3	349.1	5.2	80.9
21AR226_103	88.4	14334.750	2.687	15.409	5.7	1.00903	6.1	0.11523	1.8	0.286	703.0	11.7	708.4	31.4	726.3	124.9	703.0	11.7	96.8
21AR226_104	262.4	13772.740	1.188	17.832	5.3	0.31505	5.6	0.04178	1.6	0.277	263.9	4.0	278.1	13.6	400.3	120.4	263.9	4.0	65.9
21AR226_105	389.7	20634.600	1.411	18.240	3.6	0.31145	4.7	0.04200	1.6	0.346	265.2	4.3	275.3	11.5	363.1	100.5	265.2	4.3	73.0
21AR226_106	89.4	6869.225	1.342	15.868	8.2	0.54239	9.4	0.06490	2.3	0.240	405.4	8.8	440.0	33.6	626.3	196.8	405.4	8.8	64.7
21AR226_107	366.5	47914.810	3.261	13.670	1.6	1.49806	2.1	0.14988	1.3	0.636	900.3	11.3	929.6	12.8	1000.7	33.0	1000.7	33.0	90.0
21AR226_108	332.3	17817.480	1.060	17.759	3.6	0.47230	4.4	0.06204	1.8	0.415	388.0	6.8	392.8	14.2	421.9	88.6	388.0	6.8	92.0
21AR226_109	365.8	12155.807	1.417	18.088	5.3	0.19831	6.8	0.02673	1.5	0.224	170.0	2.6	183.7	11.4	364.2	149.6	170.0	2.6	46.7
21AR226_110	24.4	6690.210	1.821	11.399	9.9	2.66403	10.5	0.22673	1.8	0.172	1317.3	21.6	1318.5	77.6	1321.3	200.7	1321.3	200.7	99.7
21AR226_111	690.2	39267.034	2.049	19.030	3.0	0.30796	3.4	0.04295	1.5	0.433	271.1	3.9	272.6	8.0	286.5	69.3	271.1	3.9	94.6
21AR226_112	456.7	34238.633	1.749	17.677	2.8	0.46071	3.4	0.05971	1.3	0.397	373.9	4.9	384.7	10.8	451.6	69.0	373.9	4.9	82.8
21AR226_113	586.9	14312.099	1.440	18.953	2.7	0.28432	3.9	0.03999	1.7	0.431	252.8	4.2	254.1	8.8	267.1	81.1	252.8	4.2	94.7
21AR226_114	215.2	11994.614	1.307	16.943	5.9	0.32057	7.1	0.04035	1.5	0.212	255.0	3.8	282.3	17.5	516.1	152.4	255.0	3.8	49.4
21AR226_115	87.1	4321.028	1.136	12.982	14.5	0.40328	16.6	0.03980	2.4	0.143	251.6	5.8	344.0	48.4	1028.1	332.3	251.6	5.8	24.5
21AR226_116	141.4	16448.012	0.774	9.144	1.4	4.63601	2.4	0.31085	1.5	0.649	1744.9	23.5	1755.8	19.8	1769.5	32.9	1769.5	32.9	98.6
21AR226_117	114.5	5615.734	1.524	15.198	11.6	0.35532	13.1	0.04088	2.2	0.172	258.3	5.7	308.7	34.8	710.2	273.7	258.3	5.7	36.4
21AR226_118	408.3	23278.738	0.913	18.204	3.5	0.44633	4.2	0.05979	1.8	0.431	374.4	6.6	374.7	13.1	377.8	84.8	374.4	6.6	99.1
21AR226_119	537.4	1522.291	0.964	15.962	5.5	0.30574	18.4	0.04194	1.8	0.097	264.8	4.6	270.9	43.9	324.3	417.6	264.8	4.6	81.7
21AR226_120	24.1	561.966	0.612	8.126	26.9	0.51474	43.2	0.03796	10.1	0.235	240.2	23.9	421.6	149.2	1593.7	792.4	240.2	23.9	15.1
21AR226_121	129.6	9773.667	4.612	15.377	5.5	0.88844	6.1	0.10160	1.7	0.285	623.8	10.4	645.5	29.2	723.3	124.4	623.8	10.4	86.2
21AR226_122	426.6	17766.088	0.602	18.694	3.8	0.28754	4.4	0.03968	1.6	0.353	250.8	3.9	256.6	10.1	310.7	94.7	250.8	3.9	80.7
21AR226_123	29.3	1356.673	0.997	7.429	23.7	0.57057	28.0	0.03323	7.4	0.263	210.7	15.3	458.4	103.5	2022.8	481.6	210.7	15.3	10.4
21AR226_124	204.9	20511.593	10.427	17.019	4.4	0.55456	8.7	0.06956	7.1	0.819	433.5	29.9	448.0	31.5	524.0	109.4	433.5	29.9	82.7
21AR226_125	273.7	29242.322	25.441	16.932	3.0	0.73418	3.7	0.09128	1.6	0.419	563.1	8.4	559.0	16.0	543.2	73.9	563.1	8.4	103.7
21AR226_126	1555.6	80212.636	3.754	19.286	1.9	0.27242	2.5	0.03839	1.6	0.648	242.9	3.8	244.6	5.4	262.7	43.2	242.9	3.8	92.5
21AR226_127	78.4	4390.610	1.580	12.708	10.9	0.43770	11.6	0.04224	2.5	0.219	266.7	6.6	368.6	35.8	1073.6	227.1	266.7	6.6	24.8
21AR226_128	150.0	1868.273	1.977	9.660	25.6	0.14074	26.6	0.01067	4.3	0.160	68.4	2.9	133.7	33.4	1542.2	496.5	68.4	2.9	4.4
21AR226_129	262.4	22919.171	2.242	17.031	3.5	0.57583	4.3	0.07213	1.7	0.397	449.0	7.4	461.8	15.9	527.0	86.1	449.0	7.4	85.2
21AR226_130	122.2	4666.384	0.965	15.966	7.3	0.32030	8.0	0.03915	2.4	0.297	247.6	5.8	282.1	19.8	580.4	166.7	247.6	5.8	42.7
21AR226_131	265.6	4944.743	1.853	14.179	12.4	0.15156	13.7	0.01631	2.3	0.166	104.3	2.3	143.3	18.3	851.2	280.4	104.3	2.3	12.3
21AR226_132	309.5	3706.621	1.672	14.747	3.8	1.06952	10.8	0.12173	1.4	0.129	740.5	9.8	738.5	56.9	733.4	228.0	740.5	9.8	101.0
21AR226_133	36.9	443.532	2.473	4.656	59.4	0.23909	63.6	0.00930	17.9	0.282	59.7	10.7	217.7	124.8	2712.4	1034.3	59.7	10.7	2.2
21AR226_134	446.3	4948.745	3.711	17.820	3.5	0.32012	4.0	0.04387	1.3	0.325	276.8	3.6	282.0	9.9	326.5	86.4	276.8	3.6	84.8
21AR226_135	93.4	3207.332	1.581	12.610	14.1	0.23080	16.3	0.02246	5.7	0.351	143.2	8.1	210.9	31.0	1056.8	307.3	143.2	8.1	13.5
21AR226_136	346.9	68813.627	26.226	13.342	1.6	1.74441	2.3	0.17002	1.7	0.720	1012.2	15.6	1025.1	14.9	1053.6	32.4	1053.6	32.4	96.1
21AR226_137	146.8	3031.919	2.023	11.317	12.8	0.17735	16.6	0.01543	4.2	0.251	98.7	4.1	165.8	25.4	1278.2	313.8	98.7	4.1	7.7
21AR226_138	341.4	45404.789	2.205	16.961	3.4	0.68505	4.2	0.08504	2.4	0.569	526.1	12.1	529.8	17.4	546.7	75.6	526.1	12.1	96.2
21AR226_139	114.7	3677.369	1.447	12.622	15.6	0.24673	16.5	0.02389	3.7	0.226	152.2	5.6	223.9	33.1	1067.2	323.4	152.2	5.6	14.3
21AR226_140	273.4	21408.129	2.247	17.496	4.0	0.46553	4.5	0.06014	1.8	0.414	376.5	6.7	388.1	14.4	459.1	90.2	376.5	6.7	82.0
21AR226_141	81.3	6336.065	1.162	12.767	8.9	0.70190	9.2	0.06730	1.8	0.194	419.9	7.3	539.9	38.7	1086.5	182.0	419.9	7.3	38.6
21AR226_142	539.7	7106.972	2.127	15.660	7.5	0.08606	8.6	0.01016	3.6	0.416	65.1	2.3	83.8	6.9	656.2	167.8	65.1	2.3	9.9
21AR226_143	60.6	13698.766	7.585	12.794	5.1	1.82338	5.8	0.17271	1.9	0.330	1027.0	18.3	1053.9	38.2	1110.8	109.9	1110.8	109.9	92.5
21AR226_144	96.3	5436.540	0.510	14.739	9.0	0.32248	10.2	0.03621	2.3	0.225	229.3	5.2	283.8	25.3	761.8	210.3	229.3	5.2	30.1
21AR226_145	92.0	33938.462	1.183	9.195	1.8	4.57179	2.4	0.30875	1.6	0.650	1734.6	24.2	1744.1	20.4	1756.4	34.0	1756.4	34.0	98.8
21AR226_146	365.2	28647.573	2.199	13.942	5.5	0.89175	8.7	0.09126	6.7	0.765	563.0	36.1	647.3	41.8	954.6	115.1	563.0	36.1	59.0
21AR226_147	54.9	5425.296	0.366	13.418	13.0	0.62656	13.9	0.06353	2.8	0.200	397.0	10.7	493.9	54.2	973.6	277.3	397.0	10.7	40.8
21AR226_148	391.9	25107.751	5.418	18.397	3.2	0.35198	4.3	0.04771	2.1	0.495	300.5	6.2	306.2	11.3	351.3	83.8	300.5	6.2	85.5
21AR226_149	143.6	7531.973	0.374	15.305	8.2	0.34472	9.3	0.03962	1.8	0.197	250.5	4.5	300.7	24.2	712.7	194.1	250.5	4.5	35.1
21AR226_150	179.4	14762.373	8.868	17.278	4.9	0.46411	5.2	0.05970	1.6	0.303	373.8	5.8	387.1	16.8	468.4	110.3	373.8	5.8	79.8
21AR226_151	137.1	23503.702	3.003	12.055	2.3	2.14420	3.1	0.19023	1.5	0.501	1122.6	15.9	1163.2	21.3	1240.4	52.2	1240.4	52.2	90.5
21AR226_152	793.3	29123.744	2.050	19.017	3.2	0.20715	3.8	0.02906	1.6	0.430	184.7	3.0	191.2	6.6	273.2	78.4	184.7	3.0	67.6
21AR226_153	93.5	9869.416	3.054	15.345	5.5	0.88241	6.4	0.10120	2.1	0.323	621.4	12.2	642.3	30.4	717.3	128.2	621.4	12.2	86.6
21AR226_154	35.8	3668.426	0.497	11.415	19.7	0.71324	21.0	0.06230	3.8	0.180	389.6	14.3	546.7	88.7	1270.7	403.6	389.6	14.3	30.7
21AR226_155	104.5	8697.245	2.421	17.115	6.9	0.46478	7.7	0.05985	2.1	0.277	374.7	7.7	387.6	24.7	466.1	163.3	374.7	7.7	80.4
21AR226_156	231.3	5017.141	4.481	14.332	4.4	0.62408	5.1	0.06817	1.6	0.314	425.1	6.5	492.4	19.7	820.0	100.3	425.1	6.5	51.8
21AR226_157	167.6	13112.738	0.606	16.494	5.1	0.52619	6.0	0.06435	2.7	0.454	402.0	10.6	429.3	21.0	579.4	116.0	402.0	10.6	69.4
21AR226_158	352.1	3415.919	2.965	12.827	2.6	1.48108	5.9	0.14608	2.1	0.352	878.9	17.0	922.7	35.7	1029.6	111.4	1029.6	111.4	85.4
21AR226_159	98.8	8621.229	0.862	15.570	7.5	0.61154	8.9	0.07116	2.0	0.226	443.1	8.6	484.5	34.2	686.3	184.8	443.1	8.6	64.6
21AR226_160	517.6	5536.830	2.543	12.389	2.2	0.64585	4.3	0.06014	2.2	0.521	376.5	8.2	505.9	17.1	1144.7	72.9	376.5	8.2	32.9
21AR226_161	660.2	33549.527	0.705	19.102	2.8	0.26983	3.5	0.03780	1.6	0.454	239.2	3.7	242.6	7.6	276.2	71.8	239.2	3.7	86.6
21AR226_162	291.4	69749.461	8.215	13.481	1.6	1.66922	2.4	0.16368	1.7	0.706	977.2	15.2	996.9	15.1	1041.3	34.0	1041.3	34.0	93.8
21AR226_163	469.7	13917.133	10.635	17.980	2.9	0.38111	5.2	0.05066	2.2	0.432	318.6	7.0	327.9	14.5	395.2	104.6	318.6	7.0	80.6

21AR226_164	51.4	3005.452	1.252	11.895	19.5	0.50121	22.4	0.04593	2.9	0.129	289.5	8.2	412.5	76.0	1176.6	441.1	289.5	8.2	24.6
21AR226_165	177.2	27258.914	1.814	15.642	2.5	0.95740	3.2	0.10983	1.4	0.442	671.7	9.0	681.9	15.9	716.7	60.8	671.7	9.0	93.7
21AR226_166	167.9	39268.474	21.384	12.491	2.1	2.07631	3.0	0.18947	2.1	0.702	1118.5	22.0	1141.0	20.9	1184.9	43.0	1184.9	43.0	94.4
21AR226_167	495.9	1932.636	0.319	16.935	4.5	0.18459	5.5	0.02607	2.1	0.371	165.9	3.4	172.0	8.7	257.4	118.0	165.9	3.4	64.5
21AR226_168	411.4	17672.108	1.313	18.699	4.0	0.30949	4.4	0.04262	1.6	0.373	269.1	4.3	273.8	10.6	315.4	93.3	269.1	4.3	85.3
21AR226_169	216.6	5016.128	2.047	16.503	7.5	0.33092	11.7	0.04159	1.8	0.154	262.7	4.6	290.3	29.5	519.8	253.5	262.7	4.6	50.5
21AR226_170	93.7	9109.239	1.362	15.385	8.0	0.70009	8.8	0.08015	2.2	0.246	497.0	10.3	538.8	36.8	720.8	180.9	497.0	10.3	69.0
21AR226_171	291.6	13636.495	1.488	18.135	3.7	0.28161	4.7	0.03781	2.0	0.416	239.3	4.6	251.9	10.5	372.5	96.1	239.3	4.6	64.2
21AR226_172	172.2	15885.126	2.509	17.815	4.2	0.47309	4.9	0.06213	2.2	0.437	388.5	8.1	393.3	16.0	422.5	98.7	388.5	8.1	92.0
21AR226_173	216.5	40015.182	2.409	12.223	1.9	2.02222	2.6	0.18024	1.7	0.647	1068.3	16.3	1123.0	17.3	1231.3	38.2	1231.3	38.2	86.8
21AR226_174	283.3	15101.102	1.454	17.086	5.6	0.35362	6.6	0.04460	2.8	0.421	281.3	7.6	307.4	17.4	511.7	130.9	281.3	7.6	55.0
21AR226_175	286.5	37391.172	2.255	14.267	1.9	1.42126	2.7	0.14803	1.9	0.693	889.9	15.8	897.9	16.3	918.5	40.6	918.5	40.6	96.9
21AR226_176	171.5	7827.037	1.178	16.542	7.1	0.26600	7.6	0.03296	2.1	0.282	209.0	4.4	239.5	16.2	550.8	158.8	209.0	4.4	38.0
21AR226_177	446.8	1646.477	1.085	9.700	21.5	0.04129	22.3	0.00317	4.8	0.215	20.4	1.0	41.1	9.0	1518.9	411.6	20.4	1.0	1.3
21AR226_178	151.7	7096.954	1.865	16.115	7.8	0.33547	9.7	0.04061	2.3	0.233	256.6	5.7	293.7	24.9	601.2	205.3	256.6	5.7	42.7
21AR226_179	290.2	45818.856	2.634	15.054	1.6	1.12431	2.4	0.12354	1.7	0.731	750.9	12.3	765.0	12.7	807.4	33.9	750.9	12.3	93.0
21AR226_180	83.5	5057.519	1.460	14.544	10.5	0.42864	11.4	0.04728	2.8	0.243	297.8	8.1	362.2	34.7	799.6	231.8	297.8	8.1	37.2
21AR226_181	229.3	768.209	0.892	3.955	42.5	0.06586	46.1	0.00201	13.9	0.302	12.9	1.8	64.8	28.9	3105.3	709.8	12.9	1.8	0.4
21AR226_182	108.7	606.918	5.503	10.575	10.3	0.56308	37.8	0.05765	16.7	0.441	361.3	58.6	453.5	138.5	953.8	698.5	361.3	58.6	37.9
21AR226_183	232.3	509.559	1.668	4.870	69.5	0.05064	74.5	0.00203	17.3	0.232	13.1	2.3	50.2	36.5	2661.3	1248.5	13.1	2.3	0.5
21AR226_184	297.6	21824.344	3.020	13.735	1.6	1.50812	2.6	0.15192	1.6	0.624	911.7	13.7	933.7	15.7	986.8	41.0	986.8	41.0	92.4
21AR226_185	179.3	8545.347	5.328	16.496	6.7	0.31824	8.0	0.03927	1.8	0.221	248.3	4.3	280.5	19.6	559.8	170.1	248.3	4.3	44.4
21AR226_186	390.7	317.277	1.370	6.487	35.6	0.02291	45.1	0.00150	13.2	0.294	9.6	1.3	23.0	10.3	1818.1	792.1	9.6	1.3	0.5
21AR226_187	1325.0	18093.333	233.778	18.817	1.7	0.30968	2.5	0.04302	1.8	0.705	271.5	4.8	273.9	6.1	295.5	41.2	271.5	4.8	91.9
21AR226_188	881.2	1041.470	0.469	6.998	36.3	0.01533	39.0	0.00086	10.8	0.277	5.5	0.6	15.4	6.0	2094.3	664.4	5.5	0.6	0.3
21AR226_189	111.1	7650.155	1.102	15.983	7.6	0.51044	8.1	0.06123	2.3	0.286	383.1	8.6	418.7	27.7	620.9	166.7	383.1	8.6	61.7
21AR226_190	306.2	20940.687	1.054	17.784	3.2	0.54558	4.4	0.07151	2.8	0.628	445.2	12.0	442.1	15.9	426.8	77.1	445.2	12.0	104.3
21AR226_191	350.5	33478.185	0.879	17.483	3.0	0.56342	3.5	0.07225	1.7	0.482	449.7	7.4	453.8	12.9	475.3	68.4	449.7	7.4	94.6
21AR226_192	179.0	15275.450	27.334	17.036	4.1	0.54664	5.0	0.06886	2.0	0.404	429.3	8.3	442.8	17.8	514.6	99.8	429.3	8.3	83.4
21AR226_193	165.5	14654.998	2.066	16.562	5.0	0.58646	5.5	0.07179	2.1	0.387	446.9	9.2	468.6	20.6	577.3	110.1	446.9	9.2	77.4
21AR226_194	1277.4	1301.631	1.179	13.335	14.5	0.02900	27.3	0.00330	2.8	0.102	21.2	0.6	29.0	7.8	733.6	578.6	21.2	0.6	2.9
21AR226_195	476.4	1806.051	2.923	14.614	3.6	0.47819	8.5	0.05755	7.0	0.833	360.7	24.7	396.8	27.8	614.0	101.0	360.7	24.7	58.7
21AR226_196	188.9	10334.773	1.050	15.678	8.5	0.33403	9.8	0.03892	1.9	0.192	246.2	4.5	292.6	24.8	683.4	204.7	246.2	4.5	36.0
21AR226_197	96.6	6133.568	0.963	14.012	11.0	0.37045	11.8	0.03901	2.7	0.233	246.7	6.7	320.0	32.3	895.6	236.6	246.7	6.7	27.5
21AR226_198	144.3	24842.411	9.500	13.774	2.6	1.52396	4.1	0.15378	2.8	0.675	922.1	24.0	940.1	25.3	983.3	62.0	983.3	62.0	93.8
**21AR228**																			
21AR228_1	65.7	6318.212	0.813	8.578	2.2	5.00410	4.2	0.31648	2.2	0.528	1772.5	34.5	1820.0	35.7	1875.6	64.6	1875.6	64.6	94.5
21AR228_2	95.9	6291.008	1.551	12.187	2.9	2.20911	4.0	0.20033	2.0	0.498	1177.1	21.6	1183.9	28.3	1197.3	69.2	1197.3	69.2	98.3
21AR228_3	741.9	3050.497	1.154	16.784	6.5	0.09061	12.0	0.01196	1.6	0.130	76.6	1.2	88.1	10.1	411.7	265.7	76.6	1.2	18.6
21AR228_4	341.1	6331.944	1.642	17.981	3.5	0.45354	8.1	0.06138	3.9	0.489	384.0	14.7	379.8	25.6	354.7	159.0	384.0	14.7	108.3
21AR228_5	213.5	4482.984	2.910	17.406	4.3	0.46226	10.8	0.06154	1.6	0.147	385.0	5.9	385.8	34.5	391.9	238.8	385.0	5.9	98.2
21AR228_6	37.3	162.616	2.810	6.832	30.0	0.09465	147.8	0.01206	14.6	0.098	77.3	11.2	91.8	129.9	490.1		77.3	11.2	15.8
21AR228_7	45.0	811.797	0.644	12.577	13.3	0.53466	26.5	0.06252	2.7	0.101	390.9	10.2	434.9	93.7	675.9	565.4	390.9	10.2	57.8
21AR228_8	235.5	717.936	2.304	11.753	14.9	0.08692	37.9	0.00962	3.4	0.089	61.7	2.1	84.6	30.8	792.5	798.9	61.7	2.1	7.8
21AR228_9	241.4	2578.958	1.119	17.248	6.6	0.27024	12.3	0.03726	1.5	0.122	235.8	3.5	242.9	26.6	312.6	278.0	235.8	3.5	75.4
21AR228_10	96.2	1065.707	2.574	14.648	9.6	0.27134	43.7	0.03586	2.1	0.049	227.1	4.8	243.8	94.7	408.2		227.1	4.8	55.6
21AR228_11	50.5	5869.753	1.605	8.713	2.5	4.97999	4.0	0.31942	1.8	0.454	1786.9	28.6	1815.9	34.1	1850.2	65.0	1850.2	65.0	96.6
21AR228_12	35.9	1912.016	2.146	12.935	6.9	1.63324	14.3	0.16815	1.5	0.107	1001.9	14.1	983.1	89.8	942.3	290.6	942.3	290.6	106.3
21AR228_13	122.5	1589.883	1.632	16.043	8.6	0.27177	24.6	0.03689	2.3	0.093	233.5	5.3	244.1	53.5	348.4	556.5	233.5	5.3	67.0
21AR228_14	239.3	29876.878	4.437	5.903	1.2	9.57333	1.8	0.40848	1.3	0.727	2208.0	24.6	2394.6	16.6	2558.1	20.8	2558.1	20.8	86.3
21AR228_15	131.4	6401.032	2.227	13.311	2.4	1.69143	4.7	0.16730	1.9	0.406	997.2	17.5	1005.3	29.8	1023.8	86.2	1023.8	86.2	97.4
21AR228_16	155.2	2462.093	5.969	17.429	4.9	0.38854	8.2	0.05448	1.7	0.204	342.0	5.6	333.3	23.3	274.1	184.3	342.0	5.6	124.8
21AR228_17	95.8	1218.513	1.575	16.075	7.6	0.30760	30.1	0.04428	2.7	0.091	279.3	7.5	272.3	72.0	213.9		279.3	7.5	130.5
21AR228_18	307.5	3364.686	3.663	18.533	3.5	0.27364	11.8	0.03990	1.6	0.133	252.2	3.9	245.6	25.8	183.6	273.1	252.2	3.9	137.4
21AR228_19	328.1	2188.260	2.250	17.425	6.3	0.12899	26.4	0.01841	2.3	0.089	117.6	2.7	123.2	30.7	233.7		117.6	2.7	50.3
21AR228_20	306.2	2840.094	1.738	17.507	3.4	0.42355	19.8	0.05894	1.4	0.070	369.2	5.0	358.6	59.7	291.7	451.1	369.2	5.0	126.6
21AR228_21	218.8	219.561	2.187	15.373	8.9	-0.03572	1906.2	0.03935	2.3	0.001	248.8	5.6	NA	-749.1	NA		248.8	5.6	
21AR228_22	121.8	1305.702	1.631	16.491	5.1	0.53858	37.0	0.07862	1.9	0.052	487.9	9.1	437.5	131.8	181.2		487.9	9.1	269.3
21AR228_23	459.0	5222.091	1.879	18.780	3.0	0.26054	8.7	0.03747	1.9	0.216	237.2	4.4	235.1	18.3	215.7	197.2	237.2	4.4	110.0
21AR228_24	291.1	15942.762	3.769	13.277	1.9	1.53031	4.4	0.14872	3.3	0.755	893.8	27.8	942.6	27.1	1059.4	58.3	1059.4	58.3	84.4
21AR228_25	308.6	2797.960	4.321	18.484	3.5	0.28008	7.7	0.04148	2.4	0.308	262.0	6.1	250.7	17.1	147.6	171.8	262.0	6.1	177.5
21AR228_26	322.9	1879.850	1.723	17.851	3.7	0.29010	12.9	0.04355	1.5	0.116	274.8	4.0	258.6	29.3	115.9		274.8	4.0	237.1
21AR228_27	97.1	1129.853	1.456	14.699	9.2	0.29578	38.8	0.03887	2.1	0.053	245.8	5.0	263.1	90.1	420.8	872.9	245.8	5.0	58.4
21AR228_28	89.1	1278.295	2.785	15.044	10.4	0.32612	19.0	0.04301	3.3	0.174	271.4	8.8	286.6	47.5	413.1	419.9	271.4	8.8	65.7
21AR228_29	29.4	2110.082	2.959	12.204	8.2	1.95690	13.1	0.18889	1.8	0.139	1115.4	18.5	1100.8	87.8	1073.1	260.0	1073.1	260.0	103.9
21AR228_30	410.1	5920.878	3.363	17.161	2.4	0.59775	7.2	0.07776	1.6	0.226	482.7	7.5	475.8	27.2	443.6	155.3	482.7	7.5	108.8
21AR228_31	168.0	2395.451	1.801	15.978	8.5	0.30440	12.8	0.03886	2.1	0.160	245.8	5.0	269.8	30.4	485.0	279.5	245.8	5.0	50.7
21AR228_32	97.8	1393.786	2.075	15.183	9.7	0.31812	25.3	0.04169	1.9	0.075	263.3	4.9	280.5	62.0	426.9	564.6	263.3	4.9	61.7
21AR228_33	347.2	12044.114	5.186	16.284	1.9	0.81116	2.8	0.09782	1.4	0.499	601.6	7.9	603.1	12.6	609.7	51.9	601.6	7.9	98.7
21AR228_34	90.1	2555.437	1.801	15.861	6.6	0.65200	13.2	0.08256	2.0	0.150	511.4	9.7	509.7	52.7	503.0	286.6	511.4	9.7	101.7
21AR228_35	40.6	237.055	5.611	11.113	6.5	0.49841	179.3	0.13648	7.1	0.039	824.8	54.6	410.6	624.6	NA		824.8	54.6	
21AR228_36	300.2	6184.791	1.709	17.905	3.1	0.44610	9.0	0.06059	2.0	0.217	379.2	7.2	374.5	28.2	346.9	199.1	379.2	7.2	109.3
21AR228_37	65.7	3974.262	2.281	12.854	4.0	1.89518	7.1	0.18549	2.6	0.361	1096.9	26.0	1079.4	47.4	1045.1	134.1	1045.1	134.1	105.0
21AR228_38	190.6	447.732	1.279	16.181	7.6	0.10526	110.7	0.02726	2.1	0.019	173.4	3.5	101.6	107.1	NA		173.4	3.5	
21AR228_39	144.9	1744.873	1.232	15.114	9.6	0.31492	20.1	0.03945	1.9	0.094	249.4	4.6	278.0	48.9	527.0	439.3	249.4	4.6	47.3
21AR228_40	150.2	1696.979	1.352	15.300	8.3	0.31242	14.0	0.03970	2.2	0.155	250.9	5.3	276.1	33.8	495.6	304.6	250.9	5.3	50.6
21AR228_41	192.2	898.984	2.227	15.923	9.7	0.10308	44.3	0.01607	2.5	0.056	102.8	2.5	99.6	42.1	25.4		102.8	2.5	404.7
21AR228_42	195.9	9108.131	3.873	15.357	2.2	1.04830	4.5	0.11927	2.7	0.596	726.4	18.4	728.0	23.3	734.1	76.3	726.4	18.4	99.0
21AR228_43	227.8	5043.529	1.597	18.359	4.2	0.42445	10.3	0.05950	1.5	0.141	372.6	5.3	359.2	31.2	274.7	233.8	372.6	5.3	135.7
21AR228_44	69.1	248.118	1.842	11.190	22.2	0.03703	161.7	0.00942	9.9	0.061	60.4	5.9	36.9	58.7	NA		60.4	5.9	
21AR228_45	255.6	3804.526	5.859	17.784	4.4	0.35884	15.7	0.04952	14.0	0.890	311.6	42.5	311.3	42.1	310.7	162.6	311.6	42.5	100.3
21AR228_46	86.0	389.746	1.710	10.825	17.2	0.13740	107.9	0.01820	4.1	0.038	116.2	4.8	130.7	132.6	403.8		116.2	4.8	28.8
21AR228_47	88.2	833.709	3.640	11.462	13.9	0.26189	49.5	0.02695	3.5	0.071	171.4	6.0	236.2	104.4	943.4	1026.7	171.4	6.0	18.2
21AR228_48	203.4	2515.203	1.798	16.948	6.2	0.30769	11.4	0.04170	1.5	0.135	263.4	4.0	272.4	27.3	351.5	256.4	263.4	4.0	74.9
21AR228_49	296.1	6963.482	2.539	17.789	3.0	0.49627	6.3	0.06611	3.2	0.499	412.6	12.6	409.2	21.3	390.6	123.1	412.6	12.6	105.6
21AR228_50	95.7	5433.803	4.086	13.252	3.4	1.70154	6.3	0.16840	1.6	0.252	1003.3	14.7	1009.1	40.2	1022.6	123.0	1022.6	123.0	98.1
21AR228_51	240.9	5730.369	15.585	15.445	2.4	0.77399	5.9	0.08895	1.6	0.265	549.4	8.2	582.0	26.2	712.7	121.1	549.4	8.2	77.1
21AR228_52	206.2	7941.177	12.095	14.346	2.8	1.19707	6.4	0.12701	3.7	0.570	770.8	26.5	799.2	35.5	880.2	109.0	770.8	26.5	87.6
21AR228_53	261.2	4158.343	2.903	17.561	3.5	0.42717	6.2	0.05760	1.1	0.181	361.0	4.0	361.2	19.0	363.1	138.4	361.0	4.0	99.4
21AR228_54	170.5	10530.441	2.910	12.503	1.3	2.06748	3.5	0.18941	1.4	0.390	1118.2	14.0	1138.1	24.0	1177.1	63.9	1177.1	63.9	95.0
21AR228_55	62.9	4120.622	0.795	8.656	1.8	4.74994	3.7	0.30490	1.4	0.374	1715.5	21.1	1776.1	31.4	1848.8	62.8	1848.8	62.8	92.8
21AR228_56	111.4	945.409	1.767	15.428	9.6	0.37322	56.5	0.05457	1.8	0.032	342.5	6.1	322.0	156.2	177.5		342.5	6.1	192.9
21AR228_57	87.9	1667.528	3.564	15.558	8.3	0.43368	13.6	0.05612	1.8	0.135	352.0	6.3	365.8	41.9	455.1	300.1	352.0	6.3	77.4
21AR228_58	199.4	1717.259	4.356	16.211	8.6	0.19611	17.1	0.02638	1.6	0.093	167.9	2.6	181.8	28.5	368.5	384.2	167.9	2.6	45.6
21AR228_59	47.4	1395.729	8.925	13.789	7.5	0.99937	18.9	0.11494	2.2	0.117	701.4	14.7	703.5	96.1	711.2	400.3	701.4	14.7	98.6
21AR228_60	46.7	672.579	1.383	11.227	15.9	0.35446	28.3	0.03743	4.1	0.145	236.9	9.6	308.1	75.3	889.9	581.3	236.9	9.6	26.6
21AR228_61	296.0	6076.058	2.602	18.293	3.2	0.43207	9.3	0.05965	1.4	0.156	373.5	5.2	364.6	28.4	309.8	208.5	373.5	5.2	120.6
21AR228_62	199.1	4828.430	6.108	16.820	4.0	0.55369	5.2	0.07016	1.4	0.276	437.1	6.0	447.4	18.7	501.8	109.2	437.1	6.0	87.1
21AR228_63	123.4	1955.563	3.809	16.326	6.6	0.38756	17.3	0.05153	1.9	0.111	323.9	6.1	332.6	49.2	394.5	386.9	323.9	6.1	82.1
21AR228_64	1026.6	1570.916	1.979	18.729	2.1	0.21866	24.7	0.03556	1.4	0.059	225.2	3.2	200.8	45.1	NA		225.2	3.2	
21AR228_65	52.6	607.441	1.109	12.284	14.1	0.31098	80.5	0.03857	3.1	0.038	244.0	7.4	274.9	194.6	548.5		244.0	7.4	44.5
21AR228_66	147.3	1917.708	3.899	16.621	7.1	0.30174	9.8	0.04093	1.6	0.168	258.6	4.2	267.8	23.0	349.4	218.0	258.6	4.2	74.0
21AR228_67	222.9	985.567	0.500	13.441	13.5	0.10201	43.1	0.01219	2.9	0.067	78.1	2.2	98.6	40.5	629.8	935.3	78.1	2.2	12.4
21AR228_68	16.3	253.791	1.855	8.158	23.0	0.44780	102.0	0.04844	9.2	0.090	304.9	27.3	375.7	323.0	840.1		304.9	27.3	36.3
21AR228_69	194.5	776.994	2.750	11.657	22.0	0.09414	45.1	0.01001	6.4	0.143	64.2	4.1	91.3	39.4	876.5	935.1	64.2	4.1	7.3
21AR228_70	164.0	2296.351	1.747	16.349	4.1	0.55508	19.9	0.07349	2.4	0.121	457.1	10.7	448.3	72.1	404.4	442.6	457.1	10.7	113.0
21AR228_71	193.0	4534.355	4.059	17.034	3.8	0.55296	12.3	0.07124	1.9	0.150	443.6	7.9	446.9	44.5	465.1	269.8	443.6	7.9	95.4
21AR228_72	558.6	61503.995	11.956	8.165	1.2	5.59811	2.0	0.33034	1.7	0.811	1840.0	26.6	1915.8	17.6	1999.6	21.2	1999.6	21.2	92.0
21AR228_73	396.7	15060.507	12.074	13.560	1.5	1.58108	3.3	0.15761	1.6	0.493	943.5	14.3	962.8	20.5	1008.1	58.3	1008.1	58.3	93.6
21AR228_74	70.4	1225.235	3.230	14.718	7.8	0.42149	30.3	0.05456	2.5	0.081	342.5	8.2	357.1	91.4	454.3	674.3	342.5	8.2	75.4
21AR228_75	92.6	2054.675	1.191	15.473	7.8	0.48329	15.0	0.06094	2.0	0.134	381.3	7.5	400.3	49.7	512.6	327.7	381.3	7.5	74.4
21AR228_76	80.5	1269.007	2.023	13.469	12.0	0.35238	17.5	0.04068	2.5	0.141	257.1	6.2	306.5	46.4	703.1	369.8	257.1	6.2	36.6
21AR228_77	93.6	1717.455	0.673	16.175	9.9	0.45103	15.2	0.06135	2.6	0.170	383.8	9.6	378.0	47.9	343.5	338.7	383.8	9.6	111.7
21AR228_78	81.9	3933.697	2.444	13.570	4.0	1.35961	6.8	0.14075	3.0	0.447	848.9	24.1	871.7	39.7	931.0	124.4	848.9	24.1	91.2
21AR228_79	727.7	3472.114	1.074	19.024	2.5	0.24681	9.6	0.03712	1.3	0.132	234.9	2.9	224.0	19.2	111.5	223.6	234.9	2.9	210.6

21AR228_80	109.2	8625.450	2.440	11.465	1.7	2.62047	2.3	0.22249	1.2	0.511	1295.0	13.7	1306.4	16.8	1326.0	37.9	1326.0	37.9	97.7
21AR228_81	294.7	4489.803	5.590	17.154	3.8	0.53946	5.4	0.07122	2.2	0.408	443.5	9.5	438.1	19.3	410.7	110.8	443.5	9.5	108.0
21AR228_82	102.7	2126.871	1.150	17.196	5.7	0.50806	9.8	0.07202	2.2	0.225	448.3	9.6	417.1	33.7	249.3	220.9	448.3	9.6	179.8
21AR228_83	332.5	4563.097	3.444	17.644	3.8	0.36303	6.8	0.04870	2.2	0.324	306.5	6.6	314.5	18.3	374.8	144.4	306.5	6.6	81.8
21AR228_84	95.2	1614.340	1.327	16.492	7.0	0.40189	19.1	0.05663	1.9	0.099	355.1	6.5	343.0	55.5	263.1	436.2	355.1	6.5	134.9
21AR228_85	274.9	1946.773	0.515	17.569	5.3	0.24710	19.5	0.03639	1.4	0.072	230.4	3.2	224.2	39.3	160.6		230.4	3.2	143.5
21AR228_86	92.4	1233.333	0.780	14.509	9.3	0.30242	18.3	0.03849	2.1	0.115	243.5	5.0	268.3	43.2	491.7	401.8	243.5	5.0	49.5
21AR228_87	342.6	2460.660	7.527	16.365	2.8	0.54115	13.6	0.07130	2.4	0.174	444.0	10.2	439.2	48.7	415.1	300.6	444.0	10.2	107.0
21AR228_88	195.1	2776.290	10.826	15.917	3.2	0.75513	10.1	0.09519	1.8	0.178	586.2	10.1	571.2	44.1	513.1	218.4	586.2	10.1	114.2
21AR228_89	288.5	3728.328	2.928	18.253	3.9	0.32169	13.9	0.04599	1.9	0.133	289.9	5.3	283.2	34.5	229.4	319.6	289.9	5.3	126.4
21AR228_90	26.0	452.959	1.938	8.914	23.4	0.68179	71.5	0.06117	3.9	0.055	382.8	14.6	527.9	296.6	1218.2	1451.4	382.8	14.6	31.4
21AR228_91	149.9	921.776	1.426	12.994	12.7	0.15723	30.5	0.01838	3.1	0.102	117.4	3.6	148.3	42.1	676.4	651.8	117.4	3.6	17.4
21AR228_92	107.8	1306.388	1.589	14.468	8.6	0.31619	33.8	0.03964	2.2	0.065	250.6	5.4	279.0	82.6	524.9	745.5	250.6	5.4	47.7
21AR228_93	31.6	6437.928	1.355	3.694	1.3	21.57276	3.1	0.58299	2.8	0.899	2960.9	65.5	3164.7	29.8	3297.2	21.1	3297.2	21.1	89.8
21AR228_94	96.8	280.744	2.748	8.061	24.0	0.09633	48.8	0.00957	6.8	0.140	61.4	4.2	93.4	43.5	1015.0	993.1	61.4	4.2	6.0
21AR228_95	174.6	712.374	1.304	13.737	9.7	0.10626	34.4	0.01476	3.2	0.094	94.4	3.0	102.5	33.5	296.0		94.4	3.0	31.9
21AR228_96	202.6	768.274	0.981	13.522	16.4	0.09172	27.3	0.01211	3.9	0.143	77.6	3.0	89.1	23.3	410.6	605.7	77.6	3.0	18.9
21AR228_97	133.6	1352.524	3.322	15.286	8.3	0.20439	27.7	0.02713	2.5	0.091	172.6	4.3	188.8	47.7	398.2	621.0	172.6	4.3	43.3
21AR228_98	19.3	210.710	0.894	7.181	30.6	0.30093	55.9	0.03055	10.6	0.189	194.0	20.2	267.1	131.4	970.6	1139.7	194.0	20.2	20.0
21AR228_99	177.0	2524.457	1.783	17.555	4.8	0.33385	14.6	0.04705	1.8	0.126	296.4	5.3	292.5	37.0	262.5	331.8	296.4	5.3	112.9
21AR228_100	173.3	2112.959	1.512	16.530	7.1	0.28459	11.0	0.03830	2.0	0.181	242.3	4.7	254.3	24.7	367.5	243.2	242.3	4.7	65.9
21AR228_101	50.7	575.871	0.443	11.579	13.9	0.49493	54.6	0.05786	2.5	0.046	362.6	8.8	408.3	184.1	676.5	1187.0	362.6	8.8	53.6
21AR228_102	196.3	3095.627	2.385	17.532	4.0	0.38865	17.6	0.05330	1.9	0.109	334.8	6.3	333.4	50.1	324.9	398.1	334.8	6.3	103.0
21AR228_103	141.5	2706.868	2.608	16.795	5.9	0.41242	20.0	0.05488	1.8	0.089	344.4	6.0	350.6	59.5	392.9	449.0	344.4	6.0	87.7
21AR228_104	129.3	1373.392	0.873	15.647	8.1	0.28195	38.7	0.03814	2.1	0.054	241.3	4.9	252.2	86.4	355.6		241.3	4.9	67.9
21AR228_105	156.1	4373.725	1.729	14.742	3.2	1.08275	8.1	0.12087	1.9	0.240	735.6	13.5	745.0	42.7	774.2	165.2	735.6	13.5	95.0
21AR228_106	98.7	4902.884	2.784	13.916	2.9	1.57193	5.8	0.16428	1.5	0.263	980.5	14.0	959.2	36.3	911.4	116.1	911.4	116.1	107.6
21AR228_107	255.2	6600.338	4.891	17.055	2.5	0.68278	3.9	0.08680	1.6	0.406	536.6	8.3	528.5	16.3	494.5	79.6	536.6	8.3	108.5
21AR228_108	101.1	2612.225	0.822	16.074	6.3	0.75241	9.8	0.09555	1.6	0.163	588.3	9.0	569.6	42.9	496.7	214.1	588.3	9.0	118.4
21AR228_109	224.8	3164.422	6.299	17.369	4.1	0.41505	10.9	0.05639	1.7	0.160	353.6	6.0	352.5	32.4	346.2	243.2	353.6	6.0	102.1
21AR228_110	469.1	1241.376	4.182	15.628	2.1	0.69590	8.5	0.09588	2.3	0.266	590.2	12.8	536.3	35.5	314.3	187.1	590.2	12.8	187.8
21AR228_111	175.0	4284.104	3.300	17.625	3.7	0.54142	6.3	0.07267	1.7	0.266	452.2	7.3	439.4	22.6	373.5	137.3	452.2	7.3	121.1
21AR228_112	294.0	5595.989	2.106	17.817	3.1	0.46121	8.2	0.06226	2.3	0.277	389.3	8.6	385.1	26.4	360.6	178.4	389.3	8.6	108.0
21AR228_113	324.7	4615.011	2.597	16.926	3.6	0.51655	6.5	0.06682	2.0	0.302	417.0	7.9	422.8	22.5	456.1	137.5	417.0	7.9	91.4
21AR228_114	279.2	4034.929	24.547	17.241	2.8	0.52412	8.4	0.06972	2.4	0.287	434.4	10.1	427.9	29.2	393.8	179.7	434.4	10.1	110.3
21AR228_115	1153.2	7819.700	3.048	19.720	2.6	0.14856	4.4	0.02201	1.6	0.361	140.3	2.2	140.6	5.8	146.6	96.5	140.3	2.2	95.7
21AR228_116	126.3	891.014	0.548	13.863	11.2	0.20988	21.2	0.02723	2.5	0.120	173.2	4.3	193.4	37.4	449.3	468.9	173.2	4.3	38.5
21AR228_117	165.3	4271.269	2.271	13.423	2.2	1.54853	6.0	0.15743	1.9	0.326	942.5	17.1	949.9	36.8	968.1	115.2	968.1	115.2	97.4
21AR228_118	132.5	2515.822	2.708	16.462	5.2	0.52048	8.7	0.06827	3.3	0.386	425.7	13.8	425.5	30.1	425.1	178.5	425.7	13.8	100.1
21AR228_119	133.3	485.410	1.049	9.170	23.0	0.13144	32.4	0.01186	3.6	0.111	76.0	2.7	125.4	38.2	1207.2	637.4	76.0	2.7	6.3
21AR228_120	66.8	1371.203	1.215	15.236	9.0	0.45988	12.7	0.06026	2.3	0.182	377.2	8.5	384.2	40.6	427.1	278.4	377.2	8.5	88.3
21AR228_121	168.0	3648.162	2.614	14.378	5.0	0.64589	10.4	0.07047	1.8	0.173	439.0	7.6	505.9	41.4	822.3	213.7	439.0	7.6	53.4
21AR228_122	165.4	3316.774	4.562	17.270	5.7	0.44061	14.0	0.05936	1.6	0.115	371.7	5.8	370.7	43.5	365.1	314.1	371.7	5.8	101.8
21AR228_123	56.1	902.540	17.360	12.637	15.1	0.47172	50.6	0.05385	2.5	0.049	338.1	8.2	392.4	165.0	727.0	1087.1	338.1	8.2	46.5
21AR228_124	45.9	137.734	1.475	5.159	33.5	0.10096	136.0	0.00789	12.7	0.093	50.6	6.4	97.7	126.8	1485.8	2977.9	50.6	6.4	3.4
21AR228_125	491.5	19678.594	21.620	13.618	1.2	1.61782	2.0	0.16073	1.4	0.686	960.8	12.2	977.1	12.5	1014.8	29.3	1014.8	29.3	94.7
21AR228_126	284.3	5143.239	7.883	18.028	3.2	0.45304	5.9	0.06219	1.5	0.251	388.9	5.6	379.4	18.7	322.8	129.8	388.9	5.6	120.5
21AR228_127	119.6	1321.442	1.477	16.197	7.5	0.25576	40.1	0.03649	2.0	0.049	231.0	4.4	231.2	83.0	234.3		231.0	4.4	98.6
21AR228_128	58.6	966.723	1.420	13.752	11.6	0.43884	31.3	0.05496	2.8	0.089	344.9	9.3	369.4	96.9	527.4	686.4	344.9	9.3	65.4
21AR228_129	370.7	3660.085	8.100	16.418	2.2	0.73093	10.4	0.09230	1.9	0.186	569.1	10.5	557.1	44.5	509.2	224.3	569.1	10.5	111.8
21AR228_130	85.5	1063.268	1.504	15.220	7.4	0.40908	52.8	0.05663	4.2	0.080	355.1	14.5	348.2	156.0	303.4		355.1	14.5	117.0
21AR228_131	194.4	2996.994	4.885	17.503	4.3	0.43967	7.5	0.06007	1.7	0.221	376.1	6.1	370.0	23.4	333.2	166.8	376.1	6.1	112.9
21AR228_132	32.2	914.905	1.551	14.119	10.4	0.63591	21.6	0.08336	3.0	0.137	516.2	14.6	499.8	85.3	426.3	478.6	516.2	14.6	121.1
21AR228_133	224.7	11548.721	1.186	12.875	1.6	1.89157	3.0	0.17737	1.7	0.577	1052.6	16.8	1078.1	19.9	1131.1	48.7	1131.1	48.7	93.1
21AR228_134	159.6	1289.213	1.031	15.675	7.8	0.27854	18.0	0.03810	2.0	0.112	241.1	4.8	249.5	39.9	330.5	407.4	241.1	4.8	73.0
21AR228_135	57.7	939.871	2.207	15.095	9.1	0.32731	35.6	0.04637	2.9	0.081	292.2	8.3	287.5	89.3	250.6		292.2	8.3	116.6
21AR228_136	313.1	2432.260	1.366	18.073	4.6	0.25562	8.9	0.03720	1.7	0.192	235.5	4.0	231.1	18.5	188.1	204.4	235.5	4.0	125.2
21AR228_137	206.4	651.858	2.516	12.035	16.1	0.08570	45.7	0.01009	4.0	0.088	64.7	2.6	83.5	36.6	662.1	987.2	64.7	2.6	9.8
21AR228_138	116.1	7876.320	1.357	10.976	1.6	2.78568	2.5	0.22454	1.2	0.490	1305.8	14.4	1351.7	18.6	1425.8	41.4	1425.8	41.4	91.6
21AR228_139	216.0	5480.864	2.059	17.476	4.2	0.44995	7.5	0.05939	1.4	0.185	371.9	5.0	377.2	23.7	411.2	165.3	371.9	5.0	90.4
21AR228_140	57.3	1585.165	1.271	14.539	7.5	0.65532	19.7	0.07912	1.8	0.091	490.9	8.5	511.7	79.3	607.0	425.6	490.9	8.5	80.9
21AR228_141	70.2	4086.341	1.434	13.870	5.0	1.42720	6.5	0.14983	1.4	0.222	900.0	12.1	900.4	38.8	902.2	130.7	902.2	130.7	99.8
21AR228_142	146.5	8576.427	2.644	12.666	2.0	1.94964	3.0	0.18132	1.3	0.435	1074.2	13.0	1098.3	20.2	1147.4	53.9	1147.4	53.9	93.6
21AR228_143	14.4	160.070	1.244	4.313	34.9	0.61798	56.1	0.02929	11.3	0.201	186.1	20.7	488.6	218.4	2380.9	956.6	186.1	20.7	7.8
21AR228_144	459.1	5046.242	1.152	18.570	4.4	0.20449	7.0	0.02887	1.7	0.240	183.5	3.0	188.9	12.0	258.8	155.8	183.5	3.0	70.9
21AR228_145	88.6	1794.702	2.902	15.209	6.5	0.47525	13.9	0.05928	1.9	0.136	371.2	6.8	394.8	45.4	536.2	301.2	371.2	6.8	69.2
21AR228_146	92.4	143.624	0.911	11.156	18.8	-0.10606	944.6	0.03481	3.5	0.004	220.6	7.5	NA	-1286.3	NA		220.6	7.5	
21AR228_147	114.9	1150.349	1.267	14.830	12.0	0.20472	21.5	0.02690	2.1	0.097	171.1	3.5	189.1	37.0	421.2	477.9	171.1	3.5	40.6
21AR228_148	111.0	4571.267	3.626	13.258	2.6	1.63847	6.3	0.16294	1.4	0.218	973.1	12.4	985.1	39.8	1012.8	124.7	1012.8	124.7	96.1
21AR228_149	137.0	5642.349	3.701	13.487	2.9	1.59988	5.9	0.16107	1.5	0.256	962.7	13.5	970.2	36.8	988.0	115.8	988.0	115.8	97.4
21AR228_150	133.8	1907.503	1.687	15.487	10.3	0.35563	15.5	0.04500	1.9	0.121	283.7	5.2	308.9	41.4	504.8	340.0	283.7	5.2	56.2
21AR228_151	90.2	532.838	1.044	9.925	17.9	0.13610	48.4	0.01325	5.7	0.118	84.9	4.8	129.6	58.9	1055.2	981.1	84.9	4.8	8.0
21AR228_152	253.3	4656.202	1.973	17.523	3.2	0.42307	6.5	0.05652	1.6	0.248	354.4	5.5	358.2	19.6	384.1	141.2	354.4	5.5	92.3
21AR228_153	182.5	2945.411	1.899	16.434	4.4	0.52000	12.0	0.06701	5.1	0.428	418.1	20.7	425.2	41.6	464.4	239.9	418.1	20.7	90.0
21AR228_154	223.7	3636.295	11.153	17.315	3.7	0.43725	14.3	0.05849	1.6	0.109	366.5	5.5	368.3	44.1	380.9	319.4	366.5	5.5	96.2
21AR228_155	98.6	294.208	1.791	7.543	33.4	0.10128	93.5	0.00860	6.6	0.071	55.2	3.6	98.0	87.4	1326.4	1917.3	55.2	3.6	4.2
21AR228_156	26.1	392.077	0.684	9.286	17.4	0.40465	85.9	0.04087	5.6	0.065	258.2	14.2	345.0	252.5	981.4	1830.4	258.2	14.2	26.3
21AR228_157	93.2	2153.106	1.601	16.102	4.9	0.61494	15.3	0.07954	1.6	0.107	493.4	7.7	486.7	59.0	456.1	337.0	493.4	7.7	108.2
21AR228_158	108.8	1728.436	2.328	14.688	8.9	0.27905	25.3	0.03365	2.3	0.090	213.4	4.8	249.9	56.1	609.7	546.7	213.4	4.8	35.0
21AR228_159	64.7	1030.110	2.128	9.180	2.3	3.91636	22.3	0.29451	2.8	0.125	1664.0	40.8	1617.1	180.5	1557.3	415.7	1557.3	415.7	106.9
21AR228_160	333.6	11170.788	3.854	15.733	2.0	0.89619	3.2	0.10340	1.7	0.540	634.3	10.3	649.7	15.1	704.5	56.4	634.3	10.3	90.0
21AR228_161	242.3	907.605	3.767	14.207	14.7	0.09243	48.2	0.01224	2.9	0.061	78.4	2.3	89.8	41.4	404.0		78.4	2.3	19.4
21AR228_162	78.5	1436.677	0.661	15.195	7.6	0.48156	30.8	0.06207	2.1	0.068	388.2	7.9	399.1	101.6	463.9	683.6	388.2	7.9	83.7
21AR228_163	225.0	4461.487	2.633	17.866	4.4	0.45509	11.3	0.06205	1.5	0.136	388.1	5.7	380.8	35.7	338.1	252.6	388.1	5.7	114.8
21AR228_164	952.6	8028.834	3.816	19.325	2.3	0.24996	4.4	0.03598	1.6	0.358	227.8	3.5	226.5	8.9	214.1	95.0	227.8	3.5	106.4
21AR228_165	99.1	5382.570	3.305	12.049	3.0	2.04703	5.6	0.18297	2.2	0.397	1083.2	22.2	1131.3	38.2	1225.7	100.8	1225.7	100.8	88.4
21AR228_166	220.7	5072.655	12.145	17.654	3.8	0.51212	10.4	0.06818	2.3	0.219	425.2	9.3	419.9	35.6	391.8	226.8	425.2	9.3	108.5
21AR228_167	151.6	2808.609	37.793	15.903	6.5	0.47524	9.5	0.05956	1.7	0.180	372.9	6.2	394.8	31.2	525.9	205.5	372.9	6.2	70.9
21AR228_168	255.8	5446.454	14.435	17.012	3.7	0.56062	8.3	0.07191	2.1	0.248	447.7	8.9	451.9	30.2	474.6	177.6	447.7	8.9	94.3
21AR228_169	360.0	4001.512	20.964	17.970	4.0	0.29466	14.1	0.04087	2.4	0.170	258.2	6.1	262.2	32.5	299.1	316.6	258.2	6.1	86.3
21AR228_170	388.3	4573.378	1.369	18.663	3.3	0.26493	10.1	0.03792	1.3	0.128	239.9	3.0	238.6	21.4	226.7	230.9	239.9	3.0	105.8
21AR228_171	9.2	276.295	1.015	7.511	24.7	1.07703	100.4	0.09451	6.4	0.064	582.2	35.6	742.2	540.9	1261.7	2098.0	1261.7	2098.0	46.1
21AR228_172	253.5	434.699	2.999	15.958	4.1	0.21294	15.7	0.05487	2.2	0.138	344.3	7.3	196.0	28.1	NA		344.3	7.3	
21AR228_173	164.8	2428.153	1.075	17.419	4.7	0.44611	13.3	0.06251	1.9	0.144	390.9	7.3	374.5	41.7	275.8	302.1	390.9	7.3	141.7
21AR228_174	84.5	1099.143	2.173	16.845	7.5	0.35691	41.7	0.05592	2.2	0.053	350.8	7.5	309.9	111.4	13.7		350.8	7.5	2561.1
21AR228_175	123.2	9854.292	2.890	9.275	1.4	4.44892	3.2	0.29935	2.1	0.651	1688.1	31.1	1721.5	26.7	1763.1	44.7	1763.1	44.7	95.7
21AR228_176	53.0	3481.617	2.481	11.560	3.1	2.62940	5.6	0.22953	1.4	0.258	1332.0	17.4	1308.9	41.2	1272.0	105.6	1272.0	105.6	104.7
21AR228_177	187.1	2093.475	0.827	16.794	6.8	0.28312	12.0	0.03878	1.7	0.139	245.3	4.0	253.1	27.0	327.3	270.9	245.3	4.0	74.9
21AR228_178	279.9	6398.773	3.488	15.696	2.4	0.67297	6.4	0.07882	1.4	0.222	489.1	6.7	522.5	26.2	672.4	133.9	489.1	6.7	72.7
21AR228_179	181.2	1983.577	1.779	16.865	6.7	0.27716	10.3	0.03851	2.0	0.199	243.6	4.9	248.4	22.6	294.9	229.7	243.6	4.9	82.6
21AR228_180	98.9	2418.330	1.424	16.514	5.1	0.55577	13.6	0.07352	2.6	0.191	457.3	11.4	448.8	49.2	406.2	298.1	457.3	11.4	112.6
21AR228_181	262.4	15511.562	1.972	12.025	1.9	2.22107	3.1	0.19476	2.4	0.761	1147.1	25.0	1187.7	21.9	1263.2	39.6	1263.2	39.6	90.8
21AR228_182	42.3	1205.320	2.103	13.920	9.8	0.66977	19.0	0.08075	2.1	0.113	500.6	10.4	520.6	77.6	610.0	409.7	500.6	10.4	82.1
21AR228_183	149.6	1696.895	1.501	15.735	12.9	0.25333	16.4	0.03338	2.8	0.173	211.7	5.9	229.3	33.6	415.1	360.8	211.7	5.9	51.0
21AR228_184	117.5	3702.616	6.681	15.654	4.2	0.70322	9.5	0.08499	2.4	0.250	525.9	12.0	540.7	39.9	604.8	199.4	525.9	12.0	87.0
21AR228_185	55.5	1504.488	0.537	15.368	7.6	0.65502	24.5	0.08571	1.8	0.073	530.1	9.2	511.6	98.7	430.3	547.3	530.1	9.2	123.2
21AR228_186	411.5	4201.272	15.703	18.172	2.7	0.33700	10.1	0.04687	2.1	0.210	295.3	6.2	294.9	26.0	292.7	226.7	295.3	6.2	100.9
21AR228_187	107.9	7692.710	2.679	12.356	1.9	2.16719	2.8	0.19866	1.6	0.557	1168.1	16.9	1170.6	19.7	1176.0	46.7	1176.0	46.7	99.3
21AR228_188	123.2	1032.479	1.670	14.638	8.0	0.29208	40.2	0.03913	2.0	0.050	247.4	4.9	260.2	92.2	377.7		247.4	4.9	65.5
21AR228_189	330.0	4596.553	2.659	18.386	3.8	0.30629	9.4	0.04341	1.7	0.181	273.9	4.5	271.3	22.3	249.8	211.8	273.9	4.5	109.7
21AR228_190	384.9	1469.206	0.729	17.144	7.5	0.10048	34.7	0.01510	1.7	0.049	96.6	1.6	97.2	32.2	113.7		96.6	1.6	85.0
21AR228_191	112.7	1370.328	1.773	15.143	9.6	0.29510	13.9	0.03871	2.2	0.156	244.8	5.2	262.6	32.3	425.1	307.7	244.8	5.2	57.6
21AR228_192	71.1	998.040	1.428	12.791	12.5	0.35040	15.2	0.03976	2.7	0.178	251.3	6.6	305.0	40.0	739.8	316.3	251.3	6.6	34.0
21AR228_193	211.9	5138.984	1.623	17.353	3.6	0.61143	9.1	0.08068	1.9	0.213	500.2	9.3	484.5	34.9	411.8	198.1	500.2	9.3	121.5
21AR228_194	64.4	683.247	3.019	14.047	9.4	0.36976	26.2	0.05379	2.2	0.085	337.7	7.3	319.5	71.8	189.4		337.7	7.3	178.3
21AR228_195	316.3	5140.797	2.058	18.148	3.9	0.30863	6.1	0.04270	1.9	0.316	269.5	5.1	273.1	14.5	304.9	131.2	269.5	5.1	88.4

21AR228_196	80.5	474.516	2.408	13.041	16.7	0.26159	77.6	0.04116	3.3	0.042	260.0	8.3	235.9	163.7	3.5		260.0	8.3	7402.9
21AR228_197	105.8	2136.533	1.960	16.662	6.1	0.51790	9.6	0.07076	1.5	0.156	440.7	6.4	423.7	33.2	333.4	214.9	440.7	6.4	132.2
21AR228_198	431.2	1442.972	1.933	17.029	9.1	0.07264	24.3	0.01088	1.8	0.075	69.8	1.3	71.2	16.7	120.4		69.8	1.3	58.0
21AR228_199	236.8	4518.392	2.150	18.031	4.1	0.44778	7.2	0.06231	3.1	0.435	389.7	11.8	375.7	22.5	291.7	147.2	389.7	11.8	133.6
21AR228_200	34.8	627.213	1.698	12.686	12.2	0.49178	22.7	0.06428	2.6	0.116	401.6	10.3	406.1	76.0	432.9	503.7	401.6	10.3	92.8
21AR228_201	128.5	1801.527	2.300	15.042	7.6	0.37631	15.0	0.04681	2.0	0.130	294.9	5.6	324.3	41.7	542.0	325.8	294.9	5.6	54.4
21AR228_202	114.0	1321.854	5.890	17.119	6.6	0.27972	19.9	0.04301	2.0	0.100	271.4	5.3	250.4	44.2	59.1		271.4	5.3	459.1
21AR228_203	97.0	6980.196	0.969	8.786	1.8	4.73456	3.4	0.30716	1.6	0.462	1726.7	24.1	1773.4	28.9	1829.5	55.4	1829.5	55.4	94.4
21AR228_204	196.4	4047.441	2.329	17.345	3.8	0.59310	13.9	0.07963	1.7	0.121	493.9	8.0	472.8	52.6	372.9	310.9	493.9	8.0	132.5
21AR228_205	353.3	9662.160	29.706	17.300	2.3	0.63697	5.7	0.08210	1.6	0.271	508.7	7.6	500.4	22.6	463.9	122.1	508.7	7.6	109.6
21AR228_206	289.7	3238.898	1.895	17.842	4.0	0.42396	17.4	0.05971	1.6	0.091	373.9	5.8	358.9	52.5	264.2	397.8	373.9	5.8	141.5
21AR228_207	45.3	405.536	0.810	8.523	24.2	0.27590	62.9	0.02396	5.0	0.079	152.6	7.5	247.4	138.3	1282.5	1252.3	152.6	7.5	11.9
21AR228_208	610.0	2225.757	4.666	13.888	1.6	1.10055	4.2	0.12184	2.9	0.697	741.1	20.4	753.6	22.2	791.8	62.8	741.1	20.4	93.6
21AR228_209	129.6	3264.848	3.045	16.758	5.2	0.57466	12.7	0.07546	3.1	0.246	469.0	14.1	461.0	46.9	422.5	274.1	469.0	14.1	111.0
21AR228_210	391.2	12834.830	1.636	12.528	1.5	1.94896	3.0	0.17868	1.8	0.591	1059.8	17.2	1098.1	20.0	1175.7	47.5	1175.7	47.5	90.1
21AR228_211	30.7	530.270	4.307	11.483	18.9	0.44232	49.3	0.05344	3.5	0.071	335.6	11.5	371.9	153.8	605.6	1078.9	335.6	11.5	55.4
21AR228_212	18.8	554.247	0.734	10.563	18.9	0.91723	76.9	0.09618	3.6	0.047	592.0	20.4	660.9	377.7	904.6	1642.1	592.0	20.4	65.4
21AR228_213	77.4	2270.689	1.942	16.091	6.8	0.62031	15.7	0.08033	1.7	0.106	498.1	8.0	490.0	61.2	453.6	347.9	498.1	8.0	109.8
21AR228_214	94.6	1458.213	4.997	14.303	9.3	0.32332	17.3	0.03878	2.3	0.133	245.3	5.6	284.5	43.0	621.3	371.1	245.3	5.6	39.5
21AR228_215	159.2	2975.251	21.914	16.991	5.4	0.52831	16.3	0.07067	2.1	0.131	440.2	9.1	430.7	57.3	381.0	364.4	440.2	9.1	115.6
21AR228_216	79.8	1490.561	3.204	15.000	10.3	0.33190	24.7	0.04210	2.1	0.086	265.8	5.5	291.0	62.5	499.3	543.3	265.8	5.5	53.2
21AR228_217	172.4	1107.162	4.315	16.348	3.9	0.50938	9.2	0.07711	1.7	0.185	478.8	7.8	418.0	31.5	95.9		478.8	7.8	499.4
21AR228_218	120.0	6159.411	5.487	13.458	2.1	1.59049	5.0	0.16033	1.6	0.311	958.6	13.9	966.5	31.3	985.4	97.0	985.4	97.0	97.3
21AR228_219	353.8	3931.334	5.752	16.820	2.5	0.64735	13.1	0.08426	1.6	0.121	521.5	8.0	506.8	52.5	442.2	290.6	521.5	8.0	117.9
21AR228_220	329.8	6680.292	3.097	17.957	3.2	0.43342	5.2	0.05879	1.5	0.286	368.2	5.3	365.6	15.9	349.9	112.0	368.2	5.3	105.2
21AR228_221	504.3	892.317	2.419	17.699	3.2	0.21672	24.4	0.03948	2.8	0.113	249.6	6.7	199.2	44.1	NA		249.6	6.7	
21AR228_222	340.1	1005.175	2.916	16.300	2.7	0.46429	13.6	0.07208	2.4	0.178	448.7	10.5	387.2	43.9	35.8		448.7	10.5	1253.0
21AR228_223	170.7	4657.077	2.257	17.461	4.0	0.53790	6.5	0.07178	1.7	0.259	446.9	7.3	437.0	23.1	386.5	141.4	446.9	7.3	115.6
21AR228_224	452.9	968.883	1.172	13.853	15.1	0.06421	20.5	0.00812	3.2	0.157	52.1	1.7	63.2	12.6	506.3	446.4	52.1	1.7	10.3
21AR228_225	790.6	18740.982	11.474	16.868	1.6	0.71567	2.6	0.08836	1.8	0.695	545.8	9.5	548.1	11.1	558.5	41.0	545.8	9.5	97.7
21AR228_226	119.6	2703.044	26.931	16.085	6.1	0.54602	11.8	0.06946	1.6	0.133	432.9	6.6	442.4	42.3	493.0	258.2	432.9	6.6	87.8
21AR228_227	191.6	3476.031	1.060	17.644	4.8	0.41705	7.2	0.05698	1.5	0.211	357.3	5.3	353.9	21.6	333.2	160.4	357.3	5.3	107.2
21AR228_228	260.3	6273.189	1.478	17.767	3.1	0.46373	5.3	0.06219	2.0	0.375	388.9	7.5	386.8	17.1	375.3	111.1	388.9	7.5	103.7
21AR228_229	165.2	13049.251	3.713	9.910	1.5	3.64670	2.6	0.26429	1.8	0.694	1511.8	24.3	1559.8	20.7	1626.2	34.8	1626.2	34.8	93.0
21AR228_230	99.9	1370.712	2.988	16.291	7.4	0.39019	14.2	0.05571	1.8	0.129	349.5	6.2	334.5	40.5	232.7	326.0	349.5	6.2	150.2
21AR228_231	87.4	1307.500	2.335	15.148	8.5	0.36044	24.3	0.04755	2.1	0.088	299.5	6.3	312.5	65.4	412.2	542.6	299.5	6.3	72.7
21AR228_232	381.3	5857.425	1.567	18.364	3.1	0.33597	4.7	0.04680	1.5	0.318	294.8	4.3	294.1	12.1	289.4	102.6	294.8	4.3	101.9
21AR228_233	132.9	6685.945	1.713	13.139	2.6	1.68645	6.1	0.16518	1.6	0.263	985.5	14.8	1003.4	39.2	1043.6	119.6	1043.6	119.6	94.4
21AR228_234	309.5	5466.399	92.381	17.491	3.4	0.51082	8.4	0.06814	3.8	0.454	424.9	15.7	419.0	28.8	387.4	168.1	424.9	15.7	109.7
21AR228_235	428.4	5209.170	2.685	18.271	3.7	0.29978	5.9	0.04198	2.0	0.329	265.1	5.1	266.2	13.9	277.2	128.4	265.1	5.1	95.6
21AR228_236	121.4	6060.452	1.993	13.342	2.4	1.63315	4.0	0.16355	1.5	0.384	976.5	13.8	983.1	24.9	998.7	74.1	998.7	74.1	97.8
21AR228_237	16.0	154.926	0.790	7.324	35.9	0.13204	309.0	0.02342	15.9	0.051	149.3	23.4	125.9	370.0	NA		149.3	23.4	
21AR228_238	517.5	4378.621	2.727	18.499	3.6	0.15491	9.8	0.02221	1.5	0.157	141.6	2.2	146.2	13.3	222.6	223.5	141.6	2.2	63.6
21AR228_239	21.1	199.706	0.611	5.677	38.8	0.36503	56.7	0.02460	9.5	0.167	156.6	14.7	316.0	154.1	1760.4	1042.7	156.6	14.7	8.9
21AR228_240	166.8	9879.065	4.048	13.192	2.1	1.71887	3.3	0.16810	2.0	0.604	1001.6	18.3	1015.6	21.0	1046.7	52.6	1046.7	52.6	95.7
21AR228_241	142.5	1519.422	1.488	16.476	7.1	0.26940	16.2	0.03821	2.1	0.128	241.7	4.9	242.2	35.0	247.7	371.1	241.7	4.9	97.6
21AR228_242	40.5	547.840	1.081	9.626	15.8	0.50572	42.1	0.04693	3.2	0.075	295.6	9.1	415.6	143.8	1151.7	842.4	295.6	9.1	25.7
21AR228_243	67.9	901.342	1.941	11.996	13.2	0.36708	38.5	0.03942	2.8	0.074	249.2	7.0	317.5	105.0	855.5	804.1	249.2	7.0	29.1
21AR228_244	173.0	2742.019	4.204	17.107	5.3	0.43809	16.9	0.05970	2.1	0.122	373.8	7.4	368.9	52.1	339.3	379.4	373.8	7.4	110.2
21AR228_245	68.7	843.688	2.458	14.267	10.3	0.28346	48.6	0.03883	4.8	0.098	245.5	11.5	253.4	109.2	327.7		245.5	11.5	74.9
21AR228_246	246.0	7645.239	3.553	15.702	1.9	0.91040	4.8	0.10658	1.7	0.363	652.8	10.8	657.3	23.2	673.4	95.4	652.8	10.8	96.9
21AR228_247	148.7	7726.144	12.677	13.410	1.7	1.62397	3.8	0.16154	1.2	0.307	965.3	10.5	979.5	23.9	1012.3	73.5	1012.3	73.5	95.4
21AR228_248	176.7	10886.603	1.272	12.398	1.6	2.17897	2.6	0.19781	1.4	0.530	1163.6	14.5	1174.3	17.9	1195.1	43.0	1195.1	43.0	97.4
21AR228_249	50.4	658.649	1.251	12.956	18.8	0.30708	44.9	0.04054	2.8	0.062	256.2	6.9	271.9	107.1	410.4		256.2	6.9	62.4
21AR228_250	124.4	1468.501	2.968	15.770	10.9	0.28940	24.4	0.03933	2.1	0.087	248.7	5.2	258.1	55.7	345.2	552.9	248.7	5.2	72.0
21AR228_251	173.4	7036.283	3.510	13.171	1.6	1.80554	4.4	0.17749	1.4	0.316	1053.3	13.5	1047.5	28.8	1036.3	84.4	1036.3	84.4	101.6
21AR228_252	122.4	496.816	1.916	9.601	27.3	0.12059	73.1	0.01160	4.1	0.057	74.4	3.1	115.6	79.9	1079.1	1515.7	74.4	3.1	6.9
21AR228_253	394.3	6094.352	5.524	9.346	1.2	3.63153	2.0	0.25169	1.5	0.770	1447.2	19.8	1556.5	15.8	1708.9	23.3	1708.9	23.3	84.7
21AR228_254	90.0	1152.668	2.124	13.829	12.2	0.34362	22.8	0.04182	2.4	0.106	264.1	6.2	299.9	59.2	589.8	493.0	264.1	6.2	44.8
21AR228_255	222.6	32870.572	4.530	6.359	1.5	9.31335	2.7	0.43155	2.2	0.822	2312.7	43.6	2369.3	25.0	2419.1	26.3	2419.1	26.3	95.6
21AR228_256	112.9	5467.796	2.060	14.000	3.9	1.37718	7.6	0.14547	6.0	0.790	875.5	49.2	879.2	44.8	889.5	96.3	875.5	49.2	98.4
21AR228_257	74.7	644.273	1.078	13.681	13.7	0.19562	71.6	0.02803	2.8	0.039	178.2	4.9	181.4	119.0	224.5		178.2	4.9	79.4
21AR228_258	414.9	9673.095	3.335	16.724	1.8	0.69995	3.6	0.08690	1.7	0.464	537.2	8.6	538.8	15.0	546.3	69.6	537.2	8.6	98.3
21AR228_259	257.8	7752.742	30.387	14.417	2.1	1.08685	4.8	0.11652	3.7	0.769	710.5	24.8	747.0	25.3	858.8	63.5	710.5	24.8	82.7
21AR228_260	418.0	9124.232	101.328	17.406	2.4	0.54976	4.8	0.07124	1.5	0.303	443.6	6.3	444.8	17.4	452.1	102.1	443.6	6.3	98.1
21AR228_261	87.0	3354.326	1.870	12.705	3.3	1.83479	9.0	0.17832	1.5	0.163	1057.8	14.3	1058.0	58.9	1059.3	178.1	1059.3	178.1	99.9
21AR228_262	116.1	922.704	0.924	15.079	9.1	0.23281	46.2	0.03332	2.0	0.044	211.3	4.3	212.5	88.6	226.8		211.3	4.3	93.2
21AR228_263	130.9	1026.720	1.568	17.071	5.6	0.24661	27.3	0.04041	1.7	0.064	255.4	4.3	223.8	54.9	NA		255.4	4.3	
21AR228_264	92.3	5068.863	3.643	13.607	3.4	1.43872	7.5	0.14734	2.5	0.338	886.0	20.9	905.2	44.7	953.2	143.8	953.2	143.8	93.0
21AR228_265	329.7	5692.557	119.167	17.449	2.7	0.51955	5.1	0.06868	2.5	0.489	428.2	10.2	424.8	17.6	407.6	98.7	428.2	10.2	105.1
21AR228_266	162.8	10587.083	2.631	13.742	2.0	1.60318	3.2	0.16172	1.9	0.604	966.3	17.2	971.4	19.8	984.0	51.5	984.0	51.5	98.2
21AR228_267	311.9	1179.214	3.238	13.500	1.5	1.02685	4.0	0.12010	1.4	0.364	731.1	10.0	717.4	20.4	675.5	78.8	731.1	10.0	108.2
21AR228_268	335.6	5601.446	2.115	15.064	9.7	0.13144	11.6	0.01497	3.3	0.286	95.8	3.1	125.4	13.6	731.8	234.8	95.8	3.1	13.1
21AR228_269	88.8	9092.590	1.709	15.088	7.3	0.65212	8.1	0.07325	2.0	0.240	455.7	8.6	509.8	32.6	761.1	166.7	455.7	8.6	59.9
21AR228_270	351.8	78692.561	3.771	13.290	1.6	1.82761	2.4	0.17679	1.8	0.745	1049.4	17.5	1055.4	15.9	1068.8	32.6	1068.8	32.6	98.2
21AR228_271	209.8	11726.331	1.082	17.302	7.2	0.30395	8.4	0.03906	2.1	0.245	247.0	5.0	269.5	19.8	470.8	179.5	247.0	5.0	52.5
21AR228_272	538.6	34242.061	4.698	16.314	3.8	0.65324	5.6	0.07798	4.0	0.709	484.1	18.5	510.5	22.4	631.5	84.9	484.1	18.5	76.7
21AR228_273	475.1	7270.851	18.272	16.867	2.6	0.56490	4.1	0.07166	2.7	0.646	446.1	11.5	454.7	15.2	499.3	69.5	446.1	11.5	89.4
21AR228_274	49.4	3259.527	8.673	12.359	17.0	0.62698	21.2	0.05951	7.9	0.374	372.7	28.7	494.2	82.8	1106.7	392.9	372.7	28.7	33.7
21AR228_275	61.2	601.340	1.285	5.380	60.2	0.18417	62.9	0.00812	15.9	0.253	52.1	8.3	171.6	99.4	2503.0	1050.9	52.1	8.3	2.1
21AR228_276	122.2	12233.606	2.846	16.259	5.3	0.73536	6.6	0.08859	3.6	0.549	547.2	19.0	559.7	28.3	611.8	119.1	547.2	19.0	89.4
21AR228_277	198.0	32038.309	2.953	13.561	2.0	1.72763	2.9	0.17129	1.7	0.610	1019.2	16.5	1018.9	18.4	1019.0	45.9	1019.0	45.9	100.0
21AR228_278	390.6	6959.217	1.894	17.849	4.3	0.28516	6.5	0.03840	2.1	0.323	242.9	5.0	254.7	14.5	365.9	137.6	242.9	5.0	66.4
21AR228_279	256.1	2506.141	1.342	10.971	18.3	0.11634	21.2	0.00987	5.1	0.239	63.3	3.2	111.7	22.5	1326.4	400.1	63.3	3.2	4.8
21AR228_280	179.5	10033.043	0.865	17.909	7.7	0.38085	9.1	0.05093	2.2	0.237	320.2	6.7	327.7	25.5	381.8	199.0	320.2	6.7	83.9
21AR228_281	66.0	5574.943	1.149	13.563	14.9	0.56986	16.6	0.05823	2.6	0.159	364.9	9.4	457.9	61.3	957.6	336.2	364.9	9.4	38.1
21AR228_282	69.7	2479.905	0.500	10.428	19.8	0.35012	22.5	0.02821	7.8	0.345	179.3	13.8	304.8	59.4	1426.9	404.9	179.3	13.8	12.6
21AR228_283	197.0	14574.293	3.310	16.781	5.1	0.51505	5.6	0.06392	2.2	0.388	399.4	8.5	421.8	19.5	547.1	113.6	399.4	8.5	73.0
21AR228_284	467.7	37364.731	3.080	17.469	2.6	0.58141	3.3	0.07446	1.9	0.588	463.0	8.6	465.4	12.2	478.1	58.6	463.0	8.6	96.8
21AR228_285	133.5	1440.377	1.240	8.231	28.6	0.15511	31.1	0.01008	7.6	0.243	64.6	4.9	146.4	42.4	1827.1	550.1	64.6	4.9	3.5
21AR228_286	112.2	27568.803	1.220	9.187	1.6	4.15755	2.5	0.27934	1.8	0.740	1588.1	26.0	1665.7	20.4	1765.8	30.6	1765.8	30.6	89.9
21AR228_287	675.2	98428.227	6.317	13.872	1.3	1.36395	2.3	0.13803	1.9	0.822	833.5	14.8	873.6	13.4	977.4	26.6	977.4	26.6	85.3
21AR228_288	177.0	12182.599	0.834	17.183	6.0	0.49383	7.3	0.06307	2.8	0.383	394.3	10.6	407.5	24.4	484.1	148.2	394.3	10.6	81.4
21AR228_289	297.6	28016.576	6.308	17.174	3.8	0.64551	4.3	0.08119	1.9	0.453	503.2	9.4	505.7	17.0	518.0	83.7	503.2	9.4	97.2
21AR228_290	296.1	25222.959	2.207	17.550	3.8	0.57137	4.5	0.07382	2.0	0.439	459.1	8.7	458.9	16.6	458.8	89.5	459.1	8.7	100.1
21AR228_291	914.2	25044.703	3.246	19.649	2.9	0.17638	3.4	0.02549	1.8	0.514	162.3	2.8	164.9	5.2	204.4	68.2	162.3	2.8	79.4
21AR228_292	323.0	29735.203	16.217	16.958	3.2	0.60138	4.1	0.07477	2.0	0.489	464.8	8.9	478.1	15.5	543.4	77.7	464.8	8.9	85.5
21AR228_293	485.6	43593.319	29.860	14.694	1.9	1.06243	3.3	0.11406	2.6	0.781	696.3	17.3	735.0	17.5	855.8	43.4	696.3	17.3	81.4
21AR228_294	33.9	2529.934	1.045	10.817	16.8	0.58243	20.3	0.04873	4.9	0.240	306.7	14.6	466.0	75.8	1354.4	380.6	306.7	14.6	22.6
21AR228_295	309.0	29342.015	1.264	17.940	4.1	0.44195	5.0	0.05817	2.3	0.467	364.5	8.2	371.6	15.4	417.5	97.8	364.5	8.2	87.3
21AR228_296	119.6	12935.717	0.886	17.036	5.3	0.68090	6.2	0.08591	2.2	0.362	531.3	11.4	527.3	25.4	511.1	126.5	531.3	11.4	104.0
21AR228_297	130.0	3973.152	3.230	13.061	3.3	1.70481	10.2	0.16946	1.9	0.187	1009.1	17.8	1010.3	65.4	1013.9	203.5	1013.9	203.5	99.5
21AR228_298	71.6	2276.282	1.004	8.868	25.8	0.40791	26.4	0.02771	4.4	0.168	176.2	7.7	347.4	77.7	1745.4	478.9	176.2	7.7	10.1
21AR228_299	266.2	17468.673	0.579	17.725	4.4	0.46174	5.1	0.06026	1.9	0.374	377.2	7.0	385.5	16.4	436.2	105.5	377.2	7.0	86.5
21AR228_300	38.2	4703.629	2.619	11.897	14.2	0.89219	15.0	0.07993	3.7	0.247	495.7	17.7	647.5	71.8	1221.2	285.8	495.7	17.7	40.6
21AR228_301	87.2	6133.185	10.079	14.818	10.2	0.57568	11.6	0.06411	2.4	0.206	400.6	9.3	461.7	43.1	779.2	239.3	400.6	9.3	51.4
21AR228_302	121.7	33222.845	2.185	12.631	2.5	2.05068	3.2	0.18892	1.9	0.601	1115.5	19.6	1132.5	21.8	1166.1	50.5	1166.1	50.5	95.7
21AR228_303	484.7	26180.002	2.726	19.014	3.2	0.30640	4.0	0.04276	2.1	0.539	269.9	5.7	271.4	9.5	284.9	76.8	269.9	5.7	94.8
21AR228_304	189.3	9066.178	1.073	17.028	6.1	0.32604	7.1	0.04132	3.1	0.431	261.0	7.9	286.5	17.8	501.3	141.7	261.0	7.9	52.1
21AR228_305	84.1	20401.312	1.965	13.074	4.3	1.81155	5.0	0.17302	2.4	0.478	1028.7	22.7	1049.6	32.6	1094.3	87.6	1094.3	87.6	94.0
21AR228_306	429.0	23004.998	2.355	16.859	2.5	0.69556	3.2	0.08580	1.8	0.551	530.7	9.1	536.1	13.5	560.4	58.8	530.7	9.1	94.7
21AR228_307	78.4	7820.060	0.861	14.514	10.0	0.78533	11.0	0.08454	2.2	0.201	523.1	11.1	588.5	49.1	850.4	224.0	523.1	11.1	61.5
21AR228_308	207.3	16201.656	2.423	16.794	4.5	0.57229	5.6	0.07054	3.0	0.529	439.4	12.7	459.5	20.8	562.2	104.2	439.4	12.7	78.1
21AR228_309	141.2	33504.473	4.220	14.016	2.6	1.63046	3.3	0.16619	2.0	0.609	991.1	18.4	982.0	20.7	962.8	53.4	962.8	53.4	102.9
21AR228_310	202.8	33507.197	2.627	13.358	2.3	1.49590	3.3	0.14594	2.2	0.679	878.2	18.2	928.7	19.8	1051.7	48.2	1051.7	48.2	83.5
21AR228_311	246.2	23110.615	8.688	16.784	3.9	0.60473	4.3	0.07405	1.4	0.320	460.5	6.2	480.2	16.6	576.5	89.1	460.5	6.2	79.9

21AR228_312	379.1	165383.733	3.688	8.772	1.5	4.61162	5.5	0.29179	5.3	0.963	1650.5	77.7	1751.4	46.2	1874.8	26.9	1874.8	26.9	88.0
21AR228_313	33.8	9893.697	35.180	11.839	7.7	2.50833	8.3	0.21770	2.8	0.331	1269.7	31.8	1274.4	60.6	1283.3	153.5	1283.3	153.5	98.9
21AR228_314	190.6	16142.669	1.467	17.163	3.6	0.58703	4.5	0.07376	2.2	0.492	458.8	9.9	469.0	17.0	520.2	86.6	458.8	9.9	88.2
21AR228_315	189.0	18555.772	1.501	16.759	4.6	0.60344	5.1	0.07386	1.9	0.364	459.4	8.3	479.4	19.6	577.4	103.9	459.4	8.3	79.6
21AR228_316	104.3	3911.797	1.107	12.200	16.0	0.42393	17.0	0.03904	4.7	0.278	246.9	11.4	358.9	51.3	1166.7	323.2	246.9	11.4	21.2
21AR228_317	359.4	17273.586	1.126	17.824	5.2	0.29971	6.0	0.03919	1.6	0.275	247.8	4.0	266.2	13.9	431.8	127.6	247.8	4.0	57.4
21AR228_318	160.1	32635.964	2.455	6.405	1.4	7.70446	2.7	0.35748	2.3	0.861	1970.2	39.6	2197.1	24.4	2416.9	23.4	2416.9	23.4	81.5
21AR228_319	107.7	9199.049	1.570	16.009	7.5	0.51722	8.8	0.06138	2.7	0.310	384.0	10.1	423.3	30.4	644.2	179.7	384.0	10.1	59.6
21AR228_320	156.6	13251.336	1.565	16.369	5.6	0.53208	6.7	0.06440	3.5	0.521	402.3	13.5	433.2	23.5	601.7	123.0	402.3	13.5	66.9
21AR228_321	206.6	20607.965	1.218	17.044	4.4	0.59695	5.0	0.07432	1.9	0.384	462.1	8.6	475.3	19.1	540.3	101.9	462.1	8.6	85.5
21AR228_322	586.5	47095.123	1.814	13.757	1.7	1.41364	2.8	0.14128	2.1	0.743	851.9	16.6	894.7	16.6	1002.9	37.9	1002.9	37.9	84.9
21AR228_323	583.4	18986.802	15.690	15.943	2.2	0.78759	6.2	0.09195	5.6	0.912	567.1	30.6	589.8	27.7	679.2	54.2	567.1	30.6	83.5
21AR228_324	82.1	5524.730	0.831	15.772	6.2	0.47253	7.8	0.05624	2.5	0.317	352.7	8.5	392.9	25.4	638.0	159.0	352.7	8.5	55.3
21AR228_325	375.3	31378.730	3.479	17.483	2.7	0.57161	3.7	0.07289	2.1	0.559	453.5	9.0	459.1	13.6	487.7	67.3	453.5	9.0	93.0
21AR228_326	149.0	6418.520	0.971	15.446	10.7	0.25181	11.4	0.02902	2.5	0.217	184.4	4.5	228.0	23.2	707.1	236.3	184.4	4.5	26.1
21AR228_327	129.5	5401.073	2.021	14.576	8.1	0.37496	9.2	0.04098	2.2	0.241	258.9	5.7	323.3	25.6	818.9	187.6	258.9	5.7	31.6
21AR228_328	253.1	53575.100	2.726	13.212	2.0	1.75585	2.6	0.16775	1.6	0.627	999.7	15.1	1029.3	16.8	1093.7	40.5	1093.7	40.5	91.4
21AR228_329	71.9	6500.091	2.105	13.947	10.1	0.77689	10.6	0.08118	2.6	0.243	503.2	12.5	583.7	47.1	911.8	211.8	503.2	12.5	55.2
21AR228_330	389.3	80984.002	4.084	12.278	1.4	2.12461	3.9	0.18838	3.7	0.931	1112.6	37.3	1156.8	27.1	1241.6	28.1	1241.6	28.1	89.6
21AR228_331	630.9	6078.402	29.094	15.582	2.4	0.67772	7.8	0.07902	7.1	0.911	490.3	33.5	525.4	31.9	681.9	68.7	490.3	33.5	71.9
21AR228_332	208.0	1402.939	2.579	9.561	22.0	0.10059	24.3	0.00763	5.3	0.218	49.0	2.6	97.3	22.5	1540.5	447.0	49.0	2.6	3.2
21AR228_333	147.6	31942.950	1.878	13.471	2.5	1.68248	3.2	0.16458	1.7	0.531	982.2	15.5	1001.9	20.4	1046.3	54.8	1046.3	54.8	93.9
21AR228_334	139.5	17757.983	2.859	16.155	4.2	0.67605	5.3	0.07988	2.2	0.421	495.4	10.7	524.4	21.8	653.5	103.6	495.4	10.7	75.8
21AR228_335	102.9	21313.342	0.979	11.512	2.9	2.31540	3.9	0.19385	2.5	0.645	1142.2	26.2	1217.0	27.5	1353.0	57.1	1353.0	57.1	84.4
21AR228_336	107.0	25733.436	2.069	13.102	2.9	1.78148	3.8	0.17108	2.1	0.558	1018.1	20.0	1038.7	24.7	1083.3	63.2	1083.3	63.2	94.0
21AR228_337	61.1	6295.786	1.495	14.145	11.9	0.62529	12.9	0.06638	4.2	0.323	414.3	16.7	493.2	50.4	879.2	252.6	414.3	16.7	47.1
21AR228_338	70.6	1315.907	1.666	6.759	20.8	0.23869	22.9	0.01257	7.4	0.324	80.6	5.9	217.3	44.7	2198.8	376.7	80.6	5.9	3.7
21AR228_339	248.8	10464.995	0.932	17.462	5.6	0.30724	6.6	0.03994	2.1	0.318	252.5	5.2	272.0	15.8	445.1	139.6	252.5	5.2	56.7
21AR228_340	268.4	73612.875	3.122	13.249	1.5	1.83291	3.0	0.17680	2.6	0.855	1049.5	24.9	1057.3	19.7	1074.5	31.3	1074.5	31.3	97.7
21AR228_341	199.7	43195.044	2.060	13.019	2.0	1.81878	3.0	0.17292	2.2	0.729	1028.2	20.5	1052.2	19.4	1103.4	40.6	1103.4	40.6	93.2
21AR228_342	265.3	34917.836	1.091	15.726	3.1	0.89576	4.1	0.10331	2.7	0.654	633.8	16.2	649.5	19.7	705.3	65.9	633.8	16.2	89.9
21AR228_343	494.9	47557.912	1.322	16.408	2.3	0.79774	3.3	0.09585	2.2	0.686	590.1	12.6	595.6	14.7	617.5	51.4	590.1	12.6	95.6
21AR228_344	370.0	68155.624	3.064	12.662	1.9	1.81531	3.5	0.16792	2.9	0.826	1000.6	26.6	1051.0	22.7	1158.1	38.8	1158.1	38.8	86.4
21AR228_345	119.8	7290.287	0.793	15.593	7.9	0.48070	8.5	0.05627	2.0	0.233	352.9	6.8	398.6	28.0	673.8	176.7	352.9	6.8	52.4
21AR228_346	216.1	27754.750	4.673	16.310	3.2	0.83342	4.0	0.09965	2.0	0.502	612.4	11.7	615.5	18.5	628.0	74.6	612.4	11.7	97.5
21AR228_347	32.8	160.560	1.851	7.058	18.3	0.48623	28.1	0.07020	3.3	0.117	437.3	13.9	402.3	93.5	207.0		437.3	13.9	211.3
21AR228_348	116.5	9828.501	0.294	16.409	6.2	0.51194	6.8	0.06264	1.9	0.281	391.7	7.3	419.8	23.5	578.0	142.8	391.7	7.3	67.8
21AR228_349	208.0	10168.417	1.564	16.933	7.9	0.33163	8.5	0.04187	2.2	0.254	264.4	5.6	290.8	21.6	509.3	181.4	264.4	5.6	51.9
21AR228_350	123.9	5162.601	1.760	13.223	13.6	0.33540	14.8	0.03349	2.7	0.185	212.4	5.7	293.7	37.7	1004.3	295.3	212.4	5.7	21.1
21AR228_351	136.5	20510.349	2.639	13.275	2.6	1.66882	5.4	0.16217	4.7	0.869	968.8	42.5	996.7	34.5	1059.6	54.3	1059.6	54.3	91.4
21AR228_352	112.9	10426.346	1.044	16.289	7.1	0.59549	7.6	0.07226	2.3	0.308	449.7	10.2	474.4	28.9	596.3	157.2	449.7	10.2	75.4
21AR228_353	544.8	16117.973	1.627	18.248	3.5	0.18016	4.5	0.02443	2.1	0.465	155.6	3.2	168.2	6.9	350.6	89.7	155.6	3.2	44.4
21AR228_354	776.4	135981.786	131.610	12.642	1.1	1.93039	1.9	0.17856	1.6	0.821	1059.1	15.6	1091.7	13.0	1158.0	21.9	1158.0	21.9	91.5
21AR228_355	34.5	210.482	10.933	7.370	12.8	0.59464	22.9	0.06438	5.1	0.224	402.2	20.0	473.8	86.6	838.2	465.4	402.2	20.0	48.0
21AR228_356	141.0	2141.096	0.906	11.159	15.8	0.18197	20.3	0.01602	4.5	0.221	102.4	4.6	169.8	31.7	1256.0	387.5	102.4	4.6	8.2
21AR228_357	61.9	1099.498	1.279	5.252	41.0	0.28981	42.8	0.01180	11.2	0.261	75.6	8.4	258.4	97.7	2636.6	694.5	75.6	8.4	2.9
21AR228_358	311.8	43278.635	0.911	15.968	2.7	0.86490	3.5	0.10146	2.2	0.615	623.0	12.9	632.8	16.6	669.0	59.5	623.0	12.9	93.1
21AR228_359	285.6	75591.993	3.770	11.657	1.6	2.49604	2.4	0.21308	1.8	0.746	1245.2	20.4	1270.9	17.5	1315.5	31.1	1315.5	31.1	94.7
21AR228_360	254.5	4595.336	1.245	13.927	11.5	0.13284	11.9	0.01414	2.4	0.202	90.5	2.2	126.6	14.2	874.3	242.2	90.5	2.2	10.3
21AR228_361	116.3	5488.216	0.952	14.661	11.5	0.30560	12.9	0.03366	2.9	0.222	213.4	6.0	270.8	30.8	802.7	264.6	213.4	6.0	26.6
21AR228_362	436.4	19716.580	0.741	18.969	3.4	0.27138	4.7	0.03816	2.1	0.459	241.4	5.1	243.8	10.1	268.0	95.1	241.4	5.1	90.1
21AR228_363	97.5	5154.540	2.685	14.252	10.9	0.40613	11.4	0.04405	2.9	0.250	277.9	7.8	346.1	33.5	834.7	230.8	277.9	7.8	33.3
21AR228_364	173.6	5369.478	1.184	12.156	13.2	0.21347	14.1	0.01965	3.1	0.217	125.4	3.8	196.5	25.1	1168.0	272.2	125.4	3.8	10.7
21AR228_365	169.4	37123.446	1.927	12.802	2.2	1.87633	2.9	0.17693	1.9	0.637	1050.2	18.0	1072.8	19.3	1119.8	44.8	1119.8	44.8	93.8
21AR228_366	52.4	17329.975	2.199	11.853	4.6	2.37399	5.2	0.20824	2.1	0.398	1219.4	23.0	1234.8	37.1	1262.6	93.0	1262.6	93.0	96.6
21AR228_367	77.7	8936.818	0.600	15.092	7.7	0.80875	8.8	0.09169	2.3	0.257	565.5	12.2	601.8	39.9	741.6	179.8	565.5	12.2	76.3
21AR228_368	129.1	22364.306	2.310	13.700	3.2	1.53978	4.1	0.15598	2.2	0.534	934.4	18.9	946.4	25.0	975.4	69.9	975.4	69.9	95.8
21AR228_369	392.2	22512.299	8.166	17.687	3.0	0.46343	4.0	0.06064	1.9	0.477	379.5	7.1	386.6	12.9	430.4	78.8	379.5	7.1	88.2
21AR228_370	111.9	26041.160	4.495	13.303	2.7	1.79256	3.4	0.17538	1.9	0.558	1041.7	18.4	1042.8	22.3	1045.9	57.3	1045.9	57.3	99.6
21AR228_371	67.3	7065.512	2.648	12.958	4.2	1.80571	4.9	0.17541	2.0	0.412	1041.9	19.6	1047.5	32.3	1060.2	90.7	1060.2	90.7	98.3
21AR228_372	438.3	33389.441	12.829	17.819	3.6	0.41928	4.1	0.05438	1.8	0.443	341.4	6.0	355.5	12.3	450.0	81.4	341.4	6.0	75.9
21AR228_373	244.4	503.295	2.111	8.047	24.6	0.08365	41.5	0.00632	6.7	0.160	40.6	2.7	81.6	32.5	1549.9	777.5	40.6	2.7	2.6
21AR228_374	333.9	9879.520	1.296	17.302	8.0	0.20692	8.4	0.02681	1.8	0.217	170.5	3.1	191.0	14.7	452.6	182.5	170.5	3.1	37.7
21AR228_375	283.6	53022.380	2.720	13.533	1.8	1.63699	2.9	0.16223	2.2	0.765	969.2	20.2	984.6	18.5	1019.9	38.2	1019.9	38.2	95.0
21AR228_376	86.4	1189.912	1.681	8.827	19.7	0.16569	23.5	0.01188	9.8	0.416	76.2	7.4	155.7	33.9	1645.5	397.4	76.2	7.4	4.6
21AR228_377	472.9	20282.609	2.497	18.457	3.7	0.31579	4.2	0.04310	1.4	0.338	272.0	3.8	278.7	10.2	335.7	88.9	272.0	3.8	81.0
21AR228_378	158.7	6742.523	1.929	16.513	6.2	0.50935	8.0	0.06348	3.7	0.459	396.8	14.2	418.0	27.6	538.0	156.4	396.8	14.2	73.8
21AR228_379	369.5	30494.086	0.927	17.801	2.9	0.53107	3.7	0.06945	1.9	0.527	432.8	8.1	432.5	12.9	431.9	69.2	432.8	8.1	100.2
21AR228_380	177.3	6812.378	0.932	16.475	7.9	0.31563	8.9	0.03925	2.1	0.235	248.2	5.1	278.5	21.6	542.7	188.6	248.2	5.1	45.7
21AR228_381	110.7	9913.802	2.687	17.237	4.5	0.63593	6.0	0.08191	1.7	0.288	507.5	8.4	499.8	23.7	465.5	127.6	507.5	8.4	109.0
21AR228_382	322.4	55397.483	1.674	13.785	2.4	1.57718	3.3	0.15758	2.2	0.670	943.3	19.4	961.3	20.5	1003.4	49.7	1003.4	49.7	94.0
21AR228_383	72.0	6746.087	1.443	16.475	6.7	0.58410	9.7	0.07266	2.0	0.201	452.2	8.6	467.1	36.5	542.0	208.9	452.2	8.6	83.4
21AR228_384	195.0	9936.619	2.457	16.624	5.1	0.34228	5.6	0.04248	2.0	0.353	268.2	5.2	298.9	14.6	547.3	115.2	268.2	5.2	49.0
21AR228_385	706.0	2325.986	0.607	11.663	20.4	0.03162	22.3	0.00289	4.1	0.185	18.6	0.8	31.6	7.0	1180.7	435.3	18.6	0.8	1.6
21AR228_386	117.8	13005.082	0.879	16.187	5.8	0.61208	6.3	0.07349	1.7	0.262	457.1	7.3	484.9	24.5	619.2	132.2	457.1	7.3	73.8
21AR228_387	452.9	29205.430	1.801	18.190	2.3	0.45860	3.0	0.06132	1.7	0.575	383.6	6.4	383.3	9.5	382.2	54.5	383.6	6.4	100.4
21AR228_388	111.4	4330.978	0.516	14.633	15.0	0.33976	16.7	0.03803	2.8	0.169	240.6	6.7	297.0	43.0	768.3	347.2	240.6	6.7	31.3
21AR228_389	61.2	9940.888	2.196	13.441	6.4	1.57106	6.8	0.15659	1.8	0.269	937.8	15.9	958.8	42.0	1008.3	132.4	1008.3	132.4	93.0
21AR228_390	27.7	1699.724	2.793	10.139	24.9	0.67821	27.0	0.05455	4.5	0.169	342.4	15.2	525.7	110.7	1430.1	509.2	342.4	15.2	23.9
21AR228_391	85.0	12343.864	2.534	13.487	6.8	1.62579	7.6	0.16087	2.0	0.269	961.6	18.2	980.2	47.7	1023.1	147.8	1023.1	147.8	94.0
21AR228_392	185.0	13078.254	107.205	16.586	5.6	0.58820	6.0	0.07228	1.7	0.286	449.9	7.4	469.7	22.5	568.8	124.7	449.9	7.4	79.1
21AR228_393	269.1	18665.269	3.179	17.365	4.0	0.47418	4.9	0.06071	2.2	0.448	379.9	8.1	394.1	16.0	478.9	96.9	379.9	8.1	79.3
21AR228_394	342.4	43370.778	1.858	13.764	1.5	1.54728	3.4	0.15554	3.0	0.888	932.0	26.1	949.4	20.9	991.0	31.5	991.0	31.5	94.0
21AR228_395	160.0	19299.339	2.929	16.578	5.4	0.69033	5.7	0.08434	1.6	0.282	522.0	8.1	533.0	23.8	581.4	119.4	522.0	8.1	89.8
21AR228_396	305.4	14543.807	1.005	17.757	5.4	0.28660	5.9	0.03771	1.8	0.303	238.6	4.2	255.9	13.4	418.1	126.0	238.6	4.2	57.1
21AR228_397	166.4	3808.494	0.894	12.017	19.0	0.16353	19.7	0.01497	3.3	0.169	95.8	3.2	153.8	28.1	1178.5	385.2	95.8	3.2	8.1
21AR228_398	134.6	11872.712	1.718	17.073	5.6	0.49057	6.4	0.06225	2.5	0.380	389.3	9.3	405.3	21.5	498.6	131.2	389.3	9.3	78.1
21AR228_399	103.4	5672.311	0.880	12.127	13.0	0.42954	13.6	0.03914	2.4	0.174	247.5	5.7	362.9	41.4	1188.1	264.0	247.5	5.7	20.8
21AR228_400	496.4	4994.523	24.994	17.117	2.9	0.42028	4.4	0.05517	3.2	0.734	346.2	10.9	356.3	13.2	423.3	66.6	346.2	10.9	81.8
21AR228_401	463.6	26401.236	4.574	16.469	2.4	0.74949	3.7	0.08966	2.2	0.595	553.5	11.7	567.9	16.2	626.9	64.4	553.5	11.7	88.3
21AR228_402	133.5	3485.983	2.067	10.744	16.5	0.24400	19.1	0.01995	4.2	0.218	127.3	5.2	221.7	38.0	1398.5	358.0	127.3	5.2	9.1
21AR228_403	320.7	61339.314	3.142	13.729	1.7	1.56862	2.6	0.15728	2.0	0.750	941.6	17.3	957.9	16.3	996.2	35.4	996.2	35.4	94.5
21AR228_404	109.7	14431.052	1.421	15.537	6.7	0.74207	7.1	0.08526	2.2	0.310	527.5	11.1	563.6	30.6	713.3	142.7	527.5	11.1	73.9
21AR228_405	271.4	27579.955	1.822	17.157	3.4	0.64508	3.9	0.08131	1.8	0.447	503.9	8.5	505.4	15.6	513.2	77.1	503.9	8.5	98.2
21AR228_406	33.1	1800.695	1.050	9.653	21.9	0.51179	24.7	0.03885	7.7	0.310	245.7	18.5	419.6	85.0	1539.6	443.6	245.7	18.5	16.0
21AR228_407	164.5	6060.907	0.710	14.082	10.9	0.31797	12.7	0.03377	2.3	0.179	214.1	4.8	280.3	31.1	878.4	259.0	214.1	4.8	24.4
21AR228_408	501.8	10620.542	13.256	15.962	1.5	0.81184	3.2	0.09668	2.8	0.877	594.9	16.0	603.5	14.6	636.7	33.2	594.9	16.0	93.4
21AR228_409	315.9	76644.100	4.795	12.595	1.2	2.03862	2.1	0.18784	1.6	0.788	1109.7	16.8	1128.5	14.2	1165.7	25.5	1165.7	25.5	95.2
21AR228_410	120.0	9169.153	1.596	15.908	8.0	0.49737	8.4	0.05924	2.2	0.260	371.0	7.9	409.9	28.2	636.4	174.0	371.0	7.9	58.3
21AR228_411	124.7	6412.443	1.515	15.225	7.7	0.43007	8.8	0.04949	2.8	0.315	311.4	8.4	363.2	26.9	710.1	178.0	311.4	8.4	43.8
21AR228_412	55.1	669.642	2.961	6.470	41.5	0.17970	50.6	0.00962	16.3	0.322	61.7	10.0	167.8	78.4	2170.3	848.5	61.7	10.0	2.8
21AR228_413	183.7	38267.118	3.658	13.043	2.2	1.80046	3.1	0.17251	2.1	0.674	1025.9	19.8	1045.6	20.2	1087.9	45.8	1087.9	45.8	94.3
21AR228_414	197.8	11693.197	1.359	16.952	6.8	0.37730	8.0	0.04769	2.2	0.271	300.3	6.4	325.0	22.2	507.2	169.0	300.3	6.4	59.2
21AR228_415	353.3	56880.581	47.772	13.807	2.1	1.47161	5.4	0.14881	5.0	0.917	894.3	41.6	918.8	32.8	978.9	44.1	978.9	44.1	91.4
21AR228_416	152.6	38318.120	2.575	13.626	2.8	1.60838	3.6	0.16077	2.1	0.577	961.1	18.4	973.5	22.3	1002.5	59.0	1002.5	59.0	95.9
21AR228_417	156.7	11560.666	2.193	15.964	7.2	0.40168	7.8	0.04776	2.1	0.272	300.7	6.3	342.9	22.7	640.2	161.8	300.7	6.3	47.0
21AR228_418	92.6	4650.837	2.113	13.071	12.4	0.37640	13.8	0.03728	3.2	0.233	236.0	7.4	324.4	38.2	1020.9	271.1	236.0	7.4	23.1
21AR228_419	90.3	27273.172	0.785	10.170	3.3	3.31638	7.5	0.24630	6.7	0.893	1419.4	85.5	1484.9	58.6	1580.6	63.2	1580.6	63.2	89.8
21AR228_420	207.4	12394.026	1.750	16.047	6.1	0.46544	6.6	0.05530	1.7	0.259	347.0	5.8	388.0	21.2	641.9	136.7	347.0	5.8	54.1
21AR228_421	106.1	8226.250	0.323	15.783	7.3	0.52685	8.2	0.06213	2.1	0.260	388.6	8.0	429.7	28.8	657.7	170.0	388.6	8.0	59.1
21AR228_422	392.6	17337.561	1.863	18.171	4.0	0.29541	4.6	0.03960	2.0	0.436	250.4	4.9	262.8	10.6	376.1	93.2	250.4	4.9	66.6
21AR228_423	208.4	31050.483	4.646	15.600	3.0	1.01242	3.9	0.11558	2.3	0.583	705.1	15.4	710.1	20.1	727.0	67.8	705.1	15.4	97.0
21AR228_424	243.8	41106.521	2.104	14.386	2.0	1.37430	2.6	0.14384	1.6	0.601	866.3	12.9	878.0	15.6	908.4	43.6	866.3	12.9	95.4
21AR228_425	436.4	7669.136	1.163	16.526	7.1	0.11578	7.7	0.01427	2.0	0.256	91.3	1.8	111.2	8.2	562.6	163.1	91.3	1.8	16.2
21AR228_426	199.9	8356.585	0.646	15.999	7.0	0.33541	7.5	0.03987	1.8	0.236	252.0	4.3	293.7	19.0	640.8	155.8	252.0	4.3	39.3
21AR228_427	172.8	7903.005	1.051	13.634	13.0	0.29424	13.6	0.02971	3.0	0.218	188.7	5.5	261.9	31.5	981.9	271.1	188.7	5.5	19.2

21AR228_428	102.4	22895.962	1.771	13.886	3.4	1.60212	4.1	0.16211	1.7	0.410	968.5	15.1	971.0	25.6	977.7	76.2	977.7	76.2	99.1
21AR228_429	89.1	3749.324	1.400	11.819	16.9	0.46098	17.4	0.04104	2.7	0.153	259.3	6.7	384.9	55.7	1233.6	337.6	259.3	6.7	21.0
21AR228_430	187.1	23269.337	4.262	16.566	2.9	0.85060	3.8	0.10247	1.5	0.398	628.9	9.1	625.0	17.8	611.8	75.5	628.9	9.1	102.8
21AR228_431	191.5	6274.946	0.650	16.497	9.5	0.23227	11.5	0.02867	2.1	0.185	182.2	3.8	212.1	22.0	558.8	246.0	182.2	3.8	32.6
21AR228_432	120.2	32372.325	4.796	10.059	2.2	3.19895	7.0	0.23356	6.6	0.943	1353.1	81.0	1456.9	54.5	1612.5	43.8	1612.5	43.8	83.9
21AR228_433	200.2	46255.276	2.243	14.258	2.5	1.41126	4.9	0.14536	4.2	0.852	874.9	34.2	893.7	29.2	941.4	52.7	941.4	52.7	92.9
21AR228_434	211.5	10549.123	0.970	17.091	7.3	0.30691	7.7	0.03864	1.8	0.241	244.4	4.4	271.8	18.3	515.6	163.4	244.4	4.4	47.4
21AR228_435	135.6	1474.987	1.254	9.463	27.4	0.12945	34.7	0.00967	11.8	0.339	62.0	7.3	123.6	40.4	1569.6	616.4	62.0	7.3	4.0
21AR228_436	69.3	6429.698	2.649	15.793	9.5	0.67015	10.3	0.07893	2.2	0.219	489.7	10.6	520.8	41.8	660.4	214.7	489.7	10.6	74.2
21AR228_437	190.8	37130.806	2.631	13.471	1.9	1.67474	2.5	0.16315	1.5	0.580	974.2	13.2	999.0	16.1	1054.6	41.5	1054.6	41.5	92.4
21AR228_438	421.4	51603.642	2.368	13.606	1.5	1.57889	2.4	0.15521	1.8	0.767	930.1	15.8	961.9	14.7	1036.3	30.8	1036.3	30.8	89.8
21AR228_439	268.9	31142.642	2.649	17.174	3.1	0.64770	3.6	0.08071	1.5	0.415	500.4	7.2	507.1	14.3	538.3	71.4	500.4	7.2	93.0
21AR228_440	258.7	19787.636	4.926	16.447	4.4	0.52107	5.2	0.06244	1.9	0.371	390.5	7.3	425.9	18.0	623.2	103.5	390.5	7.3	62.6
21AR228_441	341.0	15182.148	2.005	17.762	5.0	0.29193	5.6	0.03812	2.4	0.426	241.2	5.7	260.1	12.9	434.8	113.4	241.2	5.7	55.5
21AR228_442	377.7	42776.001	13.807	14.135	2.4	1.27949	7.1	0.13233	6.7	0.938	801.1	50.2	836.6	40.5	932.9	50.5	801.1	50.2	85.9
21AR228_443	91.0	9618.924	1.427	15.511	7.3	0.62877	8.1	0.07183	2.4	0.297	447.2	10.3	495.3	31.6	725.3	163.1	447.2	10.3	61.7
21AR228_444	66.6	6519.239	1.647	13.802	10.9	0.60826	12.1	0.06255	2.8	0.229	391.1	10.5	482.5	46.4	944.5	240.8	391.1	10.5	41.4
21AR228_445	319.9	23059.646	2.789	17.577	4.8	0.37288	5.4	0.04789	1.9	0.346	301.5	5.6	321.8	15.0	472.1	113.0	301.5	5.6	63.9
21AR228_446	56.5	4792.031	2.597	13.140	8.9	0.81656	10.3	0.08068	2.6	0.251	500.2	12.4	606.1	47.0	1026.1	201.9	500.2	12.4	48.7
21AR228_447	141.1	6420.674	1.570	15.368	7.6	0.32868	9.0	0.03780	3.2	0.360	239.2	7.6	288.6	22.5	711.1	178.0	239.2	7.6	33.6
21AR228_448	293.5	6550.694	2.191	13.915	2.0	1.18655	4.5	0.12303	3.1	0.701	748.0	22.2	794.4	24.7	927.7	65.7	748.0	22.2	80.6
21AR228_449	188.9	33257.573	2.654	13.433	2.4	1.58390	3.1	0.15500	1.8	0.577	928.9	15.3	963.9	19.1	1045.5	50.7	1045.5	50.7	88.9
21AR228_450	260.3	10120.484	0.837	16.704	5.3	0.30700	6.7	0.03819	1.6	0.242	241.6	3.9	271.9	16.0	542.2	142.5	241.6	3.9	44.6
21AR228_451	117.5	40003.818	1.423	10.996	2.1	2.97461	2.8	0.23864	1.7	0.621	1379.6	21.2	1401.2	20.9	1434.8	41.1	1434.8	41.1	96.2
21AR228_452	282.7	69152.704	2.511	11.202	1.8	2.74281	2.7	0.22381	2.0	0.756	1302.0	24.2	1340.1	20.2	1402.5	34.0	1402.5	34.0	92.8
21AR228_453	773.5	54583.874	2.464	13.432	2.1	1.68208	4.1	0.16461	3.5	0.847	982.3	31.8	1001.8	26.3	1045.4	44.3	1045.4	44.3	94.0
21AR228_454	212.7	16192.454	1.735	16.822	5.1	0.49465	5.9	0.06140	2.6	0.444	384.2	9.8	408.1	19.8	546.7	115.2	384.2	9.8	70.3
21AR228_455	640.2	68928.164	1.585	16.417	1.9	0.81330	2.9	0.09738	2.2	0.746	599.0	12.4	604.3	13.2	625.0	41.7	599.0	12.4	95.8
21AR228_456	80.8	21546.963	1.306	13.564	4.9	1.74653	5.7	0.17373	2.7	0.474	1032.6	25.6	1025.9	36.6	1012.3	101.1	1012.3	101.1	102.0
21AR228_457	110.9	14529.944	2.218	16.455	5.7	0.81557	6.9	0.09916	3.2	0.459	609.5	18.3	605.6	31.3	592.0	132.2	609.5	18.3	103.0
21AR228_458	227.6	11381.192	0.971	17.230	7.0	0.29607	7.9	0.03791	2.9	0.362	239.9	6.7	263.3	18.3	478.7	162.9	239.9	6.7	50.1
21AR228_459	426.7	29884.601	1.921	18.383	3.0	0.43237	3.8	0.05829	2.2	0.562	365.2	7.7	364.9	11.8	363.7	71.7	365.2	7.7	100.4
21AR228_460	579.0	57335.058	1.125	16.681	1.8	0.69746	2.5	0.08491	1.6	0.643	525.3	8.0	537.3	10.2	589.2	40.8	525.3	8.0	89.2
21AR228_461	525.7	13841.915	1.863	13.199	1.1	1.46286	2.8	0.14236	2.6	0.911	858.0	20.8	915.2	17.2	1056.7	23.7	1056.7	23.7	81.2
21AR228_462	723.1	5950.647	18.146	16.598	1.7	0.52554	2.5	0.06617	1.8	0.712	413.0	7.0	428.8	8.6	515.7	38.1	413.0	7.0	80.1
21AR228_463	488.7	8481.853	3.077	15.546	2.6	0.94248	5.2	0.10954	1.7	0.331	670.1	11.0	674.2	25.9	688.9	105.6	670.1	11.0	97.3
21AR228_464	244.7	6721.846	1.484	15.544	7.9	0.25086	8.6	0.02933	2.8	0.320	186.4	5.1	227.3	17.6	676.1	175.1	186.4	5.1	27.6
21AR228_465	285.5	6654.752	1.964	15.727	9.8	0.20401	10.2	0.02418	2.3	0.224	154.0	3.5	188.5	17.5	647.1	213.2	154.0	3.5	23.8
21AR228_466	37.4	1268.950	0.695	6.832	35.1	0.51605	38.4	0.02758	7.6	0.198	175.4	13.2	422.5	132.9	2173.9	662.1	175.4	13.2	8.1
21AR228_467	162.6	7170.969	2.813	16.199	8.7	0.29186	12.7	0.03554	8.0	0.630	225.1	17.7	260.0	29.2	588.7	214.2	225.1	17.7	38.2
21AR228_468	317.4	8041.663	1.322	15.575	7.6	0.14801	9.7	0.01724	2.5	0.254	110.2	2.7	140.2	12.7	683.7	200.9	110.2	2.7	16.1
21AR228_469	251.2	3712.569	1.738	14.088	15.3	0.13001	17.3	0.01409	4.5	0.259	90.2	4.0	124.1	20.2	836.9	349.1	90.2	4.0	10.8
21AR228_470	144.9	5779.110	5.748	16.020	5.8	0.60277	7.4	0.07321	1.6	0.214	455.5	7.0	479.0	28.3	594.1	156.8	455.5	7.0	76.7
21AR228_471	119.0	12726.809	1.412	16.043	8.2	0.64595	9.1	0.07697	2.6	0.280	478.0	11.8	506.0	36.3	635.5	188.3	478.0	11.8	75.2
21AR228_472	143.1	2083.122	0.908	14.985	9.0	0.33569	18.6	0.04096	2.6	0.142	258.8	6.7	293.9	47.5	584.3	400.6	258.8	6.7	44.3
21AR228_473	158.5	16207.161	1.959	16.449	6.0	0.61769	6.4	0.07520	1.7	0.267	467.4	7.7	488.4	24.7	589.2	133.0	467.4	7.7	79.3
21AR228_474	55.6	2776.295	0.712	11.375	18.4	0.45530	19.4	0.03996	4.9	0.252	252.6	12.1	381.0	61.7	1261.6	368.0	252.6	12.1	20.0
21AR228_475	249.1	22691.713	1.618	17.570	3.0	0.56381	3.9	0.07291	1.8	0.468	453.7	8.0	454.0	14.2	456.6	76.2	453.7	8.0	99.4
21AR228_476	70.2	23671.245	2.491	11.131	2.9	3.00731	3.3	0.24515	1.5	0.463	1413.4	19.5	1409.5	25.3	1404.3	56.4	1404.3	56.4	100.6
21AR228_477	271.5	16160.217	4.815	18.174	4.5	0.44577	5.2	0.05993	1.7	0.323	375.2	6.1	374.3	16.3	370.0	110.9	375.2	6.1	101.4
21AR228_478	97.6	10125.913	4.054	14.865	9.3	0.66661	9.6	0.07365	2.0	0.206	458.1	8.8	518.6	39.1	795.9	197.8	458.1	8.8	57.6
21AR228_479	199.4	10289.711	1.167	17.321	9.2	0.29152	10.1	0.03766	2.8	0.275	238.3	6.5	259.8	23.2	458.8	216.4	238.3	6.5	51.9
21AR228_480	231.5	51050.398	4.670	12.718	1.6	2.02600	2.4	0.18887	1.7	0.707	1115.2	17.1	1124.3	16.1	1142.6	33.3	1142.6	33.3	97.6
21AR228_481	301.1	18414.767	2.040	17.358	5.4	0.32908	6.3	0.04231	2.4	0.383	267.1	6.3	288.9	15.8	469.7	128.5	267.1	6.3	56.9
21AR228_482	97.3	4739.158	1.835	13.940	8.1	0.39155	10.9	0.04143	3.6	0.335	261.7	9.4	335.5	31.1	885.9	212.2	261.7	9.4	29.5
21AR228_483	34.6	1638.684	1.914	10.526	22.9	0.56862	27.9	0.04805	4.5	0.161	302.5	13.3	457.1	102.9	1335.3	535.8	302.5	13.3	22.7
21AR228_484	399.1	37012.549	70.034	17.746	3.0	0.57699	3.7	0.07532	2.0	0.537	468.1	9.0	462.5	13.9	435.8	70.1	468.1	9.0	107.4
21AR228_485	110.5	3278.992	0.829	13.406	12.9	0.27851	13.7	0.02900	3.1	0.223	184.3	5.6	249.5	30.4	919.2	275.8	184.3	5.6	20.0
21AR228_486	185.4	14440.738	1.702	17.107	5.3	0.45077	6.4	0.05727	1.7	0.265	359.0	5.9	377.8	20.2	496.0	135.9	359.0	5.9	72.4
21AR228_487	125.9	28840.059	0.752	11.731	2.3	2.52373	3.3	0.21730	2.3	0.674	1267.6	25.9	1278.9	24.3	1298.8	48.0	1298.8	48.0	97.6
21AR228_488	535.4	32843.859	1.391	18.314	3.0	0.43507	3.6	0.05863	1.8	0.514	367.3	6.6	366.8	11.0	364.5	69.1	367.3	6.6	100.8
21AR228_489	189.2	11123.321	0.973	17.384	5.4	0.30252	6.3	0.03927	2.6	0.416	248.3	6.4	268.4	14.9	448.0	128.0	248.3	6.4	55.4
21AR228_490	235.5	11331.398	1.628	16.940	6.4	0.31797	7.1	0.04026	2.3	0.319	254.5	5.7	280.3	17.4	503.2	148.6	254.5	5.7	50.6
21AR228_491	233.5	33067.070	1.401	15.738	4.3	0.78754	4.6	0.09108	1.6	0.348	561.9	8.7	589.8	20.7	699.4	92.6	561.9	8.7	80.3
21AR228_492	199.9	959.958	2.278	13.909	7.4	0.58456	45.3	0.07518	2.9	0.063	467.3	12.9	467.4	170.3	468.8		467.3	12.9	99.7
21AR228_493	194.8	25153.190	1.872	13.775	2.4	1.59490	3.3	0.16098	2.1	0.640	962.2	18.6	968.2	20.3	982.7	50.9	982.7	50.9	97.9
21AR228_494	351.1	30065.514	8.327	18.120	3.5	0.44130	4.1	0.05866	2.0	0.475	367.4	7.0	371.2	12.9	395.4	81.7	367.4	7.0	92.9
21AR228_495	36.4	2883.176	0.521	10.327	15.2	0.81033	16.0	0.06409	4.6	0.290	400.5	18.0	602.6	72.7	1461.9	291.4	1461.9	291.4	27.4
21AR228_496	40.2	3843.646	0.504	11.145	17.7	0.75452	18.9	0.06379	4.2	0.221	398.6	16.1	570.8	82.5	1334.3	357.1	398.6	16.1	29.9
21AR228_497	165.3	34809.852	1.071	14.129	3.5	1.47921	5.7	0.15297	4.5	0.782	917.6	38.1	921.9	34.5	933.1	72.9	933.1	72.9	98.3
21AR228_498	206.8	16529.104	1.522	17.449	4.7	0.44763	5.5	0.05772	2.4	0.436	361.8	8.4	375.6	17.2	462.9	109.1	361.8	8.4	78.1
21AR228_499	174.9	8466.492	1.894	16.040	6.2	0.45299	7.3	0.05439	2.3	0.315	341.4	7.7	379.4	23.1	619.2	149.7	341.4	7.7	55.1
21AR228_500	116.8	11687.806	1.620	16.241	6.6	0.61560	7.5	0.07432	2.4	0.321	462.2	10.7	487.1	29.0	607.1	153.6	462.2	10.7	76.1
21AR228_501	351.3	15956.162	4.371	17.896	4.5	0.35071	5.7	0.04648	2.6	0.465	292.9	7.6	305.3	15.0	401.9	112.7	292.9	7.6	72.9
21AR228_502	208.7	11480.580	1.394	17.011	6.4	0.32713	6.9	0.04143	2.0	0.294	261.7	5.2	287.4	17.3	502.7	145.1	261.7	5.2	52.1
21AR228_503	105.6	22292.272	1.702	13.423	3.1	1.71093	3.6	0.16874	1.7	0.480	1005.2	16.3	1012.6	23.3	1029.7	64.6	1029.7	64.6	97.6
21AR228_504	608.3	2478.464	0.873	16.716	4.5	0.30995	15.9	0.04179	3.4	0.214	263.9	8.8	274.1	38.1	363.5	350.0	263.9	8.8	72.6
21AR228_505	56.2	2718.434	2.663	12.240	14.8	0.42981	18.7	0.04091	2.9	0.155	258.5	7.3	363.0	57.1	1101.1	370.4	258.5	7.3	23.5
21AR228_506	113.5	5389.352	0.712	14.959	9.4	0.34742	11.9	0.03939	2.8	0.235	249.1	6.8	302.8	31.1	741.3	244.7	249.1	6.8	33.6
21AR228_507	209.4	7598.288	0.745	15.630	9.7	0.26497	11.1	0.03106	2.4	0.213	197.2	4.6	238.7	23.7	670.7	232.7	197.2	4.6	29.4
21AR228_508	893.4	54759.387	1.610	18.111	1.9	0.45366	2.8	0.06007	1.8	0.642	376.1	6.6	379.8	8.9	403.8	48.3	376.1	6.6	93.1
21AR228_509	229.4	9361.210	2.193	16.659	5.5	0.32893	5.9	0.04093	1.9	0.319	258.6	4.8	288.7	14.8	541.5	122.2	258.6	4.8	47.8
21AR228_510	27.3	7007.171	2.615	11.793	7.2	2.58831	8.3	0.22748	2.2	0.267	1321.3	26.5	1297.3	60.9	1258.8	156.6	1258.8	156.6	105.0
21AR228_511	276.7	20401.052	4.446	16.834	4.9	0.47937	5.9	0.05939	3.2	0.543	371.9	11.5	397.6	19.3	551.2	107.3	371.9	11.5	67.5
21AR228_512	106.6	5188.828	1.183	13.431	13.5	0.41642	15.3	0.04223	2.7	0.174	266.6	7.0	353.5	45.7	973.1	308.0	266.6	7.0	27.4
21AR228_513	324.9	18367.585	1.103	17.126	5.6	0.35997	6.1	0.04544	2.0	0.330	286.5	5.7	312.2	16.5	509.8	127.1	286.5	5.7	56.2
21AR228_514	110.2	15006.535	1.084	15.561	4.4	1.07100	5.6	0.12286	2.3	0.419	747.0	16.4	739.2	29.2	716.7	107.2	747.0	16.4	104.2
21AR228_515	145.2	1637.093	2.038	10.319	22.9	0.11366	27.6	0.00935	4.7	0.171	60.0	2.8	109.3	28.6	1386.1	524.8	60.0	2.8	4.3
21AR228_516	184.1	15000.401	1.735	17.270	4.7	0.61828	5.2	0.07896	2.1	0.399	489.9	9.8	488.8	20.2	484.4	105.2	489.9	9.8	101.1
21AR228_517	83.2	12768.839	1.784	13.702	4.9	1.57771	5.7	0.15965	1.6	0.285	954.8	14.5	961.5	35.6	977.6	111.8	977.6	111.8	97.7
21AR228_518	169.5	5057.537	1.118	14.863	9.1	0.24945	9.9	0.02819	2.9	0.295	179.2	5.1	226.1	20.0	748.8	199.6	179.2	5.1	23.9
21AR228_519	210.6	14476.788	1.858	17.419	4.9	0.47473	5.7	0.06127	2.0	0.342	383.3	7.3	394.5	18.7	461.0	118.9	383.3	7.3	83.2
21AR228_520	462.5	6558.493	0.873	17.228	4.9	0.17813	6.9	0.02323	2.1	0.297	148.0	3.0	166.5	10.6	437.8	146.9	148.0	3.0	33.8
21AR228_521	225.8	43451.128	2.557	13.085	2.0	1.86819	3.7	0.17871	3.0	0.818	1059.9	29.2	1069.9	24.2	1091.1	42.1	1091.1	42.1	97.1
21AR228_522	216.6	5775.915	1.126	17.095	5.7	0.44103	11.2	0.05732	4.1	0.366	359.3	14.3	371.0	34.8	445.5	231.9	359.3	14.3	80.7
21AR228_523	161.0	8516.896	2.732	16.440	6.5	0.33420	8.8	0.04115	2.0	0.222	259.9	5.0	292.8	22.4	564.6	187.0	259.9	5.0	46.0
21AR228_524	60.7	924.352	2.662	5.390	41.1	0.27186	46.7	0.01150	16.7	0.357	73.7	12.2	244.2	101.3	2572.4	738.2	73.7	12.2	2.9
21AR228_525	278.2	18655.212	1.414	17.597	3.7	0.56912	4.8	0.07366	2.6	0.537	458.2	11.4	457.4	17.7	454.8	89.9	458.2	11.4	100.7
21AR228_526	329.1	12525.809	0.564	16.368	3.4	0.63379	5.9	0.07689	3.6	0.621	477.5	16.8	498.5	23.1	596.6	99.7	477.5	16.8	80.0
21AR228_527	247.2	16796.727	6.632	16.878	5.4	0.38575	6.4	0.04792	3.3	0.518	301.7	9.8	331.3	18.2	545.3	120.3	301.7	9.8	55.3
21AR228_528	456.6	7641.275	2.702	16.666	2.0	0.61925	3.6	0.07750	2.9	0.792	481.2	13.3	489.4	14.0	528.9	48.4	481.2	13.3	91.0
21AR228_529	191.5	7908.422	10.868	16.499	7.9	0.44561	11.7	0.05515	8.3	0.709	346.1	28.0	374.2	36.7	553.3	180.6	346.1	28.0	62.5
21AR228_530	83.4	33358.922	1.082	8.675	1.8	5.32573	2.6	0.33723	1.7	0.676	1873.3	28.2	1873.0	21.9	1873.4	34.1	1873.4	34.1	100.0
21AR228_531	138.7	26830.912	4.037	13.285	2.3	1.86850	2.9	0.18184	1.7	0.595	1077.0	17.0	1070.0	19.1	1056.6	46.8	1056.6	46.8	101.9
21AR228_532	174.5	4495.833	3.526	15.612	8.4	0.37274	10.5	0.04463	1.9	0.183	281.4	5.3	321.7	28.8	625.2	221.8	281.4	5.3	45.0
21AR228_533	27.6	1871.397	6.336	9.589	18.7	0.88805	21.1	0.06666	6.5	0.309	416.0	26.3	645.3	101.0	1560.7	377.8	1560.7	377.8	26.7
21AR228_534	70.4	2526.161	1.088	11.849	16.2	0.44520	18.2	0.04110	3.4	0.185	259.6	8.6	373.9	56.9	1162.0	355.2	259.6	8.6	22.3
21AR228_535	61.2	8393.752	1.284	10.137	2.8	3.39089	3.5	0.25424	1.9	0.541	1460.3	24.6	1502.3	27.3	1562.8	55.0	1562.8	55.0	93.4
21AR228_536	449.1	10995.257	1.146	18.273	3.9	0.29198	4.7	0.03977	2.3	0.482	251.4	5.6	260.1	10.8	340.2	93.2	251.4	5.6	73.9
21AR228_537	109.7	10845.181	1.393	16.330	6.9	0.62129	8.2	0.07543	3.9	0.479	468.8	17.7	490.7	31.9	595.1	155.9	468.8	17.7	78.8
21AR228_538	146.2	36871.184	4.997	13.106	3.1	1.72081	4.3	0.16473	2.9	0.680	983.0	26.5	1016.3	27.4	1089.8	62.7	1089.8	62.7	90.2
21AR228_539	113.4	25764.386	4.582	12.547	3.3	2.09253	3.7	0.19212	1.6	0.430	1132.8	16.7	1146.4	25.7	1172.9	66.9	1172.9	66.9	96.6
21AR228_540	71.3	850.964	1.820	5.665	24.8	0.21896	27.7	0.00984	11.5	0.414	63.1	7.2	201.0	50.6	2470.6	428.1	63.1	7.2	2.6
21AR228_541	225.6	58572.453	2.393	13.115	1.8	1.86642	2.4	0.17882	1.7	0.683	1060.5	16.3	1069.3	16.2	1088.0	35.8	1088.0	35.8	97.5

21AR229

21AR229_01	215.3	42018.791	5.338	13.687	1.2	1.64529	2.1	0.16476	1.6	0.767	983.2	14.4	987.7	13.0	998.8	26.8	998.8	26.8	98.4
21AR229_02	101.9	6196.294	1.150	17.144	4.5	0.48546	8.4	0.06316	2.4	0.284	394.8	9.2	401.8	28.0	443.3	179.8	394.8	9.2	89.1
21AR229_03	97.1	1176.878	1.024	11.584	18.5	0.12779	22.2	0.01254	3.4	0.152	80.4	2.7	122.1	25.6	1039.3	445.0	80.4	2.7	7.7
21AR229_04	526.3	4733.795	4.679	16.012	1.6	0.79103	2.9	0.09709	1.7	0.578	597.3	9.5	591.8	12.9	571.4	50.9	597.3	9.5	104.5
21AR229_05	629.1	27687.788	15.448	17.922	1.5	0.49692	2.8	0.06554	1.9	0.657	409.2	7.4	409.6	9.6	412.8	47.9	409.2	7.4	99.1
21AR229_06	131.2	6266.119	1.855	17.303	3.9	0.47171	5.7	0.06199	1.5	0.265	387.7	5.7	392.4	18.6	420.8	123.0	387.7	5.7	92.1
21AR229_07	220.4	14922.667	3.681	16.238	1.9	0.85925	3.2	0.10333	1.8	0.581	633.9	11.2	629.7	14.9	615.6	55.9	633.9	11.2	103.0
21AR229_08	167.0	6243.475	1.495	17.627	2.8	0.55356	5.0	0.07420	2.5	0.495	461.4	11.1	447.3	18.2	376.4	98.3	461.4	11.1	122.6
21AR229_09	121.6	4948.845	0.518	17.877	6.1	0.29582	7.6	0.04069	2.5	0.327	257.1	6.3	263.1	17.6	318.0	163.4	257.1	6.3	80.9
21AR229_10	183.3	9074.626	6.249	18.385	2.7	0.43862	5.0	0.06044	2.3	0.466	378.3	8.6	369.3	15.5	314.1	101.0	378.3	8.6	120.4
21AR229_11	45.4	3007.064	1.809	15.058	7.5	0.62833	10.1	0.07436	1.9	0.186	462.4	8.4	495.1	39.5	650.1	212.5	462.4	8.4	71.1
21AR229_12	209.1	23997.432	2.989	14.622	2.2	1.13719	8.6	0.12227	8.2	0.957	743.6	57.6	771.2	46.3	852.7	51.9	743.6	57.6	87.2
21AR229_13	63.7	36104.140	2.671	5.600	1.3	12.27331	2.6	0.50199	2.2	0.853	2622.3	48.1	2625.5	24.6	2628.7	22.7	2628.7	22.7	99.8
21AR229_14	96.9	3640.459	1.121	16.401	6.9	0.32436	15.7	0.04150	2.0	0.128	262.1	5.2	285.2	39.0	480.3	344.0	262.1	5.2	54.6
21AR229_15	195.7	7708.582	2.256	18.208	5.2	0.34883	7.2	0.04795	4.1	0.572	302.0	12.1	303.8	18.8	319.4	133.6	302.0	12.1	94.5
21AR229_16	489.6	17231.754	1.647	19.345	2.9	0.21063	3.8	0.03018	1.6	0.424	191.6	3.1	194.1	6.7	224.8	79.8	191.6	3.1	85.3
21AR229_17	393.7	30455.990	22.810	16.966	1.6	0.72414	2.7	0.09027	1.7	0.634	557.1	9.1	553.1	11.4	537.5	45.3	557.1	9.1	103.7
21AR229_18	187.1	791.421	1.605	15.576	7.5	0.12448	33.2	0.01993	2.2	0.065	127.2	2.7	119.1	37.3	NA		127.2	2.7	
21AR229_19	319.9	19290.633	3.403	17.674	1.6	0.57957	3.0	0.07567	2.3	0.772	470.2	10.5	464.2	11.1	435.5	42.3	470.2	10.5	108.0
21AR229_20	29.7	1287.388	1.833	11.367	14.2	0.44627	22.2	0.04227	3.1	0.142	266.9	8.2	374.7	69.4	1110.9	439.2	266.9	8.2	24.0
21AR229_21	186.2	8338.154	1.709	18.459	3.8	0.32311	7.1	0.04481	1.6	0.226	282.6	4.4	284.3	17.5	299.1	157.0	282.6	4.4	94.5
21AR229_22	66.2	2204.564	1.756	15.543	7.0	0.46029	11.1	0.05813	1.9	0.168	364.2	6.6	384.5	35.5	509.1	240.9	364.2	6.6	71.5
21AR229_23	56.6	14947.509	1.630	11.366	2.1	2.84057	2.9	0.23789	1.8	0.623	1375.7	22.3	1366.3	21.7	1352.4	43.5	1352.4	43.5	101.7
21AR229_24	130.7	5077.070	1.501	18.310	6.2	0.28815	11.9	0.04061	2.3	0.190	256.6	5.7	257.1	27.1	262.6	269.0	256.6	5.7	97.7
21AR229_25	231.9	46184.283	3.811	12.266	1.4	2.25813	2.8	0.20269	2.4	0.855	1189.8	25.9	1199.3	19.7	1217.4	28.6	1217.4	28.6	97.7
21AR229_26	298.4	19793.656	1.372	18.234	2.0	0.45986	3.1	0.06196	1.9	0.615	387.6	7.1	384.2	9.8	364.7	54.3	387.6	7.1	106.3
21AR229_27	368.8	40438.287	2.095	15.619	1.5	1.01624	2.5	0.11637	1.8	0.716	709.6	12.2	712.0	13.0	720.5	37.6	709.6	12.2	98.5
21AR229_28	41.1	385.660	1.344	6.792	38.7	0.14759	56.9	0.00960	7.5	0.132	61.6	4.6	139.8	74.4	1824.0	1045.5	61.6	4.6	3.4
21AR229_29	168.4	1811.604	3.192	12.834	2.2	1.57503	19.8	0.16396	1.5	0.077	978.7	13.9	960.4	123.4	919.6	407.8	919.6	407.8	106.4
21AR229_30	116.0	8404.792	1.067	16.830	3.9	0.59686	4.9	0.07543	1.6	0.333	468.8	7.4	475.2	18.6	507.5	101.8	468.8	7.4	92.4
21AR229_31	480.1	8171.380	2.154	18.982	3.9	0.11623	5.1	0.01665	1.9	0.362	106.5	2.0	111.7	5.4	224.6	111.0	106.5	2.0	47.4
21AR229_32	181.9	6856.873	1.163	18.213	3.9	0.29334	4.7	0.04044	2.1	0.450	255.6	5.3	261.2	10.8	312.9	95.6	255.6	5.3	81.7
21AR229_33	259.0	19919.753	5.918	17.050	1.8	0.69118	3.3	0.08699	2.1	0.621	537.7	10.7	533.5	13.8	516.5	57.4	537.7	10.7	104.1
21AR229_34	164.8	16238.395	23.326	15.989	2.3	0.75568	3.4	0.08936	2.2	0.660	551.7	11.9	571.5	14.9	651.9	54.9	551.7	11.9	84.6
21AR229_35	37.1	2303.954	1.249	14.295	9.7	0.65463	17.5	0.07495	2.0	0.117	465.9	9.2	511.3	70.5	720.9	370.3	465.9	9.2	64.6
21AR229_36	437.1	40222.058	8.118	15.745	1.3	0.97378	2.2	0.11249	1.8	0.798	687.2	11.6	690.4	11.1	701.9	28.5	687.2	11.6	97.9
21AR229_37	105.2	3078.416	1.113	15.857	9.1	0.23573	11.1	0.02948	2.5	0.220	187.3	4.5	214.9	21.5	530.6	237.8	187.3	4.5	35.3
21AR229_38	112.3	3993.063	2.113	17.658	5.0	0.30026	9.7	0.04136	2.2	0.229	261.2	5.7	266.6	22.8	315.0	215.0	261.2	5.7	82.9
21AR229_39	31.1	1427.347	1.767	13.851	11.8	0.46635	18.7	0.05481	2.8	0.151	344.0	9.5	388.7	60.4	664.8	396.6	344.0	9.5	51.7
21AR229_40	50.9	8981.369	4.821	12.378	2.9	2.24615	5.0	0.20697	1.5	0.293	1212.6	16.1	1195.6	34.8	1165.7	93.8	1165.7	93.8	104.0
21AR229_41	44.9	498.911	2.345	5.739	49.3	0.19975	58.7	0.00982	11.2	0.190	63.0	7.0	184.9	99.3	2318.5	1011.6	63.0	7.0	2.7
21AR229_42	424.2	6974.423	1.022	19.086	4.3	0.11154	5.7	0.01618	1.9	0.328	103.5	1.9	107.4	5.8	195.6	124.6	103.5	1.9	52.9
21AR229_43	416.0	39364.357	1.121	17.623	1.8	0.59180	2.7	0.07637	2.0	0.746	474.4	9.1	472.0	10.1	461.2	39.5	474.4	9.1	102.9
21AR229_44	146.5	3900.722	2.225	16.087	5.8	0.23231	10.3	0.02900	2.1	0.202	184.3	3.8	212.1	19.7	534.4	220.8	184.3	3.8	34.5
21AR229_45	251.5	20916.577	27.989	18.232	2.8	0.49668	3.6	0.06690	1.8	0.493	417.4	7.3	409.5	12.3	365.6	71.5	417.4	7.3	114.2
21AR229_46	171.1	12396.697	3.701	12.853	1.2	2.00647	3.3	0.19090	2.4	0.714	1126.2	24.6	1117.7	22.6	1102.0	46.6	1102.0	46.6	102.2
21AR229_47	486.1	19373.680	1.833	17.540	1.5	0.54080	2.3	0.07011	1.5	0.637	436.8	6.3	438.9	8.3	451.2	39.8	436.8	6.3	96.8
21AR229_48	169.7	6831.209	2.366	18.093	3.8	0.29035	5.0	0.03986	1.8	0.358	252.0	4.4	258.8	11.4	322.5	105.8	252.0	4.4	78.1
21AR229_49	196.7	7624.114	0.620	17.578	3.9	0.28563	4.6	0.03791	1.9	0.404	239.9	4.4	255.1	10.3	398.6	93.8	239.9	4.4	60.2
21AR229_50	145.9	12518.491	1.856	16.904	2.6	0.67411	3.8	0.08480	2.1	0.549	524.7	10.5	523.2	15.5	517.6	69.6	524.7	10.5	101.4
21AR229_51	205.4	6494.506	1.256	17.740	5.1	0.21909	6.1	0.02954	2.1	0.343	187.6	3.9	201.2	11.1	363.6	129.3	187.6	3.9	51.6
21AR229_52	46.7	1838.691	2.999	16.011	7.8	0.31335	29.4	0.04194	2.6	0.088	264.8	6.7	276.8	71.2	380.0	661.3	264.8	6.7	69.7
21AR229_53	159.8	11436.582	4.160	18.259	3.4	0.44957	5.9	0.06132	2.1	0.359	383.6	7.9	377.0	18.5	337.3	124.5	383.6	7.9	113.8
21AR229_54	671.6	14002.518	70.931	13.545	1.3	1.62469	2.6	0.16234	2.2	0.845	969.8	20.0	979.8	16.5	1003.2	28.4	1003.2	28.4	96.7
21AR229_55	453.6	26540.080	8.933	13.421	1.2	1.67717	3.2	0.16531	2.6	0.814	986.2	23.5	999.9	20.1	1030.9	37.1	1030.9	37.1	95.7
21AR229_56	137.7	10757.890	1.599	16.780	3.2	0.66883	4.4	0.08372	1.8	0.407	518.3	8.9	520.0	17.9	528.4	88.1	518.3	8.9	98.1
21AR229_57	250.1	20285.412	5.183	16.295	1.9	0.86487	3.4	0.10396	1.9	0.551	637.6	11.5	632.8	16.2	616.5	62.0	637.6	11.5	103.4
21AR229_58	145.3	39769.196	2.661	5.419	1.5	10.95885	5.9	0.43368	5.7	0.967	2322.3	111.4	2519.6	55.0	2683.4	24.9	2683.4	24.9	86.5
21AR229_59	200.7	29462.181	2.020	13.793	1.9	1.49807	4.3	0.15167	3.9	0.899	910.3	33.1	929.6	26.4	976.6	38.6	976.6	38.6	93.2
21AR229_60	152.5	4417.064	0.794	16.159	2.5	0.76253	11.1	0.09486	1.9	0.170	584.2	10.5	575.5	48.8	542.1	239.3	584.2	10.5	107.8
21AR229_61	53.6	2797.485	28.502	15.618	7.5	0.46059	11.1	0.05705	3.6	0.323	357.7	12.5	384.7	35.6	551.4	230.0	357.7	12.5	64.9
21AR229_62	124.1	7901.593	2.694	17.286	3.0	0.54674	7.2	0.07115	1.8	0.249	443.1	7.7	442.9	26.0	442.7	156.0	443.1	7.7	100.1
21AR229_63	218.5	9399.689	13.230	18.777	3.5	0.30450	5.0	0.04285	2.1	0.410	270.5	5.5	269.9	11.9	266.0	105.2	270.5	5.5	101.7
21AR229_64	133.3	23369.681	3.245	12.169	1.4	2.33442	2.4	0.20856	1.6	0.669	1221.2	17.6	1222.8	16.8	1226.6	34.4	1226.6	34.4	99.6
21AR229_65	236.6	19551.249	2.211	17.123	1.8	0.68334	3.1	0.08638	1.9	0.617	534.1	9.7	528.8	12.6	507.0	52.9	534.1	9.7	105.3
21AR229_66	78.4	3273.492	1.479	14.417	13.3	0.26766	16.1	0.03003	2.2	0.137	190.8	4.2	240.8	34.5	763.4	336.4	190.8	4.2	25.0
21AR229_67	36.6	2446.686	1.446	12.382	12.3	0.60381	13.9	0.05873	2.7	0.196	367.9	9.7	479.6	53.1	1057.6	274.3	367.9	9.7	34.8
21AR229_68	286.6	12769.438	2.572	18.881	2.9	0.31649	4.0	0.04453	2.1	0.518	280.9	5.7	279.2	9.7	266.3	78.0	280.9	5.7	105.5
21AR229_69	116.3	4472.349	1.188	17.764	5.3	0.28772	11.3	0.03956	2.0	0.181	250.1	5.0	256.8	25.7	318.8	253.7	250.1	5.0	78.5
21AR229_70	265.6	27901.080	3.740	16.733	1.9	0.73786	3.2	0.09079	2.1	0.634	560.2	11.0	561.2	14.0	565.9	54.5	560.2	11.0	99.0
21AR229_71	142.9	11039.842	2.114	17.038	3.2	0.70214	4.5	0.08911	1.4	0.307	550.3	7.3	540.1	19.0	498.1	95.0	550.3	7.3	110.5
21AR229_72	333.0	27834.973	27.630	17.756	1.8	0.52109	3.5	0.06781	2.4	0.691	423.0	10.0	425.9	12.3	442.7	56.7	423.0	10.0	95.5
21AR229_73	102.8	5587.473	1.533	17.242	4.4	0.48409	7.1	0.06364	2.0	0.287	397.7	7.9	400.9	23.5	419.9	151.9	397.7	7.9	94.7
21AR229_74	18.1	1002.080	1.801	11.592	17.3	0.56466	21.0	0.05704	4.5	0.214	357.6	15.6	454.6	76.9	981.1	418.6	357.6	15.6	36.5
21AR229_75	247.2	11884.913	6.375	17.056	2.1	0.68084	3.1	0.08611	2.1	0.690	532.5	11.0	527.3	12.8	505.8	49.6	532.5	11.0	105.3
21AR229_76	68.3	1671.902	0.813	13.036	15.5	0.22164	25.0	0.02365	2.7	0.107	150.7	4.0	203.3	46.1	868.8	517.6	150.7	4.0	17.3
21AR229_77	82.6	6920.892	1.761	16.637	5.5	0.66685	6.7	0.08354	1.8	0.273	517.2	9.2	518.8	27.4	526.7	142.3	517.2	9.2	98.2
21AR229_78	53.9	820.947	1.317	14.623	7.2	0.55970	35.5	0.08048	1.8	0.050	499.0	8.5	451.3	129.5	216.1		499.0	8.5	230.9
21AR229_79	221.3	30857.803	1.756	13.924	1.5	1.38506	3.5	0.14153	3.1	0.899	853.3	24.9	882.6	20.4	957.6	31.0	957.6	31.0	89.1
21AR229_80	265.1	11704.009	2.752	18.756	3.8	0.29807	5.2	0.04175	1.8	0.349	263.7	4.7	264.9	12.1	276.7	111.0	263.7	4.7	95.3
21AR229_81	158.1	25069.805	2.269	13.522	1.6	1.49704	2.6	0.14860	1.9	0.752	893.1	16.0	929.2	15.5	1016.7	34.1	1016.7	34.1	87.8
21AR229_82	267.6	17293.796	2.207	17.623	2.0	0.59166	3.7	0.07710	1.9	0.503	478.8	8.7	471.9	14.1	439.6	71.9	478.8	8.7	108.9
21AR229_83	177.9	9043.795	1.644	17.741	4.3	0.33715	7.4	0.04486	2.0	0.262	282.9	5.4	295.0	19.1	393.3	161.3	282.9	5.4	71.9
21AR229_84	126.6	5497.427	1.442	16.808	6.2	0.36560	7.9	0.04685	1.8	0.227	295.2	5.2	316.4	21.6	476.8	171.1	295.2	5.2	61.9
21AR229_85	91.1	4987.495	1.059	17.237	5.2	0.44705	7.8	0.05904	2.1	0.270	369.8	7.6	375.2	24.4	409.7	167.6	369.8	7.6	90.3
21AR229_86	172.8	15689.571	13.045	15.972	2.7	0.79483	4.7	0.09376	2.5	0.528	577.8	13.7	593.9	21.1	657.1	85.3	577.8	13.7	87.9
21AR229_87	247.7	20556.283	7.490	17.049	2.2	0.68486	3.1	0.08583	2.1	0.670	530.8	10.5	529.7	12.7	525.8	50.1	530.8	10.5	101.0
21AR229_88	288.5	1516.855	8.773	14.764	2.6	0.65821	6.7	0.08219	4.9	0.736	509.2	24.1	513.5	27.0	533.9	99.2	509.2	24.1	95.4
21AR229_89	95.2	6514.205	3.349	17.140	4.6	0.65651	5.8	0.08497	2.2	0.379	525.7	11.1	512.5	23.4	454.7	119.3	525.7	11.1	115.6
21AR229_90	171.9	15130.657	10.614	16.014	3.6	0.80491	11.3	0.09507	10.3	0.910	585.4	57.5	599.6	51.1	654.4	100.3	585.4	57.5	89.5
21AR229_91	119.5	7833.097	12.578	17.476	3.6	0.54685	4.4	0.07174	1.7	0.391	446.6	7.4	442.9	15.8	424.7	90.5	446.6	7.4	105.2
21AR229_92	69.9	3333.378	1.688	15.483	8.0	0.47943	15.0	0.05784	2.0	0.137	362.5	7.2	397.7	49.3	608.7	321.1	362.5	7.2	59.6
21AR229_93	244.0	11475.692	21.410	18.613	3.0	0.38099	8.4	0.05272	7.6	0.907	331.2	24.7	327.8	23.6	304.4	80.7	331.2	24.7	108.8
21AR229_94	146.2	31188.963	3.731	12.685	1.3	2.15037	2.7	0.19913	2.3	0.870	1170.7	25.1	1165.2	18.7	1155.8	26.4	1155.8	26.4	101.3
21AR229_95	90.4	3976.586	1.526	17.356	5.1	0.34204	9.6	0.04602	1.9	0.196	290.1	5.3	298.7	24.8	367.8	212.0	290.1	5.3	78.9
21AR229_96	218.6	14120.618	2.896	17.381	2.0	0.56443	3.4	0.07273	1.6	0.477	452.6	7.1	454.4	12.5	464.8	66.2	452.6	7.1	97.4
21AR229_97	161.9	8681.847	2.818	18.800	3.3	0.34997	4.5	0.04932	1.8	0.406	310.4	5.5	304.7	11.8	262.6	94.2	310.4	5.5	118.2
21AR229_98	65.2	10675.597	2.750	13.514	2.6	1.71435	4.1	0.17125	1.6	0.389	1019.0	15.0	1013.9	26.3	1003.9	76.6	1003.9	76.6	101.5
21AR229_99	122.3	6974.543	6.001	17.379	3.1	0.58273	5.7	0.07640	1.5	0.258	474.6	6.7	466.2	21.4	426.1	123.2	474.6	6.7	111.4
21AR229_100	429.6	33816.528	4.186	18.176	1.8	0.46864	2.8	0.06247	1.7	0.604	390.6	6.3	390.2	8.9	389.0	49.3	390.6	6.3	100.4
21AR229_101	59.6	293.259	2.372	8.447	29.0	0.10062	86.3	0.01054	6.7	0.078	67.6	4.5	97.3	80.1	905.9	1858.1	67.6	4.5	7.5
21AR229_102	476.1	77179.442	39.039	13.446	1.5	1.81673	2.4	0.17818	1.8	0.759	1057.0	17.6	1051.5	15.6	1040.9	31.3	1040.9	31.3	101.5
21AR229_103	139.2	24722.315	3.765	13.574	1.8	1.76233	2.8	0.17543	1.8	0.659	1042.0	17.7	1031.7	18.1	1010.9	42.6	1010.9	42.6	103.1
21AR229_104	129.6	19049.501	1.789	14.165	1.9	1.39754	3.1	0.14547	1.9	0.625	875.5	15.6	887.9	18.1	919.7	49.1	875.5	15.6	95.2
21AR229_105	264.0	3283.378	1.721	16.897	2.7	0.56758	9.9	0.07544	1.6	0.163	468.9	7.3	456.5	36.6	395.3	220.1	468.9	7.3	118.6
21AR229_106	403.5	25603.616	12.354	16.810	1.4	0.75734	2.2	0.09352	1.6	0.718	576.3	8.9	572.5	9.8	558.1	34.0	576.3	8.9	103.3
21AR229_107	91.7	15952.833	3.031	13.165	1.8	1.84859	3.1	0.17928	2.0	0.646	1063.0	19.8	1062.9	20.7	1063.6	48.2	1063.6	48.2	99.9
21AR229_108	100.3	10365.974	2.569	13.409	2.3	1.63662	4.6	0.16289	2.8	0.605	972.8	25.2	984.4	29.1	1011.2	74.6	1011.2	74.6	96.2
21AR229_109	422.6	16166.578	1.155	19.101	2.3	0.28680	3.1	0.04061	1.7	0.544	256.6	4.3	256.0	7.1	251.9	60.7	256.6	4.3	101.9
21AR229_110	319.6	35232.083	8.160	14.669	1.8	1.25083	5.7	0.13446	5.4	0.939	813.3	41.2	823.8	32.4	853.2	40.9	813.3	41.2	95.3
21AR229_111	107.6	10027.537	1.405	16.216	3.0	0.88511	5.3	0.10706	1.6	0.311	655.6	10.2	643.7	25.1	603.2	108.3	655.6	10.2	108.7
21AR229_112	45.6	4324.722	1.935	16.007	6.9	0.68805	7.7	0.08478	1.8	0.231	524.6	8.9	531.6	31.8	563.0	162.7	524.6	8.9	93.2
21AR229_113	271.2	21970.690	1.939	18.243	2.2	0.46744	3.2	0.06289	1.4	0.438	393.2	5.4	389.4	10.4	368.1	65.0	393.2	5.4	106.8
21AR229_114	12.3	888.295	1.485	11.283	11.7	1.76692	20.2	0.17727	2.8	0.137	1052.1	26.9	1033.4	131.0	994.9	407.3	994.9	407.3	105.7
21AR229_115	197.0	35989.291	7.285	13.455	1.7	1.77310	2.6	0.17471	1.9	0.729	1038.0	18.2	1035.7	16.9	1031.6	35.9	1031.6	35.9	100.6
21AR229_116	163.1	10231.135	0.690	17.485	3.4	0.50400	4.7	0.06583	2.6	0.547	411.0	10.2	414.4	16.0	434.4	87.7	411.0	10.2	94.6

21AR229_117	54.2	12712.816	1.751	11.910	2.5	2.46683	3.8	0.21686	2.1	0.547	1265.3	23.7	1262.4	27.3	1258.3	61.8	1258.3	61.8	100.6
21AR229_118	366.2	32099.744	5.299	16.212	1.9	0.88614	2.9	0.10543	2.1	0.731	646.1	13.1	644.3	13.9	638.8	42.8	646.1	13.1	101.1
21AR229_119	257.7	18196.152	4.517	18.281	2.4	0.47986	3.2	0.06477	2.0	0.616	404.6	7.7	398.0	10.5	360.9	56.9	404.6	7.7	112.1
21AR229_120	58.8	549.456	2.730	9.661	22.9	0.10569	46.3	0.00990	8.0	0.173	63.5	5.1	102.0	44.9	1132.9	919.1	63.5	5.1	5.6
21AR229_121	356.1	3057.416	15.289	16.722	2.0	0.56832	4.1	0.07513	1.7	0.409	467.0	7.5	456.9	14.9	407.7	82.9	467.0	7.5	114.5
21AR229_122	132.8	39139.315	2.817	9.665	1.3	4.12075	2.2	0.29071	1.7	0.766	1645.1	24.8	1658.4	18.2	1676.2	26.5	1676.2	26.5	98.1
21AR229_123	178.9	28955.311	4.095	13.185	1.4	1.83983	3.4	0.17762	3.0	0.904	1054.0	29.6	1059.8	22.1	1072.8	28.8	1072.8	28.8	98.2
21AR229_124	49.0	489.080	1.637	7.647	28.8	0.14030	37.0	0.00994	6.5	0.176	63.7	4.1	133.3	46.2	1668.8	679.4	63.7	4.1	3.8
21AR229_125	285.8	31951.908	14.762	14.662	2.3	1.21698	5.4	0.13054	4.8	0.891	790.9	35.7	808.4	30.0	857.7	50.7	790.9	35.7	92.2
21AR229_126	95.6	3410.151	1.997	16.831	6.5	0.31881	9.0	0.04202	2.6	0.289	265.4	6.7	281.0	22.0	414.0	191.9	265.4	6.7	64.1
21AR229_127	130.6	1310.070	2.515	11.151	16.4	0.11291	23.2	0.01041	3.3	0.140	66.7	2.2	108.6	23.9	1165.3	455.7	66.7	2.2	5.7
21AR229_128	57.3	7148.515	3.061	13.274	3.0	1.71913	6.2	0.17065	2.1	0.341	1015.7	20.0	1015.7	40.0	1016.6	118.8	1016.6	118.8	99.9
21AR229_129	175.2	9439.717	2.148	17.816	3.3	0.44727	4.4	0.05955	2.4	0.558	372.9	8.8	375.4	13.7	391.6	81.1	372.9	8.8	95.2
21AR229_130	292.5	30240.465	2.071	15.874	1.5	0.97615	2.8	0.11347	2.1	0.734	692.9	13.6	691.6	14.2	688.5	41.0	692.9	13.6	100.6
21AR229_131	385.4	37963.989	4.014	17.015	1.4	0.69970	3.0	0.08701	2.4	0.825	537.8	12.6	538.6	12.4	542.9	36.7	537.8	12.6	99.1
21AR229_132	206.2	16461.746	6.213	12.096	1.8	1.00149	3.2	0.08896	2.4	0.751	549.4	12.8	704.6	16.4	1238.0	41.8	549.4	12.8	44.4
21AR229_133	168.1	3944.592	2.759	18.073	5.6	0.21131	12.8	0.02975	2.0	0.154	189.0	3.7	194.6	22.6	264.5	289.5	189.0	3.7	71.5
21AR229_134	59.2	4683.951	2.032	16.047	7.3	0.63997	8.5	0.07854	1.9	0.222	487.4	8.9	502.3	33.8	571.7	181.0	487.4	8.9	85.2
21AR229_135	74.6	4846.017	0.805	16.424	4.1	0.77480	10.9	0.09727	1.6	0.144	598.4	9.0	582.5	48.3	522.1	236.8	598.4	9.0	114.6
21AR229_136	110.1	6319.451	0.300	17.891	4.9	0.46263	6.5	0.06269	1.6	0.246	391.9	6.0	386.1	20.7	352.1	141.3	391.9	6.0	111.3
21AR229_137	277.3	13541.790	2.000	18.506	3.3	0.29048	5.2	0.03989	1.4	0.268	252.2	3.5	258.9	12.0	321.7	114.9	252.2	3.5	78.4
21AR229_138	214.9	34640.016	7.658	13.461	1.4	1.72365	2.1	0.17002	1.5	0.714	1012.2	14.1	1017.4	13.6	1029.4	29.9	1029.4	29.9	98.3
21AR229_139	154.3	6075.039	2.766	17.268	5.5	0.30727	6.8	0.04030	2.2	0.317	254.7	5.4	272.1	16.2	425.1	143.6	254.7	5.4	59.9
21AR229_140	70.2	3529.755	1.227	15.526	6.1	0.49615	12.9	0.05984	2.0	0.152	374.6	7.1	409.1	43.4	609.4	275.7	374.6	7.1	61.5
21AR229_141	131.9	3442.638	1.614	15.248	6.8	0.25397	9.1	0.03009	1.6	0.173	191.1	3.0	229.8	18.6	647.4	191.8	191.1	3.0	29.5
21AR229_142	114.0	9252.449	1.901	17.309	3.5	0.66198	6.0	0.08566	1.6	0.267	529.8	8.1	515.8	24.2	455.3	128.0	529.8	8.1	116.4
21AR229_143	56.9	642.231	2.706	8.228	24.3	0.13976	38.5	0.01016	5.3	0.137	65.1	3.4	132.8	48.0	1621.1	716.8	65.1	3.4	4.0
21AR229_144	483.8	8153.338	10.944	17.943	1.8	0.45718	6.2	0.06171	1.6	0.263	386.0	6.1	382.3	19.6	360.7	133.9	386.0	6.1	107.0
21AR229_145	197.0	77083.297	2.583	8.213	1.3	5.97204	2.8	0.35785	2.4	0.876	1972.0	40.9	1971.8	23.9	1972.3	23.6	1972.3	23.6	100.0
21AR229_146	172.0	7798.237	2.020	18.140	4.9	0.34015	6.4	0.04654	2.0	0.314	293.2	5.8	297.3	16.5	330.2	137.5	293.2	5.8	88.8
21AR229_147	57.2	2191.632	1.029	15.926	6.8	0.36258	19.4	0.04705	2.1	0.108	296.4	6.1	314.1	52.3	448.9	428.5	296.4	6.1	66.0
21AR229_148	349.6	27244.386	22.901	18.150	2.8	0.48075	4.3	0.06420	2.9	0.672	401.1	11.1	398.6	14.0	385.0	70.7	401.1	11.1	104.2
21AR229_149	269.6	3147.002	1.162	17.185	2.7	0.43824	15.0	0.05957	2.9	0.191	373.0	10.4	369.0	46.4	344.8	333.2	373.0	10.4	108.2
21AR229_150	26.0	2667.835	4.096	15.262	8.5	0.73285	10.7	0.08897	2.4	0.223	549.5	12.5	558.2	45.9	595.1	225.7	549.5	12.5	92.3
21AR229_151	104.2	6123.750	2.396	17.925	4.4	0.47762	5.6	0.06526	1.7	0.302	407.5	6.6	396.4	18.2	333.2	120.0	407.5	6.6	122.3
21AR229_152	31.8	1487.204	0.491	11.311	19.8	0.42453	22.0	0.03926	4.6	0.208	248.3	11.2	359.3	66.6	1158.3	428.1	248.3	11.2	21.4
21AR229_153	240.6	14018.339	1.399	18.139	2.3	0.45546	4.5	0.06144	1.7	0.383	384.4	6.4	381.1	14.3	362.1	93.9	384.4	6.4	106.2
21AR229_154	409.6	33035.104	0.963	17.544	1.8	0.59339	3.2	0.07655	2.5	0.762	475.5	11.3	473.0	12.2	462.0	46.5	475.5	11.3	102.9
21AR229_155	627.0	43209.555	0.971	17.725	1.6	0.58286	2.6	0.07583	1.8	0.712	471.2	8.3	466.3	9.6	443.1	39.9	471.2	8.3	106.3
21AR229_156	135.0	2871.712	2.050	16.744	7.5	0.15276	12.5	0.02040	2.2	0.175	130.2	2.8	144.3	16.8	384.5	276.8	130.2	2.8	33.9
21AR229_157	247.9	22905.223	6.088	17.336	2.1	0.65129	3.8	0.08330	2.8	0.726	515.8	13.7	509.3	15.3	481.2	58.1	515.8	13.7	107.2
21AR229_158	264.7	9736.193	1.460	18.730	3.1	0.28591	4.1	0.04021	1.3	0.330	254.1	3.4	255.3	9.2	267.6	88.4	254.1	3.4	95.0
21AR229_159	139.8	11966.114	3.821	16.416	2.9	0.73235	5.0	0.08928	3.5	0.694	551.3	18.3	557.9	21.5	586.1	78.2	551.3	18.3	94.1
21AR229_160	86.0	4007.920	1.889	15.872	7.9	0.35011	9.6	0.04302	2.3	0.235	271.5	6.0	304.8	25.2	569.1	202.3	271.5	6.0	47.7
21AR229_161	124.6	5201.313	1.233	16.373	7.2	0.32564	8.5	0.04077	2.1	0.245	257.6	5.2	286.2	21.1	528.2	180.2	257.6	5.2	48.8
21AR229_162	232.7	28356.229	19.380	13.979	3.2	1.41890	11.0	0.14575	10.5	0.956	877.1	86.3	896.9	65.6	947.0	65.8	947.0	65.8	92.6
21AR229_163	146.9	3839.154	3.887	13.327	1.8	1.56704	10.6	0.16015	2.9	0.277	957.6	26.1	957.3	65.6	957.3	208.1	957.3	208.1	100.0
21AR229_164	144.8	24881.303	4.639	13.322	1.5	1.85587	3.5	0.18159	2.9	0.813	1075.7	28.4	1065.5	23.2	1045.7	41.4	1045.7	41.4	102.9
21AR229_165	414.7	16195.951	2.061	19.265	2.5	0.27835	4.3	0.03978	1.8	0.413	251.5	4.4	249.3	9.6	230.4	91.4	251.5	4.4	109.1
21AR229_166	190.9	15245.335	3.810	17.453	2.9	0.59145	4.3	0.07652	1.5	0.357	475.3	7.1	471.8	16.3	455.8	89.7	475.3	7.1	104.3
21AR229_167	55.8	2761.291	1.246	15.651	7.2	0.33709	16.7	0.04195	2.4	0.146	264.9	6.4	295.0	42.9	541.3	362.8	264.9	6.4	48.9
21AR229_168	444.6	7180.722	13.662	13.281	1.3	1.73249	5.1	0.17235	1.5	0.299	1025.0	14.6	1020.7	33.1	1012.2	99.4	1012.2	99.4	101.3
21AR229_169	264.3	18592.732	17.652	17.545	3.1	0.55146	5.1	0.07155	3.5	0.678	445.5	14.9	446.0	18.5	449.2	83.5	445.5	14.9	99.2
21AR229_170	38.8	2038.677	0.832	14.931	9.6	0.49687	11.9	0.06041	2.5	0.207	378.1	9.0	409.6	40.1	591.8	252.7	378.1	9.0	63.9
21AR229_171	241.8	2525.587	2.559	18.074	4.9	0.20018	17.6	0.02949	2.3	0.128	187.4	4.2	185.3	29.9	159.9		187.4	4.2	117.2
21AR229_172	235.8	22887.208	16.838	15.893	1.6	0.90983	2.5	0.10649	1.7	0.660	652.3	10.4	657.0	12.2	673.9	40.6	652.3	10.4	96.8
21AR229_173	130.0	11222.494	1.789	17.085	3.4	0.68184	4.1	0.08685	1.5	0.372	536.9	7.9	527.9	17.0	490.2	84.6	536.9	7.9	109.5
21AR229_174	199.0	6429.073	1.059	18.327	4.5	0.24530	6.5	0.03420	1.8	0.278	216.8	3.8	222.7	12.9	287.0	141.7	216.8	3.8	75.5
21AR229_175	199.7	15145.628	4.066	17.014	2.1	0.68282	3.7	0.08610	1.9	0.512	532.5	9.6	528.5	15.1	512.3	69.4	532.5	9.6	103.9
21AR229_176	89.7	5144.248	1.311	17.217	5.5	0.37122	7.7	0.04897	1.7	0.218	308.2	5.0	320.6	21.1	412.1	167.3	308.2	5.0	74.8
21AR229_177	264.5	20799.029	2.905	18.224	2.5	0.47652	3.3	0.06411	1.9	0.562	400.6	7.2	395.7	10.8	368.1	61.5	400.6	7.2	108.8
21AR229_178	26.9	233.555	1.840	4.941	30.6	0.19071	50.0	0.00945	13.5	0.271	60.6	8.2	177.2	81.4	2305.2	840.3	60.6	8.2	2.6
21AR229_179	176.1	4032.117	0.750	18.192	4.8	0.28420	8.9	0.04035	2.4	0.273	255.0	6.1	254.0	20.0	245.5	197.5	255.0	6.1	103.9
21AR229_180	201.4	8315.096	1.067	18.743	2.9	0.27784	6.4	0.03920	2.0	0.317	247.9	5.0	248.9	14.2	260.1	140.1	247.9	5.0	95.3
21AR229_181	54.5	3185.703	0.853	16.663	6.9	0.48971	8.8	0.06424	2.0	0.227	401.4	7.8	404.7	29.5	424.8	192.1	401.4	7.8	94.5
21AR229_182	80.8	3778.769	2.117	16.200	8.5	0.33861	11.8	0.04263	2.1	0.178	269.1	5.5	296.1	30.4	515.9	255.7	269.1	5.5	52.2
21AR229_183	125.1	68648.390	3.428	5.946	1.3	9.77324	3.1	0.42365	2.8	0.907	2277.0	53.6	2413.6	28.4	2531.7	21.8	2531.7	21.8	89.9
21AR229_184	136.6	2985.023	1.581	15.881	9.0	0.15652	14.5	0.01962	2.1	0.145	125.3	2.6	147.7	20.0	525.0	315.5	125.3	2.6	23.9
21AR229_185	88.2	5871.998	2.258	16.836	4.4	0.59184	7.2	0.07573	1.7	0.228	470.6	7.5	472.0	27.3	480.2	155.8	470.6	7.5	98.0
21AR229_186	210.5	18039.625	2.628	17.428	2.2	0.63527	3.5	0.08178	2.1	0.609	506.8	10.3	499.4	13.7	466.6	60.9	506.8	10.3	108.6
21AR229_187	162.4	10873.704	1.528	17.679	3.1	0.58697	3.9	0.07741	2.0	0.505	480.6	9.1	468.9	14.6	412.9	75.1	480.6	9.1	116.4
21AR229_188	363.5	24089.206	1.527	18.206	1.9	0.49121	3.0	0.06586	1.7	0.566	411.2	6.8	405.7	10.1	375.9	55.8	411.2	6.8	109.4
21AR229_189	36.8	7174.561	2.677	11.841	3.3	2.53543	4.5	0.22394	1.8	0.412	1302.7	21.7	1282.3	32.5	1249.0	79.5	1249.0	79.5	104.3
21AR229_190	324.0	447.342	1.755	7.400	22.9	0.01881	34.8	0.00131	8.1	0.234	8.4	0.7	18.9	6.5	1697.5	627.2	8.4	0.7	0.5
21AR229_191	135.1	12553.272	3.770	14.184	2.2	1.30195	5.3	0.13677	4.4	0.829	826.4	34.3	846.6	30.6	900.8	61.5	826.4	34.3	91.7
21AR229_192	81.3	8919.418	4.835	14.656	3.1	1.10711	5.1	0.12090	3.8	0.750	735.7	26.4	756.8	27.0	820.4	69.9	735.7	26.4	89.7
21AR229_193	278.9	16466.889	1.968	18.004	2.3	0.48759	3.3	0.06500	2.2	0.663	406.0	8.6	403.3	11.0	388.7	55.5	406.0	8.6	104.4
21AR229_194	70.9	7658.006	1.939	16.511	4.9	0.71680	6.9	0.08902	1.9	0.276	549.7	10.0	548.8	29.2	545.7	144.7	549.7	10.0	100.7
21AR229_195	58.7	4686.310	1.591	16.820	5.5	0.58713	12.4	0.07591	1.9	0.154	471.7	8.7	469.0	46.6	457.0	271.9	471.7	8.7	103.2
21AR229_196	79.7	11735.161	1.383	14.573	2.5	1.25608	4.6	0.13582	3.4	0.735	821.0	25.9	826.1	25.8	841.1	64.5	821.0	25.9	97.6
21AR229_197	31.2	8363.008	0.813	9.140	2.3	4.78823	3.6	0.32376	2.0	0.557	1808.0	31.5	1782.8	30.2	1754.2	54.6	1754.2	54.6	103.1
21AR229_198	358.0	39384.963	5.208	14.517	2.3	1.12188	9.0	0.11932	8.7	0.965	726.6	59.9	763.9	48.5	875.4	48.9	726.6	59.9	83.0
21AR229_199	264.3	43234.833	3.262	13.694	1.6	1.45419	4.6	0.14578	4.3	0.931	877.3	34.9	911.6	27.5	996.6	33.8	996.6	33.8	88.0
21AR229_200	326.1	17356.446	69.382	18.486	2.5	0.42674	3.2	0.05841	1.7	0.534	365.9	6.1	360.9	9.8	329.3	61.9	365.9	6.1	111.1
21AR229_201	154.0	10259.526	1.599	17.660	3.1	0.57465	4.3	0.07577	1.7	0.399	470.8	7.8	461.0	16.0	413.4	88.3	470.8	7.8	113.9
21AR229_202	310.7	24955.282	2.814	13.586	1.2	1.69950	2.3	0.16937	1.9	0.815	1008.6	17.6	1008.3	14.8	1008.5	27.1	1008.5	27.1	100.0
21AR229_203	569.9	29367.532	7.833	15.068	1.2	1.03158	2.6	0.11397	2.2	0.853	695.7	14.5	719.7	13.3	796.1	28.3	695.7	14.5	87.4
21AR229_204	221.1	13185.618	10.003	17.524	2.4	0.63373	3.3	0.08235	1.8	0.537	510.2	8.7	498.4	13.0	445.8	61.9	510.2	8.7	114.4
21AR229_205	208.8	4956.614	1.178	17.912	4.9	0.30361	8.3	0.04172	2.2	0.261	263.5	5.6	269.2	19.5	320.3	181.2	263.5	5.6	82.3
21AR229_206	437.2	6893.080	1.886	11.078	1.5	2.82192	4.4	0.23236	1.8	0.413	1346.8	22.0	1361.4	32.9	1385.1	76.7	1385.1	76.7	97.2
21AR229_207	140.9	7575.899	11.033	17.643	3.3	0.48234	5.2	0.06401	1.9	0.373	400.0	7.6	399.7	17.2	399.0	108.4	400.0	7.6	100.2
21AR229_208	81.4	2047.318	1.192	15.129	7.8	0.24018	13.0	0.02958	2.6	0.199	187.9	4.8	218.6	25.5	563.9	277.0	187.9	4.8	33.3
21AR229_209	59.2	747.871	1.979	8.893	22.0	0.13312	29.3	0.01031	5.7	0.194	66.2	3.8	126.9	35.0	1501.0	546.3	66.2	3.8	4.4
21AR229_210	51.6	2492.355	0.844	14.843	9.7	0.34421	14.6	0.04063	2.5	0.172	256.7	6.3	300.4	37.9	655.9	308.3	256.7	6.3	39.1
21AR229_211	267.4	36356.599	8.555	14.460	1.7	1.26140	4.0	0.13330	3.6	0.895	806.6	27.4	828.5	22.9	888.7	37.3	806.6	27.4	90.8
21AR229_212	163.2	1710.039	2.848	13.135	11.7	0.09800	19.9	0.01052	3.3	0.165	67.4	2.2	94.9	18.1	856.9	409.1	67.4	2.2	7.9
21AR229_213	43.1	3313.081	0.406	16.064	7.7	0.51592	16.7	0.06478	2.2	0.134	404.7	8.8	422.4	57.9	521.5	364.7	404.7	8.8	77.6
21AR229_214	79.1	3482.371	1.722	16.826	7.4	0.37434	12.6	0.04932	2.3	0.186	310.3	7.1	322.9	34.8	415.2	276.7	310.3	7.1	74.7
21AR229_215	131.0	7086.770	2.576	17.302	4.5	0.38102	6.4	0.04961	2.0	0.305	312.1	6.0	327.8	18.0	441.7	136.0	312.1	6.0	70.7
21AR229_216	130.0	35051.129	8.631	12.612	2.3	2.06914	5.4	0.19045	4.8	0.900	1123.8	50.0	1138.6	36.9	1167.9	46.5	1167.9	46.5	96.2
21AR229_217	144.9	14023.938	7.227	17.012	3.0	0.67066	3.4	0.08433	1.4	0.426	521.9	7.2	521.1	13.9	518.6	67.5	521.9	7.2	100.6
21AR229_218	143.7	10397.116	1.288	17.413	2.9	0.59080	4.4	0.07656	1.9	0.432	475.6	8.7	471.4	16.6	451.9	88.0	475.6	8.7	105.2
21AR229_219	29.4	2761.874	1.500	14.354	8.2	0.77306	13.1	0.08709	2.2	0.164	538.3	11.2	581.5	58.2	755.0	274.0	538.3	11.2	71.3
21AR229_220	22.5	863.591	1.509	9.507	19.8	0.38617	29.3	0.03150	6.4	0.218	200.0	12.6	331.6	82.9	1402.9	550.2	200.0	12.6	14.3
21AR229_221	72.4	11977.290	2.800	13.078	2.3	1.93662	3.4	0.18701	2.0	0.584	1105.2	20.2	1093.8	22.7	1072.2	55.4	1072.2	55.4	103.1
21AR229_222	202.0	8908.011	3.522	19.042	3.1	0.31457	4.1	0.04490	1.8	0.442	283.1	5.0	277.7	10.0	233.3	85.1	283.1	5.0	121.4
21AR229_223	208.3	14297.999	2.474	17.890	2.0	0.58712	3.3	0.07768	1.5	0.443	482.3	6.7	469.0	12.3	405.7	65.7	482.3	6.7	118.9
21AR229_224	118.3	4151.039	1.715	18.001	5.4	0.31193	7.3	0.04354	2.2	0.299	274.7	5.9	275.7	17.7	284.8	160.0	274.7	5.9	96.4
21AR229_225	53.5	1413.904	1.917	14.400	10.4	0.34907	23.6	0.04293	2.7	0.115	271.0	7.2	304.0	61.9	567.0	511.0	271.0	7.2	47.8
21AR229_226	95.9	2279.679	1.918	14.320	11.6	0.22757	13.6	0.02602	2.1	0.152	165.6	3.4	208.2	25.5	723.3	284.7	165.6	3.4	22.9
21AR229_227	42.1	9201.305	1.542	11.298	3.7	2.76261	4.4	0.23059	1.9	0.438	1337.6	23.4	1345.5	33.0	1358.9	76.8	1358.9	76.8	98.4
21AR229_228	235.1	1833.187	1.809	17.326	3.7	0.28661	11.7	0.04188	1.9	0.161	264.4	4.9	255.9	26.5	179.1	269.6	264.4	4.9	147.6
21AR229_229	126.9	12780.730	3.766	14.295	2.1	1.26532	4.6	0.13354	3.6	0.783	808.0	27.5	830.3	26.2	891.3	59.5	808.0	27.5	90.7
21AR229_230	349.0	15634.171	24.351	11.500	2.3	2.15719	5.0	0.18210	4.4	0.877	1078.4	43.5	1167.4	34.6	1337.2	46.3	1337.2	46.3	80.6
21AR229_231	233.6	8653.200	1.876	18.463	3.5	0.29385	5.4	0.04068	1.8	0.334	257.1	4.6	261.6	12.5	303.2	116.4	257.1	4.6	84.8
21AR229_232	173.2	34924.782	3.642	12.569	1.7	2.21187	2.6	0.20300	1.9	0.740	1191.4	20.8	1184.8	18.1	1173.7	34.4	1173.7	34.4	101.5

21AR229_233	196.7	17066.948	1.421	17.484	2.5	0.63728	3.7	0.08223	2.5	0.671	509.4	12.1	500.6	14.5	461.5	60.4	509.4	12.1	110.4
21AR229_234	113.5	27248.003	2.810	11.528	1.5	2.73191	2.5	0.23017	1.9	0.741	1335.4	22.5	1337.2	18.7	1340.9	32.6	1340.9	32.6	99.6
21AR229_235	263.4	21730.509	4.235	16.524	1.4	0.83815	2.3	0.10174	1.7	0.735	624.6	10.3	618.1	10.9	595.4	34.5	624.6	10.3	104.9
21AR229_236	198.7	8901.218	2.196	18.297	2.8	0.47530	5.4	0.06526	2.3	0.426	407.5	9.0	394.8	17.5	322.3	110.0	407.5	9.0	126.4
21AR229_237	201.5	15781.949	11.205	17.289	2.2	0.65899	2.9	0.08417	1.5	0.530	521.0	7.6	514.0	11.6	484.1	54.0	521.0	7.6	107.6
21AR229_238	59.9	658.335	2.403	8.425	26.3	0.13970	28.8	0.01039	4.6	0.161	66.6	3.1	132.8	35.8	1578.1	534.3	66.6	3.1	4.2
21AR229_239	194.9	17432.231	5.122	16.456	2.0	0.72087	3.8	0.08738	2.7	0.701	540.0	13.8	551.2	16.2	598.6	58.8	540.0	13.8	90.2
21AR229_240	157.9	4287.266	1.738	17.019	6.2	0.19828	10.5	0.02603	2.3	0.224	165.6	3.8	183.7	17.6	423.6	227.7	165.6	3.8	39.1
21AR229_241	229.5	39036.615	51.785	16.489	1.9	0.85677	2.8	0.10327	1.8	0.633	633.5	10.9	628.4	13.3	610.7	47.6	633.5	10.9	103.7
21AR229_242	288.3	9438.016	14.077	17.894	3.3	0.46401	6.6	0.06202	2.9	0.440	387.9	10.9	387.0	21.1	382.8	132.4	387.9	10.9	101.3
21AR229_243	107.3	20527.942	2.321	12.353	1.5	2.34980	2.3	0.21225	1.5	0.649	1240.8	17.2	1227.5	16.7	1205.0	35.1	1205.0	35.1	103.0
21AR229_244	83.2	8670.162	2.194	16.807	4.1	0.68305	5.7	0.08564	1.4	0.247	529.7	7.1	528.6	23.4	524.8	120.5	529.7	7.1	100.9
21AR229_245	263.1	8268.441	2.980	18.276	4.6	0.20798	5.2	0.02848	1.5	0.294	181.0	2.7	191.9	9.0	328.2	112.0	181.0	2.7	55.2
21AR229_246	35.7	6983.111	1.140	8.727	1.9	5.06572	3.0	0.32726	1.8	0.598	1825.1	28.9	1830.4	25.8	1837.2	44.2	1837.2	44.2	99.3
21AR229_247	175.4	12242.097	3.572	13.273	1.3	1.82288	2.1	0.17791	1.4	0.651	1055.6	13.4	1053.7	13.9	1050.8	32.3	1050.8	32.3	100.5
21AR229_248	142.8	2282.416	1.897	16.074	8.0	0.16003	15.1	0.02076	1.8	0.120	132.4	2.4	150.7	21.2	450.0	334.4	132.4	2.4	29.4
21AR229_249	168.4	14109.861	0.976	17.675	3.2	0.57451	3.9	0.07488	1.6	0.410	465.5	7.2	460.9	14.4	439.1	78.8	465.5	7.2	106.0
21AR229_250	195.8	3248.002	1.567	17.198	7.3	0.13190	9.5	0.01780	2.0	0.208	113.8	2.2	125.8	11.3	360.9	210.3	113.8	2.2	31.5
21AR229_251	73.9	529.757	2.559	8.561	20.7	0.13296	51.1	0.01064	4.5	0.087	68.2	3.0	126.8	61.0	1439.1	987.6	68.2	3.0	4.7
21AR229_252	98.2	1321.623	1.062	14.391	13.3	0.12774	26.2	0.01582	2.5	0.094	101.2	2.5	122.1	30.2	552.4	572.5	101.2	2.5	18.3
21AR229_253	285.1	37555.918	5.339	16.831	1.9	0.79573	2.8	0.09774	2.1	0.733	601.1	12.0	594.4	12.8	569.8	42.1	601.1	12.0	105.5
21AR229_254	98.6	12770.677	2.506	14.158	2.6	1.42140	6.6	0.14804	5.8	0.882	889.9	48.0	897.9	39.1	918.6	63.4	918.6	63.4	96.9
21AR229_255	269.8	11154.169	1.686	18.651	3.3	0.29708	4.4	0.04112	2.0	0.450	259.8	5.1	264.1	10.3	303.8	89.9	259.8	5.1	85.5
21AR229_256	176.0	11669.590	1.063	18.096	3.2	0.49200	4.1	0.06595	1.8	0.445	411.7	7.3	406.3	13.7	376.4	82.7	411.7	7.3	109.4
21AR229_257	73.7	4959.988	0.704	16.062	7.7	0.52784	8.5	0.06474	2.2	0.255	404.4	8.5	430.4	29.8	572.8	178.9	404.4	8.5	70.6
21AR229_258	76.4	4465.991	1.684	17.178	6.2	0.50451	7.2	0.06647	2.0	0.283	414.9	8.2	414.8	24.5	415.1	154.4	414.9	8.2	99.9
21AR229_259	128.6	21695.061	3.295	13.333	1.5	1.82629	2.5	0.17784	1.6	0.653	1055.2	16.0	1054.9	16.6	1055.3	38.5	1055.3	38.5	100.0
21AR229_260	113.2	8180.831	1.049	17.553	4.0	0.46706	5.7	0.06114	2.2	0.389	382.6	8.2	389.2	18.4	429.6	116.9	382.6	8.2	89.1
21AR229_261	170.2	22632.728	3.902	15.093	1.6	1.20330	2.6	0.13295	1.5	0.585	804.7	11.5	802.1	14.5	795.9	44.4	804.7	11.5	101.1
21AR229_262	182.7	9357.266	3.746	17.898	4.4	0.31860	5.9	0.04253	1.5	0.254	268.5	4.0	280.8	14.5	385.6	128.7	268.5	4.0	69.6
21AR229_263	190.9	59129.254	0.755	8.645	1.1	5.38896	2.0	0.33846	1.7	0.847	1879.3	27.6	1883.1	17.1	1888.1	19.1	1888.1	19.1	99.5
21AR229_264	227.5	19862.333	17.473	16.859	1.5	0.73236	3.2	0.09097	2.0	0.635	561.2	11.0	557.9	13.8	545.4	54.3	561.2	11.0	102.9
21AR229_265	106.5	8068.597	1.588	17.230	3.2	0.67524	7.1	0.08725	1.7	0.238	539.3	8.8	523.9	29.2	458.4	154.0	539.3	8.8	117.6
21AR229_266	76.3	30073.513	1.337	8.670	1.8	5.41523	2.9	0.34249	2.2	0.768	1898.6	36.4	1887.3	24.7	1875.6	33.3	1875.6	33.3	101.2
21AR229_267	87.6	7498.692	1.924	16.387	3.2	0.68314	7.6	0.08410	2.3	0.303	520.5	11.5	528.7	31.2	564.9	157.3	520.5	11.5	92.1
21AR229_268	82.1	6426.089	1.064	16.564	4.6	0.67395	8.7	0.08439	1.8	0.206	522.3	9.0	523.1	35.5	527.8	186.4	522.3	9.0	99.0
21AR229_269	39.0	1967.928	0.796	13.921	11.6	0.51125	16.7	0.05770	2.1	0.127	361.6	7.5	419.3	57.5	751.1	351.4	361.6	7.5	48.2
21AR229_270	152.8	13166.856	1.718	16.224	2.4	0.89124	3.2	0.10719	1.7	0.524	656.4	10.3	647.0	15.1	615.3	58.1	656.4	10.3	106.7
21AR229_271	56.5	11264.051	2.989	13.486	3.2	1.70562	4.7	0.17038	3.1	0.657	1014.2	29.0	1010.6	30.1	1003.8	72.0	1003.8	72.0	101.0
21AR229_272	207.3	13999.922	1.549	17.892	2.8	0.48965	3.9	0.06500	2.5	0.638	406.0	9.8	404.7	13.1	398.3	67.5	406.0	9.8	101.9
21AR229_273	246.9	72037.082	26.403	5.769	1.1	11.39681	1.9	0.47919	1.5	0.802	2523.7	31.4	2556.2	17.5	2582.7	18.7	2582.7	18.7	97.7
21AR229_274	30.7	5786.706	1.070	12.470	4.5	2.16570	6.0	0.20283	1.8	0.303	1190.5	19.9	1170.1	41.9	1133.4	114.3	1133.4	114.3	105.0
21AR229_275	235.1	10482.737	1.234	19.223	2.8	0.28281	4.3	0.04068	1.6	0.360	257.0	3.9	252.9	9.6	215.5	92.9	257.0	3.9	119.3
21AR229_276	87.5	6175.129	1.660	17.387	4.3	0.60937	6.4	0.08045	1.9	0.297	498.8	9.2	483.2	24.7	410.5	137.2	498.8	9.2	121.5
21AR229_277	134.3	4894.439	1.680	18.666	4.5	0.27388	9.0	0.03943	2.0	0.220	249.3	4.8	245.8	19.7	213.2	203.6	249.3	4.8	116.9
21AR229_278	215.0	2157.221	0.943	14.922	9.8	0.10365	12.7	0.01251	2.2	0.174	80.1	1.8	100.1	12.1	608.3	269.9	80.1	1.8	13.2
21AR229_279	413.7	9155.130	2.881	17.356	1.6	0.56240	3.4	0.07307	1.8	0.541	454.6	8.0	453.1	12.3	446.2	63.0	454.6	8.0	101.9
21AR229_280	120.0	3810.806	2.235	17.391	4.8	0.43652	6.1	0.05923	1.9	0.312	370.9	6.9	367.8	19.0	349.2	132.0	370.9	6.9	106.2
21AR229_281	325.4	38926.528	2.650	12.844	1.1	2.00550	2.1	0.18844	1.7	0.803	1112.9	17.2	1117.4	14.2	1126.9	25.0	1126.9	25.0	98.8
21AR229_282	213.4	12534.072	2.386	18.522	2.4	0.45632	5.0	0.06290	1.6	0.319	393.3	6.1	381.7	15.9	313.2	108.0	393.3	6.1	125.6
21AR229_283	55.8	4606.521	1.472	15.889	6.5	0.68994	9.9	0.08402	3.0	0.301	520.1	14.9	532.8	40.9	588.5	204.3	520.1	14.9	88.4
21AR229_284	363.9	67684.277	2.218	13.092	1.3	1.91070	2.3	0.18264	1.8	0.779	1081.4	17.6	1084.8	15.1	1092.6	28.6	1092.6	28.6	99.0
21AR229_285	68.2	3632.277	1.315	16.650	6.1	0.47377	7.7	0.06154	1.9	0.250	385.0	7.2	393.8	25.0	446.6	165.0	385.0	7.2	86.2
21AR229_286	65.7	13993.534	3.881	12.316	1.9	2.38895	2.9	0.21696	1.9	0.666	1265.8	22.3	1239.3	20.8	1194.4	42.8	1194.4	42.8	106.0
21AR229_287	308.0	21700.711	5.245	18.567	2.4	0.48822	3.3	0.06676	1.8	0.542	416.6	7.3	403.7	11.1	331.5	63.6	416.6	7.3	125.7
**21AR234**																			
21AR234_1	56.0	2531.869	1.655	10.694	12.8	0.57247	14.8	0.04737	3.5	0.235	298.4	10.1	459.6	54.7	1375.5	276.9	298.4	10.1	21.7
21AR234_2	172.1	10262.301	1.544	17.244	3.6	0.58968	4.7	0.07579	1.6	0.351	470.9	7.4	470.7	17.5	470.3	96.6	470.9	7.4	100.1
21AR234_3	392.1	26495.296	1.848	17.356	2.4	0.67290	3.0	0.08541	1.6	0.518	528.3	7.9	522.5	12.3	497.9	56.6	528.3	7.9	106.1
21AR234_4	180.4	3056.606	2.295	14.744	11.6	0.17090	14.9	0.01962	2.9	0.194	125.3	3.6	160.2	22.1	714.9	310.9	125.3	3.6	17.5
21AR234_5	237.0	2334.294	1.798	16.603	5.1	0.29952	9.4	0.04020	1.6	0.168	254.1	3.9	266.0	21.9	373.6	207.9	254.1	3.9	68.0
21AR234_6	496.4	6698.385	1.359	13.863	1.3	1.31370	3.8	0.13594	3.5	0.927	821.7	27.1	851.8	21.9	931.9	29.2	821.7	27.1	88.2
21AR234_7	153.3	9940.770	1.236	16.881	3.9	0.70029	5.3	0.08780	1.7	0.324	542.5	8.9	539.0	22.2	525.0	110.1	542.5	8.9	103.3
21AR234_8	647.2	24710.696	1.626	19.194	2.5	0.28210	3.1	0.03967	1.6	0.511	250.8	3.9	252.3	6.9	267.4	60.8	250.8	3.9	93.8
21AR234_9	207.5	7346.398	2.685	16.351	6.9	0.32114	8.7	0.03931	1.7	0.191	248.5	4.0	282.8	21.4	577.4	185.1	248.5	4.0	43.0
21AR234_10	25.9	1818.376	0.708	8.978	25.1	0.92412	25.7	0.06446	3.7	0.145	402.7	14.5	664.5	125.3	1697.1	469.9	1697.1	469.9	23.7
21AR234_11	407.0	35259.059	3.484	15.284	1.5	1.16371	3.1	0.12953	2.5	0.798	785.2	18.3	783.7	16.9	780.3	39.3	785.2	18.3	100.6
21AR234_12	162.8	21781.025	2.553	13.442	2.1	1.73108	2.7	0.17069	1.5	0.570	1015.9	14.5	1020.2	17.5	1030.1	45.1	1030.1	45.1	98.6
21AR234_13	137.4	3966.078	0.682	15.461	7.8	0.31287	10.8	0.03716	2.0	0.188	235.2	4.7	276.4	26.0	642.2	227.2	235.2	4.7	36.6
21AR234_14	168.7	25447.479	7.571	13.647	1.7	1.75872	2.6	0.17532	1.9	0.719	1041.4	18.1	1030.4	16.9	1008.0	36.8	1008.0	36.8	103.3
21AR234_15	263.9	13591.104	2.067	17.807	4.1	0.45808	4.7	0.06028	1.8	0.385	377.3	6.6	382.9	15.0	417.7	96.8	377.3	6.6	90.3
21AR234_16	426.9	35576.567	4.360	16.910	2.4	0.69438	4.8	0.08580	4.1	0.847	530.7	20.8	535.4	20.0	556.7	55.8	530.7	20.8	95.3
21AR234_17	251.6	14640.317	2.189	17.505	2.8	0.59600	3.6	0.07704	1.9	0.531	478.5	8.8	474.7	13.6	457.5	67.2	478.5	8.8	104.6
21AR234_18	302.8	14280.982	13.937	17.580	3.2	0.46583	4.3	0.06051	2.1	0.493	378.7	7.9	388.3	14.0	446.8	84.1	378.7	7.9	84.8
21AR234_19	171.3	8805.199	1.313	17.385	5.4	0.36864	6.7	0.04788	2.1	0.310	301.5	6.1	318.6	18.2	447.0	140.7	301.5	6.1	67.5
21AR234_20	388.6	389.783	1.706	3.917	79.7	0.03022	83.5	0.00097	17.3	0.207	6.3	1.1	30.2	24.9	3023.7	1378.5	6.3	1.1	0.2
21AR234_21	188.3	10310.954	1.191	17.603	4.5	0.47585	7.4	0.06239	2.8	0.386	390.1	10.8	395.2	24.1	426.0	151.8	390.1	10.8	91.6
21AR234_22	230.5	6485.190	0.887	17.031	4.0	0.64161	5.6	0.08261	1.5	0.270	511.7	7.4	503.3	22.0	466.4	118.4	511.7	7.4	109.7
21AR234_23	261.2	5185.985	2.340	17.188	6.0	0.17245	7.3	0.02265	2.8	0.390	144.4	4.1	161.5	10.9	421.7	149.7	144.4	4.1	34.2
21AR234_24	177.4	5206.362	1.024	15.605	8.7	0.33106	11.6	0.03933	2.1	0.181	248.7	5.1	290.4	29.3	641.9	245.3	248.7	5.1	38.7
21AR234_25	490.8	8325.678	5.006	16.329	2.1	0.68336	6.6	0.08353	2.6	0.398	517.2	13.0	528.8	27.1	580.4	131.1	517.2	13.0	89.1
21AR234_26	277.6	87017.760	4.106	6.837	1.3	7.92467	2.3	0.39445	1.8	0.807	2143.4	33.7	2222.5	20.6	2296.9	23.2	2296.9	23.2	93.3
21AR234_27	152.7	9484.748	11.900	16.281	5.7	0.59813	6.6	0.07263	2.5	0.375	452.0	10.8	476.0	25.1	594.7	132.7	452.0	10.8	76.0
21AR234_28	493.4	14993.079	0.905	18.734	3.2	0.27464	3.8	0.03817	1.7	0.453	241.5	4.0	246.4	8.2	294.7	76.4	241.5	4.0	81.9
21AR234_29	90.9	3990.896	1.188	13.685	11.2	0.41059	13.1	0.04303	2.7	0.202	271.6	7.0	349.3	38.8	905.6	265.1	271.6	7.0	30.0
21AR234_30	106.6	6719.689	6.099	15.501	7.2	0.58928	8.1	0.06868	2.9	0.361	428.2	12.2	470.4	30.7	683.0	162.2	428.2	12.2	62.7
21AR234_31	120.7	433.070	4.179	10.907	9.6	0.44477	41.5	0.05571	19.2	0.463	349.5	65.4	373.6	129.9	527.0	812.4	349.5	65.4	66.3
21AR234_32	189.4	20026.697	1.501	16.975	3.9	0.65872	4.7	0.08244	1.7	0.370	510.7	8.5	513.8	18.9	528.9	95.5	510.7	8.5	96.6
21AR234_33	132.9	14701.898	2.655	13.474	2.5	1.57528	3.3	0.15661	1.8	0.539	937.9	15.4	960.5	20.4	1013.4	56.0	1013.4	56.0	92.6
21AR234_34	434.6	79715.560	3.392	13.206	1.2	1.80976	2.2	0.17428	1.8	0.811	1035.7	17.2	1049.0	14.5	1077.7	26.0	1077.7	26.0	96.1
21AR234_35	167.7	6356.432	2.262	16.955	6.6	0.32594	9.4	0.04178	2.0	0.215	263.9	5.2	286.5	23.4	476.0	203.0	263.9	5.2	55.4
21AR234_36	437.1	18575.712	0.405	17.804	3.0	0.46506	4.1	0.06102	1.8	0.440	381.8	6.7	387.8	13.2	424.3	82.0	381.8	6.7	90.0
21AR234_37	229.3	6501.335	0.945	16.559	6.5	0.26260	7.1	0.03282	1.8	0.262	208.1	3.8	236.8	14.9	532.1	149.4	208.1	3.8	39.1
21AR234_38	207.4	26280.063	1.635	13.669	1.9	1.60308	2.7	0.16042	1.5	0.568	959.1	13.5	971.4	16.7	1000.2	44.6	1000.2	44.6	95.9
21AR234_39	339.7	48560.888	2.019	13.629	1.5	1.60070	2.3	0.15931	1.6	0.674	952.9	13.8	970.5	14.4	1011.3	34.6	1011.3	34.6	94.2
21AR234_40	252.1	19277.054	1.282	17.677	2.5	0.58196	3.4	0.07568	1.9	0.548	470.3	8.4	465.7	12.6	444.2	62.9	470.3	8.4	105.9
21AR234_41	180.0	3243.818	0.477	16.690	4.5	0.45277	8.3	0.05935	2.2	0.269	371.7	8.1	379.2	26.3	426.3	178.3	371.7	8.1	87.2
21AR234_42	398.9	5779.876	0.736	16.047	8.8	0.13664	11.1	0.01660	2.0	0.179	106.1	2.1	130.0	13.5	593.6	236.9	106.1	2.1	17.9
21AR234_43	164.1	1000.229	0.754	14.328	8.1	0.31022	17.3	0.04083	1.7	0.099	258.0	4.3	274.4	41.6	417.4	385.5	258.0	4.3	61.8
21AR234_44	161.8	6121.122	2.903	16.339	7.1	0.37866	8.9	0.04668	2.3	0.259	294.1	6.7	326.1	24.9	562.0	188.1	294.1	6.7	52.3
21AR234_45	650.5	9922.339	1.035	15.891	1.6	0.83642	3.1	0.09893	2.0	0.647	608.2	11.6	617.2	14.2	651.3	50.4	608.2	11.6	93.4
21AR234_46	365.4	78080.499	0.598	10.898	1.3	3.02173	2.3	0.23992	1.8	0.805	1386.3	23.0	1413.1	17.5	1454.6	25.8	1454.6	25.8	95.3
21AR234_47	516.5	13249.237	1.634	18.615	4.1	0.20302	4.9	0.02806	2.4	0.482	178.4	4.2	187.7	8.5	307.4	98.7	178.4	4.2	58.0
21AR234_48	270.5	5714.872	15.024	15.260	3.5	0.70164	6.0	0.08097	3.6	0.607	501.9	17.4	539.8	24.9	703.9	100.7	501.9	17.4	71.3
21AR234_49	122.7	7271.996	0.507	15.873	6.9	0.55237	8.2	0.06593	4.2	0.513	411.6	16.8	446.5	29.7	632.0	151.7	411.6	16.8	65.1
21AR234_50	108.2	5741.504	1.226	16.849	7.3	0.45352	9.5	0.05794	2.0	0.212	363.1	7.1	379.7	30.2	483.6	206.0	363.1	7.1	75.1
21AR234_51	389.8	8313.588	1.175	17.598	4.1	0.30927	4.9	0.04086	1.8	0.359	258.2	4.5	273.6	11.8	408.9	102.6	258.2	4.5	63.1
21AR234_52	457.0	62707.451	6.694	13.447	1.3	1.73049	2.9	0.16982	2.5	0.861	1011.1	23.1	1019.9	18.4	1039.7	29.5	1039.7	29.5	97.2
21AR234_53	406.3	12455.308	2.827	18.146	6.0	0.20468	7.4	0.02755	1.6	0.215	175.2	2.7	189.1	12.7	367.4	162.2	175.2	2.7	47.7
21AR234_54	167.5	6637.766	0.786	15.865	6.9	0.32689	9.5	0.03888	1.8	0.188	245.9	4.3	287.2	23.8	639.6	200.9	245.9	4.3	38.4
21AR234_55	152.9	23623.056	1.498	13.547	2.1	1.68143	3.1	0.16620	2.0	0.642	991.2	18.3	1001.5	19.7	1025.1	48.1	1025.1	48.1	96.7
21AR234_56	142.7	5786.051	2.897	17.589	6.3	0.32193	7.9	0.04283	2.0	0.255	270.4	5.3	283.4	19.5	393.3	171.1	270.4	5.3	68.7
21AR234_57	171.2	10697.715	2.501	17.509	5.1	0.45352	6.0	0.05899	1.9	0.314	369.5	6.7	379.7	18.9	443.8	125.9	369.5	6.7	83.2
21AR234_58	365.7	8364.976	6.567	13.326	1.5	1.50681	3.3	0.14898	2.9	0.872	895.3	23.8	933.1	20.0	1024.6	32.4	1024.6	32.4	87.4
21AR234_59	284.4	15859.813	3.926	17.762	2.9	0.54891	4.3	0.07194	1.8	0.414	447.8	7.8	444.3	15.6	426.9	88.0	447.8	7.8	104.9

21AR234_60	469.2	3678.454	1.261	16.762	3.7	0.55900	14.1	0.07278	1.7	0.123	452.9	7.6	450.9	51.2	441.7	310.7	452.9	7.6	102.5
21AR234_61	491.1	16542.571	1.056	18.583	3.3	0.28633	4.8	0.03923	1.8	0.382	248.1	4.5	255.7	10.9	327.1	101.1	248.1	4.5	75.8
21AR234_62	84.4	791.367	1.573	12.125	14.9	0.37003	40.4	0.04177	3.8	0.093	263.8	9.7	319.7	110.9	750.9	857.9	263.8	9.7	35.1
21AR234_63	513.5	14430.627	1.457	19.066	2.9	0.25949	3.6	0.03662	1.8	0.505	231.9	4.2	234.3	7.6	259.3	72.0	231.9	4.2	89.4
21AR234_64	99.5	5841.846	0.949	15.498	8.2	0.51971	9.5	0.06099	2.2	0.228	381.6	8.0	425.0	33.0	668.3	198.4	381.6	8.0	57.1
21AR234_65	100.2	30206.026	0.916	9.149	1.8	4.41390	2.7	0.29526	2.0	0.737	1667.8	29.1	1715.0	22.2	1773.8	33.1	1773.8	33.1	94.0
21AR234_66	458.2	2622.132	17.038	15.997	2.9	0.49969	6.6	0.06387	1.8	0.271	399.1	6.9	411.5	22.3	482.4	140.0	399.1	6.9	82.7
21AR234_67	89.0	6391.787	1.913	15.669	6.8	0.61548	8.5	0.07280	2.0	0.241	453.0	8.9	487.0	32.9	651.3	177.0	453.0	8.9	69.6
21AR234_68	198.2	15685.474	1.685	16.466	4.1	0.72904	5.1	0.08866	2.0	0.386	547.6	10.3	556.0	21.8	591.5	101.8	547.6	10.3	92.6
21AR234_69	257.8	22512.942	2.996	16.440	2.5	0.87008	3.9	0.10493	2.1	0.542	643.3	13.0	635.6	18.5	609.4	71.1	643.3	13.0	105.6
21AR234_70	94.3	3773.040	2.103	13.611	13.0	0.39391	15.2	0.04117	2.6	0.173	260.1	6.7	337.2	43.6	911.5	308.2	260.1	6.7	28.5
21AR234_71	208.3	2186.789	2.087	10.615	22.1	0.12306	23.2	0.01021	3.5	0.150	65.5	2.3	117.8	25.8	1371.4	443.2	65.5	2.3	4.8
21AR234_72	154.0	32046.726	0.988	9.385	1.4	4.01092	2.2	0.27518	1.7	0.755	1567.0	23.3	1636.4	18.0	1727.5	26.6	1727.5	26.6	90.7
21AR234_73	214.6	14762.155	3.172	13.407	1.6	1.58012	3.9	0.15644	3.3	0.838	937.0	28.6	962.4	24.3	1021.9	43.1	1021.9	43.1	91.7
21AR234_74	311.4	26276.770	3.488	17.523	3.1	0.58003	4.2	0.07481	2.4	0.562	465.1	10.6	464.5	15.7	462.5	77.5	465.1	10.6	100.6
21AR234_75	191.1	441.870	1.712	5.621	37.5	0.05679	42.3	0.00278	11.3	0.267	17.9	2.0	56.1	23.1	2323.7	707.0	17.9	2.0	0.8
21AR234_76	206.3	16860.635	10.635	16.824	3.9	0.65344	4.8	0.08131	2.0	0.423	503.9	9.8	510.6	19.2	541.5	95.0	503.9	9.8	93.1
21AR234_77	211.4	1542.126	2.875	14.501	10.7	0.23296	25.8	0.02853	3.3	0.128	181.3	5.9	212.6	49.6	576.1	559.3	181.3	5.9	31.5
21AR234_78	727.6	537.930	1.068	5.959	35.1	0.01850	45.9	0.00094	10.3	0.224	6.0	0.6	18.6	8.5	2264.1	781.4	6.0	0.6	0.3
21AR234_79	111.4	26040.513	1.825	8.956	1.7	3.98690	2.5	0.26136	1.9	0.736	1496.8	24.9	1631.5	20.6	1810.7	31.2	1810.7	31.2	82.7
21AR234_80	430.7	19372.938	1.027	17.874	4.1	0.33026	4.6	0.04359	1.6	0.339	275.0	4.2	289.8	11.7	411.3	97.6	275.0	4.2	66.9
21AR234_81	553.9	7251.495	3.711	16.462	2.3	0.67314	3.9	0.08347	1.6	0.416	516.8	8.1	522.6	16.0	549.2	77.6	516.8	8.1	94.1
21AR234_82	154.1	40754.851	0.851	8.634	1.3	5.01463	2.2	0.31633	1.7	0.794	1771.8	27.1	1821.8	18.6	1880.2	24.1	1880.2	24.1	94.2
21AR234_83	113.2	19233.638	1.856	13.215	2.4	1.74374	3.2	0.16962	1.7	0.522	1010.0	15.5	1024.8	20.5	1057.6	54.7	1057.6	54.7	95.5
21AR234_84	306.1	15090.416	2.028	18.180	4.4	0.34407	5.3	0.04641	1.5	0.291	292.5	4.4	300.3	13.7	362.2	113.6	292.5	4.4	80.7
21AR234_85	190.7	19236.825	4.058	15.380	2.8	1.05931	3.6	0.11960	1.9	0.518	728.3	12.8	733.5	18.7	750.3	64.7	728.3	12.8	97.1
21AR234_86	120.4	14368.571	2.433	13.446	3.2	1.71203	4.1	0.17006	1.7	0.426	1012.5	16.3	1013.0	26.1	1015.2	74.8	1015.2	74.8	99.7
21AR234_87	192.1	3018.877	1.132	12.757	9.6	0.14866	11.5	0.01471	3.6	0.316	94.1	3.4	140.7	15.1	1022.9	220.5	94.1	3.4	9.2
21AR234_88	476.7	55032.920	2.387	13.407	1.4	1.72884	2.2	0.16952	1.6	0.740	1009.5	15.1	1019.3	14.0	1041.4	29.7	1041.4	29.7	96.9
21AR234_89	248.1	7792.667	0.961	18.181	4.7	0.32423	6.5	0.04446	1.4	0.208	280.4	3.7	285.1	16.2	324.9	144.8	280.4	3.7	86.3
21AR234_90	221.7	21318.987	2.910	16.521	3.3	0.77830	3.8	0.09475	1.7	0.446	583.5	9.6	584.5	17.1	589.2	74.7	583.5	9.6	99.0
21AR234_91	124.0	54545.667	1.331	5.569	1.2	11.31144	1.8	0.45955	1.3	0.745	2437.6	26.8	2549.1	16.5	2639.9	19.6	2639.9	19.6	92.3
21AR234_92	188.5	433.194	1.514	4.342	31.2	0.06926	38.0	0.00249	14.1	0.371	16.0	2.3	68.0	25.0	2843.1	580.6	16.0	2.3	0.6
21AR234_93	294.0	14730.194	2.574	17.594	3.3	0.45107	4.2	0.05884	1.7	0.414	368.6	6.2	378.0	13.1	437.4	84.3	368.6	6.2	84.3
21AR234_94	88.9	5755.722	2.241	14.925	7.3	0.56007	9.3	0.06322	2.9	0.312	395.2	11.1	451.6	33.9	750.7	186.6	395.2	11.1	52.6
21AR234_95	468.9	78635.452	1.987	13.552	1.4	1.68641	2.2	0.16610	1.6	0.742	990.6	14.8	1003.4	13.9	1032.4	29.5	1032.4	29.5	95.9
21AR234_96	362.7	12938.235	5.158	13.413	1.6	1.48130	5.7	0.14681	5.1	0.894	883.1	42.1	922.8	34.6	1019.7	51.8	1019.7	51.8	86.6
21AR234_97	365.8	18422.556	38.452	17.864	3.1	0.46968	4.3	0.06196	1.8	0.421	387.5	6.8	391.0	14.0	412.4	87.3	387.5	6.8	94.0
21AR234_98	93.7	2262.345	1.222	12.435	12.5	0.26054	13.6	0.02559	3.1	0.225	162.9	4.9	235.1	28.6	1037.9	268.2	162.9	4.9	15.7
21AR234_99	174.9	21498.026	2.820	13.856	2.7	1.39753	5.9	0.14228	5.2	0.881	857.5	42.0	887.9	35.2	965.2	57.6	965.2	57.6	88.8
21AR234_100	287.5	25443.877	9.109	16.870	2.7	0.71717	4.4	0.08891	3.4	0.779	549.1	18.0	549.0	18.6	549.5	60.0	549.1	18.0	99.9
21AR234_101	180.3	3596.119	1.316	14.721	11.4	0.17901	13.5	0.02036	4.0	0.293	129.9	5.1	167.2	20.8	735.1	273.2	129.9	5.1	17.7
21AR234_102	443.0	41467.838	239.257	16.481	1.9	0.81657	2.7	0.09838	2.0	0.725	604.9	11.5	606.1	12.5	611.7	40.9	604.9	11.5	98.9
21AR234_103	288.8	51874.116	1.175	13.582	1.5	1.71394	2.7	0.16982	2.2	0.813	1011.1	20.2	1013.8	17.1	1020.3	31.4	1020.3	31.4	99.1
21AR234_104	246.4	9490.966	2.250	17.672	4.7	0.30734	5.8	0.04058	1.8	0.309	256.4	4.5	272.1	13.8	410.4	122.6	256.4	4.5	62.5
21AR234_105	124.6	17371.330	1.915	14.525	3.2	1.29056	3.9	0.13788	1.5	0.391	832.6	11.9	841.5	22.2	866.0	74.2	832.6	11.9	96.1
21AR234_106	281.6	8077.751	1.743	15.729	7.8	0.22007	8.8	0.02582	2.2	0.250	164.4	3.6	202.0	16.0	668.5	181.6	164.4	3.6	24.6
21AR234_107	106.5	8240.498	1.767	16.166	6.6	0.61702	7.5	0.07471	1.7	0.231	464.5	7.8	488.0	29.1	600.8	158.3	464.5	7.8	77.3
21AR234_108	526.2	6456.376	1.039	16.720	8.9	0.10021	10.3	0.01267	1.9	0.183	81.2	1.5	97.0	9.5	506.3	222.2	81.2	1.5	16.0
21AR234_109	327.3	9403.251	2.956	14.236	2.4	0.90454	6.5	0.09565	5.9	0.903	588.9	32.9	654.1	31.3	887.2	57.6	588.9	32.9	66.4
21AR234_110	108.4	8891.903	1.738	16.516	4.7	0.65998	5.6	0.08149	1.8	0.317	505.0	8.7	514.6	22.7	558.3	116.3	505.0	8.7	90.5
21AR234_111	226.8	19649.766	1.402	17.476	3.3	0.59204	3.9	0.07626	1.8	0.455	473.8	8.1	472.2	14.7	465.4	76.8	473.8	8.1	101.8
21AR234_112	171.0	7304.474	1.152	17.206	6.2	0.32434	7.2	0.04204	1.7	0.233	265.5	4.4	285.2	18.0	451.4	156.3	265.5	4.4	58.8
21AR234_113	77.3	11322.547	1.279	12.803	3.4	2.05991	4.5	0.19484	1.5	0.342	1147.5	16.1	1135.6	30.6	1113.7	84.0	1113.7	84.0	103.0
21AR234_114	99.8	16286.958	0.905	12.473	2.8	2.17985	3.5	0.20006	1.8	0.523	1175.6	19.8	1174.6	24.5	1173.6	59.3	1173.6	59.3	100.2
21AR234_115	241.8	19288.611	3.473	16.629	3.3	0.75344	5.4	0.09235	3.7	0.690	569.4	20.1	570.2	23.4	574.4	84.3	569.4	20.1	99.1
21AR234_116	314.5	12988.070	1.261	17.585	5.4	0.32007	6.9	0.04163	1.7	0.247	262.9	4.4	282.0	17.1	444.0	149.7	262.9	4.4	59.2
21AR234_117	514.3	44252.740	5.317	13.412	1.5	1.56949	3.5	0.15387	3.1	0.897	922.6	27.0	958.2	21.7	1041.7	31.2	1041.7	31.2	88.6
21AR234_118	875.0	74510.293	84.096	17.487	1.5	0.57789	3.9	0.07380	3.6	0.922	459.0	15.9	463.1	14.5	484.3	33.4	459.0	15.9	94.8
21AR234_119	109.4	8373.327	2.397	15.496	5.9	0.71534	7.3	0.08295	1.9	0.259	513.7	9.4	547.9	31.0	693.8	150.7	513.7	9.4	74.0
21AR234_120	318.5	7736.167	1.700	17.767	3.6	0.43148	5.9	0.05786	1.4	0.234	362.6	4.8	364.2	17.9	375.4	128.1	362.6	4.8	96.6
21AR234_121	172.9	8099.800	0.844	16.098	6.3	0.32985	8.5	0.03988	2.1	0.251	252.1	5.3	289.4	21.5	603.7	179.1	252.1	5.3	41.8
21AR234_122	182.4	5713.177	1.183	16.082	7.5	0.32531	8.8	0.03979	1.9	0.217	251.5	4.7	286.0	21.8	578.9	185.9	251.5	4.7	43.5
21AR234_123	484.7	42243.478	4.958	16.230	1.8	0.87559	2.6	0.10425	1.6	0.636	639.2	10.0	638.6	12.2	637.3	42.8	639.2	10.0	100.3
21AR234_124	101.4	1796.127	1.376	9.641	23.5	0.23856	24.5	0.01813	5.8	0.236	115.8	6.6	217.2	47.9	1537.7	449.7	115.8	6.6	7.5
21AR234_125	157.6	11280.401	1.634	16.152	4.8	0.62407	5.4	0.07463	2.0	0.368	464.0	8.9	492.4	21.1	627.8	108.5	464.0	8.9	73.9
21AR234_126	161.3	25066.103	2.055	13.475	2.6	1.68918	3.4	0.16733	2.0	0.597	997.4	18.7	1004.5	21.7	1020.7	55.3	1020.7	55.3	97.7
21AR234_127	332.4	21568.767	1.516	17.842	3.7	0.46462	4.3	0.06121	1.9	0.431	383.0	7.0	387.5	14.0	415.2	87.7	383.0	7.0	92.2
21AR234_128	293.7	23717.897	69.576	17.297	3.0	0.62587	3.8	0.07982	2.0	0.538	495.0	9.7	493.5	14.8	487.4	70.3	495.0	9.7	101.6
21AR234_129	256.1	41719.920	3.746	13.197	1.6	1.80545	2.5	0.17457	1.9	0.729	1037.2	17.8	1047.4	16.6	1069.6	35.0	1069.6	35.0	97.0
21AR234_130	197.2	4314.022	1.739	15.331	8.2	0.24242	13.2	0.02857	2.2	0.167	181.6	3.9	220.4	26.1	659.0	278.5	181.6	3.9	27.6
21AR234_131	346.0	19854.219	1.537	17.898	3.3	0.46673	4.3	0.06173	1.9	0.432	386.1	7.0	388.9	13.9	406.7	86.7	386.1	7.0	94.9
21AR234_132	233.0	16501.829	2.155	16.880	3.6	0.59706	4.2	0.07460	1.7	0.414	463.8	7.7	475.4	15.9	532.6	83.4	463.8	7.7	87.1
21AR234_133	133.5	5352.473	0.726	15.115	7.4	0.34923	9.2	0.04013	2.0	0.218	253.6	5.0	304.1	24.3	713.2	191.7	253.6	5.0	35.6
21AR234_134	383.6	1648.082	1.398	15.798	4.0	0.34125	21.2	0.04572	5.2	0.246	288.2	14.7	298.1	54.8	377.8	463.5	288.2	14.7	76.3
21AR234_135	179.4	23461.796	3.949	13.793	2.0	1.51211	4.6	0.15262	4.1	0.887	915.7	34.9	935.3	28.2	982.8	43.2	982.8	43.2	93.2
21AR234_136	772.8	3869.049	2.099	16.721	2.0	0.49618	2.6	0.06459	1.4	0.519	403.5	5.4	409.1	8.9	442.0	50.4	403.5	5.4	91.3
21AR234_137	373.9	13368.035	3.557	18.432	3.3	0.27790	5.2	0.03814	1.4	0.267	241.3	3.3	249.0	11.5	323.3	113.7	241.3	3.3	74.6
21AR234_138	366.1	31054.720	29.184	17.709	2.6	0.56293	3.3	0.07332	1.4	0.426	456.1	6.2	453.4	12.0	440.7	66.1	456.1	6.2	103.5
21AR234_139	170.0	24458.113	132.836	12.727	1.8	1.96138	4.5	0.18334	3.9	0.860	1085.2	38.8	1102.4	30.3	1137.3	45.8	1137.3	45.8	95.4
21AR234_140	272.0	20154.540	8.834	17.718	3.3	0.47720	4.0	0.06244	1.3	0.326	390.5	4.9	396.1	13.1	430.4	83.9	390.5	4.9	90.7
21AR234_141	305.9	92383.143	4.046	7.924	1.0	5.63217	1.5	0.32575	1.1	0.738	1817.8	17.3	1921.0	12.8	2035.2	17.7	2035.2	17.7	89.3
21AR234_142	548.4	4848.399	0.948	18.611	3.1	0.26601	8.3	0.03825	2.6	0.315	242.0	6.2	239.5	17.7	216.4	182.2	242.0	6.2	111.8
21AR234_143	152.1	8313.192	1.526	17.242	5.2	0.47417	5.9	0.06142	2.1	0.347	384.3	7.7	394.1	19.4	453.0	123.9	384.3	7.7	84.8
21AR234_144	108.2	7912.571	1.251	14.337	4.5	1.30427	6.0	0.13985	2.0	0.332	843.8	15.6	847.6	34.2	858.4	116.7	843.8	15.6	98.3
21AR234_145	212.5	10924.478	1.138	17.368	3.9	0.47280	5.3	0.06111	1.7	0.330	382.4	6.4	393.1	17.1	457.8	110.1	382.4	6.4	83.5
21AR234_146	63.9	357.190	1.168	6.825	36.4	0.18770	54.2	0.01262	10.9	0.202	80.8	8.8	174.7	87.0	1764.8	987.6	80.8	8.8	4.6
21AR234_147	231.4	20424.032	1.227	16.479	2.9	0.77010	3.9	0.09354	1.3	0.340	576.4	7.3	579.8	17.3	594.0	79.9	576.4	7.3	97.0
21AR234_148	249.4	6241.055	1.254	15.978	8.7	0.19806	9.8	0.02394	1.6	0.159	152.5	2.3	183.5	16.4	604.3	208.6	152.5	2.3	25.2
21AR234_149	22.4	3023.918	1.708	12.311	11.6	1.54370	13.0	0.14688	2.2	0.168	883.5	18.0	948.0	80.4	1101.9	257.5	1101.9	257.5	80.2
21AR234_150	243.4	41423.166	3.411	13.412	1.6	1.79293	2.4	0.17599	1.8	0.729	1045.0	17.1	1042.9	15.8	1039.3	33.6	1039.3	33.6	100.6
21AR234_151	288.0	14560.725	2.984	17.679	4.2	0.35225	5.5	0.04618	1.6	0.297	291.1	4.6	306.4	14.5	426.0	116.8	291.1	4.6	68.3
21AR234_152	319.1	52784.323	5.942	13.466	1.5	1.75424	2.7	0.17271	2.2	0.809	1027.0	20.6	1028.7	17.3	1033.2	31.8	1033.2	31.8	99.4
21AR234_153	201.0	8327.384	0.889	17.242	5.8	0.31423	6.6	0.04070	1.8	0.282	257.1	4.7	277.5	15.9	453.3	139.7	257.1	4.7	56.7
21AR234_154	219.7	27696.377	2.556	13.957	2.3	1.57650	3.3	0.16142	2.0	0.609	964.7	17.8	961.0	20.2	953.5	52.9	953.5	52.9	101.2
21AR234_155	546.5	7403.659	2.700	16.565	1.9	0.68811	3.2	0.08578	1.6	0.506	530.5	8.3	531.7	13.3	537.6	60.7	530.5	8.3	98.7
21AR234_156	54.7	1880.261	1.477	11.322	13.6	0.45463	14.5	0.04096	3.4	0.235	258.8	8.6	380.5	45.9	1210.2	277.3	258.8	8.6	21.4
21AR234_157	757.9	8933.733	41.767	12.274	1.3	2.12656	4.2	0.19380	2.5	0.602	1141.9	26.4	1157.5	29.0	1187.6	66.1	1187.6	66.1	96.2
21AR234_158	266.4	7146.766	1.826	18.332	6.3	0.20872	7.2	0.02895	2.1	0.294	184.0	3.8	192.5	12.6	299.3	156.6	184.0	3.8	61.5
21AR234_159	285.8	12040.347	6.013	17.684	3.1	0.60346	4.4	0.07938	2.5	0.574	492.4	11.9	479.4	16.7	418.6	80.1	492.4	11.9	117.6
21AR234_160	58.9	2121.311	0.780	13.386	16.1	0.30472	25.5	0.03264	4.2	0.166	207.1	8.6	270.1	60.4	860.4	522.9	207.1	8.6	24.1
21AR234_161	342.7	7585.933	1.159	18.901	3.7	0.27765	7.4	0.03964	2.3	0.314	250.6	5.7	248.8	16.3	232.9	162.1	250.6	5.7	107.6
21AR234_162	483.6	14891.467	9.060	17.487	2.9	0.56495	3.8	0.07315	2.1	0.540	455.1	9.1	454.7	14.1	454.1	71.8	455.1	9.1	100.2
21AR234_163	332.6	10072.858	1.139	13.867	2.0	1.43603	4.1	0.14767	3.0	0.741	887.9	25.3	904.1	24.6	944.7	56.6	944.7	56.6	94.0
21AR234_164	427.4	1697.969	1.262	16.366	8.5	0.12428	23.8	0.01724	2.3	0.095	110.2	2.5	118.9	26.7	299.0	542.0	110.2	2.5	36.8
21AR234_165	582.7	5289.810	1.568	17.240	6.0	0.09738	7.4	0.01283	2.3	0.316	82.2	1.9	94.4	6.7	415.5	157.4	82.2	1.9	19.8
21AR234_166	114.3	8903.062	4.657	16.483	5.9	0.66395	8.7	0.08181	4.9	0.564	506.9	23.8	517.0	35.1	563.0	156.0	506.9	23.8	90.0
21AR234_167	149.4	6143.773	2.389	16.873	5.2	0.43497	5.8	0.05562	2.0	0.348	348.9	6.9	366.7	18.0	481.8	121.1	348.9	6.9	72.4
21AR234_168	171.6	24585.815	1.647	13.483	2.3	1.71036	2.8	0.16908	1.3	0.483	1007.1	12.6	1012.4	17.9	1024.9	49.4	1024.9	49.4	98.3
21AR234_169	346.2	31732.771	4.981	16.484	2.0	0.77598	2.5	0.09376	1.4	0.563	577.8	7.7	583.2	11.0	605.3	44.3	577.8	7.7	95.4
21AR234_170	602.7	7671.950	2.661	18.544	3.3	0.31905	6.6	0.04464	1.5	0.226	281.5	4.1	281.2	16.2	279.1	147.3	281.5	4.1	100.9
21AR234_171	538.9	7675.723	4.319	13.376	1.7	1.48955	4.3	0.14852	3.9	0.909	892.7	32.7	926.1	26.2	1007.6	36.5	1007.6	36.5	88.6
21AR234_172	234.1	8640.443	1.039	16.768	6.2	0.31782	6.7	0.03991	1.5	0.231	252.3	3.8	280.2	16.3	521.3	142.1	252.3	3.8	48.4
21AR234_173	1035.7	100390.511	29.801	16.686	1.4	0.76142	2.1	0.09269	1.6	0.763	571.4	8.9	574.8	9.3	589.2	29.8	571.4	8.9	97.0
21AR234_174	97.3	6100.951	3.850	15.245	7.7	0.56719	8.5	0.06529	2.7	0.315	407.7	10.6	456.2	31.4	709.4	172.2	407.7	10.6	57.5
21AR234_175	276.9	25233.134	2.631	17.036	2.7	0.65538	3.4	0.08211	1.4	0.422	508.7	6.9	511.8	13.5	526.6	66.6	508.7	6.9	96.6

21AR234_176	24.7	3768.077	1.791	13.062	9.8	1.57691	12.2	0.15783	2.1	0.174	944.7	18.6	961.2	75.7	999.8	243.7	999.8	243.7	94.5
21AR234_177	64.8	14922.565	0.903	11.194	3.3	2.90135	3.9	0.23909	1.7	0.440	1381.9	21.6	1382.3	29.8	1383.6	68.0	1383.6	68.0	99.9
21AR234_178	127.9	16497.002	1.876	12.888	3.1	1.75650	6.6	0.16675	5.4	0.820	994.2	49.6	1029.6	42.5	1106.4	75.1	1106.4	75.1	89.9
21AR234_179	137.0	8531.461	5.781	17.485	5.3	0.55927	6.1	0.07347	1.9	0.306	457.0	8.3	451.1	22.3	421.7	130.0	457.0	8.3	108.4
21AR234_180	154.3	8382.446	2.872	17.365	4.8	0.49608	7.1	0.06446	2.1	0.294	402.7	8.1	409.0	23.9	446.1	150.8	402.7	8.1	90.3
21AR234_181	233.1	10680.470	2.324	17.924	4.1	0.31874	4.8	0.04270	2.0	0.406	269.5	5.2	280.9	11.9	377.9	99.5	269.5	5.2	71.3
21AR234_182	518.2	33189.370	6.455	17.413	1.8	0.66064	2.4	0.08451	1.5	0.650	523.0	7.7	515.0	9.6	480.6	39.8	523.0	7.7	108.8
21AR234_183	281.8	11906.617	1.407	18.237	5.7	0.30059	6.5	0.04088	1.8	0.274	258.3	4.5	266.9	15.2	343.8	141.2	258.3	4.5	75.1
21AR234_184	136.7	7812.290	6.045	16.752	4.7	0.45754	7.6	0.05768	1.7	0.223	361.5	5.9	382.5	24.2	513.0	162.5	361.5	5.9	70.5
21AR234_185	79.1	4725.103	1.724	14.011	10.6	0.75569	11.5	0.08063	1.7	0.149	499.9	8.3	571.5	50.3	868.6	236.0	499.9	8.3	57.6
21AR234_186	217.6	13385.309	1.987	16.969	3.3	0.65098	3.9	0.08205	1.5	0.388	508.3	7.5	509.1	15.8	513.4	79.8	508.3	7.5	99.0
21AR234_187	184.3	5140.493	1.337	16.419	6.4	0.32628	7.2	0.04096	2.0	0.280	258.8	5.1	286.7	18.0	522.1	151.7	258.8	5.1	49.6
21AR234_188	981.1	950.631	0.816	7.272	25.0	0.01615	33.7	0.00095	8.8	0.261	6.1	0.5	16.3	5.4	2000.3	582.2	6.1	0.5	0.3
21AR234_189	128.5	3307.217	1.448	13.539	10.7	0.27607	15.4	0.02883	2.8	0.179	183.2	5.0	247.5	33.9	912.7	313.0	183.2	5.0	20.1
21AR234_190	243.8	17019.217	84.685	18.332	3.4	0.47386	4.5	0.06437	2.7	0.595	402.1	10.5	393.8	14.7	346.4	81.9	402.1	10.5	116.1
21AR234_191	149.1	55158.047	3.054	9.133	1.2	4.74778	1.9	0.31560	1.5	0.753	1768.2	22.5	1775.7	16.2	1785.3	23.2	1785.3	23.2	99.0
21AR234_192	183.3	7517.525	0.731	16.466	8.5	0.31511	9.7	0.03893	1.8	0.183	246.2	4.3	278.1	23.6	557.1	208.4	246.2	4.3	44.2
21AR234_193	88.7	4674.193	1.136	15.332	9.0	0.48473	10.5	0.05680	2.2	0.213	356.2	7.7	401.3	34.8	671.2	219.7	356.2	7.7	53.1
21AR234_194	195.2	50735.208	0.838	9.144	1.2	4.50035	1.8	0.30037	1.3	0.731	1693.1	19.3	1731.0	14.8	1778.0	22.1	1778.0	22.1	95.2
21AR234_195	659.2	20119.888	2.230	19.037	2.5	0.29884	3.4	0.04200	1.7	0.510	265.2	4.5	265.5	7.9	268.7	66.4	265.2	4.5	98.7
21AR234_196	390.9	8833.276	1.381	18.153	3.2	0.34980	6.2	0.04767	1.7	0.277	300.2	5.0	304.6	16.2	339.3	133.9	300.2	5.0	88.5
21AR234_197	34.0	1021.037	1.219	8.004	25.7	0.60778	32.5	0.03970	4.8	0.147	251.0	11.8	482.2	125.1	1817.2	588.3	251.0	11.8	13.8
21AR234_198	497.0	9459.117	5.581	18.763	2.4	0.30355	5.0	0.04278	1.8	0.355	270.1	4.7	269.2	11.9	262.4	108.2	270.1	4.7	102.9
21AR234_199	204.2	20229.417	5.019	14.985	2.6	1.09275	4.2	0.12071	3.1	0.737	734.7	21.4	749.8	22.2	796.3	59.2	734.7	21.4	92.3
21AR234_200	370.1	3474.369	1.897	13.731	13.0	0.09813	17.0	0.01040	2.2	0.127	66.7	1.4	95.0	15.4	881.6	348.6	66.7	1.4	7.6
21AR234_201	895.8	11578.758	5.015	16.941	1.6	0.59608	2.9	0.07525	2.4	0.833	467.7	11.0	474.7	11.1	509.8	35.6	467.7	11.0	91.7
21AR234_202	494.9	4255.883	1.423	17.068	2.8	0.46864	9.7	0.06196	2.4	0.246	387.5	9.0	390.3	31.5	407.3	211.0	387.5	9.0	95.2
21AR234_203	227.6	8758.862	1.658	17.370	4.0	0.35124	4.8	0.04574	1.6	0.335	288.3	4.5	305.7	12.7	440.9	100.9	288.3	4.5	65.4
21AR234_204	84.0	3392.949	1.042	14.755	10.6	0.33948	12.7	0.03898	2.1	0.164	246.5	5.0	296.8	32.6	714.6	266.0	246.5	5.0	34.5
21AR234_205	59.5	12621.061	1.126	11.327	4.5	2.65224	5.1	0.22189	1.8	0.349	1291.8	21.0	1315.3	37.9	1354.5	92.9	1354.5	92.9	95.4
21AR234_206	250.6	13071.450	2.473	17.051	4.7	0.46728	5.3	0.05924	2.3	0.424	371.0	8.2	389.3	17.3	500.7	106.4	371.0	8.2	74.1
21AR234_207	124.3	28026.440	2.819	13.247	2.1	1.82308	3.5	0.17681	2.7	0.764	1049.5	26.0	1053.8	23.1	1063.6	45.6	1063.6	45.6	98.7
21AR234_208	118.5	1956.419	1.746	14.634	9.1	0.42683	21.6	0.05102	2.4	0.112	320.8	7.6	360.9	65.7	628.8	464.3	320.8	7.6	51.0
21AR234_209	408.5	23576.894	1.274	17.595	2.7	0.56623	3.4	0.07326	1.6	0.459	455.7	6.9	455.6	12.5	455.7	67.3	455.7	6.9	100.0
21AR234_210	72.7	13825.380	3.014	13.610	3.9	1.69018	4.7	0.16968	1.7	0.363	1010.3	16.0	1004.8	30.0	993.7	89.1	993.7	89.1	101.7
21AR234_211	623.8	44829.152	43.765	17.801	1.8	0.56183	3.2	0.07316	2.4	0.770	455.2	10.8	452.7	11.6	441.3	45.2	455.2	10.8	103.2
21AR234_212	138.0	23762.951	1.774	13.360	2.5	1.64493	4.3	0.16114	3.1	0.724	963.1	27.6	987.6	26.9	1043.3	59.3	1043.3	59.3	92.3
21AR234_213	202.1	11481.111	3.390	15.790	4.5	0.73598	6.4	0.08622	4.4	0.685	533.1	22.6	560.1	27.8	672.0	100.5	533.1	22.6	79.3
21AR234_214	245.4	45837.239	2.096	12.240	1.4	2.25900	2.1	0.20183	1.6	0.744	1185.1	17.1	1199.6	14.9	1226.6	27.9	1226.6	27.9	96.6
21AR234_215	107.7	4222.894	0.708	15.335	10.2	0.34754	12.5	0.04090	2.6	0.206	258.4	6.5	302.9	32.6	661.9	261.6	258.4	6.5	39.0
21AR234_216	313.2	14568.590	1.826	18.039	4.0	0.33605	5.6	0.04488	1.8	0.321	283.0	5.0	294.2	14.4	384.6	119.7	283.0	5.0	73.6
21AR234_217	248.0	5824.322	1.280	16.841	8.5	0.19807	11.1	0.02536	1.8	0.166	161.5	2.9	183.5	18.6	478.4	241.1	161.5	2.9	33.7
21AR234_218	249.6	26786.954	1.608	14.706	2.0	1.24204	2.9	0.13374	1.4	0.470	809.2	10.3	819.8	16.2	849.7	52.8	809.2	10.3	95.2
21AR234_219	651.5	21743.230	1.085	19.437	2.5	0.27958	3.4	0.04000	2.2	0.628	252.8	5.3	250.3	7.6	227.8	61.6	252.8	5.3	111.0
21AR234_220	282.2	10328.243	2.253	18.055	5.8	0.29558	6.4	0.03978	1.8	0.280	251.5	4.4	262.9	14.8	367.2	137.8	251.5	4.4	68.5
21AR234_221	174.2	26513.271	4.197	12.678	2.1	2.05834	2.9	0.19078	2.0	0.683	1125.6	20.6	1135.1	19.9	1154.0	42.2	1154.0	42.2	97.5
21AR234_222	220.3	5966.083	0.847	15.736	8.1	0.23617	8.7	0.02806	2.2	0.252	178.4	3.8	215.3	16.8	641.9	180.5	178.4	3.8	27.8
21AR234_223	211.6	13608.720	1.445	17.435	4.5	0.47391	5.3	0.06114	1.9	0.353	382.5	6.9	393.9	17.2	462.0	109.5	382.5	6.9	82.8
21AR234_224	101.5	1577.919	1.431	11.172	18.5	0.19986	24.7	0.01803	3.9	0.159	115.2	4.5	185.0	41.8	1208.0	482.6	115.2	4.5	9.5
21AR234_225	298.8	42936.479	6.211	13.433	1.3	1.74579	1.9	0.17103	1.4	0.727	1017.8	13.2	1025.6	12.4	1043.2	26.7	1043.2	26.7	97.6
21AR234_226	92.3	3371.149	0.848	14.196	11.2	0.38409	12.1	0.04214	3.3	0.269	266.1	8.5	330.0	34.1	810.5	244.0	266.1	8.5	32.8
21AR234_227	246.8	2624.026	1.917	15.924	4.7	0.46941	5.3	0.05971	1.8	0.346	373.9	6.6	390.8	17.1	493.2	109.4	373.9	6.6	75.8
21AR234_228	159.1	2878.524	1.541	12.543	12.9	0.21314	15.3	0.02069	2.6	0.172	132.0	3.4	196.2	27.3	1061.3	303.4	132.0	3.4	12.4
21AR234_229	102.9	7793.250	2.352	15.879	5.5	0.69194	7.2	0.08223	1.6	0.227	509.4	8.0	534.0	30.0	641.2	151.3	509.4	8.0	79.4
21AR234_230	434.5	11425.116	2.048	18.623	3.0	0.31828	5.6	0.04409	1.7	0.298	278.1	4.5	280.6	13.6	302.1	120.9	278.1	4.5	92.1
21AR234_231	159.4	15739.972	2.597	15.923	3.5	0.89223	4.5	0.10468	1.6	0.353	641.8	9.8	647.6	21.8	668.8	91.1	641.8	9.8	96.0
21AR234_232	298.3	10156.575	14.471	18.056	5.6	0.31772	7.0	0.04276	1.7	0.237	269.9	4.4	280.1	17.1	367.2	152.9	269.9	4.4	73.5
21AR234_233	175.4	8080.253	1.290	17.560	5.3	0.46921	6.2	0.06178	1.9	0.305	386.4	7.1	390.6	20.1	416.6	131.9	386.4	7.1	92.8
21AR234_234	278.0	22443.956	1.394	17.349	2.8	0.58381	3.4	0.07433	1.6	0.475	462.2	7.1	466.9	12.6	491.2	65.5	462.2	7.1	94.1
21AR234_235	128.7	15507.711	1.174	13.484	2.7	1.63515	4.0	0.16251	1.7	0.422	970.7	15.2	983.8	25.2	1014.1	73.4	1014.1	73.4	95.7
21AR234_236	75.2	9586.718	1.880	14.944	5.5	0.92930	6.0	0.10320	1.8	0.307	633.1	11.1	667.3	29.5	785.3	120.6	633.1	11.1	80.6
21AR234_237	287.9	17606.995	1.853	17.812	3.1	0.54905	3.7	0.07211	1.7	0.464	448.8	7.4	444.4	13.2	422.2	72.3	448.8	7.4	106.3
21AR234_238	36.9	383.268	1.171	4.771	41.7	0.30995	44.5	0.01269	13.5	0.304	81.3	10.9	274.1	106.9	2626.7	713.3	81.3	10.9	3.1
21AR234_239	76.9	14528.732	4.759	12.465	2.8	2.11798	3.6	0.19414	1.7	0.478	1143.7	18.1	1154.7	25.0	1176.1	62.9	1176.1	62.9	97.2
21AR234_240	76.6	13539.807	1.245	12.280	3.1	2.26490	3.9	0.20475	1.7	0.434	1200.8	18.5	1201.4	27.4	1203.5	69.1	1203.5	69.1	99.8
21AR234_241	321.5	42036.688	8.227	15.205	1.6	1.13271	2.2	0.12575	1.4	0.624	763.6	10.0	769.0	12.0	785.9	36.5	763.6	10.0	97.2
21AR234_242	560.8	54649.743	20.210	16.177	1.3	0.88994	2.1	0.10502	1.5	0.719	643.7	9.4	646.3	10.2	656.3	31.8	643.7	9.4	98.1
21AR234_243	400.5	5899.438	1.775	17.528	2.6	0.44723	3.8	0.05952	1.3	0.352	372.7	4.8	375.3	11.8	392.7	78.7	372.7	4.8	94.9
21AR234_244	360.1	22760.652	0.944	18.189	3.4	0.46269	4.1	0.06189	1.5	0.376	387.1	5.8	386.1	13.2	381.1	85.5	387.1	5.8	101.6
21AR234_245	612.1	52832.324	243.728	15.989	1.4	0.91266	2.0	0.10657	1.3	0.668	652.8	8.3	658.5	9.7	679.0	31.7	652.8	8.3	96.1
21AR234_246	122.4	8854.431	1.889	16.348	6.0	0.60040	7.0	0.07314	2.3	0.329	455.0	10.1	477.5	26.5	587.7	142.8	455.0	10.1	77.4
21AR234_247	242.1	14345.428	1.544	17.650	4.1	0.58230	5.1	0.07607	1.9	0.376	472.6	8.8	465.9	19.2	434.1	106.3	472.6	8.8	108.9
21AR234_248	363.1	22686.930	207.084	17.233	3.3	0.57075	4.0	0.07217	1.8	0.437	449.2	7.6	458.5	14.8	506.2	79.3	449.2	7.6	88.8
21AR234_249	187.6	1801.731	1.027	11.534	17.3	0.13567	24.7	0.01249	5.6	0.225	80.0	4.4	129.2	29.9	1167.7	477.9	80.0	4.4	6.9
21AR234_250	289.9	20167.409	94.409	16.450	2.6	0.70940	4.0	0.08555	2.0	0.501	529.2	10.2	544.4	16.9	609.6	75.0	529.2	10.2	86.8
21AR234_251	869.1	7147.913	5.549	13.669	1.4	1.33532	3.4	0.13592	3.1	0.908	821.6	23.6	861.2	19.6	965.5	28.9	821.6	23.6	85.1
21AR234_252	246.4	26215.446	5.851	14.970	2.3	1.16742	3.9	0.12764	3.1	0.776	774.4	22.3	785.4	21.6	817.9	52.0	774.4	22.3	94.7
21AR234_253	470.7	12044.297	0.733	17.874	5.9	0.20703	7.4	0.02752	1.7	0.229	175.0	2.9	191.1	13.0	395.2	162.5	175.0	2.9	44.3
21AR234_254	423.9	14710.522	2.378	18.751	3.4	0.28988	4.9	0.04027	1.7	0.340	254.5	4.2	258.5	11.3	295.7	105.9	254.5	4.2	86.1
21AR234_255	91.1	908.923	1.822	12.267	12.5	0.40812	28.7	0.04517	2.9	0.100	284.8	8.0	347.5	84.5	792.2	601.8	284.8	8.0	36.0
21AR234_256	295.3	21685.772	3.594	17.240	2.8	0.64015	3.2	0.08117	1.2	0.385	503.1	5.9	502.4	12.6	500.2	64.8	503.1	5.9	100.6
21AR234_257	879.5	4602.739	8.023	17.665	2.0	0.39440	8.2	0.05359	1.2	0.147	336.5	4.0	337.6	23.5	345.8	183.1	336.5	4.0	97.3
21AR234_258	95.2	1344.681	2.098	7.451	19.9	0.20671	21.6	0.01205	6.6	0.308	77.2	5.1	190.8	37.5	2020.6	364.5	77.2	5.1	3.8
21AR234_259	222.2	13339.896	2.375	16.417	3.4	0.73635	4.5	0.08921	2.2	0.493	550.9	11.6	560.3	19.2	599.6	84.1	550.9	11.6	91.9
21AR234_260	462.9	15782.362	1.634	18.583	4.0	0.28656	4.5	0.03928	1.6	0.354	248.3	3.9	255.9	10.2	326.2	95.5	248.3	3.9	76.1
21AR234_261	1245.6	36670.814	20.992	13.375	1.4	1.70935	2.1	0.16658	1.5	0.676	993.3	13.4	1012.0	13.8	1053.8	31.9	1053.8	31.9	94.3
21AR234_262	49.3	621.298	1.397	8.756	21.0	0.32211	27.0	0.02547	5.3	0.197	162.1	8.5	283.5	66.8	1462.8	504.7	162.1	8.5	11.1
21AR234_263	352.7	10717.429	1.957	18.237	4.1	0.30985	5.0	0.04217	1.5	0.306	266.3	4.0	274.1	12.0	342.0	107.8	266.3	4.0	77.9
21AR234_264	155.2	28793.105	1.801	10.775	1.5	3.12820	2.2	0.24576	1.4	0.633	1416.6	17.4	1439.7	16.6	1474.8	31.7	1474.8	31.7	96.1
21AR234_265	291.0	7347.773	1.937	15.164	9.0	0.24571	10.1	0.02785	1.6	0.159	177.1	2.8	223.1	20.1	741.8	210.1	177.1	2.8	23.9
21AR234_266	684.7	20818.128	1.105	19.468	2.2	0.27217	3.1	0.03897	1.3	0.421	246.4	3.2	244.4	6.8	226.3	65.2	246.4	3.2	108.9
21AR234_267	293.8	46246.284	5.914	13.759	1.7	1.57177	2.6	0.15728	2.0	0.756	941.7	17.4	959.1	16.3	1000.2	35.0	1000.2	35.0	94.1
21AR234_268	109.3	5304.451	2.517	16.241	5.5	0.75012	7.3	0.09233	2.7	0.368	569.3	14.6	568.3	31.8	565.2	147.8	569.3	14.6	100.7
21AR234_269	204.3	6960.438	0.885	17.126	6.4	0.27630	7.1	0.03554	2.1	0.296	225.2	4.7	247.7	15.7	468.3	151.0	225.2	4.7	48.1
21AR234_270	428.7	22329.832	2.019	17.747	2.6	0.55147	3.5	0.07171	1.3	0.367	446.5	5.5	446.0	12.5	444.4	71.8	446.5	5.5	100.5
21AR234_271	380.7	11181.239	1.073	18.544	4.6	0.28668	5.8	0.03946	1.7	0.299	249.5	4.3	255.9	13.2	316.6	126.8	249.5	4.3	78.8
21AR234_272	119.3	43976.601	1.486	5.778	2.0	9.40937	7.3	0.39425	7.0	0.960	2142.5	127.1	2378.7	66.7	2588.5	33.9	2588.5	33.9	82.8
21AR234_273	522.9	4989.158	1.284	14.661	1.6	1.06459	6.0	0.11860	1.8	0.299	722.5	12.2	736.1	31.3	778.6	120.1	722.5	12.2	92.8
21AR234_274	297.8	10211.111	1.628	12.262	1.8	2.15061	3.8	0.19428	1.8	0.478	1144.5	19.2	1165.2	26.5	1204.9	66.3	1204.9	66.3	95.0
21AR234_275	250.9	12802.345	1.243	18.666	4.3	0.29342	5.0	0.04053	1.4	0.272	256.1	3.4	261.2	11.5	308.6	109.2	256.1	3.4	83.0
21AR234_276	148.3	28297.911	8.449	13.977	2.2	1.50044	3.2	0.15300	2.1	0.681	917.8	18.4	930.6	19.2	961.9	47.3	961.9	47.3	95.4
21AR234_277	223.4	7808.103	2.012	16.466	7.7	0.34208	9.1	0.04215	1.4	0.151	266.1	3.6	298.7	23.5	563.1	195.5	266.1	3.6	47.3
21AR234_278	68.0	828.879	1.062	7.148	33.0	0.25463	35.3	0.01492	7.8	0.221	95.5	7.4	230.3	72.7	2012.4	614.9	95.5	7.4	4.7
21AR234_279	131.5	6650.431	2.109	15.703	8.4	0.37978	9.4	0.04479	2.7	0.291	282.4	7.6	326.9	26.3	657.7	193.4	282.4	7.6	42.9
21AR234_280	113.4	16551.654	2.510	13.246	3.3	1.73086	3.8	0.16821	1.7	0.444	1002.2	15.5	1020.1	24.2	1059.4	67.9	1059.4	67.9	94.6
21AR234_281	334.8	26730.205	4.466	16.496	2.7	0.77041	3.6	0.09308	2.0	0.563	573.7	11.0	580.0	15.7	605.5	63.7	573.7	11.0	94.7
21AR234_282	325.8	4445.684	1.452	14.612	14.7	0.12914	15.5	0.01437	1.9	0.122	92.0	1.7	123.3	18.0	781.1	323.1	92.0	1.7	11.8
21AR234_283	327.4	50449.025	8.751	13.436	1.5	1.73625	2.0	0.16985	1.2	0.622	1011.3	11.6	1022.1	12.9	1046.2	31.6	1046.2	31.6	96.7
21AR234_284	170.6	10178.178	2.314	17.517	4.8	0.49137	5.4	0.06431	1.8	0.325	401.8	6.9	405.8	18.1	430.0	114.1	401.8	6.9	93.4
21AR234_285	563.5	5549.872	1.532	16.223	3.9	0.42777	9.2	0.05257	5.4	0.585	330.3	17.4	361.6	28.1	568.8	162.9	330.3	17.4	58.1
21AR234_286	409.6	21953.260	78.419	17.735	2.8	0.53832	3.9	0.07007	1.4	0.360	436.6	5.9	437.3	13.7	442.1	80.2	436.6	5.9	98.8
21AR234_287	868.7	20682.475	16.983	16.653	1.5	0.70905	2.3	0.08666	1.7	0.739	535.7	8.7	544.2	9.6	580.7	33.5	535.7	8.7	92.3
21AR234_288	195.3	509.633	0.981	13.301	9.2	0.25133	24.8	0.03946	2.0	0.081	249.5	4.9	227.7	50.6	8.8		249.5	4.9	2844.8
21AR234_289	255.8	18138.317	1.256	17.455	3.9	0.57446	4.3	0.07381	1.5	0.352	459.1	6.7	460.9	15.8	470.9	88.6	459.1	6.7	97.5
21AR234_290	97.0	4505.723	1.240	13.382	11.1	0.46545	11.6	0.04721	1.7	0.150	297.4	5.1	388.0	37.5	972.7	234.8	297.4	5.1	30.6
21AR234_291	228.9	55303.121	2.342	10.929	1.4	3.05824	2.0	0.24326	1.4	0.686	1403.6	17.6	1422.3	15.5	1451.2	28.1	1451.2	28.1	96.7

21AR234_292	153.7	6183.443	2.484	16.185	7.1	0.46106	10.1	0.05630	2.6	0.261	353.1	9.0	385.0	32.3	582.6	211.6	353.1	9.0	60.6
21AR234_293	240.4	7413.430	1.276	18.494	7.0	0.29513	10.0	0.04116	2.6	0.258	260.0	6.6	262.6	23.2	286.4	221.1	260.0	6.6	90.8
21AR234_294	222.9	12543.851	3.063	15.952	5.7	0.41896	8.1	0.04955	5.3	0.659	311.8	16.3	355.3	24.3	651.5	130.7	311.8	16.3	47.9
21AR234_295	171.9	19348.688	1.923	13.000	2.0	1.89785	2.6	0.18098	1.4	0.547	1072.3	13.8	1080.3	17.0	1097.4	42.9	1097.4	42.9	97.7
21AR234_296	591.5	8472.051	1.728	18.483	5.8	0.12099	7.5	0.01679	1.6	0.214	107.3	1.7	116.0	8.3	298.5	167.8	107.3	1.7	35.9
21AR234_297	361.4	12541.141	1.590	18.130	3.0	0.33530	4.1	0.04512	1.5	0.365	284.5	4.2	293.6	10.6	367.6	87.0	284.5	4.2	77.4
21AR234_298	114.7	13523.450	1.811	16.291	5.0	0.89363	5.7	0.10761	1.8	0.322	658.8	11.4	648.3	27.1	612.8	115.5	658.8	11.4	107.5
21AR234_299	178.7	48454.281	1.544	8.669	1.2	5.17610	1.8	0.32519	1.2	0.687	1815.0	19.3	1848.7	15.1	1887.6	23.3	1887.6	23.3	96.2
21AR234_300	205.7	8300.198	0.682	17.842	6.5	0.27890	7.4	0.03714	1.7	0.227	235.1	3.9	249.8	16.3	391.1	160.7	235.1	3.9	60.1
21AR234_301	117.3	5757.966	2.589	14.859	7.8	0.42675	8.9	0.04761	2.3	0.256	299.8	6.7	360.9	27.1	775.4	181.7	299.8	6.7	38.7
21AR234_302	198.2	8804.384	4.571	16.920	6.3	0.32883	7.4	0.04138	1.5	0.210	261.4	4.0	288.7	18.5	516.7	158.1	261.4	4.0	50.6
21AR234_303	428.1	32788.514	1.661	16.825	2.1	0.72640	2.4	0.08880	1.2	0.506	548.4	6.5	554.4	10.5	580.2	45.8	548.4	6.5	94.5
21AR234_304	724.2	93014.626	17.003	15.596	1.9	1.01331	2.7	0.11529	2.0	0.726	703.4	13.2	710.5	13.9	734.2	39.6	703.4	13.2	95.8
21AR234_305	334.0	21795.203	5.596	13.008	1.4	1.79420	3.6	0.17018	3.2	0.897	1013.1	30.2	1043.4	23.4	1108.2	31.7	1108.2	31.7	91.4
21AR234_306	254.2	13645.221	10.522	16.121	3.7	0.68189	4.8	0.08085	2.0	0.416	501.2	9.5	527.9	19.6	646.0	92.8	501.2	9.5	77.6
21AR234_307	164.9	19738.704	1.429	13.246	2.1	1.83246	2.8	0.17798	1.5	0.543	1055.9	14.6	1057.2	18.1	1060.6	46.6	1060.6	46.6	99.6
21AR234_308	527.3	2031.387	2.564	14.479	3.5	0.63386	19.6	0.07438	4.2	0.212	462.5	18.5	498.5	77.2	668.5	410.7	462.5	18.5	69.2
21AR234_309	237.4	13527.292	2.025	17.564	3.4	0.52884	4.2	0.06883	1.8	0.441	429.1	7.6	431.0	14.7	442.5	83.5	429.1	7.6	97.0
21AR234_310	324.2	15827.147	1.288	16.367	2.3	0.79990	3.2	0.09662	1.8	0.571	594.6	10.3	596.8	14.4	606.1	56.7	594.6	10.3	98.1
21AR234_311	308.0	14088.281	7.404	18.607	3.1	0.42412	4.9	0.05850	1.4	0.294	366.5	5.2	359.0	14.9	311.8	107.2	366.5	5.2	117.5
21AR234_312	337.6	17181.488	7.319	17.843	3.1	0.54817	4.1	0.07224	1.6	0.392	449.6	6.9	443.8	14.6	414.6	83.6	449.6	6.9	108.5
21AR234_313	330.2	98277.139	1.481	5.668	1.3	10.48011	2.0	0.43263	1.5	0.742	2317.6	28.7	2478.2	18.4	2613.3	22.2	2613.3	22.2	88.7
21AR234_314	466.0	14792.130	1.577	18.091	5.1	0.21667	6.4	0.02905	1.8	0.280	184.6	3.3	199.1	11.6	376.3	139.0	184.6	3.3	49.1
21AR234_315	526.8	38124.360	3.534	13.354	1.8	1.58470	3.8	0.15478	3.3	0.872	927.7	28.9	964.2	23.9	1049.3	37.9	1049.3	37.9	88.4
21AR234_316	575.7	522.670	1.541	4.212	57.3	0.02608	59.9	0.00088	15.3	0.256	5.7	0.9	26.1	15.5	2940.0	959.8	5.7	0.9	0.2
21AR234_317	69.9	3802.356	1.008	14.297	9.5	0.59316	11.8	0.06524	2.1	0.179	407.4	8.3	472.9	44.5	805.3	242.5	407.4	8.3	50.6
21AR234_318	195.8	2209.783	2.884	11.929	2.3	2.00246	3.9	0.18822	1.6	0.421	1111.7	16.6	1116.3	26.1	1126.2	69.8	1126.2	69.8	98.7
21AR234_319	308.3	19888.307	1.929	16.909	3.3	0.66437	3.7	0.08280	1.3	0.354	512.8	6.4	517.3	14.8	538.0	74.7	512.8	6.4	95.3
21AR234_320	174.5	15411.212	3.943	15.377	5.2	0.75633	8.9	0.08596	6.7	0.753	531.6	34.2	571.9	38.9	736.4	123.7	531.6	34.2	72.2
21AR234_321	689.0	257636.692	3.004	6.260	1.2	9.20018	1.8	0.41970	1.4	0.750	2259.1	26.4	2358.1	16.9	2445.6	20.6	2445.6	20.6	92.4
21AR234_322	128.8	4889.286	2.423	15.022	10.9	0.36764	14.6	0.04210	2.9	0.198	265.8	7.5	317.9	39.8	720.5	304.0	265.8	7.5	36.9
21AR234_323	172.1	5447.083	3.906	16.347	5.5	0.33499	8.6	0.04171	2.2	0.253	263.4	5.6	293.4	21.9	540.0	182.3	263.4	5.6	48.8
21AR234_324	361.9	16959.009	9.368	17.681	3.0	0.46793	3.6	0.06121	1.4	0.391	383.0	5.2	389.8	11.6	431.3	73.6	383.0	5.2	88.8
21AR234_325	179.2	9172.613	1.301	17.031	4.9	0.49568	6.1	0.06325	1.4	0.232	395.3	5.5	408.8	20.7	486.3	131.9	395.3	5.5	81.3
21AR234_326	277.4	10208.857	6.389	17.331	4.4	0.33150	5.0	0.04294	1.5	0.294	271.1	3.9	290.7	12.7	452.8	106.5	271.1	3.9	59.9
21AR234_327	100.5	5241.339	1.758	14.680	10.5	0.44390	13.3	0.04950	2.1	0.157	311.4	6.3	373.0	41.5	776.6	276.7	311.4	6.3	40.1
21AR234_328	224.3	5191.905	6.232	16.503	4.7	0.47353	15.4	0.05975	11.3	0.734	374.1	41.2	393.6	50.4	511.0	230.4	374.1	41.2	73.2
21AR234_329	563.9	22439.050	2.445	19.510	2.5	0.30699	3.4	0.04422	1.6	0.482	279.0	4.5	271.8	8.1	211.9	69.0	279.0	4.5	131.6
21AR234_330	142.3	7942.922	1.578	17.451	6.5	0.43389	6.9	0.05702	1.9	0.280	357.5	6.7	365.9	21.2	420.6	147.8	357.5	6.7	85.0
21AR234_331	268.9	5999.251	1.765	17.261	7.5	0.20035	9.3	0.02631	1.9	0.205	167.4	3.2	185.4	15.8	422.1	204.1	167.4	3.2	39.7
21AR234_332	817.9	60645.327	8.860	16.889	1.5	0.66955	2.0	0.08280	1.3	0.665	512.8	6.4	520.4	8.0	555.0	32.0	512.8	6.4	92.4
21AR234_333	142.1	2655.496	1.577	10.567	18.6	0.22168	20.4	0.01805	3.2	0.156	115.3	3.6	203.3	37.7	1406.4	387.6	115.3	3.6	8.2
21AR234_334	165.8	2084.773	0.520	11.934	22.7	0.12541	24.1	0.01188	5.3	0.221	76.1	4.0	120.0	27.2	1110.5	470.4	76.1	4.0	6.9
21AR234_335	260.8	19199.935	118.366	17.198	3.5	0.56331	4.2	0.07161	1.4	0.333	445.8	6.0	453.7	15.2	494.5	86.6	445.8	6.0	90.2
21AR234_336	291.5	14275.245	1.804	17.888	3.4	0.45915	4.0	0.06092	1.4	0.350	381.2	5.2	383.7	12.7	399.5	83.6	381.2	5.2	95.4
21AR234_337	188.3	3467.249	1.590	14.640	16.4	0.17311	18.3	0.01965	2.6	0.140	125.5	3.2	162.1	27.5	738.7	385.2	125.5	3.2	17.0
21AR234_338	275.1	10033.781	1.392	18.554	4.5	0.28515	5.4	0.03960	1.7	0.315	250.3	4.2	254.7	12.1	296.4	116.3	250.3	4.2	84.5
21AR234_339	176.9	5835.099	5.453	16.530	9.7	0.31813	10.5	0.03989	1.8	0.170	252.1	4.4	280.5	25.6	524.7	226.2	252.1	4.4	48.1
21AR234_340	301.1	11952.865	0.641	17.548	3.3	0.47326	4.9	0.06173	1.6	0.320	386.1	5.8	393.4	15.9	437.6	102.7	386.1	5.8	88.2
21AR234_341	149.8	9359.281	2.435	16.273	6.9	0.65821	8.8	0.07992	5.3	0.596	495.6	25.1	513.5	35.6	594.8	153.6	495.6	25.1	83.3
21AR234_342	291.7	16874.705	4.059	17.930	3.9	0.46985	4.8	0.06224	1.9	0.384	389.2	7.0	391.1	15.7	402.9	99.7	389.2	7.0	96.6
21AR234_343	273.6	2753.444	2.991	13.934	12.2	0.11247	14.5	0.01230	2.1	0.148	78.8	1.7	108.2	14.9	818.2	300.1	78.8	1.7	9.6
21AR234_344	215.0	27714.277	3.808	13.541	2.1	1.55192	2.4	0.15392	1.3	0.514	922.9	10.8	951.3	15.0	1018.3	42.3	1018.3	42.3	90.6
21AR234_345	123.0	12096.769	2.175	16.531	5.6	0.68680	6.3	0.08426	1.7	0.279	521.5	8.7	530.9	25.8	572.5	130.6	521.5	8.7	91.1
21AR234_346	98.0	32055.907	1.444	10.208	2.2	3.50759	4.3	0.26137	3.7	0.858	1496.8	49.3	1528.9	34.0	1574.4	41.3	1574.4	41.3	95.1
21AR234_347	202.8	8170.244	2.730	16.451	6.4	0.35523	8.5	0.04375	1.7	0.195	276.1	4.5	308.6	22.7	563.8	182.2	276.1	4.5	49.0
21AR234_348	93.0	7300.386	4.258	15.487	5.7	0.84108	7.9	0.09766	1.6	0.208	600.7	9.4	619.7	36.7	691.0	165.2	600.7	9.4	86.9
21AR234_349	435.2	26444.354	39.437	17.723	2.6	0.54483	3.7	0.07087	1.7	0.459	441.4	7.2	441.6	13.2	443.6	73.1	441.4	7.2	99.5
21AR234_350	404.5	55253.238	7.939	14.178	1.8	1.34377	3.0	0.13897	2.3	0.772	838.9	18.1	864.9	17.4	933.0	39.0	838.9	18.1	89.9
21AR234_351	320.1	20535.133	14.488	15.967	2.9	0.76735	5.5	0.09015	4.4	0.804	556.5	23.5	578.2	24.2	665.7	69.7	556.5	23.5	83.6
21AR234_352	539.1	163618.820	33.692	7.426	1.3	6.33991	2.0	0.34274	1.6	0.771	1899.8	25.8	2024.0	17.8	2153.8	22.6	2153.8	22.6	88.2
21AR234_353	267.9	30801.435	4.131	15.154	2.0	1.07070	2.6	0.11893	1.5	0.587	724.4	10.3	739.1	13.4	784.6	43.5	724.4	10.3	92.3
21AR234_354	183.4	5585.835	3.016	15.355	9.3	0.24043	10.3	0.02798	2.2	0.214	177.9	3.9	218.8	20.4	686.3	215.7	177.9	3.9	25.9
21AR234_355	174.2	10311.571	0.735	17.474	5.4	0.56366	6.6	0.07349	2.9	0.439	457.1	12.7	453.9	24.0	438.5	131.4	457.1	12.7	104.2
21AR234_356	118.0	1504.743	1.285	13.014	14.3	0.14640	27.7	0.01584	4.3	0.155	101.3	4.3	138.7	35.9	840.1	572.1	101.3	4.3	12.1
21AR234_357	387.6	86046.279	1.780	5.600	1.5	10.45398	2.4	0.42637	1.9	0.773	2289.3	35.7	2475.8	22.2	2633.4	25.2	2633.4	25.2	86.9
21AR234_358	385.0	8908.402	2.640	13.467	1.5	1.48473	2.7	0.14870	2.1	0.761	893.7	17.5	924.2	16.7	998.5	36.2	998.5	36.2	89.5
21AR234_359	467.3	7271.934	3.848	16.581	2.1	0.66748	3.3	0.08330	2.0	0.617	515.8	10.0	519.2	13.3	535.1	56.4	515.8	10.0	96.4
21AR234_360	278.8	8745.439	0.768	16.981	3.5	0.64078	6.2	0.08148	1.6	0.264	505.0	7.9	502.8	24.5	493.9	131.6	505.0	7.9	102.2
21AR234_361	464.8	94631.526	2.703	11.350	1.4	2.66635	2.1	0.22060	1.5	0.737	1285.1	17.7	1319.2	15.2	1375.9	26.8	1375.9	26.8	93.4
21AR234_362	274.8	15285.958	2.786	13.086	1.5	1.80292	2.4	0.17382	1.3	0.552	1033.1	12.6	1046.5	15.6	1075.4	40.1	1075.4	40.1	96.1
21AR234_363	411.0	12591.140	1.659	18.328	4.9	0.28590	5.5	0.03896	1.6	0.297	246.4	4.0	255.3	12.5	339.2	119.8	246.4	4.0	72.6
21AR234_364	640.1	29687.427	1.496	17.320	1.8	0.59353	2.7	0.07545	2.0	0.727	468.9	9.0	473.1	10.3	494.5	41.3	468.9	9.0	94.8
21AR234_365	61.0	5132.653	0.435	13.313	11.7	0.71437	12.1	0.07188	2.0	0.164	447.5	8.6	547.3	51.2	989.1	242.9	447.5	8.6	45.2
21AR234_366	100.2	14568.075	1.159	12.245	2.6	2.13490	3.3	0.19207	1.7	0.519	1132.6	17.7	1160.2	22.6	1212.9	55.1	1212.9	55.1	93.4
21AR234_367	477.1	11304.197	19.178	16.074	1.8	0.73355	3.6	0.08740	1.9	0.527	540.2	9.9	558.6	15.5	635.6	66.2	540.2	9.9	85.0
21AR234_368	110.9	4208.181	1.370	14.766	6.8	0.41361	8.3	0.04670	1.8	0.219	294.2	5.2	351.5	24.6	750.2	170.8	294.2	5.2	39.2
21AR234_369	96.6	1596.374	1.350	12.641	10.8	0.37469	21.6	0.03874	2.2	0.101	245.0	5.3	323.1	59.7	933.3	441.6	245.0	5.3	26.3
21AR234_370	225.1	7852.075	1.014	17.377	5.7	0.35916	7.7	0.04686	2.3	0.294	295.2	6.5	311.6	20.6	437.1	163.7	295.2	6.5	67.5
21AR234_371	163.8	17092.378	2.730	15.457	3.5	1.03370	4.1	0.11783	1.7	0.408	718.0	11.5	720.8	21.4	730.2	80.1	718.0	11.5	98.3
21AR234_372	150.7	25841.812	1.769	12.827	2.1	1.97130	2.9	0.18502	1.9	0.652	1094.3	18.9	1105.8	19.5	1129.1	43.6	1129.1	43.6	96.9
21AR234_373	327.7	48807.424	17.748	13.283	1.7	1.76753	2.7	0.17124	2.0	0.734	1019.0	19.0	1033.6	17.8	1065.6	37.5	1065.6	37.5	95.6
21AR234_374	231.6	23772.430	3.689	13.443	1.8	1.60083	2.7	0.15762	1.7	0.630	943.5	14.9	970.5	16.8	1033.0	42.2	1033.0	42.2	91.3
21AR234_375	422.2	30016.475	2.411	16.925	2.5	0.64051	3.9	0.07909	2.9	0.745	490.7	13.6	502.6	15.4	558.4	56.4	490.7	13.6	87.9
21AR234_376	256.9	15220.074	1.689	17.072	3.8	0.58385	4.5	0.07331	1.7	0.369	456.1	7.3	466.9	16.8	521.5	91.4	456.1	7.3	87.5
21AR234_377	160.8	9970.893	1.982	16.892	6.0	0.58748	6.3	0.07361	1.6	0.250	457.9	7.0	469.3	23.8	526.2	134.5	457.9	7.0	87.0
21AR234_378	303.9	15277.431	1.493	18.377	4.3	0.33280	5.1	0.04504	1.3	0.261	284.0	3.7	291.7	12.9	354.6	111.3	284.0	3.7	80.1
21AR234_379	115.4	7303.558	1.401	16.603	5.9	0.60133	6.8	0.07470	1.6	0.239	464.4	7.3	478.1	26.1	545.0	145.0	464.4	7.3	85.2
21AR234_380	90.4	4041.319	2.014	14.120	13.0	0.51674	15.2	0.05537	1.9	0.122	347.4	6.3	423.0	52.5	860.0	313.2	347.4	6.3	40.4
21AR234_381	468.9	29645.432	1.321	17.530	2.2	0.58316	2.9	0.07505	1.6	0.544	466.5	7.2	466.5	11.0	467.4	54.5	466.5	7.2	99.8
21AR234_382	80.6	14252.737	1.716	13.136	3.9	1.82342	4.5	0.17560	1.5	0.326	1042.9	14.2	1053.9	29.6	1077.6	85.7	1077.6	85.7	96.8
21AR234_383	330.7	23289.856	0.976	16.756	2.3	0.73580	2.9	0.09015	1.4	0.469	556.4	7.3	560.0	12.6	575.2	56.3	556.4	7.3	96.7
21AR234_384	90.7	4778.218	0.953	14.402	11.3	0.56014	12.1	0.06106	2.3	0.187	382.1	8.4	451.6	44.0	824.0	247.4	382.1	8.4	46.4
21AR234_385	1044.2	17322.026	37.563	17.516	1.4	0.54354	3.2	0.06997	1.9	0.610	436.0	8.1	440.8	11.3	466.6	55.4	436.0	8.1	93.4
21AR234_386	166.6	12548.223	2.297	15.405	4.8	0.74018	11.6	0.08406	10.3	0.887	520.3	51.6	562.5	50.3	738.0	114.0	520.3	51.6	70.5
21AR234_387	105.9	17203.399	5.964	12.524	2.9	2.10526	3.6	0.19292	1.8	0.500	1137.2	18.5	1150.5	24.5	1176.7	61.0	1176.7	61.0	96.6
21AR234_388	357.0	12187.974	2.257	18.481	4.4	0.29635	5.2	0.04056	2.0	0.374	256.3	4.9	263.5	12.2	329.4	110.4	256.3	4.9	77.8
21AR234_389	178.3	18029.883	2.787	13.173	2.6	1.57817	3.6	0.15265	2.3	0.648	915.8	19.7	961.6	22.1	1068.9	54.5	1068.9	54.5	85.7
21AR234_390	338.6	49386.478	12.138	13.269	1.6	1.73401	2.4	0.16802	1.6	0.690	1001.2	15.2	1021.2	15.3	1065.4	34.6	1065.4	34.6	94.0
21AR234_391	335.0	39451.542	13.277	16.473	2.4	0.74980	3.9	0.09029	2.8	0.729	557.3	15.1	568.1	16.8	612.6	57.2	557.3	15.1	91.0
21AR234_392	296.6	21929.908	1.978	16.938	3.7	0.67222	4.2	0.08367	1.8	0.426	518.0	8.9	522.1	17.1	540.9	83.1	518.0	8.9	95.8
21AR234_393	291.6	26578.188	2.661	12.737	1.5	1.83825	2.0	0.17098	1.3	0.642	1017.5	12.1	1059.2	13.2	1147.1	30.4	1147.1	30.4	88.7
21AR234_394	55.9	1870.107	0.694	10.903	12.7	0.45502	21.7	0.03928	4.2	0.196	248.4	10.3	380.8	68.9	1293.5	414.6	248.4	10.3	19.2
21AR234_395	204.2	5134.012	3.429	16.852	8.6	0.31690	11.3	0.04077	1.8	0.159	257.6	4.5	279.5	27.5	468.1	246.4	257.6	4.5	55.0
21AR234_396	216.8	17317.222	2.103	14.135	1.9	1.47549	3.4	0.15339	1.7	0.512	920.0	14.8	920.4	20.4	922.3	59.5	922.3	59.5	99.7
21AR234_397	263.3	15146.408	1.074	16.837	3.5	0.66179	4.2	0.08248	1.5	0.350	510.9	7.2	515.7	16.9	537.9	85.8	510.9	7.2	95.0
21AR234_398	269.9	2570.174	2.608	15.086	13.3	0.10288	15.5	0.01237	3.3	0.215	79.3	2.6	99.4	14.7	615.6	327.8	79.3	2.6	12.9
21AR234_399	366.7	4158.317	5.490	16.113	4.9	0.63718	10.3	0.07933	2.5	0.247	492.1	12.0	500.6	40.6	540.3	217.9	492.1	12.0	91.1
21AR234_400	228.7	4188.065	2.056	15.615	7.9	0.16164	9.0	0.01941	2.1	0.233	123.9	2.6	152.1	12.7	618.8	189.3	123.9	2.6	20.0
21AR234_401	477.0	35146.804	7.026	15.636	2.5	0.80481	9.9	0.09236	9.6	0.966	569.5	52.1	599.5	44.8	715.9	54.6	569.5	52.1	79.6
21AR234_402	242.0	3622.934	4.065	16.900	5.4	0.41461	7.6	0.05483	2.0	0.268	344.1	6.8	352.2	22.6	406.9	163.4	344.1	6.8	84.6
21AR234_403	278.2	20174.641	20.411	16.727	2.9	0.73393	3.9	0.09050	1.7	0.434	558.5	9.2	558.9	17.0	561.3	77.5	558.5	9.2	99.5
21AR234_404	381.8	74817.552	2.783	9.903	1.1	3.67214	2.3	0.26511	2.0	0.871	1515.9	27.3	1565.3	18.5	1633.4	21.2	1633.4	21.2	92.8
21AR234_405	73.5	1935.232	1.741	9.667	20.9	0.36738	24.4	0.02776	4.3	0.175	176.5	7.4	317.7	66.6	1548.0	453.0	176.5	7.4	11.4
21AR234_406	585.0	18218.088	3.716	13.546	1.3	1.46457	3.2	0.14591	2.6	0.796	878.0	21.1	915.9	19.5	1009.2	39.7	1009.2	39.7	87.0
21AR234_407	399.5	1656.755	1.635	16.078	5.1	0.40556	24.1	0.05529	3.4	0.142	346.9	11.5	345.7	70.6	338.2	542.1	346.9	11.5	102.6

21AR234_408	200.2	16253.753	2.917	17.803	4.0	0.54150	4.6	0.07140	1.5	0.322	444.6	6.3	439.4	16.3	413.4	96.5	444.6	6.3	107.5
21AR234_409	290.8	18687.467	0.997	17.735	3.7	0.54796	4.4	0.07175	1.7	0.396	446.7	7.5	443.7	15.7	429.0	89.6	446.7	7.5	104.1
21AR234_410	516.2	46802.057	148.274	16.319	2.0	0.75915	3.2	0.09066	2.3	0.725	559.4	12.5	573.5	14.1	630.8	47.8	559.4	12.5	88.7
21AR234_411	206.6	30935.665	1.619	13.185	1.9	1.63099	2.7	0.15753	1.6	0.572	943.0	13.7	982.2	17.2	1071.9	45.1	1071.9	45.1	88.0
21AR234_412	954.9	109954.836	22.277	11.894	1.3	2.24230	2.3	0.19447	1.8	0.798	1145.6	19.1	1194.4	16.0	1284.7	26.7	1284.7	26.7	89.2
21AR234_413	152.0	13359.782	1.999	16.262	4.4	0.80628	5.6	0.09728	1.6	0.295	598.5	9.4	600.4	25.3	608.5	115.2	598.5	9.4	98.4
21AR234_414	175.3	6323.970	3.369	13.610	2.7	1.51481	6.7	0.15496	3.2	0.474	928.7	27.7	936.4	41.3	955.3	121.4	955.3	121.4	97.2
21AR234_415	505.8	877.109	0.949	8.327	31.1	0.02886	36.4	0.00201	7.7	0.211	13.0	1.0	28.9	10.4	1697.3	662.0	13.0	1.0	0.8
21AR234_416	266.4	6414.023	1.652	16.692	6.7	0.21623	7.4	0.02733	1.5	0.199	173.8	2.5	198.8	13.4	507.0	159.5	173.8	2.5	34.3
21AR234_417	229.7	14684.812	1.581	17.103	5.2	0.46475	6.6	0.05891	2.4	0.365	369.0	8.6	387.6	21.2	500.9	135.0	369.0	8.6	73.7
21AR234_418	100.1	4961.783	1.026	15.494	7.7	0.59777	10.0	0.07055	1.7	0.172	439.5	7.3	475.8	38.0	656.0	211.4	439.5	7.3	67.0
21AR234_419	935.9	9075.586	1.322	17.709	9.0	0.07615	9.9	0.01010	2.4	0.242	64.8	1.6	74.5	7.1	400.7	216.0	64.8	1.6	16.2
21AR234_420	492.7	15708.329	2.563	18.739	2.7	0.29848	3.6	0.04144	1.5	0.414	261.8	3.8	265.2	8.4	296.7	74.7	261.8	3.8	88.2
21AR234_421	178.2	9250.518	4.279	17.261	5.5	0.42464	6.7	0.05485	2.3	0.339	344.2	7.7	359.4	20.4	459.2	140.7	344.2	7.7	75.0
21AR234_422	441.0	5455.514	1.820	16.843	6.2	0.12755	11.0	0.01636	1.9	0.172	104.6	2.0	121.9	12.6	474.4	238.8	104.6	2.0	22.1
21AR234_423	90.6	11623.255	1.571	13.243	4.9	1.53217	6.0	0.15015	1.6	0.271	901.8	13.6	943.4	36.7	1042.5	116.1	1042.5	116.1	86.5
21AR234_424	135.1	10881.054	3.427	15.936	4.6	0.82328	8.4	0.09758	6.0	0.709	600.2	34.3	609.9	38.7	646.9	127.9	600.2	34.3	92.8
21AR234_425	103.1	14005.437	1.242	13.160	3.8	1.79011	4.5	0.17398	2.2	0.494	1034.0	21.1	1041.9	29.1	1059.3	78.2	1059.3	78.2	97.6
21AR234_426	254.7	10259.786	5.490	18.367	4.5	0.31342	6.9	0.04304	2.2	0.324	271.6	6.0	276.8	16.7	322.0	148.3	271.6	6.0	84.3
21AR234_427	64.5	8890.357	1.557	13.040	6.1	1.70184	7.6	0.16500	1.6	0.216	984.5	14.9	1009.2	48.5	1064.2	148.9	1064.2	148.9	92.5
21AR234_428	314.5	24057.163	221.719	17.245	3.0	0.64757	3.6	0.08209	1.4	0.398	508.6	7.0	507.0	14.3	500.7	72.4	508.6	7.0	101.6
21AR234_429	456.3	21197.872	189.419	18.049	3.1	0.41498	4.2	0.05522	1.5	0.360	346.5	5.1	352.5	12.6	393.1	88.4	346.5	5.1	88.1
21AR234_430	280.1	14623.280	4.285	17.137	3.8	0.37412	4.2	0.04749	1.4	0.329	299.1	4.0	322.7	11.5	497.9	86.9	299.1	4.0	60.1
21AR234_431	418.2	34158.467	76.642	16.688	2.9	0.76225	3.6	0.09327	1.9	0.541	574.8	10.6	575.3	15.7	578.2	65.3	574.8	10.6	99.4
21AR234_432	148.9	3634.342	1.388	16.095	6.0	0.57157	9.2	0.07154	1.9	0.211	445.4	8.3	459.0	33.8	528.8	196.3	445.4	8.3	84.2
21AR234_433	488.7	12254.725	1.547	17.970	3.8	0.20549	4.6	0.02747	1.8	0.396	174.7	3.1	189.8	7.9	382.6	93.9	174.7	3.1	45.7
21AR234_434	139.4	7798.892	2.207	16.475	5.5	0.57421	6.3	0.07104	2.2	0.348	442.4	9.3	460.7	23.2	554.0	127.9	442.4	9.3	79.9
21AR234_435	109.1	4738.686	1.829	14.496	11.1	0.37887	13.5	0.04182	2.6	0.191	264.1	6.7	326.2	37.7	797.9	278.4	264.1	6.7	33.1
21AR234_436	155.9	32514.188	1.911	11.766	1.9	2.42881	2.7	0.20909	1.7	0.618	1224.0	18.6	1251.2	19.4	1299.1	41.1	1299.1	41.1	94.2
21AR234_437	540.5	1810.632	0.931	16.498	3.9	0.27245	14.1	0.03776	2.1	0.147	238.9	4.9	244.6	30.6	300.9	317.4	238.9	4.9	79.4
21AR234_438	273.1	34425.094	5.767	14.796	2.9	1.17861	7.7	0.12765	7.1	0.919	774.4	51.7	790.7	42.3	837.6	63.2	774.4	51.7	92.5
21AR234_439	91.6	5178.801	0.769	14.817	9.9	0.55955	11.4	0.06309	2.4	0.208	394.4	9.1	451.2	41.7	753.4	236.4	394.4	9.1	52.3
21AR234_440	259.1	7650.588	1.104	16.901	5.7	0.29286	6.9	0.03731	1.6	0.225	236.1	3.6	260.8	15.9	489.7	148.6	236.1	3.6	48.2
21AR234_441	385.8	12960.134	1.945	17.635	3.1	0.52123	5.0	0.06842	3.3	0.663	426.6	13.8	426.0	17.5	423.5	84.2	426.6	13.8	100.7
21AR234_442	263.6	7642.468	1.104	17.287	4.8	0.29092	5.7	0.03794	1.8	0.319	240.1	4.3	259.3	13.1	437.8	121.0	240.1	4.3	54.8
21AR234_443	238.5	1888.615	1.292	13.500	11.9	0.11795	16.1	0.01293	3.1	0.194	82.8	2.6	113.2	17.2	811.9	330.2	82.8	2.6	10.2
21AR234_444	133.1	24171.843	1.511	13.479	2.5	1.70616	3.2	0.16878	2.0	0.605	1005.4	18.2	1010.8	20.6	1023.5	52.0	1023.5	52.0	98.2
21AR234_445	93.0	5291.137	1.300	15.617	8.3	0.50123	10.3	0.05954	1.6	0.154	372.8	5.8	412.5	35.0	642.3	219.4	372.8	5.8	58.0
21AR234_446	95.0	2149.242	0.449	13.617	10.4	0.36513	17.1	0.03978	2.3	0.133	251.5	5.6	316.0	46.3	825.3	353.4	251.5	5.6	30.5
21AR234_447	646.7	21243.288	2.304	16.068	1.5	0.84519	3.2	0.10007	2.6	0.791	614.8	15.0	622.0	15.1	649.2	42.6	614.8	15.0	94.7
21AR234_448	85.5	1813.394	1.029	12.924	12.8	0.33224	18.8	0.03478	3.1	0.166	220.4	6.8	291.3	47.5	908.2	382.0	220.4	6.8	24.3
21AR234_449	472.7	7764.739	1.940	18.455	3.9	0.29666	8.3	0.04134	2.4	0.292	261.1	6.2	263.8	19.2	288.4	180.6	261.1	6.2	90.5
21AR234_450	370.4	92937.566	4.835	8.666	1.0	5.16806	2.3	0.32715	2.0	0.889	1824.5	32.0	1847.4	19.3	1873.9	18.8	1873.9	18.8	97.4
21AR234_451	957.3	19021.522	0.870	19.135	3.1	0.16645	3.6	0.02357	1.4	0.390	150.2	2.1	156.3	5.3	251.5	76.9	150.2	2.1	59.7
21AR234_452	454.2	11469.049	1.510	18.725	4.5	0.19788	6.1	0.02768	1.8	0.288	176.0	3.1	183.3	10.2	279.7	134.0	176.0	3.1	62.9
21AR234_453	430.0	14546.065	1.164	18.109	3.2	0.28612	4.4	0.03848	1.6	0.370	243.4	3.9	255.5	9.9	369.0	91.8	243.4	3.9	66.0
21AR234_454	335.3	88188.394	2.627	10.677	1.2	3.12391	1.9	0.24362	1.4	0.755	1405.5	18.1	1438.6	14.6	1488.7	23.6	1488.7	23.6	94.4
21AR234_455	350.1	11704.092	1.780	18.130	3.8	0.28837	4.3	0.03894	1.7	0.396	246.3	4.1	257.3	9.8	359.8	89.4	246.3	4.1	68.5
21AR234_456	111.4	6452.608	1.837	15.028	9.2	0.50089	10.4	0.05669	2.0	0.187	355.5	6.8	412.3	35.4	745.3	217.0	355.5	6.8	47.7
21AR234_457	400.7	15266.611	2.146	17.677	2.9	0.46701	4.1	0.06111	1.3	0.317	382.4	4.8	389.1	13.1	430.2	85.8	382.4	4.8	88.9
21AR234_458	1101.5	87626.313	1.439	17.020	1.5	0.68794	2.2	0.08542	1.6	0.731	528.4	8.2	531.6	9.2	546.0	32.9	528.4	8.2	96.8
21AR234_459	566.3	49649.974	8.244	14.964	1.7	0.99019	4.0	0.10831	3.6	0.900	662.9	22.7	698.8	20.3	817.0	36.5	662.9	22.7	81.1
21AR234_460	237.7	7915.751	1.458	17.513	5.4	0.33335	6.1	0.04390	1.3	0.214	277.0	3.5	292.1	15.5	415.9	132.9	277.0	3.5	66.6
21AR234_461	208.9	12720.037	3.464	17.081	4.5	0.58689	5.9	0.07441	3.3	0.565	462.7	14.8	468.9	22.0	500.3	106.6	462.7	14.8	92.5
21AR234_462	136.4	701.397	1.684	13.202	11.0	0.46514	66.5	0.06158	4.5	0.068	385.2	17.0	387.8	215.3	404.3		385.2	17.0	95.3
21AR234_463	89.4	4754.764	1.343	14.939	10.7	0.50414	11.8	0.05740	2.7	0.228	359.8	9.5	414.5	40.3	732.7	244.3	359.8	9.5	49.1
21AR234_464	221.8	38582.406	2.484	5.228	1.6	10.99510	2.5	0.41893	1.9	0.758	2255.6	35.9	2522.7	23.2	2745.9	26.8	2745.9	26.8	82.1
21AR234_465	526.9	32919.332	26.461	18.335	2.4	0.42033	2.9	0.05653	1.4	0.483	354.5	4.8	356.3	8.7	368.9	57.2	354.5	4.8	96.1
21AR234_466	417.5	121005.646	2.287	9.438	1.4	4.19031	2.4	0.28805	1.9	0.810	1631.8	27.6	1672.1	19.4	1723.9	25.4	1723.9	25.4	94.7
21AR234_467	150.0	2922.023	0.478	15.907	5.9	0.48058	10.6	0.06040	1.6	0.146	378.0	5.7	398.5	35.1	519.6	231.2	378.0	5.7	72.8
21AR234_468	146.1	7606.215	1.540	16.782	6.4	0.57484	9.0	0.07252	4.8	0.529	451.3	20.8	461.1	33.5	511.2	168.8	451.3	20.8	88.3
21AR234_469	426.8	28187.563	70.691	17.503	2.9	0.55182	4.2	0.07091	2.8	0.672	441.6	12.1	446.2	15.2	470.8	69.2	441.6	12.1	93.8
21AR234_470	368.8	14856.679	3.014	18.717	4.1	0.30670	5.8	0.04255	1.6	0.282	268.6	4.3	271.6	13.7	298.3	125.9	268.6	4.3	90.0
21AR234_471	224.7	63267.268	1.264	8.688	1.5	5.20903	2.3	0.32991	1.7	0.755	1837.9	27.4	1854.1	19.4	1873.0	26.9	1873.0	26.9	98.1
21AR234_472	630.6	23442.235	2.449	18.790	3.3	0.30847	4.3	0.04267	1.8	0.414	269.4	4.6	273.0	10.2	305.0	88.2	269.4	4.6	88.3
21AR234_473	106.3	17432.322	1.754	13.441	3.8	1.67328	4.2	0.16546	1.5	0.361	987.0	13.8	998.4	26.5	1024.4	78.7	1024.4	78.7	96.4
21AR234_474	61.7	3223.446	0.658	12.519	13.2	0.63719	15.5	0.06140	2.3	0.150	384.1	8.7	500.6	61.4	1076.6	308.7	384.1	8.7	35.7
21AR234_475	503.4	8798.260	1.087	17.568	4.6	0.12453	5.1	0.01639	1.6	0.320	104.8	1.7	119.2	5.8	416.8	108.6	104.8	1.7	25.2
21AR234_476	110.4	6663.154	1.591	15.219	8.5	0.49139	9.9	0.05626	2.0	0.205	352.8	7.0	405.9	33.2	720.8	206.5	352.8	7.0	49.0
21AR234_477	206.4	7868.437	2.248	16.852	5.5	0.34889	6.0	0.04415	1.6	0.262	278.5	4.3	303.9	15.8	504.5	127.8	278.5	4.3	55.2
21AR234_478	800.2	17212.197	1.485	18.901	2.7	0.26534	4.0	0.03708	1.4	0.354	234.7	3.3	239.0	8.6	281.8	86.4	234.7	3.3	83.3
21AR234_479	301.1	6758.094	2.998	16.754	8.8	0.22362	9.3	0.02827	2.1	0.226	179.7	3.7	204.9	17.2	507.1	198.5	179.7	3.7	35.4
21AR234_480	690.4	24205.882	4.941	13.272	1.3	1.60308	2.2	0.15601	1.6	0.726	934.6	13.7	971.4	13.5	1056.6	30.0	1056.6	30.0	88.4
21AR234_481	697.8	26351.175	3.499	18.800	2.7	0.34257	3.2	0.04730	1.4	0.451	297.9	4.1	299.1	8.2	309.4	64.0	297.9	4.1	96.3
21AR234_482	359.0	45759.760	6.724	13.880	1.8	1.31727	2.2	0.13346	1.2	0.558	807.5	9.2	853.3	12.6	975.1	36.8	807.5	9.2	82.8
21AR234_483	220.2	10179.884	1.184	16.479	7.1	0.30671	8.2	0.03761	1.5	0.180	238.0	3.4	271.6	19.4	573.3	174.6	238.0	3.4	41.5
21AR234_484	621.9	1467.271	2.562	9.548	30.0	0.03285	31.1	0.00251	6.1	0.198	16.1	1.0	32.8	10.0	1530.3	577.3	16.1	1.0	1.1
21AR234_485	157.0	63819.464	2.074	5.382	1.5	12.55242	2.2	0.49149	1.6	0.734	2577.1	35.0	2646.7	21.1	2701.0	25.1	2701.0	25.1	95.4
21AR234_486	299.7	5895.264	1.606	16.449	7.4	0.22317	9.5	0.02781	1.9	0.196	176.8	3.2	204.5	17.6	538.5	204.5	176.8	3.2	32.8
21AR234_487	641.4	25801.601	4.048	17.458	2.1	0.57914	3.3	0.07421	2.2	0.651	461.5	9.7	463.9	12.4	476.9	55.9	461.5	9.7	96.8
21AR234_488	400.6	36195.296	175.148	17.867	2.8	0.51862	3.2	0.06787	1.4	0.454	423.3	5.9	424.2	11.0	430.0	62.8	423.3	5.9	98.5
21AR234_489	94.7	1975.757	3.648	13.267	14.0	0.26102	15.2	0.02788	3.2	0.208	177.3	5.5	235.5	31.9	866.2	308.4	177.3	5.5	20.5
21AR234_490	323.0	107637.598	1.830	9.237	1.2	4.51328	1.9	0.30366	1.5	0.779	1709.4	21.9	1733.4	15.5	1763.3	21.4	1763.3	21.4	96.9
21AR234_491	308.2	15089.747	1.235	18.058	3.8	0.35543	4.4	0.04753	1.7	0.393	299.3	5.0	308.8	11.6	382.0	90.1	299.3	5.0	78.4
21AR234_492	94.5	6176.702	1.607	14.712	9.5	0.57710	9.9	0.06396	1.7	0.168	399.7	6.4	462.6	36.7	789.3	204.3	399.7	6.4	50.6
21AR234_493	183.9	24881.973	3.873	13.937	3.2	1.29448	8.8	0.13231	8.1	0.926	801.0	61.2	843.3	50.2	957.0	67.5	801.0	61.2	83.7
21AR234_494	318.3	12416.579	1.026	19.037	3.3	0.29763	4.3	0.04219	1.4	0.335	266.4	3.8	264.5	10.0	249.4	93.4	266.4	3.8	106.8
21AR234_495	238.7	8944.972	3.471	17.751	5.3	0.32419	6.5	0.04314	1.6	0.238	272.3	4.1	285.1	16.2	392.7	141.9	272.3	4.1	69.3
21AR234_496	71.8	3155.384	1.101	11.777	19.2	0.43670	20.0	0.03952	2.9	0.146	249.9	7.2	367.9	61.9	1201.2	391.8	249.9	7.2	20.8
21AR234_497	776.7	49727.492	1.446	17.756	1.7	0.55448	2.2	0.07205	1.3	0.594	448.5	5.6	447.9	7.9	446.1	39.2	448.5	5.6	100.5
21AR234_498	193.4	11277.011	0.801	17.200	4.4	0.50230	6.0	0.06432	1.7	0.282	401.8	6.6	413.3	20.5	478.5	128.0	401.8	6.6	84.0
21AR234_499	395.6	10703.842	1.045	18.155	5.7	0.21085	7.1	0.02857	1.5	0.215	181.6	2.7	194.3	12.6	352.2	157.2	181.6	2.7	51.6
21AR234_500	367.6	29250.860	1.670	17.008	2.6	0.64725	3.1	0.08073	1.6	0.518	500.5	7.7	506.8	12.3	536.3	57.5	500.5	7.7	93.3
21AR234_501	435.4	109044.210	87.984	10.321	4.6	3.09377	6.9	0.23286	5.1	0.743	1349.5	62.4	1431.1	52.9	1555.5	86.5	1555.5	86.5	86.8
21AR234_502	285.6	17025.677	33.774	17.585	3.9	0.53210	4.3	0.06919	1.5	0.349	431.3	6.3	433.2	15.2	444.5	90.1	431.3	6.3	97.0
21AR234_503	134.4	5481.095	1.994	15.621	9.8	0.35107	11.9	0.04166	2.3	0.192	263.1	5.9	305.5	31.5	644.4	251.7	263.1	5.9	40.8
21AR234_504	300.4	12242.249	1.827	18.210	5.0	0.29867	6.1	0.04049	1.8	0.288	255.9	4.4	265.4	14.3	351.0	132.7	255.9	4.4	72.9
21AR234_505	152.1	19742.454	15.434	13.339	3.9	1.62576	4.5	0.15961	1.9	0.434	954.6	17.2	980.2	28.2	1038.9	81.5	1038.9	81.5	91.9
21AR234_506	229.0	9453.276	1.829	18.015	3.9	0.32222	5.7	0.04349	1.6	0.284	274.4	4.4	283.6	14.2	361.0	124.1	274.4	4.4	76.0
21AR234_507	254.0	597.683	1.684	7.243	28.7	0.04809	38.9	0.00303	8.1	0.209	19.5	1.6	47.7	18.1	1880.4	692.8	19.5	1.6	1.0
21AR234_508	414.2	17373.879	0.752	18.308	4.2	0.27498	5.5	0.03724	2.3	0.413	235.7	5.2	246.7	12.0	353.4	112.9	235.7	5.2	66.7
21AR234_509	108.5	18771.557	4.711	13.523	2.9	1.63877	3.7	0.16306	1.4	0.391	973.8	13.1	985.2	23.4	1011.7	69.2	1011.7	69.2	96.3
21AR234_510	247.3	16120.893	7.094	17.122	3.9	0.58720	4.2	0.07444	1.4	0.342	462.8	6.5	469.1	15.9	500.8	87.4	462.8	6.5	92.4
21AR234_511	177.4	6881.981	2.470	17.390	5.7	0.32661	9.1	0.04288	2.0	0.219	270.7	5.3	287.0	22.7	423.0	198.1	270.7	5.3	64.0
21AR234_512	691.2	27151.228	4.688	18.792	2.5	0.30522	3.1	0.04221	1.3	0.439	266.5	3.5	270.5	7.3	305.7	62.8	266.5	3.5	87.2
21AR234_513	167.4	2030.567	0.878	15.731	7.1	0.51482	22.2	0.06649	1.9	0.085	415.0	7.6	421.7	76.6	459.4	491.6	415.0	7.6	90.3
21AR234_514	1057.1	21730.991	2.608	19.178	2.0	0.26565	2.8	0.03758	1.8	0.620	237.8	4.1	239.2	6.1	253.9	51.4	237.8	4.1	93.7
21AR234_515	403.9	21279.844	29.364	18.480	3.1	0.45306	3.5	0.06179	1.1	0.316	386.5	4.2	379.4	11.1	337.4	75.2	386.5	4.2	114.5
21AR234_516	77.4	801.883	3.041	6.780	31.3	0.17967	34.7	0.00999	9.7	0.279	64.1	6.2	167.8	53.7	2104.2	589.9	64.1	6.2	3.0
21AR234_517	416.2	6912.170	1.208	17.226	5.8	0.15435	8.8	0.02010	1.4	0.155	128.3	1.7	145.7	11.9	440.7	192.7	128.3	1.7	29.1
21AR234_518	151.6	11018.961	0.937	16.675	5.0	0.57883	5.7	0.07190	1.5	0.261	447.6	6.4	463.7	21.1	545.2	119.8	447.6	6.4	82.1
21AR234_519	1181.8	26492.329	4.927	19.183	2.0	0.26145	2.6	0.03694	1.4	0.547	233.8	3.3	235.8	5.5	257.1	50.0	233.8	3.3	90.9
21AR234_520	90.0	4057.497	0.974	14.609	10.1	0.50312	14.2	0.05636	1.9	0.132	353.4	6.5	413.8	48.3	767.0	297.0	353.4	6.5	46.1
21AR234_521	936.3	80.435	0.671	5.144	25.0	-0.00006	11109.2	0.00077	9.7	0.001	5.0	0.5	NA	-6.4	NA		5.0	0.5	

21AR238

21AR238_1	533.3	4170.684	1.032	17.504	6.0	0.28656	14.6	0.03896	2.6	0.176	246.4	6.2	255.8	33.1	344.2	325.9	246.4	6.2	71.6
21AR238_2	228.5	11835.804	4.356	17.943	3.9	0.30902	5.4	0.04128	1.6	0.289	260.8	4.0	273.4	13.0	384.1	116.2	260.8	4.0	67.9
21AR238_3	266.9	4808.815	0.978	13.512	12.4	0.12130	13.3	0.01244	2.8	0.209	79.7	2.2	116.2	14.6	950.8	267.0	79.7	2.2	8.4
21AR238_4	550.9	29322.428	4.625	18.918	2.9	0.30089	3.7	0.04185	1.8	0.481	264.3	4.6	267.1	8.7	292.5	74.2	264.3	4.6	90.4
21AR238_5	356.1	4662.009	5.522	15.335	2.7	0.79276	4.2	0.09296	1.7	0.411	573.0	9.4	592.7	18.8	669.8	81.5	573.0	9.4	85.6
21AR238_6	32.3	10723.121	6.756	11.715	6.8	2.34217	7.1	0.20296	1.8	0.253	1191.2	19.6	1225.2	50.5	1286.3	133.8	1286.3	133.8	92.6
21AR238_7	177.3	490.558	1.772	4.609	46.8	0.04189	50.8	0.00159	18.3	0.360	10.2	1.9	41.7	20.8	2756.4	791.9	10.2	1.9	0.4
21AR238_8	134.1	25703.380	7.999	13.569	2.9	1.70405	3.6	0.16986	2.0	0.562	1011.4	19.1	1010.1	23.2	1008.1	60.8	1008.1	60.8	100.3
21AR238_9	49.3	919.400	3.388	6.571	39.0	0.21774	46.2	0.01149	14.4	0.312	73.7	10.5	200.0	83.9	2195.5	773.0	73.7	10.5	3.4
21AR238_10	101.5	17181.806	6.097	11.575	2.4	2.48344	3.2	0.21132	1.6	0.499	1235.8	18.0	1267.2	23.3	1321.7	54.1	1321.7	54.1	93.5
21AR238_11	387.1	36734.771	28.297	14.414	2.6	0.78362	4.2	0.08272	3.2	0.759	512.3	15.8	587.5	18.9	890.9	57.0	512.3	15.8	57.5
21AR238_12	78.5	18461.845	5.260	12.818	3.5	1.89164	4.0	0.17833	1.9	0.464	1057.8	18.1	1078.2	26.6	1120.4	70.7	1120.4	70.7	94.4
21AR238_13	805.5	31885.027	60.972	13.531	1.2	1.68852	2.5	0.16741	2.1	0.852	997.8	19.4	1004.2	15.7	1019.0	26.2	1019.0	26.2	97.9
21AR238_14	196.7	13933.673	6.901	15.687	5.9	0.36816	6.6	0.04274	1.8	0.277	269.8	4.9	318.3	18.1	691.2	136.0	269.8	4.9	39.0
21AR238_15	424.0	21874.394	8.051	16.143	2.2	0.83573	3.6	0.09929	2.1	0.599	610.2	12.4	616.8	16.5	641.9	61.5	610.2	12.4	95.1
21AR238_16	229.8	18047.209	7.343	17.542	3.6	0.47017	4.7	0.06091	2.0	0.428	381.2	7.4	391.3	15.2	452.6	94.0	381.2	7.4	84.2
21AR238_17	342.1	19340.554	8.943	13.156	1.7	1.62296	2.3	0.15720	1.5	0.649	941.2	13.3	979.1	14.7	1066.1	35.8	1066.1	35.8	88.3
21AR238_18	124.5	28484.858	5.775	13.716	3.8	1.60690	4.2	0.16142	1.6	0.376	964.7	14.1	972.9	26.1	992.4	78.7	992.4	78.7	97.2
21AR238_19	371.6	17671.751	2.891	18.086	5.0	0.21278	5.7	0.02842	1.7	0.292	180.6	3.0	195.9	10.2	384.8	123.3	180.6	3.0	46.9
21AR238_20	129.6	11252.417	25.018	16.095	6.5	0.56250	8.6	0.06722	4.6	0.543	419.4	18.8	453.2	31.3	629.2	154.9	419.4	18.8	66.7
21AR238_21	257.4	9277.096	3.763	17.890	4.2	0.43196	6.3	0.05786	1.6	0.259	362.6	5.7	364.6	19.3	377.9	136.5	362.6	5.7	95.9
21AR238_22	158.2	11213.886	4.522	16.015	8.8	0.34165	9.3	0.04066	2.0	0.216	256.9	5.1	298.4	24.1	638.3	195.8	256.9	5.1	40.3
21AR238_23	234.7	36197.686	17.513	15.021	3.1	1.05594	3.6	0.11611	1.6	0.444	708.2	10.7	731.8	18.7	805.9	67.2	708.2	10.7	87.9
21AR238_24	178.7	88830.594	1.435	8.682	1.3	5.08437	2.3	0.32164	1.8	0.805	1797.7	28.4	1833.5	19.1	1875.1	24.1	1875.1	24.1	95.9
21AR238_25	985.5	14302.922	1.960	18.114	4.0	0.09156	4.9	0.01229	2.0	0.408	78.7	1.6	89.0	4.2	373.8	101.1	78.7	1.6	21.1
21AR238_26	211.4	45601.061	4.456	13.241	1.6	1.77061	2.1	0.17123	1.2	0.593	1018.9	11.6	1034.7	13.5	1069.3	33.5	1069.3	33.5	95.3
21AR238_27	256.6	1163.556	4.907	6.189	57.0	0.07317	58.0	0.00354	7.6	0.131	22.8	1.7	71.7	40.2	2347.7	1006.4	22.8	1.7	1.0
21AR238_28	455.5	47161.814	6.429	17.890	2.1	0.51663	3.3	0.06774	2.5	0.751	422.5	10.3	422.9	11.6	425.9	49.2	422.5	10.3	99.2
21AR238_29	412.4	25756.132	4.792	18.738	3.7	0.29264	4.1	0.04032	1.5	0.361	254.8	3.7	260.6	9.5	314.5	87.4	254.8	3.7	81.0
21AR238_30	76.7	4061.810	3.458	13.070	11.6	0.40598	12.3	0.04045	2.5	0.206	255.6	6.4	346.0	36.2	1008.9	244.9	255.6	6.4	25.3
21AR238_31	527.7	6087.825	55.474	15.456	2.3	0.74882	7.0	0.08739	3.1	0.447	540.1	16.1	567.5	30.3	680.1	133.1	540.1	16.1	79.4
21AR238_32	203.8	8560.187	4.506	16.512	6.8	0.31825	8.9	0.03936	2.2	0.250	248.8	5.4	280.6	21.7	555.0	187.2	248.8	5.4	44.8
21AR238_33	540.9	21016.429	2.589	18.876	2.9	0.28464	3.8	0.03966	2.2	0.596	250.7	5.5	254.3	8.5	289.0	69.0	250.7	5.5	86.7
21AR238_34	415.9	22790.697	4.388	18.675	3.7	0.31771	4.1	0.04374	1.8	0.432	276.0	4.8	280.1	10.1	316.1	84.4	276.0	4.8	87.3
21AR238_35	257.7	84987.927	4.285	11.375	1.3	2.76719	1.9	0.22970	1.4	0.726	1332.9	16.5	1346.7	14.1	1369.6	25.0	1369.6	25.0	97.3
21AR238_36	168.3	17993.899	3.926	17.847	4.4	0.46948	5.0	0.06192	1.7	0.344	387.3	6.5	390.8	16.4	412.7	105.9	387.3	6.5	93.9
21AR238_37	137.1	17179.238	2.646	16.601	5.2	0.66831	5.6	0.08195	1.6	0.286	507.8	7.7	519.7	22.6	573.4	115.8	507.8	7.7	88.6
21AR238_38	459.7	51721.124	15.501	12.637	1.3	2.08423	2.0	0.19260	1.4	0.702	1135.4	14.3	1143.6	13.5	1160.0	27.7	1160.0	27.7	97.9
21AR238_39	30.0	13429.609	2.592	9.075	5.1	4.35202	5.4	0.29064	1.4	0.259	1644.7	20.3	1703.3	44.6	1776.9	95.3	1776.9	95.3	92.6
21AR238_40	159.2	9625.707	3.575	15.391	8.1	0.34003	8.5	0.03906	2.1	0.252	247.0	5.2	297.2	21.8	713.7	174.0	247.0	5.2	34.6
21AR238_41	155.9	12616.338	8.049	16.679	5.4	0.42728	6.6	0.05298	2.3	0.351	332.8	7.5	361.2	20.1	549.2	135.4	332.8	7.5	60.6
21AR238_42	98.5	46175.828	3.226	8.109	1.6	5.81052	2.4	0.34438	1.8	0.753	1907.7	30.2	1948.0	21.1	1991.8	28.5	1991.8	28.5	95.8
21AR238_43	304.6	1065.901	1.791	14.597	5.9	0.29820	50.2	0.03970	4.1	0.082	251.0	10.2	265.0	117.2	391.6		251.0	10.2	64.1
21AR238_44	255.2	10432.349	1.956	16.314	7.9	0.20874	8.4	0.02541	1.8	0.219	161.7	2.9	192.5	14.7	589.5	178.1	161.7	2.9	27.4
21AR238_45	414.1	83428.309	4.736	13.029	1.4	1.78930	2.4	0.17037	1.9	0.804	1014.2	18.0	1041.6	15.5	1100.4	28.3	1100.4	28.3	92.2
21AR238_46	192.0	12840.082	47.362	17.336	5.6	0.33197	6.2	0.04280	1.6	0.262	270.2	4.3	291.1	15.8	463.1	133.6	270.2	4.3	58.3
21AR238_47	68.0	2524.938	3.848	10.803	5.5	2.58451	9.6	0.21655	1.5	0.155	1263.6	17.1	1296.3	70.2	1351.5	182.7	1351.5	182.7	93.5
21AR238_48	288.2	8128.327	3.954	13.680	1.5	1.44489	2.5	0.14763	2.0	0.785	887.7	16.5	907.8	15.2	957.8	32.0	957.8	32.0	92.7
21AR238_49	97.8	6506.506	0.959	16.330	7.0	0.51120	7.9	0.06313	2.6	0.336	394.6	10.1	419.3	27.0	558.1	161.6	394.6	10.1	70.7
21AR238_50	350.7	38307.925	10.171	13.509	1.4	1.53549	2.4	0.15210	1.9	0.800	912.7	16.4	944.7	14.8	1020.8	29.2	1020.8	29.2	89.4
21AR238_51	249.5	19842.073	3.326	17.989	4.5	0.44145	5.3	0.05872	2.0	0.382	367.8	7.3	371.3	16.5	393.9	110.0	367.8	7.3	93.4
21AR238_52	820.8	36897.886	8.118	17.443	1.8	0.56609	2.8	0.07263	1.7	0.597	452.0	7.3	455.5	10.3	474.3	49.6	452.0	7.3	95.3
21AR238_53	250.3	8233.219	6.755	12.109	1.7	2.13155	4.8	0.19226	2.8	0.578	1133.6	28.8	1159.1	33.1	1207.9	77.1	1207.9	77.1	93.8
21AR238_54	636.2	11224.672	21.164	16.368	4.0	0.52637	14.4	0.06420	13.2	0.915	401.1	51.4	429.4	50.5	585.1	126.1	401.1	51.4	68.6
21AR238_55	8.4	707.664	6.211	6.275	35.0	1.36550	38.1	0.07067	12.7	0.332	440.2	53.9	874.2	224.2	2229.8	627.4	2229.8	627.4	19.7
21AR238_56	173.5	31714.819	2.370	13.311	1.8	1.83889	2.9	0.17963	2.2	0.769	1064.9	21.6	1059.5	18.9	1049.1	37.0	1049.1	37.0	101.5
21AR238_57	228.1	12040.557	5.799	17.678	5.1	0.30449	5.6	0.04008	1.8	0.316	253.4	4.4	269.9	13.4	416.9	119.7	253.4	4.4	60.8
21AR238_58	575.3	75529.361	14.898	15.952	1.9	0.93851	3.2	0.10941	2.5	0.806	669.3	16.2	672.1	15.5	682.3	39.9	669.3	16.2	98.1
21AR238_59	292.2	6052.044	23.931	14.571	1.9	1.00281	6.1	0.11027	4.9	0.813	674.3	31.5	705.2	30.8	805.9	73.9	674.3	31.5	83.7
21AR238_60	761.0	9325.135	14.569	14.735	1.4	0.83413	2.5	0.09164	1.8	0.715	565.2	9.5	615.9	11.3	807.8	35.9	565.2	9.5	70.0
21AR238_61	108.9	21301.965	3.809	12.778	4.1	1.88426	4.5	0.17693	1.7	0.375	1050.2	16.3	1075.6	29.7	1128.3	82.6	1128.3	82.6	93.1
21AR238_62	247.5	13221.161	2.093	17.919	5.5	0.29595	6.4	0.03942	2.2	0.347	249.2	5.5	263.2	14.9	390.9	135.3	249.2	5.5	63.8
21AR238_63	260.2	16334.457	3.826	17.726	4.9	0.34702	6.1	0.04554	3.0	0.491	287.1	8.4	302.5	16.0	424.2	118.8	287.1	8.4	67.7
21AR238_64	243.8	9684.579	4.459	17.179	8.0	0.23503	9.7	0.03018	4.5	0.468	191.7	8.6	214.3	18.7	472.1	189.7	191.7	8.6	40.6
21AR238_65	153.1	12381.907	1.049	15.571	7.0	0.53129	8.2	0.06131	2.7	0.327	383.6	9.9	432.7	28.7	704.1	164.1	383.6	9.9	54.5
21AR238_66	179.3	10201.372	5.488	17.659	6.2	0.39403	6.6	0.05194	1.8	0.269	326.4	5.7	337.3	19.0	414.0	142.3	326.4	5.7	78.8
21AR238_67	62.3	12726.108	4.767	13.802	6.7	1.64114	7.2	0.16740	1.9	0.266	997.8	17.7	986.1	45.3	961.3	141.3	961.3	141.3	103.8
21AR238_68	369.4	17484.027	2.582	17.755	5.2	0.30518	5.8	0.04002	2.0	0.346	253.0	5.0	270.4	13.7	425.6	120.8	253.0	5.0	59.4
21AR238_69	935.4	91258.307	22.648	17.609	1.6	0.56506	2.6	0.07261	2.0	0.772	451.9	8.7	454.8	9.5	470.6	36.4	451.9	8.7	96.0
21AR238_70	154.2	28637.167	4.984	13.548	2.3	1.67863	3.0	0.16684	2.0	0.644	994.7	18.0	1000.5	19.3	1014.0	47.0	1014.0	47.0	98.1
21AR238_71	871.4	19071.077	23.098	17.442	1.8	0.53105	2.4	0.06830	1.3	0.544	425.9	5.4	432.5	8.5	468.7	44.7	425.9	5.4	90.9
21AR238_72	21.7	1010.517	1.385	8.792	26.7	0.51994	31.7	0.03779	13.2	0.417	239.1	31.0	425.1	110.2	1621.1	538.6	239.1	31.0	14.7
21AR238_73	101.2	1934.181	3.060	8.301	30.2	0.22179	31.8	0.01422	5.0	0.156	91.0	4.5	203.4	58.7	1850.6	572.1	91.0	4.5	4.9
21AR238_74	201.3	14497.514	1.514	17.010	4.2	0.46491	5.3	0.05854	2.1	0.396	366.7	7.5	387.7	17.0	515.6	106.7	366.7	7.5	71.1
21AR238_75	194.9	20861.665	4.328	16.937	4.5	0.66015	4.9	0.08229	1.8	0.375	509.8	8.9	514.7	19.6	537.7	98.7	509.8	8.9	94.8
21AR238_76	191.8	10120.143	3.305	17.455	6.6	0.36776	7.9	0.04791	1.9	0.241	301.7	5.6	318.0	21.6	440.2	171.2	301.7	5.6	68.5
21AR238_77	609.7	28806.809	2.142	19.214	3.2	0.27057	3.7	0.03820	1.8	0.473	241.7	4.2	243.2	8.1	258.5	75.8	241.7	4.2	93.5
21AR238_78	130.8	855.222	7.189	7.276	29.7	0.08477	33.8	0.00506	10.7	0.317	32.6	3.5	82.6	26.8	1977.7	575.5	32.6	3.5	1.6
21AR238_79	433.9	23496.436	3.445	18.636	3.5	0.28840	4.5	0.03958	1.9	0.415	250.2	4.6	257.3	10.3	323.4	93.7	250.2	4.6	77.4
21AR238_80	215.9	144616.032	12.348	4.187	1.4	17.18013	2.7	0.52425	2.3	0.860	2717.2	50.6	2944.9	25.5	3105.2	21.6	3105.2	21.6	87.5
21AR238_81	100.8	14039.585	5.069	15.095	5.2	1.05385	6.1	0.11771	2.6	0.424	717.4	17.5	730.8	31.6	773.1	115.8	717.4	17.5	92.8
21AR238_82	179.3	37447.165	4.127	13.386	1.7	1.65432	2.5	0.16217	1.6	0.636	968.8	14.3	991.2	15.8	1041.9	39.0	1041.9	39.0	93.0
21AR238_83	262.6	14293.804	4.334	16.923	4.9	0.34562	5.4	0.04340	1.9	0.354	273.9	5.1	301.4	14.0	521.4	109.8	273.9	5.1	52.5
21AR238_84	468.6	40892.147	33.046	16.470	2.2	0.79168	2.7	0.09565	1.5	0.557	588.9	8.6	592.1	12.3	605.6	49.3	588.9	8.6	97.2
21AR238_85	109.0	7925.562	3.172	15.759	7.3	0.51182	8.4	0.06056	2.4	0.280	379.1	8.7	419.7	28.8	650.4	172.9	379.1	8.7	58.3
21AR238_86	864.4	44745.542	89.154	18.325	2.7	0.36838	5.5	0.04953	4.7	0.848	311.6	14.3	318.5	15.1	369.7	66.0	311.6	14.3	84.3
21AR238_87	325.4	16790.307	2.471	18.014	4.3	0.30409	4.8	0.04064	1.9	0.407	256.8	4.9	269.6	11.3	383.1	97.9	256.8	4.9	67.0
21AR238_88	231.0	20255.832	3.929	17.386	4.3	0.56211	4.8	0.07218	1.9	0.387	449.3	8.1	452.9	17.7	472.2	98.9	449.3	8.1	95.2
21AR238_89	106.5	4972.049	1.378	14.052	10.9	0.35139	13.7	0.03754	3.2	0.235	237.6	7.5	305.8	36.2	866.2	276.6	237.6	7.5	27.4
21AR238_90	234.9	4484.355	1.736	14.823	12.2	0.14284	13.8	0.01622	3.0	0.217	103.7	3.1	135.6	17.5	738.6	284.4	103.7	3.1	14.0
21AR238_91	237.4	34696.041	4.593	16.668	3.0	0.69178	3.8	0.08469	2.2	0.577	524.1	11.1	533.9	15.9	577.0	68.1	524.1	11.1	90.8
21AR238_92	83.5	9034.381	5.206	15.129	7.0	0.82318	7.8	0.09301	2.6	0.332	573.3	14.1	609.8	35.6	748.7	154.6	573.3	14.1	76.6
21AR238_93	599.6	18875.573	3.396	18.378	2.9	0.31648	4.0	0.04304	2.7	0.668	271.6	7.2	279.2	9.9	344.0	68.0	271.6	7.2	79.0
21AR238_94	344.4	25946.386	6.907	17.076	2.4	0.63153	3.3	0.07942	1.8	0.540	492.6	8.4	497.1	12.9	518.3	60.6	492.6	8.4	95.0
21AR238_95	376.5	16731.493	4.286	18.135	3.3	0.32365	3.9	0.04350	1.7	0.442	274.5	4.6	284.7	9.6	370.5	78.0	274.5	4.6	74.1
21AR238_96	137.0	42971.646	2.904	11.260	1.9	2.50985	2.6	0.20688	1.8	0.678	1212.2	19.4	1274.9	18.8	1383.1	36.6	1383.1	36.6	87.6
21AR238_97	371.9	25527.117	10.845	16.992	2.6	0.64581	3.4	0.08082	2.1	0.609	501.0	10.1	505.9	13.6	529.0	59.4	501.0	10.1	94.7
21AR238_98	156.4	3886.259	1.756	13.929	10.9	0.23287	12.9	0.02494	2.5	0.197	158.8	4.0	212.6	24.7	861.2	262.7	158.8	4.0	18.4
21AR238_99	187.7	64827.638	1.496	11.476	1.4	2.70450	2.2	0.22438	1.7	0.762	1305.0	19.8	1329.7	16.3	1370.5	27.4	1370.5	27.4	95.2
21AR238_100	633.5	29698.563	2.690	16.098	1.5	0.86858	2.9	0.10269	1.9	0.664	630.2	11.6	634.8	13.7	652.3	46.8	630.2	11.6	96.6
21AR238_101	323.4	23748.518	5.008	16.861	3.5	0.50138	8.6	0.06226	7.6	0.886	389.4	28.7	412.6	29.1	545.9	86.9	389.4	28.7	71.3
21AR238_102	129.8	31747.113	4.657	13.109	2.4	1.93734	3.5	0.18621	2.5	0.702	1100.8	24.9	1094.1	23.5	1081.5	50.2	1081.5	50.2	101.8
21AR238_103	246.7	14716.014	6.018	17.032	3.7	0.54323	4.2	0.06860	1.6	0.392	427.7	6.8	440.5	15.0	509.3	85.0	427.7	6.8	84.0
21AR238_104	128.1	41952.890	2.267	12.439	2.3	2.04280	2.8	0.18601	1.6	0.568	1099.7	16.2	1129.9	19.3	1189.2	45.9	1189.2	45.9	92.5
21AR238_105	196.7	18691.532	8.486	16.806	4.9	0.65218	5.4	0.08085	2.0	0.380	501.2	9.9	509.8	21.6	549.5	108.6	501.2	9.9	91.2
21AR238_106	277.5	13622.054	1.875	18.363	4.9	0.29222	6.3	0.03981	2.1	0.336	251.7	5.2	260.3	14.4	339.6	133.8	251.7	5.2	74.1
21AR238_107	118.8	3801.599	2.923	15.006	9.7	0.21008	14.2	0.02432	3.1	0.219	154.9	4.7	193.6	25.0	697.5	295.3	154.9	4.7	22.2
21AR238_108	79.5	7951.149	3.849	15.813	6.6	0.69119	7.2	0.08185	2.1	0.297	507.2	10.4	533.5	29.8	648.8	147.1	507.2	10.4	78.2
21AR238_109	149.5	14825.616	3.714	17.702	5.3	0.46258	5.9	0.06062	2.2	0.380	379.4	8.2	386.1	18.8	427.2	121.0	379.4	8.2	88.8
21AR238_110	176.3	38183.904	3.770	12.970	2.2	1.81889	3.0	0.17273	1.9	0.646	1027.1	18.3	1052.3	19.5	1105.7	45.5	1105.7	45.5	92.9
21AR238_111	227.0	13939.292	4.507	17.809	5.3	0.32058	6.1	0.04232	2.0	0.323	267.2	5.2	282.3	15.1	410.9	129.4	267.2	5.2	65.0
21AR238_112	275.1	28883.205	5.321	16.408	2.6	0.78260	3.7	0.09418	2.1	0.578	580.2	11.7	587.0	16.3	614.2	64.5	580.2	11.7	94.5
21AR238_113	261.2	26777.492	5.042	16.782	3.8	0.65217	4.4	0.08032	1.9	0.426	498.1	9.1	509.8	17.8	563.8	87.5	498.1	9.1	88.3
21AR238_114	534.7	6701.736	1.486	17.216	4.9	0.12996	6.9	0.01691	1.7	0.251	108.1	1.8	124.1	8.0	442.6	148.1	108.1	1.8	24.4
21AR238_115	52.9	2975.739	1.657	12.492	15.3	0.40852	16.8	0.03949	4.4	0.259	249.7	10.7	347.8	49.5	1070.3	326.7	249.7	10.7	23.3
21AR238_116	128.6	5162.255	2.491	15.791	5.6	0.75795	9.4	0.09112	2.8	0.299	562.1	15.2	572.8	41.3	616.4	194.3	562.1	15.2	91.2

21AR238_117	146.3	11089.607	3.131	16.978	4.4	0.57511	5.8	0.07274	1.6	0.278	452.6	7.0	461.3	21.5	505.7	122.6	452.6	7.0	89.5
21AR238_118	280.4	11811.204	2.447	17.273	5.2	0.31286	6.3	0.04024	1.9	0.301	254.3	4.7	276.4	15.1	468.8	132.1	254.3	4.7	54.2
21AR238_119	373.2	18779.191	2.743	18.722	3.9	0.27596	4.4	0.03821	1.7	0.400	241.7	4.1	247.4	9.6	303.3	91.4	241.7	4.1	79.7
21AR238_120	284.3	11907.267	5.013	17.184	4.1	0.29466	4.6	0.03769	1.6	0.347	238.5	3.7	262.2	10.7	480.9	95.6	238.5	3.7	49.6
21AR238_121	58.0	1135.774	3.023	6.887	25.7	0.23258	28.5	0.01268	9.2	0.324	81.3	7.5	212.3	54.6	2138.5	473.9	81.3	7.5	3.8
21AR238_122	180.6	11534.166	9.863	16.544	5.7	0.46328	6.4	0.05698	2.0	0.305	357.2	6.8	386.5	20.7	566.8	133.8	357.2	6.8	63.0
21AR238_123	225.4	8297.107	11.300	13.303	2.5	1.56657	4.3	0.15521	3.0	0.706	930.1	26.2	957.1	26.6	1020.4	61.5	1020.4	61.5	91.2
21AR238_124	87.2	11306.272	2.039	14.762	7.6	0.82965	8.0	0.09084	2.1	0.258	560.5	11.1	613.4	36.9	814.9	162.0	560.5	11.1	68.8
21AR238_125	113.9	9058.717	2.586	16.070	6.7	0.57753	8.5	0.06932	2.1	0.250	432.1	8.9	462.9	31.7	619.6	178.5	432.1	8.9	69.7
21AR238_126	198.4	11291.218	1.905	16.596	7.7	0.32536	8.4	0.04016	2.4	0.285	253.8	5.9	286.0	20.9	559.4	175.2	253.8	5.9	45.4
21AR238_127	205.0	21732.327	14.618	16.455	3.1	0.79954	3.7	0.09681	1.9	0.519	595.7	10.9	596.6	16.7	600.8	68.5	595.7	10.9	99.1
21AR238_128	287.5	26170.114	2.883	17.513	4.3	0.55868	4.8	0.07201	2.1	0.429	448.2	8.9	450.7	17.4	464.0	96.0	448.2	8.9	96.6
21AR238_129	45.3	10766.334	3.077	12.679	7.0	1.75572	7.7	0.16496	2.2	0.292	984.3	20.5	1029.3	49.8	1127.0	146.8	1127.0	146.8	87.3
21AR238_130	166.5	11116.155	9.172	17.298	4.8	0.46337	5.7	0.05978	2.2	0.378	374.3	7.8	386.6	18.3	461.8	117.0	374.3	7.8	81.1
21AR238_131	588.8	10651.815	3.409	18.304	3.9	0.20355	6.0	0.02786	1.6	0.270	177.1	2.8	188.1	10.3	329.3	131.4	177.1	2.8	53.8
21AR238_132	78.2	3844.795	1.422	12.569	18.5	0.42030	20.8	0.04038	2.4	0.115	255.2	6.0	356.3	62.6	1082.2	416.2	255.2	6.0	23.6
21AR238_133	43.8	1548.899	1.927	9.739	21.2	0.35638	24.4	0.02772	6.8	0.278	176.3	11.8	309.5	65.2	1493.6	445.8	176.3	11.8	11.8
21AR238_134	403.9	62805.733	6.546	15.255	2.0	1.11625	2.8	0.12453	2.0	0.692	756.6	14.0	761.2	15.2	775.5	43.1	756.6	14.0	97.6
21AR238_135	187.9	9081.976	4.834	16.072	6.5	0.36429	7.1	0.04378	2.1	0.296	276.2	5.7	315.4	19.3	617.2	147.0	276.2	5.7	44.8
21AR238_136	265.3	46264.257	2.151	13.471	1.7	1.70267	2.5	0.16779	1.8	0.708	999.9	16.6	1009.5	16.2	1031.3	36.1	1031.3	36.1	97.0
21AR238_137	146.0	19412.234	5.506	15.246	4.5	0.94124	11.0	0.10574	9.9	0.905	648.0	61.2	673.5	54.0	760.9	98.2	648.0	61.2	85.2
21AR238_138	176.0	8504.691	2.266	17.483	7.1	0.29985	7.7	0.03937	2.3	0.297	248.9	5.6	266.3	18.0	422.7	164.2	248.9	5.6	58.9
21AR238_139	476.6	44485.562	4.687	18.055	2.5	0.46991	3.2	0.06219	1.9	0.592	388.9	7.1	391.1	10.3	405.1	57.5	388.9	7.1	96.0
21AR238_140	324.8	21579.018	25.902	18.165	4.0	0.34474	4.6	0.04624	1.6	0.349	291.4	4.6	300.8	12.0	374.9	97.4	291.4	4.6	77.7
21AR238_141	149.0	6033.711	1.914	14.866	9.0	0.32560	9.9	0.03660	2.4	0.240	231.7	5.4	286.2	24.7	759.5	202.6	231.7	5.4	30.5
21AR238_142	132.7	36746.524	4.316	11.812	2.3	2.24271	4.1	0.19408	3.3	0.811	1143.5	34.4	1194.5	28.5	1288.9	46.1	1288.9	46.1	88.7
21AR238_143	388.0	3806.361	7.904	17.839	4.7	0.29683	15.1	0.04143	2.0	0.130	261.7	5.0	263.9	35.2	284.9	343.6	261.7	5.0	91.9
21AR238_144	167.5	10374.712	4.604	16.012	10.3	0.33885	11.0	0.04039	2.0	0.178	255.3	4.9	296.3	28.4	634.7	233.8	255.3	4.9	40.2
21AR238_145	86.3	4452.359	1.505	13.545	10.9	0.39583	11.7	0.04080	3.7	0.317	257.8	9.4	338.6	33.8	940.0	228.0	257.8	9.4	27.4
21AR238_146	197.4	36574.945	3.726	13.018	2.7	1.63974	6.7	0.15614	6.1	0.912	935.3	53.5	985.6	42.5	1100.3	55.3	1100.3	55.3	85.0
21AR238_147	293.5	1433.394	3.892	15.245	7.5	0.29428	35.5	0.03865	1.9	0.053	244.4	4.5	261.9	82.0	422.3	796.6	244.4	4.5	57.9
21AR238_148	401.6	3631.190	1.692	15.117	10.6	0.10732	13.7	0.01258	2.7	0.197	80.6	2.2	103.5	13.5	670.6	288.5	80.6	2.2	12.0
21AR238_149	29.2	1380.407	1.106	9.280	31.8	0.52594	35.3	0.03920	5.7	0.162	247.9	13.9	429.1	123.6	1573.9	657.0	247.9	13.9	15.7
21AR238_150	381.4	41814.488	16.824	14.353	1.6	1.24604	4.7	0.13094	4.4	0.935	793.2	33.1	821.6	26.7	900.1	34.8	793.2	33.1	88.1
21AR238_151	253.5	32159.922	29.867	16.849	3.0	0.64926	4.8	0.08050	3.6	0.759	499.1	17.5	508.0	19.2	549.4	68.3	499.1	17.5	90.8
21AR238_152	217.0	13514.810	2.707	17.734	5.2	0.34186	5.8	0.04503	1.8	0.316	284.0	5.1	298.6	15.0	415.5	122.8	284.0	5.1	68.3
21AR238_153	255.5	7050.620	1.363	17.445	8.2	0.28975	10.7	0.03821	5.5	0.518	241.7	13.1	258.4	24.4	413.2	204.7	241.7	13.1	58.5
21AR238_154	218.4	18836.163	22.074	17.320	4.0	0.58058	4.6	0.07426	2.1	0.457	461.7	9.5	464.8	17.3	481.1	91.3	461.7	9.5	96.0
21AR238_155	32.9	1320.299	2.752	8.181	27.7	0.47347	29.9	0.03083	6.9	0.229	195.8	13.2	393.6	97.7	1822.7	531.3	195.8	13.2	10.7
21AR238_156	255.5	22874.372	2.951	17.260	2.8	0.65909	3.8	0.08386	1.6	0.429	519.1	8.1	514.1	15.3	492.5	75.5	519.1	8.1	105.4
21AR238_157	265.9	123148.875	2.219	8.672	1.2	5.14472	2.2	0.32560	1.8	0.825	1817.0	28.3	1843.5	18.4	1874.4	22.0	1874.4	22.0	96.9
21AR238_158	292.3	43247.708	24.183	13.692	1.9	1.58714	4.6	0.15916	4.1	0.903	952.1	36.4	965.2	28.4	996.0	39.8	996.0	39.8	95.6
21AR238_159	139.4	8498.468	1.704	14.872	9.0	0.39597	9.4	0.04405	2.1	0.228	277.9	5.8	338.7	27.1	781.6	192.4	277.9	5.8	35.5
21AR238_160	164.6	9719.401	3.175	15.637	7.7	0.35942	8.4	0.04198	1.9	0.222	265.1	4.9	311.8	22.6	678.1	175.2	265.1	4.9	39.1
21AR238_161	207.3	51355.776	4.434	12.654	1.5	2.04367	2.1	0.18951	1.5	0.688	1118.7	14.9	1130.2	14.4	1153.1	30.3	1153.1	30.3	97.0
21AR238_162	77.7	14822.238	6.981	13.293	4.6	1.74062	5.5	0.17092	1.9	0.351	1017.2	18.0	1023.7	35.2	1038.5	103.2	1038.5	103.2	97.9
21AR238_163	536.4	14040.682	3.056	18.537	3.6	0.28668	4.7	0.03952	1.8	0.387	249.8	4.5	255.9	10.7	313.2	99.1	249.8	4.5	79.8
21AR238_164	209.7	17763.830	8.133	17.071	4.6	0.66752	5.6	0.08428	2.6	0.456	521.6	12.9	519.2	22.9	509.6	110.4	521.6	12.9	102.4
21AR238_165	105.8	22339.065	2.929	12.425	2.4	2.19606	3.1	0.20056	1.7	0.566	1178.3	18.6	1179.8	21.3	1183.3	49.8	1183.3	49.8	99.6
21AR238_166	108.7	23137.865	2.373	14.026	4.3	1.36219	4.7	0.14030	1.5	0.313	846.4	11.6	872.8	27.5	941.4	91.3	846.4	11.6	89.9
21AR238_167	143.0	12102.610	1.111	15.904	5.8	0.54633	6.3	0.06447	2.0	0.316	402.8	7.8	442.6	22.7	656.2	128.5	402.8	7.8	61.4
21AR238_168	416.4	46699.875	4.825	17.092	2.2	0.68156	2.9	0.08526	1.8	0.618	527.4	9.1	527.7	11.9	530.0	49.9	527.4	9.1	99.5
21AR238_169	117.7	5341.281	2.311	14.418	12.8	0.30787	14.3	0.03362	2.2	0.152	213.2	4.6	272.5	34.1	820.5	294.6	213.2	4.6	26.0
21AR238_170	205.5	25477.622	5.411	16.783	4.3	0.69714	5.5	0.08605	3.2	0.589	532.2	16.5	537.1	22.9	559.0	96.6	532.2	16.5	95.2
21AR238_171	459.8	7754.371	64.724	17.039	2.4	0.57000	4.1	0.07327	2.2	0.523	455.8	9.5	458.0	15.3	470.0	78.3	455.8	9.5	97.0
21AR238_172	515.6	37672.153	3.379	18.836	2.6	0.42164	3.1	0.05833	1.7	0.548	365.4	6.0	357.2	9.3	305.2	58.9	365.4	6.0	119.7
21AR238_173	164.8	22129.020	4.664	13.975	2.5	1.41017	3.1	0.14498	1.5	0.493	872.7	12.3	893.2	18.2	945.2	54.6	945.2	54.6	92.3
21AR238_174	475.0	107071.490	7.421	13.068	1.6	1.84691	2.6	0.17634	2.1	0.791	1046.9	20.1	1062.3	17.3	1095.0	32.1	1095.0	32.1	95.6
21AR238_175	149.2	13208.533	2.849	17.278	5.8	0.56128	6.5	0.07210	2.2	0.339	448.8	9.6	452.4	23.8	471.4	135.7	448.8	9.6	95.2
21AR238_176	74.8	1041.187	3.806	6.623	35.3	0.19567	37.0	0.01031	9.3	0.250	66.1	6.1	181.5	61.5	2198.7	627.9	66.1	6.1	3.0
21AR238_177	51.2	4063.114	2.743	13.752	11.1	0.61096	12.5	0.06440	2.7	0.218	402.4	10.6	484.2	48.1	893.7	251.9	402.4	10.6	45.0
21AR238_178	144.3	7996.743	4.382	15.795	5.7	0.35522	8.2	0.04213	2.0	0.243	266.0	5.2	308.6	21.9	645.7	171.6	266.0	5.2	41.2
21AR238_179	75.6	4030.800	2.200	14.796	12.0	0.35618	13.4	0.04057	3.6	0.272	256.4	9.2	309.4	35.7	731.6	273.3	256.4	9.2	35.0
21AR238_180	152.6	17676.749	7.248	16.222	3.8	0.82006	6.5	0.09829	5.3	0.804	604.4	30.4	608.1	30.0	622.8	84.0	604.4	30.4	97.0
21AR238_181	35.0	1880.255	1.965	9.082	21.1	0.55986	24.3	0.03977	8.1	0.332	251.4	19.9	451.4	88.6	1663.6	425.7	251.4	19.9	15.1
21AR238_182	1209.0	56109.507	2.060	19.267	2.0	0.26892	2.4	0.03797	1.4	0.564	240.2	3.2	241.8	5.2	258.6	45.7	240.2	3.2	92.9
21AR238_183	388.5	31672.056	4.680	17.435	2.4	0.56866	3.1	0.07287	1.8	0.574	453.4	7.9	457.1	11.5	477.0	56.6	453.4	7.9	95.0
21AR238_184	58.7	588.180	3.193	11.380	17.2	0.36245	76.7	0.04173	4.2	0.055	263.6	10.9	314.0	207.8	708.7		263.6	10.9	37.2
21AR238_185	496.5	57462.833	2.004	16.767	2.1	0.73530	2.9	0.09024	1.8	0.633	557.0	9.8	559.7	12.5	571.6	49.0	557.0	9.8	97.4
21AR238_186	345.7	36556.488	22.136	17.319	3.1	0.57699	3.8	0.07335	1.8	0.472	456.3	7.8	462.5	13.9	494.4	72.9	456.3	7.8	92.3
21AR238_187	100.0	4291.093	2.333	14.744	9.9	0.34574	13.7	0.03910	2.9	0.210	247.2	6.9	301.5	35.6	747.0	282.4	247.2	6.9	33.1
21AR238_188	80.6	2440.770	2.582	13.290	12.3	0.39482	18.2	0.04149	2.9	0.158	262.0	7.4	337.9	52.4	900.2	372.3	262.0	7.4	29.1
21AR238_189	327.1	8551.515	6.495	17.326	3.6	0.45473	6.5	0.05918	1.7	0.256	370.6	6.0	380.6	20.5	442.5	138.9	370.6	6.0	83.8
21AR238_190	107.1	6627.677	2.748	14.554	10.3	0.37914	10.9	0.04154	2.2	0.199	262.3	5.6	326.4	30.5	813.7	223.8	262.3	5.6	32.2
21AR238_191	348.3	21965.598	2.443	18.254	3.8	0.35879	4.5	0.04835	1.6	0.355	304.4	4.8	311.3	12.1	364.4	95.2	304.4	4.8	83.5
21AR238_192	154.9	543.520	1.245	5.361	40.6	0.06666	45.2	0.00298	18.8	0.416	19.2	3.6	65.5	28.7	2480.1	701.1	19.2	3.6	0.8
21AR238_193	234.4	14552.303	5.030	17.471	3.7	0.48320	5.3	0.06269	2.1	0.405	392.0	8.1	400.3	17.5	449.4	107.2	392.0	8.1	87.2
21AR238_194	91.8	18057.980	2.396	12.911	3.7	1.87043	4.3	0.17800	2.3	0.527	1056.1	22.2	1070.7	28.6	1101.4	73.6	1101.4	73.6	95.9
21AR238_195	485.2	28599.260	3.516	17.715	2.5	0.54013	3.5	0.07045	1.9	0.543	438.9	8.1	438.5	12.6	437.5	66.1	438.9	8.1	100.3
21AR238_196	314.5	26276.446	29.014	17.812	3.3	0.56592	4.3	0.07428	2.3	0.536	461.9	10.3	455.4	15.9	423.5	81.5	461.9	10.3	109.1
21AR238_197	332.5	11453.332	3.014	12.447	1.5	1.82513	2.2	0.16819	1.4	0.657	1002.1	13.2	1054.5	14.2	1165.5	32.3	1165.5	32.3	86.0
21AR238_198	144.7	44374.716	2.607	9.164	1.5	4.48820	2.2	0.30043	1.5	0.691	1693.5	22.6	1728.8	18.2	1772.6	28.9	1772.6	28.9	95.5
21AR238_199	94.3	7845.701	1.887	15.517	8.6	0.51672	10.6	0.06009	2.3	0.215	376.2	8.3	423.0	36.6	687.7	220.7	376.2	8.3	54.7
21AR238_200	264.7	28358.475	2.826	17.370	3.4	0.58943	4.4	0.07521	2.3	0.527	467.5	10.6	470.5	16.8	486.3	83.5	467.5	10.6	96.1
21AR238_201	378.4	15017.016	3.633	17.861	5.1	0.21691	5.9	0.02870	1.8	0.296	182.4	3.2	199.3	10.8	405.4	127.1	182.4	3.2	45.0
21AR238_202	48.0	12866.521	7.151	12.849	6.2	1.92746	6.6	0.18282	1.7	0.262	1082.4	17.3	1090.7	44.3	1108.1	127.8	1108.1	127.8	97.7
21AR238_203	269.0	15353.385	1.657	17.296	3.5	0.55708	4.4	0.07079	1.9	0.435	440.9	8.2	449.6	16.1	495.2	88.0	440.9	8.2	89.0
21AR238_204	167.9	18231.624	2.744	17.247	3.8	0.65628	4.6	0.08355	2.1	0.449	517.3	10.4	512.3	18.7	491.2	91.5	517.3	10.4	105.3
21AR238_205	186.0	21231.340	4.851	13.670	2.7	1.45401	4.3	0.14599	3.2	0.750	878.5	26.3	911.5	25.7	993.4	57.4	993.4	57.4	88.4
21AR238_206	692.3	93558.282	26.312	17.040	1.7	0.69461	2.5	0.08640	1.8	0.715	534.2	9.2	535.6	10.5	542.3	38.5	534.2	9.2	98.5
21AR238_207	469.3	44374.861	4.232	17.306	1.9	0.62644	2.6	0.07915	1.6	0.605	491.1	7.4	493.9	10.2	507.9	45.6	491.1	7.4	96.7
21AR238_208	623.3	662.102	3.237	5.158	39.6	0.01952	46.4	0.00081	14.9	0.322	5.2	0.8	19.6	9.0	2606.7	741.6	5.2	0.8	0.2
21AR238_209	287.2	20833.076	2.784	18.013	3.9	0.45455	4.5	0.06020	1.6	0.352	376.8	5.8	380.5	14.4	403.5	94.8	376.8	5.8	93.4
21AR238_210	136.7	32545.152	2.260	12.959	2.9	1.89938	4.5	0.17970	3.3	0.742	1065.4	32.5	1080.9	29.7	1113.1	59.6	1113.1	59.6	95.7
21AR238_211	45.1	13305.192	5.577	12.661	4.6	2.11192	5.2	0.19678	2.2	0.419	1158.0	23.2	1152.7	36.1	1143.6	94.6	1143.6	94.6	101.3
21AR238_212	359.0	76543.038	10.042	13.530	1.5	1.65145	2.3	0.16215	1.7	0.745	968.7	15.2	990.1	14.4	1038.7	30.7	1038.7	30.7	93.3
21AR238_213	205.9	5848.409	2.314	15.084	7.4	0.16692	8.7	0.01895	2.3	0.268	121.0	2.8	156.7	12.7	738.8	177.9	121.0	2.8	16.4
21AR238_214	246.0	3387.535	5.575	13.331	17.1	0.10312	17.8	0.01056	3.8	0.215	67.7	2.6	99.7	16.9	954.0	356.7	67.7	2.6	7.1
21AR238_215	289.2	11085.677	1.817	17.971	5.0	0.21945	6.9	0.02927	1.8	0.265	186.0	3.4	201.5	12.6	387.7	149.9	186.0	3.4	48.0
21AR238_216	481.1	50679.880	9.733	17.046	1.9	0.66629	2.6	0.08269	1.6	0.626	512.2	7.9	518.5	10.5	547.1	43.9	512.2	7.9	93.6
21AR238_217	91.6	10763.981	3.633	15.622	7.0	0.55664	7.5	0.06436	2.1	0.283	402.1	8.3	449.3	27.4	700.0	154.0	402.1	8.3	57.4
21AR238_218	174.7	9787.988	2.586	16.577	6.9	0.33792	8.1	0.04161	1.6	0.193	262.8	4.0	295.6	20.9	564.1	174.0	262.8	4.0	46.6
21AR238_219	75.8	6172.786	1.140	14.663	11.0	0.65189	12.3	0.07172	3.3	0.272	446.5	14.4	509.6	49.1	804.9	247.0	446.5	14.4	55.5
21AR238_220	45.3	2269.223	2.782	9.941	17.8	0.48663	18.8	0.03732	4.2	0.225	236.2	9.8	402.6	62.5	1520.2	346.4	236.2	9.8	15.5
21AR238_221	37.9	1443.228	1.539	7.501	23.7	0.49361	25.8	0.02880	7.6	0.293	183.0	13.6	407.4	86.7	2019.5	439.4	183.0	13.6	9.1
21AR238_222	74.6	3060.067	2.084	10.770	12.6	0.40459	13.9	0.03312	3.4	0.248	210.1	7.1	345.0	40.7	1396.1	258.4	210.1	7.1	15.0
21AR238_223	566.0	120371.947	4.990	12.347	1.1	2.07544	1.8	0.18596	1.4	0.772	1099.5	14.1	1140.7	12.3	1220.9	22.5	1220.9	22.5	90.1
21AR238_224	271.8	14316.611	1.638	18.357	4.7	0.28572	5.3	0.03873	2.1	0.392	245.0	5.0	255.2	11.9	351.0	110.1	245.0	5.0	69.8
21AR238_225	335.3	53348.883	6.759	13.952	1.7	1.42738	2.9	0.14485	2.3	0.802	872.0	19.0	900.5	17.3	971.8	35.2	971.8	35.2	89.7
21AR238_226	262.0	57438.210	10.973	13.412	1.7	1.70617	2.5	0.16652	1.8	0.727	992.9	16.9	1010.8	16.2	1050.9	35.0	1050.9	35.0	94.5
21AR238_227	173.8	15236.770	1.902	15.162	3.1	0.95003	4.1	0.10619	1.6	0.378	650.6	9.6	678.1	20.3	771.5	80.1	650.6	9.6	84.3
21AR238_228	25.4	1039.477	0.848	8.456	25.6	0.56640	28.1	0.03916	7.3	0.261	247.6	17.8	455.7	103.1	1713.2	500.4	247.6	17.8	14.5
21AR238_229	85.6	5074.791	2.021	15.627	10.1	0.51479	11.2	0.06119	2.4	0.216	382.8	9.0	421.7	38.6	640.9	235.2	382.8	9.0	59.7
21AR238_230	411.3	33553.472	6.719	17.074	1.7	0.67808	2.5	0.08476	1.4	0.558	524.5	7.1	525.6	10.4	531.6	45.9	524.5	7.1	98.7
21AR238_231	222.7	42783.804	6.711	14.464	2.1	1.27091	4.5	0.13423	3.9	0.878	811.9	29.9	832.8	25.3	889.7	44.1	811.9	29.9	91.3
21AR238_232	334.0	1184.269	2.186	6.300	41.9	0.05151	42.9	0.00252	8.5	0.197	16.2	1.4	51.0	21.4	2327.8	730.0	16.2	1.4	0.7

21AR238_233	125.6	2127.148	2.105	8.251	24.9	0.20272	26.4	0.01281	4.4	0.168	82.1	3.6	187.4	45.1	1876.5	470.4	82.1	3.6	4.4
21AR238_234	395.7	31106.671	3.460	18.121	3.2	0.45663	4.0	0.06058	1.8	0.468	379.2	6.8	381.9	12.6	399.5	78.2	379.2	6.8	94.9
21AR238_235	272.3	27057.746	4.985	17.538	3.6	0.56223	4.6	0.07229	2.3	0.509	450.0	10.1	453.0	16.7	469.3	87.1	450.0	10.1	95.9
21AR238_236	311.9	8664.278	6.327	17.015	3.0	0.60350	5.3	0.07675	1.6	0.307	476.7	7.4	479.5	20.1	493.7	110.4	476.7	7.4	96.6
21AR238_237	588.6	9959.720	2.306	18.893	3.1	0.26591	5.2	0.03752	1.5	0.284	237.4	3.4	239.4	11.0	259.9	113.9	237.4	3.4	91.4
21AR238_238	176.5	28635.074	6.463	16.856	3.9	0.78825	4.2	0.09730	1.5	0.349	598.6	8.4	590.2	18.9	559.1	86.3	598.6	8.4	107.1
21AR238_239	286.0	21017.322	4.070	18.199	4.7	0.36193	5.1	0.04844	1.6	0.313	304.9	4.8	313.7	13.8	380.1	109.5	304.9	4.8	80.2
21AR238_240	55.7	2798.734	2.401	11.836	15.0	0.40073	19.0	0.03671	3.5	0.182	232.4	7.9	342.2	55.1	1177.1	369.8	232.4	7.9	19.7
21AR238_241	309.2	2264.161	1.868	16.586	5.2	0.26363	9.0	0.03564	2.1	0.237	225.7	4.7	237.6	19.1	357.4	197.9	225.7	4.7	63.2
21AR238_242	132.2	2000.685	1.539	8.916	20.3	0.17972	21.5	0.01233	4.6	0.214	79.0	3.6	167.8	33.3	1727.0	387.4	79.0	3.6	4.6
21AR238_243	167.6	68696.814	5.712	8.328	1.5	5.20383	2.9	0.31591	2.5	0.859	1769.7	38.4	1853.2	24.6	1949.1	26.4	1949.1	26.4	90.8
21AR238_244	107.8	1482.686	3.308	9.842	24.6	0.13795	28.0	0.01089	8.0	0.288	69.8	5.6	131.2	34.4	1466.1	511.1	69.8	5.6	4.8
21AR238_245	70.3	2260.931	2.092	15.326	9.6	0.51426	17.4	0.06362	3.2	0.185	397.6	12.4	421.3	60.1	554.3	374.0	397.6	12.4	71.7
21AR238_246	226.2	7811.204	2.988	16.885	5.1	0.32205	7.0	0.04085	2.0	0.278	258.1	4.9	283.5	17.4	499.5	148.5	258.1	4.9	51.7
21AR238_247	292.7	15473.134	4.505	18.429	4.2	0.31070	5.3	0.04239	2.2	0.409	267.6	5.7	274.7	12.7	336.7	109.5	267.6	5.7	79.5
21AR238_248	191.5	34227.987	3.573	13.701	2.1	1.55942	4.3	0.15630	3.5	0.828	936.2	30.8	954.2	26.4	997.0	48.6	997.0	48.6	93.9
21AR238_249	28.1	2513.831	17.218	10.125	20.7	1.03575	24.2	0.08080	4.0	0.165	500.9	19.2	721.8	125.0	1488.0	452.9	1488.0	452.9	33.7
21AR238_250	61.6	2786.505	0.812	12.770	16.1	0.36433	19.9	0.03621	3.3	0.164	229.3	7.4	315.4	54.0	1014.2	399.2	229.3	7.4	22.6
21AR238_251	371.6	43003.850	5.420	16.146	2.3	0.83577	3.3	0.09869	2.3	0.697	606.7	13.4	616.8	15.3	654.9	50.9	606.7	13.4	92.6
21AR238_252	38.1	10066.188	3.135	12.041	7.4	2.25251	8.0	0.20037	2.1	0.259	1177.3	22.2	1197.6	55.9	1235.2	150.7	1235.2	150.7	95.3
21AR238_253	219.5	28377.297	540.984	16.377	3.5	0.81030	4.0	0.09698	1.8	0.447	596.7	10.2	602.6	18.2	626.0	77.2	596.7	10.2	95.3
21AR238_254	653.4	57388.679	163.298	17.561	1.9	0.53666	2.4	0.06870	1.3	0.555	428.3	5.5	436.2	8.4	479.0	43.7	428.3	5.5	89.4
21AR238_255	201.3	3374.562	4.345	12.856	2.1	1.57707	3.1	0.15557	1.6	0.522	932.1	14.2	961.2	19.5	1029.3	54.2	1029.3	54.2	90.6
21AR238_256	274.1	29114.813	8.397	17.496	3.5	0.56884	3.9	0.07293	1.4	0.372	453.8	6.3	457.3	14.2	475.9	79.1	453.8	6.3	95.4
21AR238_257	93.2	11589.571	2.562	16.240	8.2	0.69694	8.7	0.08398	1.8	0.213	519.9	9.2	537.0	36.1	611.2	182.8	519.9	9.2	85.1
21AR238_258	303.9	3836.363	4.331	12.885	15.2	0.10451	16.0	0.01028	2.5	0.157	65.9	1.7	100.9	15.4	1034.6	320.1	65.9	1.7	6.4
21AR238_259	179.4	7424.839	2.944	15.941	9.1	0.28027	9.7	0.03352	2.0	0.204	212.6	4.2	250.9	21.6	627.3	205.3	212.6	4.2	33.9
21AR238_260	293.6	16672.300	6.645	17.818	4.4	0.32763	5.4	0.04311	1.9	0.353	272.1	5.0	287.8	13.4	418.1	112.2	272.1	5.0	65.1
21AR238_261	149.3	28736.967	8.049	13.590	2.6	1.72357	3.3	0.17158	2.1	0.619	1020.8	19.4	1017.4	21.3	1010.8	52.8	1010.8	52.8	101.0
21AR238_262	150.1	11466.633	3.923	16.977	4.8	0.55480	5.5	0.06989	2.1	0.382	435.5	8.8	448.1	20.0	514.6	111.8	435.5	8.8	84.6
21AR238_263	200.4	9748.382	3.509	16.335	9.4	0.33212	10.3	0.04046	2.5	0.239	255.7	6.2	291.2	26.0	587.4	216.7	255.7	6.2	43.5
21AR238_264	138.0	12994.281	3.639	16.711	6.2	0.65039	6.5	0.08058	1.8	0.282	499.6	8.9	508.7	26.2	551.0	136.9	499.6	8.9	90.7
21AR238_265	508.2	63503.521	14.716	15.779	1.6	0.86803	3.8	0.10003	3.5	0.903	614.6	20.4	634.5	18.1	707.0	35.0	614.6	20.4	86.9
21AR238_266	89.1	33582.991	1.751	8.400	1.9	4.72757	3.7	0.28997	3.1	0.856	1641.4	45.4	1772.1	30.7	1930.7	33.9	1930.7	33.9	85.0
21AR238_267	423.2	20689.669	4.052	18.915	3.2	0.31347	3.9	0.04374	1.6	0.414	275.9	4.4	276.9	9.5	285.6	81.9	275.9	4.4	96.6
21AR238_268	274.4	42136.253	5.820	14.256	1.9	1.23928	4.4	0.12919	3.9	0.888	783.3	29.0	818.6	24.9	916.6	42.0	783.3	29.0	85.5
21AR238_269	205.8	9849.616	3.635	16.514	8.0	0.33224	8.4	0.04094	2.1	0.251	258.7	5.4	291.3	21.4	562.5	178.2	258.7	5.4	46.0
21AR238_270	842.7	23289.682	21.933	13.723	1.2	1.48047	3.2	0.14915	2.7	0.843	896.2	22.2	922.4	19.1	986.5	34.5	986.5	34.5	90.8
21AR238_271	83.2	27431.382	2.842	10.009	1.5	3.74494	2.2	0.27426	1.4	0.611	1562.4	19.1	1581.0	18.0	1606.8	33.2	1606.8	33.2	97.2
21AR238_272	619.4	21793.235	1.190	18.858	3.0	0.28657	3.7	0.03989	2.1	0.571	252.2	5.2	255.9	8.4	290.8	69.5	252.2	5.2	86.7
21AR238_273	503.7	3534.566	0.904	16.140	3.7	0.58138	14.7	0.07325	3.4	0.227	455.7	14.8	465.3	55.1	514.3	315.9	455.7	14.8	88.6
21AR238_274	540.4	33005.574	1.707	17.251	2.5	0.46396	3.8	0.05874	2.6	0.690	368.0	9.4	387.0	12.2	503.4	60.4	368.0	9.4	73.1
21AR238_275	361.5	21748.895	2.099	17.425	3.1	0.55972	3.8	0.07188	1.6	0.412	447.4	6.8	451.3	13.9	472.2	76.8	447.4	6.8	94.8
21AR238_276	394.4	17739.391	1.700	18.111	3.5	0.29164	4.3	0.03906	1.8	0.425	247.0	4.4	259.9	9.9	378.6	87.9	247.0	4.4	65.2
21AR238_277	274.0	17576.187	2.447	16.782	3.4	0.64126	3.9	0.07948	1.6	0.398	493.0	7.4	503.1	15.5	549.9	78.2	493.0	7.4	89.7
21AR238_278	407.6	14482.294	1.229	17.217	2.8	0.53076	4.1	0.06772	2.1	0.517	422.4	8.6	432.3	14.4	486.3	77.2	422.4	8.6	86.9
21AR238_279	82.9	2169.486	2.146	12.411	12.5	0.26697	20.3	0.02627	4.0	0.198	167.1	6.6	240.3	43.4	1034.6	403.0	167.1	6.6	16.2
21AR238_280	142.3	10045.567	1.647	16.884	6.7	0.48401	8.2	0.06100	1.9	0.236	381.7	7.2	400.8	27.3	513.7	175.9	381.7	7.2	74.3
21AR238_281	331.0	11115.334	0.304	17.576	5.2	0.28732	5.8	0.03764	1.9	0.329	238.2	4.4	256.4	13.1	427.8	121.7	238.2	4.4	55.7
21AR238_282	135.6	11318.075	1.364	16.468	5.2	0.70849	6.7	0.08675	1.8	0.261	536.3	9.0	543.9	28.3	576.5	141.2	536.3	9.0	93.0
21AR238_283	126.4	2861.800	2.769	12.692	15.1	0.23063	15.9	0.02276	3.8	0.237	145.1	5.4	210.7	30.3	1028.1	313.7	145.1	5.4	14.1
21AR238_284	900.3	103686.090	3.213	13.433	1.3	1.66068	2.5	0.16280	2.1	0.847	972.3	19.1	993.6	15.8	1041.9	26.8	1041.9	26.8	93.3
21AR238_285	1335.2	6863.067	1.220	15.850	1.2	0.57101	2.1	0.06821	1.7	0.781	425.3	6.9	458.7	7.9	630.2	29.0	425.3	6.9	67.5
21AR238_286	53.7	2433.933	1.832	11.777	15.6	0.59669	17.3	0.05495	3.0	0.172	344.9	10.0	475.1	65.9	1166.7	339.2	344.9	10.0	29.6
21AR238_287	142.6	5340.843	0.973	15.189	7.9	0.33293	9.1	0.03836	2.4	0.268	242.7	5.8	291.8	23.0	707.3	185.6	242.7	5.8	34.3
21AR238_288	220.3	8730.610	4.022	17.324	7.2	0.31338	7.9	0.04073	2.0	0.254	257.3	5.1	276.8	19.1	445.6	170.0	257.3	5.1	57.7
21AR238_289	473.5	36993.820	5.083	17.495	2.6	0.56040	3.6	0.07191	2.4	0.664	447.7	10.3	451.8	13.1	473.7	59.5	447.7	10.3	94.5
21AR238_290	346.4	10327.033	5.142	14.515	2.9	1.05179	12.2	0.11350	11.2	0.913	693.0	73.3	729.8	63.6	845.3	104.0	693.0	73.3	82.0
21AR238_291	345.2	8902.217	3.089	17.473	5.7	0.20712	7.1	0.02714	1.7	0.248	172.6	3.0	191.1	12.3	427.2	152.4	172.6	3.0	40.4
21AR238_292	345.7	8185.031	1.979	18.168	5.1	0.19261	6.0	0.02631	2.1	0.345	167.4	3.4	178.9	9.8	333.8	126.7	167.4	3.4	50.2
21AR238_293	449.5	83552.663	4.647	12.801	1.5	1.95606	2.4	0.18243	1.9	0.778	1080.2	18.9	1100.5	16.4	1141.8	30.5	1141.8	30.5	94.6
21AR238_294	161.2	10359.292	0.751	15.824	4.2	0.83755	4.7	0.09852	1.7	0.360	605.7	9.8	617.8	21.7	663.1	93.9	605.7	9.8	91.4
21AR238_295	248.1	8020.454	0.989	16.426	7.0	0.31079	8.8	0.03825	2.0	0.226	242.0	4.7	274.8	21.2	565.3	187.2	242.0	4.7	42.8
21AR238_296	182.7	4935.578	1.507	14.899	8.4	0.23473	10.3	0.02660	2.6	0.253	169.2	4.3	214.1	19.8	742.6	210.1	169.2	4.3	22.8
21AR238_297	776.8	4901.959	2.994	13.578	2.0	0.56929	5.2	0.05862	4.7	0.903	367.2	16.7	457.6	19.1	942.1	45.7	367.2	16.7	39.0
21AR238_298	246.7	18967.910	1.730	16.364	3.0	0.70631	3.7	0.08524	1.6	0.426	527.3	7.9	542.6	15.5	608.0	72.1	527.3	7.9	86.7
21AR238_299	648.5	48274.370	1.859	17.783	1.9	0.54639	2.6	0.07116	1.7	0.655	443.2	7.3	442.6	9.3	440.8	43.7	443.2	7.3	100.5
21AR238_300	215.2	14586.215	3.097	16.226	2.8	0.78389	3.7	0.09421	1.6	0.423	580.4	8.6	587.7	16.4	617.0	72.0	580.4	8.6	94.1
21AR238_301	340.6	2356.362	1.390	15.971	4.0	0.48470	8.1	0.06259	2.1	0.263	391.3	8.1	401.3	27.0	459.9	174.2	391.3	8.1	85.1
21AR238_302	225.7	652.474	0.942	5.385	31.9	0.06646	35.7	0.00291	9.4	0.263	18.7	1.8	65.3	22.6	2515.2	583.9	18.7	1.8	0.7
21AR238_303	72.8	3314.712	1.631	14.538	10.8	0.43243	13.4	0.04896	2.7	0.202	308.1	8.1	364.9	41.1	744.4	277.7	308.1	8.1	41.4
21AR238_304	382.8	20110.247	3.459	15.414	2.1	0.97257	4.9	0.11032	4.2	0.864	674.6	27.2	689.8	24.6	740.6	52.2	674.6	27.2	91.1
21AR238_305	91.8	6654.125	2.383	15.394	6.4	0.73375	7.0	0.08504	1.9	0.266	526.2	9.5	558.8	30.2	694.8	144.5	526.2	9.5	75.7
21AR238_306	377.9	3958.041	1.237	16.028	10.2	0.09760	11.1	0.01210	3.3	0.297	77.5	2.5	94.6	10.0	549.9	230.9	77.5	2.5	14.1
21AR238_307	462.3	20837.765	0.930	16.147	2.2	0.79936	2.9	0.09500	1.8	0.610	585.0	10.0	596.5	13.2	641.2	50.1	585.0	10.0	91.2
21AR238_308	225.8	34826.569	5.134	13.283	1.8	1.68425	3.0	0.16370	2.3	0.751	977.3	20.4	1002.6	19.1	1059.2	40.0	1059.2	40.0	92.3
21AR238_309	238.9	550.369	2.671	12.262	9.7	0.29197	89.9	0.03861	2.7	0.030	244.2	6.4	260.1	206.9	406.6		244.2	6.4	60.1
21AR238_310	105.9	14561.508	3.513	13.018	4.6	1.69373	6.0	0.16253	3.5	0.591	970.8	32.0	1006.2	38.3	1084.9	97.1	1084.9	97.1	89.5
21AR238_311	243.1	17012.858	2.024	16.943	3.8	0.55865	4.6	0.06990	1.8	0.404	435.5	7.7	450.6	16.6	529.5	91.3	435.5	7.7	82.2
21AR238_312	1110.3	1659.763	7.735	10.084	14.2	0.02176	16.6	0.00174	4.5	0.270	11.2	0.5	21.9	3.6	1435.7	305.5	11.2	0.5	0.8
21AR238_313	119.2	2200.223	3.373	14.628	7.0	0.69421	14.5	0.08196	4.9	0.337	507.8	23.8	535.3	60.3	655.2	293.2	507.8	23.8	77.5
21AR238_314	659.9	11397.552	1.651	18.238	2.7	0.30956	3.5	0.04215	2.0	0.559	266.1	5.1	273.8	8.5	341.2	66.3	266.1	5.1	78.0
21AR238_315	254.4	72321.601	1.479	9.088	1.4	4.47640	2.3	0.29711	1.8	0.783	1676.9	27.0	1726.6	19.4	1788.1	26.5	1788.1	26.5	93.8
21AR238_316	266.1	13937.818	3.914	13.611	2.1	1.53509	4.3	0.15448	3.6	0.841	926.0	31.3	944.5	26.5	988.8	47.4	988.8	47.4	93.7
21AR238_317	379.2	13299.535	0.959	18.617	3.6	0.27407	4.5	0.03798	1.9	0.421	240.3	4.5	245.9	9.9	301.2	93.7	240.3	4.5	79.8
21AR238_318	661.2	4116.095	1.546	18.218	3.7	0.28280	13.6	0.04015	3.2	0.232	253.8	7.9	252.9	30.5	245.7	305.4	253.8	7.9	103.3
21AR238_319	259.9	511.416	0.534	12.572	9.4	0.23659	89.8	0.03381	2.3	0.025	214.4	4.8	215.6	174.9	230.4		214.4	4.8	93.1
21AR238_320	184.2	11568.996	5.837	17.020	4.1	0.63019	5.5	0.07964	1.9	0.340	494.0	9.0	496.2	21.7	507.5	114.6	494.0	9.0	97.3
21AR238_321	100.1	27783.776	1.675	9.424	1.9	4.17634	2.5	0.28733	1.5	0.591	1628.2	21.4	1669.4	20.6	1722.4	37.3	1722.4	37.3	94.5
21AR238_322	109.8	6276.515	0.615	15.918	6.6	0.54813	7.8	0.06582	3.2	0.408	410.9	12.7	443.8	28.1	618.9	153.9	410.9	12.7	66.4
21AR238_323	808.9	8491.190	1.758	17.967	5.6	0.09111	6.2	0.01225	2.0	0.321	78.5	1.6	88.5	5.3	369.0	133.3	78.5	1.6	21.3
21AR238_324	198.9	11902.160	2.003	17.200	4.6	0.46072	5.7	0.05884	2.0	0.349	368.6	7.2	384.8	18.3	484.4	118.4	368.6	7.2	76.1
21AR238_325	67.9	9668.711	2.605	13.509	5.1	1.60921	6.0	0.16124	1.9	0.311	963.6	16.7	973.8	37.6	997.7	115.9	997.7	115.9	96.6
21AR238_326	119.0	1313.696	2.745	7.523	29.1	0.19361	31.4	0.01150	7.1	0.226	73.7	5.2	179.7	51.7	1988.1	547.5	73.7	5.2	3.7
21AR238_327	117.0	7221.511	1.992	15.922	6.4	0.67096	8.5	0.08021	2.2	0.255	497.4	10.4	521.3	34.9	628.3	178.2	497.4	10.4	79.2
21AR238_328	539.2	1280.704	2.036	17.201	5.9	0.24692	43.1	0.03850	1.5	0.034	243.5	3.5	224.1	86.6	25.3		243.5	3.5	963.8
21AR238_329	109.0	2239.217	36.277	12.958	3.7	1.55249	12.4	0.15940	2.2	0.177	953.4	19.4	951.5	76.3	947.8	249.1	947.8	249.1	100.6
21AR238_330	173.6	6365.419	2.252	15.054	12.2	0.31890	12.6	0.03613	2.2	0.177	228.8	5.0	281.1	31.0	743.0	263.1	228.8	5.0	30.8
21AR238_331	158.0	39613.517	1.071	8.777	1.7	4.48128	4.9	0.28617	4.6	0.939	1622.4	66.5	1727.5	41.0	1858.1	30.6	1858.1	30.6	87.3
21AR238_332	251.1	14564.109	2.644	17.508	4.9	0.43937	6.3	0.05678	2.7	0.431	356.0	9.4	369.8	19.5	458.1	125.8	356.0	9.4	77.7
21AR238_333	199.9	47730.857	2.216	11.083	1.8	2.85718	2.3	0.23038	1.5	0.643	1336.5	17.9	1370.7	17.4	1425.2	33.8	1425.2	33.8	93.8
21AR238_334	362.2	8656.395	2.922	17.143	7.1	0.20947	8.1	0.02682	1.5	0.189	170.6	2.6	193.1	14.3	479.0	176.6	170.6	2.6	35.6
21AR238_335	70.1	2442.434	1.068	10.863	17.2	0.45741	18.7	0.03843	2.8	0.148	243.1	6.6	382.5	59.7	1346.1	358.3	243.1	6.6	18.1
21AR238_336	281.3	7378.639	0.811	15.628	3.1	0.81693	5.6	0.09565	1.7	0.306	588.9	9.7	606.3	25.7	673.1	114.6	588.9	9.7	87.5
21AR238_337	124.9	3834.123	0.697	16.107	8.8	0.29209	9.6	0.03636	2.3	0.236	230.2	5.1	260.2	22.0	540.6	203.7	230.2	5.1	42.6
21AR238_338	201.0	18242.364	5.544	15.844	4.2	0.91433	7.6	0.10646	6.3	0.826	652.1	39.1	659.4	37.0	685.1	91.8	652.1	39.1	95.2
21AR238_339	543.9	3582.356	1.840	15.570	2.4	0.78626	11.6	0.09480	1.9	0.161	583.9	10.4	589.1	51.7	610.0	247.0	583.9	10.4	95.7
21AR238_340	274.2	17112.927	2.401	17.373	3.4	0.55707	4.6	0.07128	1.7	0.367	443.9	7.3	449.6	16.8	480.1	95.4	443.9	7.3	92.4
21AR238_341	173.0	484.044	1.263	12.548	12.0	0.25531	46.9	0.03765	2.1	0.044	238.2	4.8	230.9	96.9	157.8		238.2	4.8	151.0
21AR238_342	224.9	8974.743	2.094	17.424	4.3	0.54626	5.3	0.07086	2.4	0.456	441.3	10.3	442.5	19.0	449.8	104.7	441.3	10.3	98.1
21AR238_343	300.8	18169.758	3.876	17.899	3.4	0.44000	4.4	0.05780	2.5	0.563	362.2	8.7	370.3	13.6	421.7	81.1	362.2	8.7	85.9
21AR238_344	278.8	8364.517	3.488	16.783	6.0	0.22144	7.3	0.02758	2.4	0.330	175.4	4.2	203.1	13.5	539.7	151.6	175.4	4.2	32.5
21AR238_345	268.1	19526.646	65.658	17.508	3.4	0.53918	4.1	0.06919	1.8	0.453	431.3	7.7	437.9	14.5	473.8	80.2	431.3	7.7	91.0
21AR238_346	396.1	17843.858	2.189	13.636	1.7	1.54989	3.0	0.15455	1.9	0.631	926.4	16.3	950.4	18.4	1007.3	46.9	1007.3	46.9	92.0
21AR238_347	86.7	3783.586	1.268	15.225	8.6	0.48203	9.7	0.05637	2.2	0.231	353.5	7.7	399.5	31.9	675.9	201.1	353.5	7.7	52.3
21AR238_348	59.2	18091.482	0.881	8.649	2.3	4.99713	3.0	0.31630	1.7	0.552	1771.7	25.6	1818.8	25.4	1874.1	45.1	1874.1	45.1	94.5

21AR238_349	284.2	18736.632	5.484	17.356	4.0	0.57972	5.7	0.07411	3.8	0.668	460.9	16.9	464.3	21.2	482.2	93.5	460.9	16.9	95.6
21AR238_350	133.2	3356.267	2.153	14.734	11.2	0.31622	13.4	0.03615	1.8	0.136	228.9	4.1	279.0	32.7	724.1	282.3	228.9	4.1	31.6
21AR238_351	201.0	7121.588	2.192	16.748	5.2	0.47568	7.5	0.06001	2.2	0.300	375.7	8.2	395.1	24.5	511.3	157.1	375.7	8.2	73.5
21AR238_352	544.8	37576.319	21.138	14.113	2.0	1.37790	4.9	0.14139	4.5	0.914	852.5	35.8	879.5	28.9	949.0	40.6	949.0	40.6	89.8
21AR238_353	478.1	1831.059	1.571	16.462	6.7	0.30737	28.3	0.04214	2.0	0.070	266.1	5.1	272.1	67.5	325.6	643.3	266.1	5.1	81.7
21AR238_354	38.1	6141.916	3.225	12.998	9.6	1.65270	10.3	0.16018	1.8	0.179	957.8	16.4	990.6	65.1	1064.9	203.8	1064.9	203.8	89.9
21AR238_355	153.4	27405.269	4.422	13.176	2.7	1.80426	3.5	0.17311	2.1	0.594	1029.2	20.0	1047.0	23.1	1085.2	57.0	1085.2	57.0	94.8
21AR238_356	196.1	24949.943	1.889	15.174	2.4	1.10454	3.1	0.12176	1.6	0.505	740.7	11.1	755.5	16.7	800.7	56.7	740.7	11.1	92.5
21AR238_357	374.6	20027.871	2.481	17.573	2.9	0.54812	3.8	0.07047	1.5	0.393	439.0	6.4	443.8	13.8	469.5	78.4	439.0	6.4	93.5
21AR238_358	196.6	4846.754	1.670	15.787	10.5	0.22064	11.4	0.02641	2.1	0.182	168.0	3.4	202.4	20.9	626.1	241.9	168.0	3.4	26.8
21AR238_359	63.9	3733.479	1.693	14.538	9.2	0.64481	11.0	0.07173	2.3	0.205	446.6	9.8	505.3	44.0	781.6	227.3	446.6	9.8	57.1
21AR238_360	174.7	6219.233	1.824	14.436	10.2	0.27551	12.2	0.02972	2.7	0.223	188.8	5.1	247.1	26.7	846.0	247.1	188.8	5.1	22.3
21AR238_361	56.4	8763.703	1.763	12.579	4.6	1.90671	5.5	0.17689	1.5	0.280	1050.0	14.9	1083.4	36.6	1152.2	104.7	1152.2	104.7	91.1
21AR238_362	462.2	30530.795	238.391	17.085	1.7	0.63074	2.7	0.07879	1.8	0.667	488.9	8.6	496.6	10.7	533.0	44.6	488.9	8.6	91.7
21AR238_363	341.9	19730.109	20.643	17.204	3.5	0.57648	6.2	0.07284	4.8	0.779	453.3	21.2	462.2	23.0	507.8	85.6	453.3	21.2	89.3
21AR238_364	197.6	7168.175	1.427	15.418	9.3	0.27029	10.3	0.03119	2.6	0.252	198.0	5.1	242.9	22.4	704.0	213.3	198.0	5.1	28.1
21AR238_365	303.9	69139.293	1.760	11.319	1.9	2.73230	2.7	0.22480	2.0	0.723	1307.2	23.3	1337.3	20.3	1386.7	36.2	1386.7	36.2	94.3
21AR238_366	329.0	11452.147	1.048	17.373	5.6	0.29251	6.1	0.03769	1.9	0.318	238.5	4.5	260.5	14.0	465.0	128.0	238.5	4.5	51.3
21AR238_367	305.1	11083.563	1.586	17.612	5.8	0.32019	7.4	0.04183	1.5	0.197	264.2	3.8	282.0	18.1	434.1	160.8	264.2	3.8	60.8
21AR238_368	21.9	652.037	1.468	5.330	34.2	0.89780	40.8	0.03866	8.0	0.195	244.5	19.1	650.6	196.6	2542.9	678.3	244.5	19.1	9.6
21AR238_369	789.4	34967.379	2.317	17.613	1.7	0.54757	2.2	0.07046	1.4	0.621	438.9	5.8	443.4	7.9	467.8	38.2	438.9	5.8	93.8
21AR238_370	287.6	4277.401	1.701	15.634	8.8	0.13353	13.2	0.01599	2.4	0.180	102.2	2.4	127.3	15.8	625.1	279.8	102.2	2.4	16.4
21AR238_371	313.3	13199.422	3.697	17.894	3.9	0.46042	4.7	0.06093	1.5	0.323	381.3	5.6	384.6	15.0	405.1	99.2	381.3	5.6	94.1
21AR238_372	264.5	4486.551	2.903	16.630	3.5	0.62330	7.1	0.07956	1.5	0.219	493.5	7.3	491.9	27.5	485.6	152.1	493.5	7.3	101.6
21AR238_373	174.0	12843.521	1.629	17.128	5.1	0.56767	5.9	0.07200	1.7	0.288	448.2	7.4	456.5	21.7	499.6	124.6	448.2	7.4	89.7
21AR238_374	97.5	1635.839	2.155	8.208	30.8	0.26383	32.8	0.01687	5.0	0.152	107.8	5.3	237.8	69.6	1855.8	590.1	107.8	5.3	5.8
21AR238_375	401.7	10632.348	0.819	17.820	3.5	0.45479	4.7	0.06032	1.7	0.364	377.6	6.2	380.6	14.8	400.1	97.5	377.6	6.2	94.4
21AR238_376	198.2	30763.197	4.765	13.582	1.9	1.68496	2.8	0.16755	1.8	0.659	998.6	16.9	1002.9	17.7	1013.1	42.4	1013.1	42.4	98.6
21AR238_377	207.9	1789.350	2.342	10.992	20.2	0.10125	24.5	0.00886	4.9	0.198	56.9	2.7	97.9	22.8	1267.0	469.7	56.9	2.7	4.5
21AR238_378	74.0	2562.090	1.851	11.166	16.1	0.44346	17.6	0.03836	2.6	0.150	242.7	6.3	372.7	55.1	1289.7	340.1	242.7	6.3	18.8
21AR238_379	656.3	95808.104	2.756	14.294	1.5	1.30047	2.2	0.13554	1.7	0.752	819.4	13.0	845.9	12.9	917.2	30.5	819.4	13.0	89.3
21AR238_380	117.8	18432.061	10.419	12.747	3.3	1.79989	3.9	0.16874	1.9	0.477	1005.2	17.4	1045.4	25.5	1131.4	68.5	1131.4	68.5	88.8
21AR238_381	172.1	1640.201	4.716	14.742	6.7	0.41534	12.1	0.05128	5.5	0.459	322.4	17.4	352.7	36.0	558.5	234.0	322.4	17.4	57.7
21AR238_382	971.1	3379.793	2.015	18.242	2.5	0.27591	3.5	0.03981	1.5	0.417	251.6	3.6	247.4	7.7	208.4	74.1	251.6	3.6	120.7
21AR238_383	365.4	21948.261	1.241	17.834	3.5	0.46760	4.2	0.06153	1.7	0.398	384.9	6.3	389.5	13.6	418.0	86.2	384.9	6.3	92.1
21AR238_384	555.4	7259.820	17.795	14.382	1.6	1.20723	3.2	0.13042	2.0	0.618	790.3	14.7	803.9	17.8	842.9	52.5	790.3	14.7	93.8
21AR238_385	330.3	12001.796	0.792	18.242	4.2	0.28109	4.8	0.03827	1.6	0.325	242.1	3.7	251.5	10.6	341.1	102.1	242.1	3.7	71.0
21AR238_386	59.8	3405.446	0.531	13.471	7.9	0.57817	10.9	0.06025	2.6	0.238	377.2	9.5	463.3	40.6	917.4	217.9	377.2	9.5	41.1
21AR238_387	253.5	2817.964	0.914	11.955	16.1	0.10199	17.0	0.00947	3.1	0.182	60.7	1.9	98.6	16.0	1150.9	333.2	60.7	1.9	5.3
21AR238_388	787.7	871.256	0.937	7.418	17.2	0.02166	21.9	0.00132	9.9	0.452	8.5	0.8	21.8	4.7	1938.4	350.3	8.5	0.8	0.4
21AR238_389	415.9	37646.839	1.168	15.971	1.8	0.90018	2.3	0.10550	1.4	0.620	646.5	8.9	651.8	11.2	671.1	39.2	646.5	8.9	96.3
21AR238_390	20.7	760.648	0.744	5.194	38.8	0.92124	42.3	0.03811	7.7	0.182	241.1	18.2	663.0	206.4	2609.7	700.3	241.1	18.2	9.2
21AR238_391	500.9	10486.345	1.238	17.678	5.7	0.17903	6.2	0.02366	1.8	0.289	150.8	2.7	167.2	9.6	407.9	133.6	150.8	2.7	37.0
21AR238_392	656.2	2322.552	1.189	17.274	3.1	0.22887	12.5	0.03236	4.1	0.330	205.3	8.4	209.3	23.7	254.9	272.6	205.3	8.4	80.5
21AR238_393	628.4	22543.453	2.170	18.663	3.5	0.27857	4.0	0.03837	1.9	0.461	242.7	4.4	249.5	8.9	314.9	81.1	242.7	4.4	77.1
21AR238_394	188.8	9031.500	0.407	16.116	6.2	0.32172	6.9	0.03879	2.4	0.340	245.3	5.7	283.2	17.2	610.2	141.2	245.3	5.7	40.2
21AR238_395	122.9	9021.354	1.163	16.199	5.4	0.67357	7.0	0.08164	2.1	0.300	505.9	10.3	522.9	28.7	598.7	145.1	505.9	10.3	84.5
21AR238_396	328.1	16864.707	2.170	17.246	3.2	0.57373	4.6	0.07319	2.6	0.562	455.4	11.4	460.4	17.0	486.7	83.9	455.4	11.4	93.6
21AR238_397	57.1	4294.930	68.043	13.794	11.7	0.78548	14.3	0.08276	7.4	0.516	512.6	36.4	588.6	63.9	894.6	252.9	512.6	36.4	57.3
21AR238_398	163.3	28917.036	2.782	13.454	2.2	1.71426	3.0	0.16941	2.0	0.654	1008.9	18.5	1013.9	19.4	1025.5	46.4	1025.5	46.4	98.4
21AR238_399	217.8	3821.134	1.484	15.900	9.4	0.22452	15.1	0.02773	2.5	0.164	176.3	4.3	205.7	28.2	558.1	326.1	176.3	4.3	31.6
21AR238_400	169.4	3603.003	1.359	14.299	10.4	0.22669	11.8	0.02509	2.4	0.206	159.7	3.8	207.5	22.2	792.6	242.7	159.7	3.8	20.2
21AR238_401	51.2	1333.253	0.768	7.404	29.9	0.43589	31.0	0.02543	5.8	0.186	161.9	9.2	367.4	95.6	2019.9	543.1	161.9	9.2	8.0
21AR238_402	195.0	10040.214	1.643	17.366	6.0	0.36081	7.3	0.04680	2.1	0.293	294.9	6.1	312.8	19.6	449.9	154.8	294.9	6.1	65.5
21AR238_403	157.4	21255.494	2.581	12.935	2.2	1.70497	3.0	0.16198	1.9	0.627	967.7	17.2	1010.4	19.5	1104.9	47.5	1104.9	47.5	87.6
21AR238_404	113.4	8263.394	3.585	16.916	5.1	0.55414	6.0	0.07034	2.1	0.350	438.2	8.8	447.7	21.6	497.6	123.0	438.2	8.8	88.1
21AR238_405	659.6	18983.845	194.576	15.345	1.4	1.03937	2.8	0.11750	1.6	0.591	716.2	11.1	723.6	14.4	747.6	47.4	716.2	11.1	95.8
21AR238_406	264.8	10736.787	1.664	18.232	5.0	0.31473	5.7	0.04285	2.0	0.355	270.5	5.3	277.8	13.8	341.5	119.8	270.5	5.3	79.2
21AR238_407	380.5	15754.228	2.283	18.615	3.1	0.30146	3.9	0.04158	1.4	0.349	262.6	3.5	267.5	9.3	311.9	84.1	262.6	3.5	84.2
21AR238_408	171.6	25605.246	3.060	14.223	2.7	1.40698	3.4	0.14689	1.8	0.536	883.5	15.1	891.9	20.3	913.6	59.3	883.5	15.1	96.7
21AR238_409	134.2	7640.044	1.036	16.782	6.9	0.49137	9.2	0.06202	2.1	0.232	387.9	8.1	405.8	30.8	510.2	197.0	387.9	8.1	76.0
21AR238_410	218.7	11979.454	1.714	17.349	6.5	0.32327	7.1	0.04172	2.3	0.320	263.5	5.9	284.4	17.7	461.3	149.5	263.5	5.9	57.1
21AR238_411	367.1	72822.508	7.224	7.882	1.3	5.82987	2.1	0.33505	1.6	0.793	1862.8	26.5	1950.9	17.9	2046.4	22.3	2046.4	22.3	91.0
21AR238_412	322.5	1343.750	3.217	15.298	6.1	0.53355	34.6	0.07127	3.2	0.092	443.8	13.7	434.2	122.2	384.4	778.1	443.8	13.7	115.4
21AR238_413	138.7	10820.409	3.070	16.340	6.0	0.66693	7.6	0.08113	2.3	0.298	502.8	10.9	518.8	30.8	590.9	156.9	502.8	10.9	85.1
21AR238_414	95.5	3759.661	1.734	12.553	12.6	0.41829	13.6	0.03997	2.8	0.206	252.6	6.9	354.8	40.8	1093.4	267.0	252.6	6.9	23.1
21AR238_415	72.6	3850.087	3.848	13.711	11.7	0.51279	14.7	0.05360	2.8	0.190	336.6	9.2	420.3	50.7	910.9	297.9	336.6	9.2	37.0
21AR238_416	217.4	38556.603	1.299	11.190	1.7	2.91887	2.8	0.23729	2.2	0.777	1372.6	26.8	1386.8	21.1	1409.6	33.6	1409.6	33.6	97.4
21AR238_417	314.6	48425.951	1.750	11.431	1.3	2.67557	2.5	0.22202	2.0	0.830	1292.5	23.8	1321.7	18.1	1370.2	26.3	1370.2	26.3	94.3
21AR238_418	276.8	27795.260	1.092	17.545	3.5	0.57611	4.0	0.07381	1.6	0.410	459.1	7.2	462.0	14.7	477.2	80.0	459.1	7.2	96.2
21AR238_419	236.5	15560.084	1.169	17.020	3.2	0.62125	3.9	0.07774	1.6	0.419	482.6	7.7	490.6	15.3	529.2	78.2	482.6	7.7	91.2
21AR238_420	133.0	5564.956	1.281	15.346	9.0	0.32959	10.0	0.03823	2.4	0.243	241.9	5.8	289.3	25.2	693.1	207.0	241.9	5.8	34.9
21AR238_421	176.6	17306.226	17.794	15.613	3.6	0.86354	7.0	0.09913	5.7	0.811	609.3	32.9	632.1	32.8	715.1	86.6	609.3	32.9	85.2
21AR238_422	257.5	8653.934	1.423	16.968	5.6	0.28181	7.7	0.03572	2.1	0.276	226.3	4.7	252.1	17.2	500.9	163.3	226.3	4.7	45.2
21AR238_423	119.3	30872.502	2.839	10.816	1.5	3.04685	2.6	0.24034	2.0	0.773	1388.5	24.7	1419.4	19.6	1467.0	30.8	1467.0	30.8	94.6
21AR238_424	130.7	4221.560	0.801	15.597	10.5	0.27170	12.4	0.03244	2.7	0.216	205.8	5.4	244.1	26.8	631.1	260.5	205.8	5.4	32.6
21AR238_425	205.0	621.732	1.012	5.696	39.1	0.06149	44.4	0.00287	17.2	0.387	18.5	3.2	60.6	26.1	2405.3	703.8	18.5	3.2	0.8
21AR238_426	228.4	25192.184	5.952	16.508	3.9	0.72531	4.3	0.08756	1.7	0.393	541.1	8.8	553.8	18.4	607.4	85.6	541.1	8.8	89.1
21AR238_427	134.3	16409.837	2.868	15.653	4.5	0.82202	5.1	0.09450	2.1	0.414	582.1	11.8	609.2	23.5	712.2	99.1	582.1	11.8	81.7
21AR238_428	69.3	2337.764	2.361	10.614	20.8	0.45083	21.6	0.03686	3.2	0.148	233.3	7.3	377.9	68.2	1398.6	410.6	233.3	7.3	16.7
21AR238_429	341.3	16128.460	0.847	18.331	4.1	0.28702	4.6	0.03864	2.0	0.425	244.4	4.7	256.2	10.5	367.0	94.5	244.4	4.7	66.6
21AR238_430	354.1	24594.446	3.272	17.432	2.9	0.57208	3.3	0.07276	1.5	0.440	452.8	6.4	459.4	12.2	493.4	65.5	452.8	6.4	91.8
21AR238_431	671.1	34514.062	1.495	16.277	1.6	0.78817	2.7	0.09348	2.1	0.764	576.1	11.3	590.1	12.1	645.5	37.4	576.1	11.3	89.2
21AR238_432	232.5	60394.864	1.451	8.839	1.5	4.58417	3.6	0.29354	3.2	0.900	1659.2	47.3	1746.4	30.0	1853.2	28.4	1853.2	28.4	89.5
21AR238_433	336.6	10545.515	2.191	18.120	5.0	0.27822	6.2	0.03739	2.0	0.316	236.6	4.6	249.2	13.8	370.6	133.0	236.6	4.6	63.9
21AR238_434	99.3	9425.728	1.450	15.581	5.9	0.71658	6.3	0.08270	1.8	0.285	512.2	8.8	548.6	26.7	703.8	128.4	512.2	8.8	72.8
21AR238_435	154.1	2181.450	1.053	14.262	9.2	0.28360	11.0	0.03230	4.0	0.367	204.9	8.1	253.5	24.6	732.2	216.6	204.9	8.1	28.0
21AR238_436	421.1	23019.097	1.169	18.222	2.6	0.43244	3.4	0.05769	2.0	0.581	361.6	6.9	364.9	10.4	387.1	62.1	361.6	6.9	93.4
21AR238_437	699.9	2075.599	1.632	17.283	2.4	0.28035	3.8	0.04002	2.1	0.547	253.0	5.1	250.9	8.4	232.8	72.7	253.0	5.1	108.7
21AR238_438	358.7	15953.489	1.701	17.506	5.2	0.28755	6.0	0.03709	2.3	0.392	234.8	5.4	256.6	13.6	462.1	122.2	234.8	5.4	50.8
21AR238_439	107.5	8862.706	4.301	15.899	5.4	0.82755	6.7	0.09794	1.8	0.274	602.4	10.5	612.2	30.8	650.0	138.2	602.4	10.5	92.7
21AR238_440	128.2	14562.124	1.031	11.172	2.0	2.67396	3.3	0.21897	1.9	0.555	1276.4	21.5	1321.3	24.7	1395.6	53.4	1395.6	53.4	91.5
21AR238_441	111.3	7868.333	1.393	13.342	3.8	1.54695	6.4	0.15359	3.2	0.498	921.1	27.4	949.3	39.5	1016.2	112.6	1016.2	112.6	90.6
21AR238_442	170.7	4266.755	1.635	15.682	10.8	0.22140	14.1	0.02659	2.4	0.171	169.2	4.0	203.1	26.0	618.2	300.7	169.2	4.0	27.4
21AR238_443	187.4	4153.153	0.483	16.010	9.5	0.22420	10.3	0.02757	2.3	0.218	175.3	3.9	205.4	19.2	567.1	219.8	175.3	3.9	30.9
21AR238_444	155.1	2482.499	1.208	13.453	4.0	1.25387	16.2	0.13260	4.7	0.291	802.7	35.6	825.1	91.5	887.0	320.4	802.7	35.6	90.5
21AR238_445	230.1	8441.937	1.198	17.400	6.5	0.30613	8.4	0.03982	2.4	0.290	251.7	6.0	271.2	20.0	443.6	178.6	251.7	6.0	56.8
21AR238_446	29.0	2066.039	1.342	13.210	12.8	0.67822	18.4	0.07152	3.7	0.199	445.3	15.7	525.7	75.6	893.1	373.1	445.3	15.7	49.9
21AR238_447	205.9	14054.722	9.391	17.372	4.0	0.65447	5.1	0.08402	1.8	0.358	520.1	9.1	511.2	20.5	472.9	105.5	520.1	9.1	110.0
21AR238_448	42.8	8834.542	0.688	13.376	6.6	1.78542	7.6	0.17681	1.9	0.250	1049.5	18.5	1040.2	49.7	1021.4	149.7	1021.4	149.7	102.8
21AR238_449	170.9	2455.315	1.229	11.680	15.1	0.17460	18.0	0.01584	2.8	0.155	101.3	2.8	163.4	27.2	1196.3	351.6	101.3	2.8	8.5
21AR238_450	564.3	33942.917	1.538	18.224	2.6	0.45158	3.6	0.06009	2.0	0.570	376.2	7.5	378.4	11.3	393.0	66.2	376.2	7.5	95.7
21AR238_451	133.1	6758.587	0.498	16.763	6.3	0.50987	9.2	0.06423	2.4	0.263	401.3	9.4	418.4	31.5	514.4	195.0	401.3	9.4	78.0
21AR238_452	172.9	1697.400	3.650	12.696	3.7	1.54518	23.8	0.15922	2.5	0.103	952.5	21.8	948.6	146.7	940.5	486.0	940.5	486.0	101.3
21AR238_453	313.6	19913.524	5.298	17.234	3.7	0.55561	4.6	0.07027	2.0	0.439	437.8	8.6	448.7	16.8	505.9	91.7	437.8	8.6	86.5
21AR238_454	252.5	18305.705	3.284	16.886	5.3	0.52540	5.8	0.06516	2.0	0.345	406.9	7.9	428.7	20.4	548.8	119.4	406.9	7.9	74.1
21AR238_455	70.3	1248.874	2.252	9.814	16.4	0.23354	19.7	0.01865	5.1	0.257	119.1	6.0	213.1	37.9	1443.4	363.4	119.1	6.0	8.3
21AR238_456	190.3	14165.331	1.627	12.734	2.0	1.93803	2.6	0.18114	1.5	0.589	1073.2	15.2	1094.3	17.5	1137.5	41.9	1137.5	41.9	94.3
21AR238_457	211.0	36868.987	3.136	13.296	1.9	1.72247	2.7	0.16711	1.7	0.645	996.2	16.0	1016.9	17.3	1062.8	41.4	1062.8	41.4	93.7
21AR238_458	191.1	13524.358	1.689	16.970	3.9	0.57689	5.1	0.07226	2.0	0.388	449.7	8.6	462.5	18.9	527.1	102.9	449.7	8.6	85.3
21AR238_459	151.5	2046.053	2.384	7.909	40.9	0.16549	42.4	0.01000	5.3	0.125	64.2	3.4	155.5	61.1	1956.9	759.1	64.2	3.4	3.3
21AR238_460	357.7	13965.791	1.215	18.503	5.4	0.26166	6.7	0.03565	3.5	0.520	225.8	7.8	236.0	14.2	339.8	130.2	225.8	7.8	66.4
21AR238_461	540.1	15712.232	1.846	17.620	2.6	0.54877	4.2	0.07098	1.9	0.449	442.0	8.1	444.2	15.2	456.4	83.5	442.0	8.1	96.9
21AR238_462	884.4	72829.776	25.602	15.669	1.7	0.92393	3.1	0.10485	2.6	0.835	642.8	15.9	664.4	15.2	739.5	36.2	642.8	15.9	86.9
21AR238_463	129.6	6343.626	1.178	15.964	6.9	0.50075	7.6	0.05982	2.4	0.313	374.5	8.7	412.2	25.8	629.9	155.7	374.5	8.7	59.5
21AR238_464	236.5	6698.389	1.154	16.859	5.5	0.43837	7.8	0.05539	1.9	0.247	347.5	6.5	369.1	24.1	507.9	165.9	347.5	6.5	68.4

21AR238_465	748.1	14735.601	1.996	18.162	2.1	0.43475	3.0	0.05806	1.6	0.524	363.8	5.5	366.5	9.1	384.9	56.9	363.8	5.5	94.5
21AR238_466	100.2	14372.862	2.693	14.957	4.2	1.23685	4.6	0.13560	1.6	0.354	819.7	12.6	817.5	26.0	812.1	90.6	819.7	12.6	100.9
21AR238_467	50.2	2199.037	1.799	10.823	20.3	0.55648	22.1	0.04671	3.8	0.171	294.3	10.8	449.2	80.2	1348.0	421.4	294.3	10.8	21.8
21AR238_468	87.6	13157.161	1.750	9.581	3.1	3.39715	5.2	0.23738	4.1	0.784	1373.1	50.6	1503.7	41.0	1693.8	59.8	1693.8	59.8	81.1
21AR238_469	190.8	10448.752	2.259	17.218	6.1	0.47205	7.1	0.06019	2.6	0.365	376.8	9.5	392.6	23.2	487.9	146.3	376.8	9.5	77.2
21AR238_470	373.3	49612.126	4.631	12.982	1.8	1.71752	6.0	0.16182	5.7	0.950	966.9	50.9	1015.1	38.3	1121.5	37.1	1121.5	37.1	86.2
21AR238_471	105.0	6778.824	4.401	15.947	6.6	0.62110	7.3	0.07414	2.1	0.282	461.1	9.2	490.5	28.5	631.6	151.5	461.1	9.2	73.0
21AR238_472	373.8	9511.796	2.091	17.917	5.2	0.18390	6.2	0.02450	2.2	0.361	156.0	3.4	171.4	9.7	390.2	128.8	156.0	3.4	40.0
21AR238_473	136.6	10980.475	5.938	16.980	5.3	0.67808	8.6	0.08517	6.6	0.764	526.9	33.4	525.6	35.4	521.0	122.1	526.9	33.4	101.1
21AR238_474	174.4	24016.153	4.213	13.797	2.4	1.67982	3.4	0.16896	2.2	0.651	1006.4	20.7	1000.9	21.7	989.8	52.5	989.8	52.5	101.7
21AR238_475	275.1	6206.866	0.853	17.719	5.2	0.21271	5.9	0.02842	2.0	0.339	180.7	3.5	195.8	10.5	383.6	124.2	180.7	3.5	47.1
21AR238_476	431.0	36704.874	11.717	15.930	3.6	0.70065	8.8	0.08124	8.1	0.913	503.5	39.1	539.2	37.0	693.9	77.0	503.5	39.1	72.6
21AR238_477	171.9	15435.827	1.361	16.345	3.3	0.81619	3.8	0.09800	1.7	0.449	602.7	9.9	605.9	17.4	619.1	73.7	602.7	9.9	97.3
21AR238_478	217.7	9000.762	1.676	16.555	6.0	0.31855	6.9	0.03919	1.9	0.278	247.8	4.6	280.8	16.8	566.2	143.7	247.8	4.6	43.8
21AR238_479	167.9	13582.356	1.873	16.341	5.6	0.67774	6.0	0.08153	2.1	0.343	505.2	10.0	525.4	24.7	614.9	122.1	505.2	10.0	82.2
21AR238_480	30.6	5025.884	2.123	11.437	7.7	2.16670	10.6	0.18554	6.9	0.652	1097.2	69.6	1170.4	73.6	1309.4	155.8	1309.4	155.8	83.8
21AR238_481	467.4	34610.587	9.153	16.977	2.6	0.64264	3.2	0.07965	1.6	0.516	494.1	7.8	503.9	12.7	550.0	59.6	494.1	7.8	89.8
21AR238_482	143.2	27754.496	3.086	13.549	2.9	1.67185	3.6	0.16536	1.9	0.526	986.5	17.2	997.9	22.7	1023.9	61.6	1023.9	61.6	96.3
21AR238_483	423.0	18239.268	3.564	18.481	3.8	0.29098	4.9	0.03957	2.2	0.454	250.2	5.4	259.3	11.2	344.1	98.4	250.2	5.4	72.7
21AR238_484	106.3	4650.349	1.240	15.712	10.3	0.33197	13.6	0.03976	2.0	0.149	251.3	5.0	291.1	34.5	624.5	290.5	251.3	5.0	40.2
21AR238_485	226.2	355.016	1.786	3.960	38.0	0.07146	41.0	0.00235	10.9	0.267	15.2	1.7	70.1	27.8	2982.3	643.8	15.2	1.7	0.5
21AR238_486	256.5	6091.301	0.858	15.671	7.0	0.24413	9.7	0.02884	2.2	0.229	183.3	4.0	221.8	19.3	654.0	202.1	183.3	4.0	28.0
21AR238_487	120.5	3880.238	0.392	17.018	9.6	0.29918	11.3	0.03948	2.4	0.209	249.6	5.8	265.8	26.4	411.6	246.6	249.6	5.8	60.6
21AR238_488	555.4	5282.299	5.795	16.511	2.7	0.64151	7.4	0.08044	3.3	0.441	498.8	15.6	503.2	29.3	524.6	145.1	498.8	15.6	95.1
21AR238_489	189.9	8518.725	1.428	17.021	5.7	0.35082	6.4	0.04464	2.2	0.345	281.5	6.1	305.3	16.9	492.3	132.7	281.5	6.1	57.2
21AR238_490	276.2	20157.677	1.761	17.715	3.3	0.48268	4.6	0.06287	3.1	0.675	393.1	11.7	399.9	15.1	440.6	75.0	393.1	11.7	89.2
21AR238_491	865.2	19998.733	1.413	18.890	3.1	0.16863	4.0	0.02345	1.9	0.487	149.4	2.9	158.2	5.8	293.5	79.5	149.4	2.9	50.9
21AR238_492	509.4	46236.186	1.406	16.307	2.1	0.81519	3.0	0.09699	2.0	0.679	596.7	11.5	605.4	13.5	638.8	46.9	596.7	11.5	93.4
21AR238_493	253.7	17581.381	1.182	16.357	2.8	0.78495	3.5	0.09452	1.7	0.491	582.2	9.5	588.3	15.5	612.8	65.3	582.2	9.5	95.0
21AR238_494	227.6	26841.506	3.831	13.825	2.2	1.32902	6.0	0.13462	5.4	0.907	814.2	41.3	858.5	34.5	975.5	51.1	814.2	41.3	83.5
21AR238_495	310.9	21641.320	183.944	17.284	2.5	0.57033	3.4	0.07254	1.6	0.478	451.4	7.1	458.2	12.6	493.4	66.0	451.4	7.1	91.5
21AR238_496	40.6	6214.563	2.783	12.902	6.6	1.69804	7.8	0.16418	1.8	0.230	980.0	16.3	1007.8	50.0	1069.7	153.0	1069.7	153.0	91.6
21AR238_497	91.2	3793.252	2.359	14.644	10.3	0.34602	12.6	0.03903	2.3	0.183	246.8	5.6	301.7	32.9	752.6	261.6	246.8	5.6	32.8
21AR238_498	234.0	224.336	1.018	9.923	9.3	0.06884	51.6	0.01479	2.8	0.055	94.6	2.7	67.6	33.8	NA		94.6	2.7	
21AR238_499	357.4	13513.512	2.075	18.386	3.5	0.30200	4.5	0.04124	2.0	0.439	260.5	5.0	268.0	10.5	334.7	91.0	260.5	5.0	77.8
21AR238_500	107.3	3986.518	1.698	13.478	12.4	0.33583	13.8	0.03462	2.4	0.172	219.4	5.1	294.0	35.3	939.4	279.2	219.4	5.1	23.4
21AR238_501	324.6	25156.869	2.639	17.149	2.7	0.64882	3.6	0.08180	1.9	0.537	506.9	9.4	507.7	14.4	512.6	66.8	506.9	9.4	98.9
21AR238_502	427.4	17784.063	14.397	15.434	3.1	0.89339	7.4	0.10172	6.6	0.892	624.5	39.4	648.2	35.5	732.6	71.0	624.5	39.4	85.2
21AR238_503	132.9	21800.717	3.079	12.395	2.3	2.10387	3.2	0.19148	1.9	0.584	1129.4	19.3	1150.1	22.0	1190.2	51.2	1190.2	51.2	94.9
21AR238_504	276.3	26641.677	7.970	16.261	2.8	0.82341	3.4	0.09839	1.7	0.497	605.0	9.8	609.9	15.7	629.3	63.8	605.0	9.8	96.1
21AR238_505	336.0	33115.613	3.710	16.838	2.4	0.70020	3.4	0.08651	2.1	0.614	534.9	10.7	538.9	14.2	556.9	58.4	534.9	10.7	96.0
21AR238_506	158.3	37110.498	1.769	8.956	1.3	4.55698	2.0	0.29825	1.4	0.705	1682.6	20.4	1741.4	16.3	1813.6	25.1	1813.6	25.1	92.8
21AR238_507	216.9	14885.341	1.143	18.075	4.7	0.46086	5.3	0.06173	1.8	0.347	386.1	6.9	384.9	17.0	378.3	112.2	386.1	6.9	102.1
21AR238_508	143.6	27551.193	2.992	12.324	2.3	2.13997	3.0	0.19328	1.6	0.546	1139.1	17.0	1161.8	20.6	1205.2	49.2	1205.2	49.2	94.5
21AR238_509	151.3	15933.857	5.379	16.389	4.9	0.66456	5.4	0.08052	1.9	0.354	499.2	9.2	517.4	22.0	599.5	109.9	499.2	9.2	83.3
21AR238_510	201.5	20161.293	25.435	16.445	3.6	0.67974	7.5	0.08240	6.4	0.853	510.4	31.3	526.6	30.7	598.4	84.6	510.4	31.3	85.3
21AR238_511	201.9	8402.966	3.514	17.047	5.9	0.30892	7.9	0.03949	1.8	0.231	249.7	4.5	273.3	18.9	482.5	169.2	249.7	4.5	51.7
21AR238_512	861.9	21917.125	2.658	19.293	3.0	0.19356	4.1	0.02753	1.6	0.389	175.1	2.7	179.7	6.7	241.6	86.8	175.1	2.7	72.5
21AR238_513	377.5	44815.669	1.178	15.397	1.9	1.01446	4.0	0.11422	3.4	0.848	697.2	22.2	711.1	20.3	756.1	44.3	697.2	22.2	92.2
21AR238_514	110.8	11520.415	1.325	15.897	4.9	0.82357	6.2	0.09721	1.8	0.285	598.0	10.0	610.0	28.3	655.9	126.7	598.0	10.0	91.2
21AR238_515	472.0	15641.958	220.358	17.522	2.6	0.52510	4.1	0.06810	2.3	0.569	424.7	9.6	428.5	14.4	450.2	75.1	424.7	9.6	94.3
21AR238_516	132.8	8740.858	2.466	15.985	5.1	0.59514	7.2	0.07117	1.8	0.254	443.2	7.8	474.1	27.2	627.8	149.8	443.2	7.8	70.6
21AR238_517	87.1	10617.743	2.365	13.914	4.3	1.44824	4.7	0.14956	1.8	0.375	898.5	14.9	909.1	28.4	936.0	89.9	936.0	89.9	96.0
21AR238_518	294.9	18817.834	2.988	16.552	3.1	0.72619	4.0	0.08867	2.2	0.541	547.6	11.5	554.3	17.3	582.7	73.9	547.6	11.5	94.0
21AR238_519	425.6	5541.571	7.627	16.105	2.2	0.66870	10.6	0.08188	5.6	0.528	507.3	27.2	519.9	43.0	576.5	195.0	507.3	27.2	88.0
21AR238_520	212.0	36800.471	3.944	12.467	1.5	2.12133	2.3	0.19357	1.8	0.763	1140.7	18.4	1155.8	16.0	1185.0	29.6	1185.0	29.6	96.3
21AR238_521	185.5	9947.683	2.798	16.311	5.4	0.45731	6.4	0.05567	1.7	0.262	349.2	5.7	382.4	20.3	589.4	133.4	349.2	5.7	59.3
21AR238_522	385.6	40990.645	4.693	16.245	2.0	0.80925	3.0	0.09633	2.1	0.706	592.9	11.8	602.0	13.5	637.7	45.2	592.9	11.8	93.0
21AR238_523	443.3	40456.077	5.456	14.289	2.2	1.17608	7.4	0.12306	7.1	0.952	748.1	50.0	789.5	40.9	909.0	47.1	748.1	50.0	82.3
21AR238_524	652.6	7060.027	41.164	16.382	1.8	0.68497	2.6	0.08460	1.6	0.624	523.5	8.1	529.8	10.7	557.9	44.0	523.5	8.1	93.8
21AR238_525	384.9	14503.688	2.206	18.236	5.5	0.29651	6.4	0.04013	2.9	0.460	253.6	7.3	263.7	14.8	354.8	127.7	253.6	7.3	71.5
21AR238_526	103.4	1995.754	2.036	12.053	18.6	0.23183	20.5	0.02226	3.7	0.181	141.9	5.2	211.7	39.1	1083.3	404.5	141.9	5.2	13.1
21AR238_527	415.3	3871.616	3.277	17.648	3.9	0.30665	8.2	0.04216	1.5	0.186	266.2	4.0	271.6	19.6	319.4	183.5	266.2	4.0	83.3
21AR238_528	400.3	2364.159	1.164	16.994	4.1	0.28053	8.5	0.03874	2.1	0.252	245.0	5.1	251.1	18.8	309.3	186.6	245.0	5.1	79.2
21AR238_529	216.6	11103.984	2.888	18.014	5.6	0.35661	6.6	0.04784	2.1	0.314	301.2	6.1	309.7	17.5	374.8	140.5	301.2	6.1	80.4
21AR238_530	302.4	19714.613	1.321	17.532	3.1	0.56169	3.8	0.07253	1.9	0.502	451.4	8.4	452.6	14.0	459.8	73.7	451.4	8.4	98.2
21AR238_531	37.3	469.470	0.676	8.214	22.4	0.50342	55.3	0.03969	5.5	0.099	250.9	13.5	414.0	188.6	1468.2	1065.6	250.9	13.5	17.1
21AR238_532	150.9	5788.089	1.875	16.987	7.7	0.31354	8.6	0.04043	2.5	0.287	255.5	6.2	276.9	20.8	463.2	182.6	255.5	6.2	55.2
21AR238_533	631.4	5056.253	0.977	17.042	3.0	0.54467	8.8	0.07081	3.2	0.366	441.0	13.8	441.5	31.6	445.0	182.7	441.0	13.8	99.1
21AR238_534	242.0	16943.947	1.668	17.528	4.7	0.45813	5.4	0.05914	2.0	0.366	370.4	7.2	383.0	17.3	460.6	111.9	370.4	7.2	80.4
21AR238_535	102.2	5481.892	1.242	15.196	9.0	0.48486	11.0	0.05567	2.2	0.201	349.3	7.5	401.4	36.4	714.7	228.5	349.3	7.5	48.9
21AR238_536	76.4	2294.956	0.488	12.662	10.6	0.31648	19.7	0.03156	3.8	0.194	200.3	7.5	279.2	48.2	1007.2	393.7	200.3	7.5	19.9
21AR238_537	333.7	7737.659	1.547	17.817	3.7	0.42449	8.3	0.05678	2.4	0.296	356.0	8.5	359.3	25.0	381.1	177.2	356.0	8.5	93.4

* data corrected for common-Pb

[1]"Best Age" cutoff is 900 Ma, such that analyses with $^{206}Pb/^{238}U$ ages <900 Ma report $^{206}Pb/^{238}U$ Best Ages, and analyses with $^{206}Pb/^{238}U$ ages >900 Ma report $^{207}Pb/^{206}Pb$ Best Ages

[2]concordance calculated as (($^{206}Pb/^{238}U$ age)/($^{206}Pb^{*}/^{207}Pb^{*}$ age)) * 100

Supplementary Table S2: U-Pb data for Cerro Azul samples.

Table S3
Sources used in Figure 4

Tibetan Plateau Uplift

[1]Wang et al., 2008
[2]Yu and Guo, 2021
[3]Su et al., 2019
[4]Lu et al., 2016
[5]Miao et al., 2022
[6]DeCelles et al., 2011b
[7]Ding et al., 2022

C4 Ecosystems

[8]Quade et al., 1989
[9]Kohn, 2010
[10]Zhou et al., 2017
[11]Latorre et al., 1997
[12]Hynek et al., 2012
[13]Freeman and Colarusso, 2001
[14]An et al., 2005
[15]Passey et al., 2009
[16]Zhang et al., 2011

Benthic O Isotopes

[17]Westerhold et al., 2020

Sea Surface Temperatures

Sea surface temperatures were calculated using the approach of Herbert et al. (2016). Data across multiple sediment cores were stacked to yield temperature signatures for each zone.

Mid Lat NH (Mid Latitude Northern Hemisphere): ODP 1010; ODP 1021; ODP 1208; and Mediterranean.
Mid Lat SH (Mid Latitude Southern Hemisphere): DSDP 594; ODP 1088; ODP 1125; and ODP 1085.
Tropics: ODP 722; ODP 846; ODP 850; ODP 1241; and U1338.

[18]Herbert et al., 2016
[19]Rousselle et al., 2013
[20]Emeis et al., 2000
[21]Emeis et al., 2003
[22]Cleaveland and Herbert, 2009
[23]Herbert et al., 2015
[24]Tzanova et al., 2015
[25]Herbert et al., 2010
[26]Huang et al., 2007
[27]Liu and Herbert, 2004
[28]Lawrence et al., 2006
[29]Zhang et al., 2014
[30]Lawrence et al., 2009
[31]LaRiviere et al., 2012

[32]Rommerskirchen et al., 2011
[33]Fedorov et al., 2015
[34]Seki et al., 2012

Elevation Central Cordillera (~14°–27°S)

[35]Boschman et al., 2021
[36]Garzione et al., 2006
[37]Horton et al., 2001
[38]Scheuber et al., 2006
[39]Saylor and Horton, 2014
[40]Sundell et al., 2019
[41]DeCelles et al., 2011a
[42]Quade et al., 2015
[43]Horton et al., 2005
[44]Hoke and Garzione, 2008
[45]Carrapa and DeCelles, 2015

Elevation South-Central Cordillera (~27°–34°S)

[46]Giambiagi et al., 2016
[47]Martínez et al., 2015
[48]Levina et al., 2014,
[49]Giambiagi et al., 2017
[50]Bissig et al., 2002
[51]Hoke et al., 2014
[52]Ruskin and Jordan, 2007

Elevation Northern Patagonia (~34°–48°S)

[53]Mathiasen and Premoli, 2010
[54]Colwyn et al., 2019
[55]Chang et al., 2019
[56]Giambiagi et al., 2016
[57]Gutiérrez et al., 2013
[58]Fosdick et al., 2011

Supplementary References:

1. Wang C, et al. Constraints on the early uplift history of the Tibetan Plateau. *Proceedings of the National Academy of Sciences* **105**, 4987-4992 (2008).
2. Yu X, Guo Z. Surface uplift of the Tibetan Plateau: Constraints from isostatic effects of Cenozoic sedimentary accumulation. *Journal of Asian Earth Sciences* **208**, 104662 (2021). 3. Su T, et al. No high Tibetan plateau until the Neogene. *Science Advances* **5**, eaav2189 (2019).
4. Lu R, He D, Xu X, Liu B. Crustal-scale tectonic wedging in the central Longmen Shan: Constraints on the uplift mechanism in the southeastern margin of the Tibetan Plateau. *Journal of Asian Earth Sciences* **117**, 73-81 (2016).
5. Miao Y, et al. A new biologic paleoaltimetry indicating Late Miocene rapid uplift of northern Tibet Plateau. *Science* **378**, 1074-1079 (2022).
6. DeCelles P, Kapp P, Quade J, Gehrels G. Oligocene–Miocene Kailas basin, southwestern Tibet: Record of postcollisional upper-plate extension in the Indus-Yarlung suture zone. *Geological Society of America Bulletin* **12**3, 1337-1362 (2011).

7. Ding L, et al. Timing and mechanisms of Tibetan Plateau uplift. *Nature Reviews Earth & Environment* **3**, 652-667 (2022).
8. Quade J, Cerling TE, Bowman JR. Development of Asian monsoon revealed by marked ecological shift during the latest Miocene in northern Pakistan. *Nature* **342**, 163-166 (1989).
9. Kohn MJ. Carbon isotope compositions of terrestrial C3 plants as indicators of (paleo) ecology and (paleo) climate. *Proceedings of the National Academy of Sciences* **107**, 19691-19695 (2010).
10. Zhou B, et al. New sedimentary evidence reveals a unique history of C4 biomass in continental East Asia since the early Miocene. *Scientific Reports* **7**, 170 (2017).
11. Latorre C, Quade J, McIntosh WC. The expansion of C4 grasses and global change in the late Miocene: stable isotope evidence from the Americas. *Earth Planet Sci Lett* **146**, 83-96 (1997).
12. Hynek SA, Passey BH, Prado JL, Brown FH, Cerling TE, Quade J. Small mammal carbon isotope ecology across the Miocene–Pliocene boundary, northwestern Argentina. *Earth Planet Sci Lett* **321**, 177-188 (2012).
13. Freeman K, Colarusso L. Molecular and isotopic records of C4 grassland expansion in the late Miocene. *Geochim Cosmochim Acta* **65**, 1439-1454 (2001).
14. Zhisheng A, et al. Multiple expansions of C4 plant biomass in East Asia since 7 Ma coupled with strengthened monsoon circulation. Geology 33, 705-708 (2005).
15. Passey BH, et al. Strengthened East Asian summer monsoons during a period of high-latitude warmth? Isotopic evidence from Mio-Pliocene fossil mammals and soil carbonates from northern China. *Earth Planet Sci Lett* **277**, 443-452 (2009).
16. Zhang J, et al. Holocene monsoon climate documented by oxygen and carbon isotopes from lake sediments and peat bogs in China: a review and synthesis. *Quaternary Science Reviews* **30**, 1973-1987 (2011).
17. Westerhold T, et al. An astronomically dated record of Earth’s climate and its predictability over the last 66 million years. *Science* **369**, 1383-1387 (2020).
18. Herbert TD, Lawrence KT, Tzanova A, Peterson LC, Caballero-Gill R, Kelly CS. Late Miocene global cooling and the rise of modern ecosystems. *Nature Geoscience* **9**, 843-847 (2016).
19. Rousselle G, Beltran C, Sicre M-A, Raffi I, De Rafélis M. Changes in sea-surface conditions in the Equatorial Pacific during the middle Miocene–Pliocene as inferred from coccolith geochemistry. *Earth Planet Sci Lett* **361**, 412-421 (2013).
20. Emeis K-C, et al. Temperature and salinity variations of Mediterranean Sea surface waters over the last 16,000 years from records of planktonic stable oxygen isotopes and alkenone unsaturation ratios. *Palaeogeography, Palaeoclimatology, Palaeoecology* **158**, 259-280 (2000).
21. Emeis KC, et al. Eastern Mediterranean surface water temperatures and δ18O composition during deposition of sapropels in the late Quaternary. *Paleoceanography* **18**, (2003).
22. Cleaveland LC, Herbert TD. Preservation of the alkenone paleotemperature proxy in uplifted marine sequences: A test from the Vrica outcrop, Crotone, Italy. *Geology* **37**, 179-182 (2009).
23. Herbert TD, Ng G, Peterson LC. Evolution of Mediterranean sea surface temperatures 3.5–1.5 Ma: regional and hemispheric influences. *Earth Planet Sci Lett* **409**, 307-318 (2015).
24. Tzanova A, Herbert TD, Peterson L. Cooling Mediterranean Sea surface temperatures during the Late Miocene provide a climate context for evolutionary transitions in Africa and Eurasia. *Earth Planet Sci Lett* **419**, 71-80 (2015).
25. Herbert TD, Peterson LC, Lawrence KT, Liu Z. Tropical ocean temperatures over the past 3.5 million years. *Science* **328**, 1530-1534 (2010).

26. Huang Y, Clemens SC, Liu W, Wang Y, Prell WL. Large-scale hydrological change drove the late Miocene C4 plant expansion in the Himalayan foreland and Arabian Peninsula. *Geology* **35**, 531-534 (2007).
27. Liu Z, Herbert TD. High-latitude influence on the eastern equatorial Pacific climate in the early Pleistocene epoch. *Nature* **42**7, 720-723 (2004).
28. Lawrence KT, Liu Z, Herbert TD. Evolution of the eastern tropical Pacific through Plio-Pleistocene glaciation. *Science* **312**, 79-83 (2006).
29. Zhang YG, Pagani M, Liu Z. A 12-million-year temperature history of the tropical Pacific Ocean. *Science* **344**, 84-87 (2014).
30. Lawrence KT, Herbert TD, Brown CM, Raymo ME, Haywood AM. High-amplitude variations in North Atlantic sea surface temperature during the early Pliocene warm period. *Paleoceanography* **24**, (2009).
31. LaRiviere JP, et al. Late Miocene decoupling of oceanic warmth and atmospheric carbon dioxide forcing. *Nature* **486**, 97-100 (2012).
32. Rommerskirchen F, Condon T, Mollenhauer G, Dupont L, Schefuss E. Miocene to Pliocene development of surface and subsurface temperatures in the Benguela Current system. *Paleoceanography* **26**, (2011).
33. Fedorov AV, Burls NJ, Lawrence KT, Peterson LC. Tightly linked zonal and meridional sea surface temperature gradients over the past five million years. *Nature Geoscience* **8**, 975-980 (2015).
34. Seki O, Schmidt DN, Schouten S, Hopmans EC, Sinninghe Damsté JS, Pancost RD. Paleoceanographic changes in the Eastern Equatorial Pacific over the last 10 Myr. *Paleoceanography* **27**, (2012).
35. Boschman LM. Andean mountain building since the Late Cretaceous: A paleoelevation reconstruction. *Earth-Science Reviews* **220**, 103640 (2021).
36. Garzione CN, Molnar P, Libarkin JC, MacFadden BJ. Rapid late Miocene rise of the Bolivian Altiplano: Evidence for removal of mantle lithosphere. *Earth Planet Sci Lett* **241**, 543-556 (2006).
37. Horton B, Hampton B, Waanders G. Paleogene synorogenic sedimentation in the Altiplano plateau and implications for initial mountain building in the central Andes. *Geological Society of America Bulletin* **113**, 1387-1400 (2001).
38. Scheuber E, et al. Exhumation and basin development related to formation of the central Andean plateau, 21 S. *The Andes: Active Subduction Orogeny*, 285-301 (2006).
39. Saylor JE, Horton BK. Nonuniform surface uplift of the Andean plateau revealed by deuterium isotopes in Miocene volcanic glass from southern Peru. *Earth Planet Sci Lett* **387**, 120-131 (2014).
40. Sundell KE, Saylor JE, Lapen TJ, Horton BK. Implications of variable late Cenozoic surface uplift across the Peruvian central Andes. *Scientific Reports* **9**, 4877 (2019).
41. DeCelles PG, Carrapa B, Horton BK, Gehrels GE. Cenozoic foreland basin system in the central Andes of northwestern Argentina: Implications for Andean geodynamics and modes of deformation. *Tectonics* **30**, 6 (2011).
42. Quade J, et al. The growth of the central Andes, 22 S–26 S. *Geol Soc Am Mem* **212**, 277-308 (2015).
43. Horton BK. Revised deformation history of the central Andes: Inferences from Cenozoic foredeep and intermontane basins of the Eastern Cordillera, Bolivia. *Tectonics* **24**, 3 (2005).
44. Hoke GD, Garzione CN. Paleosurfaces, paleoelevation, and the mechanisms for the late Miocene topographic development of the Altiplano plateau. *Earth Planet Sci Lett* **271**, 192-201 (2008).
45. Carrapa B, DeCelles PG. Regional exhumation and kinematic history of the central Andes in response to cyclical orogenic processes. *Geol Soc Am Mem* **212**, 201-213 (2015).

46. Giambiagi L, et al. Cenozoic orogenic evolution of the southern central Andes (32–36 S). *Growth of the Southern Andes*, 63-98 (2016).
47. Martínez F, Arriagada C, Valdivia R, Deckart K, Peña M. Geometry and kinematics of the Andean thick-skinned thrust systems: Insights from the Chilean Frontal Cordillera (28°–28.5° S), Central Andes. *Journal of South American Earth Sciences* **64**, 307-324 (2015).
48. Levina M, Horton BK, Fuentes F, Stockli DF. Cenozoic sedimentation and exhumation of the foreland basin system preserved in the Precordillera thrust belt (31–32 S), southern central Andes, Argentina. *Tectonics* **33**, 9, 1659-1680 (2014).
49. Giambiagi L, et al. Cenozoic shift from compression to strike-slip stress regime in the high Andes at 30 S, during the shallowing of the slab: Implications for the El Indio/Tambo mineral district. *Tectonics* **36**, 11, 2714-2735 (2017).
50. Bissig T, Clark AH, Lee JK, Hodgson CJ. Miocene landscape evolution and geomorphologic controls on epithermal processes in the El Indio-Pascua Au-Ag-Cu belt, Chile and Argentina. *Economic Geology* **97**, 971-996 (2002).
51. Hoke GD, Giambiagi LB, Garzione CN, Mahoney JB, Strecker MR. Neogene paleoelevation of intermontane basins in a narrow, compressional mountain range, southern Central Andes of Argentina. *Earth Planet Sci Lett* **406**, 153-164 (2014).
52. Ruskin BG, Jordan TE. Climate change across continental sequence boundaries: paleopedology and lithofacies of Iglesia Basin, northwestern Argentina. *Journal of Sedimentary Research* **77**, 661-679 (2007).
53. Mathiasen P, Premoli AC. Out in the cold: genetic variation of Nothofagus pumilio (Nothofagaceae) provides evidence for latitudinally distinct evolutionary histories in austral South America. *Molecular Ecology* **19**, 371-385 (2010).
54. Colwyn DA, et al. Growth and steady state of the Patagonian Andes. *American Journal of Science* **319**, 431-472 (2019).
55. Chang Q, et al. Steady topography of Patagonian Andes through Cenozoic reconstructed by archives of precipitation hydrogen isotope composition. In: *AGU Fall Meeting Abstracts* (2019).
56. Gutiérrez NM, Hinojosa LF, Le Roux JP, Pedroza V. Evidence for an Early-Middle Miocene age of the Navidad Formation (central Chile): Paleontological, paleoclimatic and tectonic implications. *Andean Geology* **40**, 66-78 (2013).
57. Fosdick JC, Romans BW, Fildani A, Bernhardt A, Calderón M, Graham SA. Kinematic evolution of the Patagonian retroarc fold-and-thrust belt and Magallanes foreland basin, Chile and Argentina, 51 30′ S. *Geological Society of America Bulletin* **123**, 1679-1698 (2011).
